# Gene Mapping via Bulked Segregant RNA-Seq (BSR-Seq)

**DOI:** 10.1371/journal.pone.0036406

**Published:** 2012-05-07

**Authors:** Sanzhen Liu, Cheng-Ting Yeh, Ho Man Tang, Dan Nettleton, Patrick S. Schnable

**Affiliations:** 1 Department of Agronomy, Iowa State University, Ames, Iowa, United States of America; 2 Interdepartmental Genetics Graduate Program, Iowa State University, Ames, Iowa, United States of America; 3 Department of Statistics, Iowa State University, Ames, Iowa, United States of America; 4 Center for Plant Genomics, Iowa State University, Ames, Iowa, United States of America; University of Massachusetts Amherst, United States of America

## Abstract

Bulked segregant analysis (BSA) is an efficient method to rapidly and efficiently map genes responsible for mutant phenotypes. BSA requires access to quantitative genetic markers that are polymorphic in the mapping population. We have developed a modification of BSA (BSR-Seq) that makes use of RNA-Seq reads to efficiently map genes even in populations for which no polymorphic markers have been previously identified. Because of the digital nature of next-generation sequencing (NGS) data, it is possible to conduct *de novo* SNP discovery and quantitatively genotype BSA samples by analyzing the same RNA-Seq data using an empirical Bayesian approach. In addition, analysis of the RNA-Seq data provides information on the effects of the mutant on global patterns of gene expression at no extra cost. In combination these results greatly simplify gene cloning experiments. To demonstrate the utility of this strategy BSR-Seq was used to clone the *glossy3* (*gl3*) gene of maize. Mutants of the *glossy* loci exhibit altered accumulation of epicuticular waxes on juvenile leaves. By subjecting the reference allele of *gl3* to BSR-Seq, we were able to map the *gl3* locus to an ∼2 Mb interval. The single gene located in the ∼2 Mb mapping interval whose expression was down-regulated in the mutant pool was subsequently demonstrated to be the *gl3* gene via the analysis of multiple independent transposon induced mutant alleles. The *gl3* gene encodes a putative *myb* transcription factor, which directly or indirectly affects the expression of a number of genes involved in the biosynthesis of very-long-chain fatty acids.

## Introduction

Next generation sequencing (NGS) technologies [1]–[5] are revolutionizing biology much as PCR technologies did at the end of the last century. Adaptations of NGS technologies are proving to be superior to alternative technologies for genome-wide measurements of mRNA, small RNAs, transcription-factor binding sites, DNA methylation, chromatin structure and structural variation [1], [2], [6], [7].

The mapping of the genetic determinants of phenotypic variation is often a key step in the characterization of mutants and QTLs. In complex genomes mapping remains a non-trivial process. Bulked segregant analysis (BSA) is a method used to rapidly identify genetic markers linked to a genomic region associated with the selected phenotype [8]. Genetic linkage between markers and the causal gene is determined via quantification of allelic frequencies of genetic markers in the pools (bulks) of organisms that do and do not express a given phenotype. A wide variety of genetic markers have been used for BSA. The only requirement is that selected markers provide quantitative measurements of allelic frequencies. Examples include hybridization-based markers such as Restriction Fragment Length Polymorphisms (RFLPs) [8], Single Feature Polymorphisms (SFPs) [9], and Diversity Array Markers (DArTs) [10], as well as PCR-based methods such as Random Amplified Polymorphic DNAs (RAPDs) [11], Simple Sequence Repeats (SSRs, or microsatellites) [12]–[14], Amplified Fragment Length Polymorphisms (AFLPs) [15], [16], Cleaved Amplified Polymorphic Sequence (CAPS) for detecting Single Nucleotide Polymorphisms (SNPs) [17] and Sequenom SNP-typing [18]. More recently the efficiency of BSA has been greatly enhanced by the application of sequence-based markers such as restriction-site associated DNA (RAD) markers [19] and whole genome sequencing [20]. Both of these technologies fail to select against repetitive genomic sequences that are not typically useful in mapping experiments. In addition, even given the extraordinary advances in the throughput of NGS, whole genome sequencing remains expensive for large genomes, making it less suitable for mapping experiments that include many mutants.

One of the most widely adopted adaptations of NGS technology is RNA-Seq [21]–[27], which enables the comparative quantification of gene expression in, for example, various genotypes. RNA-Seq relies on the principle that read counts for each transcript from the NGS data reflects relative transcript concentrations. This relative quantification is reproducible and highly accurate [2], [25]–[28]. RNA-Seq reads can also be mined for DNA sequence polymorphisms such as single nucleotide polymorphisms (SNPs) [29], [30], which can be converted into genetic markers [18], [31]–[34].

We combined the power of BSA with the ease of RNA-Seq and appropriate statistical procedures into a new genetic mapping strategy called BSR-Seq (Bulked Segregant RNA-Seq). As a proof of concept, RNA-Seq was conducted on mutant and non-mutant pools of maize seedlings segregating for *gl3,* a recessive mutant involved in the accumulation of epicuticular wax [35], [36]. After quantifying allele frequency via read counts in RNA-Seq, a Bayesian-based BSA approach was developed to map *gl3*. The resulting mapping data were consistent with previous mapping results obtained via independent approaches. The mapping results were combined with transcriptional profiles from the RNA-Seq data to facilitate the cloning of *gl3*. The successful cloning of *gl3* demonstrates the utility of BSR-Seq.

## Results

**Figure 1:**
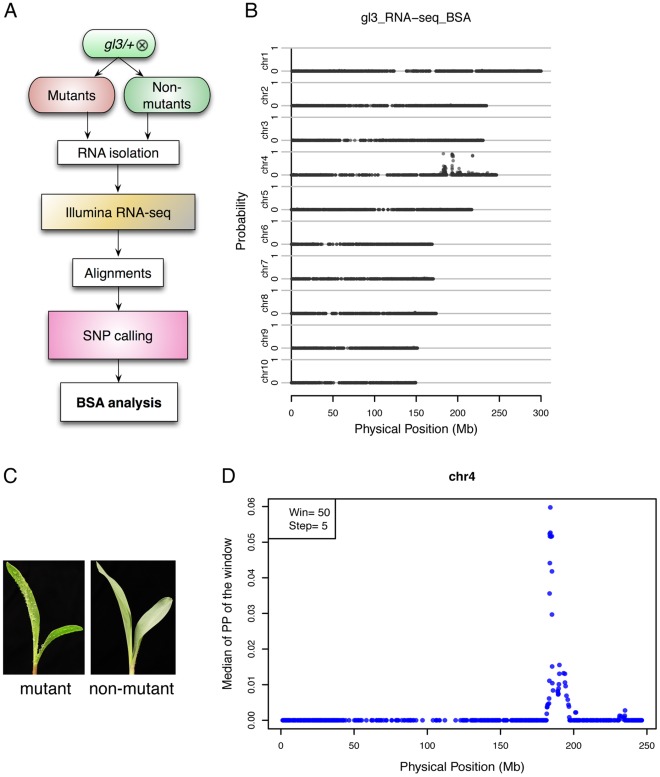
BSR-Seq. A. A flowchart of BSR-Seq experimental design. B. The physical position of each SNP marker (x-axis) was plotted versus the probability of each SNP marker being in complete linkage disequilibrium with the causal gene (y-axis). C. *gl3* mutants (the *gl3-ref* allele) express a glossy phenotype due to altered accumulation of epicuticular waxes. Water is sprayed on seedlings to distinguish mutant (*gl3-ref*/*gl3-ref*) from non-mutant (*gl3-ref*/+ or +/+). D. Chromosome 4 was scanned by using a window containing 50 SNPs with a step size of 5 SNPs. Within each window, the median linkage probability obtained from a Bayesian BSA analysis across all the 50 SNPs was determined and was plotted against the middle physical position of the window.

An individual heterozygous for the *gl3-ref* allele was self-pollinated to generate a segregating F_2_ population. Consistent with expectations based on the fact that the *gl3* mutant is recessive, ∼25% of the F_2_ seedlings expressed the mutant phenotype. RNA samples from mutant and non-mutant individuals from this F_2_ population were combined into two separate pools and subjected to RNA-Seq (Figure 1, Methods). One lane of an Illumina GAIIx flowcell was used for each of the two RNA samples; each lane yielded more than 13 million 75-bp single-end reads (Table S1). Reads that had been trimmed based on quality scores were mapped to the B73 maize reference genome using GSNAP that allows intron-spanning alignments (Methods). In total, 53.3% and 54.9% of the trimmed reads from the mutant and non-mutant pools were uniquely mapped to the reference genome, respectively. Of these uniquely mapped reads, 89.5–90.5% were located in high-confidence gene models [37]. In total, 76% (24,757/32,540) of the gene models had read(s) from at least one of the RNA-Seq datasets (Table S1).

### RNA-Seq of *gl3* vs. Non-mutant Siblings

An RNA-Seq analysis was conducted on the 16,726 maize genes for which at least 40 uniquely mapped reads were obtained from the combined mutant and non-mutant pools. In this analysis 1,095 genes were differentially expressed between the two pools (FDR = 0.1%; and having an absolute log2 fold change of >0.8; Figures S1–4; Tables S2 and S3).

### Mapping of *gl3* via BSR-Seq

To map the *gl3* gene, polymorphic SNPs that could serve as genetic markers were identified in the mapping population using the RNA-Seq data. By pooling RNA-Seq data from the two samples statistical power was increased and more than 64,000 high-confidence SNPs were discovered (Tables S4 and S5).

We next sought to identify SNPs that linked to the causal gene. In the absence of allele-specific expression (ASE), the two alleles of a given SNP site should be detected in approximately equal numbers of RNA-Seq reads when considering both pools of RNA-Seq data. Only one allele of a SNP that is completely linked to the causal gene should be present among the RNA-Seq reads from the mutant pool. In practice, however, as a consequence of ASE and sampling bias, particularly for genes that are expressed at low levels, only a single allele of many SNPs are detected in the mutant pool. To correct for these and other biological and technical artifacts, we used an empirical Bayesian approach to estimate linkage probability that is the probability of a SNP exhibiting complete linkage disequilibrium with the causal gene (Methods).

The linkage probability of each SNP was plotted against its physical coordinate in the reference genome as shown in Figure 1B. SNP markers having high probabilities of being linked to the *gl3* gene clustered on the long arm on chromosome 4. No SNP markers with high linkage probability were observed on any other chromosomes. This BSR-Seq-based localization of the *gl3* gene is consistent with previous mapping results obtained using independent approaches [18]. To narrow down the interval within which the *gl3* gene is located, chromosome 4 was scanned using a window of a fixed number of SNPs (N = 50) and a step size of 5 SNPs. The median linkage probability across the 50 SNPs of each window was plotted against the physical midpoint of each window (Figure 1D). A strong peak, indicating a high probability of complete linkage disequilibrium with the *gl3* gene, was observed at physical position ∼183–194 Mb of the B73 reference genome. The top 10 windows with the highest median linkage probability were located at physical position ∼183.5–185.2 Mb.

### The Cloning and Validation of *gl3*

**Figure 2:**
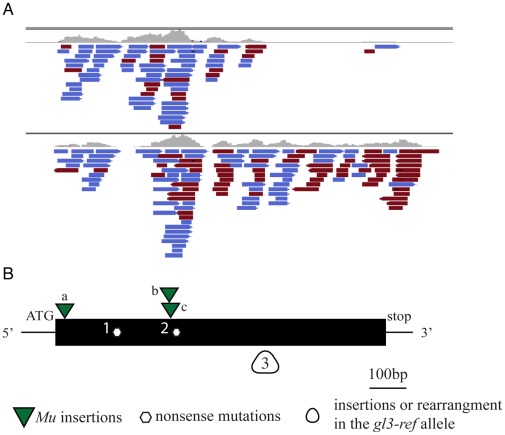
Gene structure of the *gl3* gene and lesions of its mutant alleles. A. RNA-Seq reads shown in the Integrative Genomics Viewer. Blue indicates reads that have a forward orientation relative to the reference genome; red indicates reverse orientation. B. Based on the supporting ESTs and the annotation from the gene models, the *gl3* gene contains only a single exon. All six lesions associated with *gl3* mutant alleles are located in the coding region. They include *Mu* insertion alleles (a: *gl3-93-4700-5*; b: *gl3-93-4700-6*; c: *gl3-93B111*), EMS alleles (1: *gl3-AEW-A632-363-EMS*, premature stop at position 171 nt in coding region (G->A); 2: *gl3-94-1001-326-EMS*, premature stop at position 358 nt in coding region (C–T)) and the reference allele (3: *gl3-ref*, insertion or rearrangement at 430–758 nt of the coding region).

To validate the mapping results from the BSR-Seq experiment we cloned the *gl3* gene. The high copy *Mu* transposon system is widely used as a mutagenic agent in forward genetic mutant screens of maize [38]. Using this transposon system we generated six additional *gl3* mutant alleles (Methods). The genomic sequences flanking the *Mu* transposons in maize stocks carrying each of these newly isolated *gl3-Mu* alleles were independently determined using the DLA-454 method [39]. We expected that most, if not all, of these independent alleles would contain a *Mu* transposon insertion site within the *gl3* gene. Among the 48 genes (4a.53 B73 filtered gene set) located in the ∼2 Mb interval identified by the BSR-Seq experiment, two genes contained ≥3 independent *Mu* insertions. Based on the RNA-Seq experiment, one of these genes, GRMZM2G162434, was significantly down-regulated in the mutant pool as compared to the non-mutant pool (Figure 2A). Using PCR primers specific to the inverted repeats of *Mu* transposons and to the *gl3* candidate gene (GRMZM2G162434) it was possible to amplify *Mu* insertions from three of the six *gl3* alleles derived from the forward *Mu* mutant screen. In addition, two previously identified EMS-induced alleles of *gl3* contain the typical EMS-induced G/C-to-A/T transitions [40] in GRMZM2G162434; both of these transitions generated premature stop codons (Figure 2B). Sequence analysis of the reference allele, *gl3-ref*, originally reported in 1928 [35], appears to contain a large insertion or other rearrangement between 430–758 nt of the coding region of GRMZM2G162434 that can not be PCR amplified (Figure 2B). Consistently, very few RNA-Seq reads were obtained 3′ of this region (Figure 2A).

Based on comparisons to the Arabidopsis and rice genomes, the *gl3* gene is predicted to encode an R2R3 type *myb* transcription factor [41] that contains two *Myb* DNA-binding domains. The GL3 protein is most similar to the Arabidopsis proteins MYB30 and MYB60. Consistent with the phenotype of the *gl3* mutant, the Arabidopsis *Myb30* gene regulates the biosynthesis of very-long-chain fatty acids [42], which are precursors to epicuticular waxes. Considering 30 maize candidate genes implicated in the accumulation of epicuticular waxes, including maize orthologs of Arabidopsis epicuticular wax genes (Methods), 22 accumulated at least 40 read counts across the two pools. Of these 22 genes, 6 are differentially expressed in the *gl3* mutant as compared to non-mutant siblings (3 up-regulated, 3 down-regulated) (Table S6).

Collectively, these results demonstrate that GRMZM2G162434 is the *gl3* gene. Because GRMZM2G162434 is located within the ∼2 Mb interval defined by the BSR-Seq experiment, these results demonstrate the utility of BSR-Seq for gene mapping and cloning.

## Discussion

### Advantages of BSR-Seq

Evolving NGS technologies are powerful tools for answering biological questions. For example, RNA-Seq is a highly accurate and robust approach for quantifying gene expression. Here we have reported a BSA-based mapping strategy (BSR-Seq) that relies on RNA-Seq data. For relatively small genomes that contain little repetitive DNA (e.g., *Caenorhabditis elegans* or Arabidopsis), it is feasible to conduct BSA using whole genome shotgun (WGS) sequencing [20], [43]. However, for mapping mutants in large genomes (e.g., maize and humans), WGS is not cost efficient. In these species BSR-Seq is both inexpensive and efficient.

BSR-Seq provides not only the map position of a gene responsible for a mutant phenotype but also the effects of such a mutant on global patterns of gene expression. The expression patterns of genes within the mapping interval can be used to prioritize candidate genes based on the fact that the causal gene will often be down-regulated in the mutant pool as compared to the non-mutant pool. In addition, this strategy yields a collection of polymorphic SNPs that are tightly linked to the mutant. These SNPs could be used to fine map the mutant or clone the affected gene via chromosome walking. Hence, BSR-Seq is not only an efficient strategy for mapping genes, but also yields other data that facilitate gene cloning.

### Potential Problems and Strategies for the Improvement

We have used BSR-Seq to successfully map five genes (unpublished results). The size of the mapping interval obtained from a BSR-Seq experiment depends on the number of individuals included in the mutant and non-mutant pools, the sequencing depth, and the density of polymorphisms in the mapping population. For each parameter more is better. In the reported proof-of-principle experiment designed to map the *gl3* locus, we included only ∼30 individuals in each pool and generated only one lane of GAIIx data for each pool (∼13 M reads/pool). Maize is a highly polymorphic species whose transcriptome contains >4 SNPs/kb [30]. Given these conditions it was possible to map the *gl3* gene to an interval of only a few megabases. In the *gl3* BSR-Seq experiment, the haplotype of either parent was not used for the BSR-Seq analysis.

The mutants used to map the *gl3* gene are fully recessive and easily distinguished from non-mutants. The accurate classification of mutant and non-mutant individuals is not always so straightforward. The inclusion of non-mutants in the mutant pool would negatively impact a BSR-Seq experiment. In our Bayesian analysis approach, a SNP is classified as having a high probability of being in complete linkage disequilibrium with the causal gene only if it is “fixed” in the mutant pool, i.e., the mutant pool contains only a single allele. This criterion is too strict if non-mutants are likely to be inadvertently included in the mutant pool as a consequence of misclassification errors. To adapt our approach for the existence of such errors, a gene could still be classified as having a high probability of being in complete linkage disequilibrium with the causal gene even if it exhibited some defined (but low) level of apparent recombination with the causal gene. The inadvertent inclusion of a small number of mutants in the non-mutant pool (as would be the case for an incompletely recessive mutant) is less likely to seriously impact mapping accuracy.

A mutant that influences the regulation of allele-specific expression [44]–[47] has the potential to generate false-positive SNPs in a BSR-Seq mapping experiment. To avoid these problems the second stage of our analysis focuses on only that subset of SNPs whose read counts in the mutant and non-mutant pools suggest that they co-localize with the causal gene (Methods). Using this two-stage approach, we did not observe any SNPs that incorrectly exhibited a high probability of linkage to the *gl3* gene outside of chromosome 4.

In principle, BSR-Seq could be extended to other applications, including the mapping of genes defined by dominant mutants and major QTL loci. In this case the analysis would, however, require some modifications. A related situation would be the mapping of a causal gene whose expression is influenced by genetic modifiers. We anticipate that BSR-Seq could be adapted to enable the simultaneous mapping of the causal gene and the modifiers.

One of the advantages of BSR-Seq is that it yields not only mapping data, but also information on the effects of the mutant on global patterns of gene expression. Of the 1,095 significantly differentially expressed genes in the RNA-Seq experiment, 446 were down-regulated and 633 were up-regulated in the *gl3* mutants as compared to their non-mutant siblings, yielding a ratio of down-regulated to up-regulated genes of 0.7 (Table S7). In contrast, considering only the genes on chromosome 4 (which contains the *gl3* gene) this ratio is 1.6, which is significantly higher than that of all other chromosomes (Pearson’s Chi-square test, χ^2^ = 22.11, df = 1, p-value = 2.58e−06). And within the 180–195 Mb interval of chromosome 4 that contains the *gl3* gene, this ratio is 3.3 (10∶3). This bias likely reflects the combined effects of downwardly biased read counts in gene linked to *gl3* due to polymorphisms in expressed genes in coupling with *gl3*. Hence, interpretation of differential gene expression within the mapping interval must be treated with caution.

During BSR-Seq, RNA-Seq reads are used for both the identification and quantification of SNPs and tests for differential gene expression. This BSR-Seq experiment made use of unreplicated RNA-Seq data. The lack of replication would not be expected to adversely affect the mapping results. On the other hand, to accurately identify differentially expressed genes it would be desirable to have replicated RNA-Seq data.

The decision of which tissue from which to collect RNA-Seq data does not seriously impact the genetic mapping function of BSR-Seq. It is not necessary that the causal gene be expressed in the samples used for BSR-Seq. This is because SNPs in all genes located near the causal gene and that are expressed in these samples can be used as markers to map the causal gene. On the other hand, one of the advantages of BSR-Seq over other mapping strategies is that it also has the potential to provide expression data. Hence, it would be ideal to extract the RNA from a tissue in which the causal gene is expressed. This would most likely be the case if a tissue in which the mutant phenotype is evident is selected for BSR-Seq.

Both WGS-BSA and BSR-Seq depend on access to a reference genome and both approaches are affected by the quality of that reference genome and the degree of structural variation within the species being analyzed. Mis-assemblies in the reference genome and copy number variation (CNV) between the genomes present in the mapping population and the reference genome could potentially negatively influence mapping success. Even so, although the B73 reference genome (version 1) contained a major assembly error in the vicinity of *gl3* (data not shown), and maize contains extremely high levels of CNV [48] we were able to successfully map *gl3* using BSR-Seq.

## Methods

### Genetic Materials

A plant carrying a *gl3-ref* allele in a non-B73 genetic background was crossed to the inbred line B73. The *gl3-ref* allele was obtained from Donald Robertson, Iowa State University [36]. A single progeny was self-pollinated to generate a segregating F_2_ population for use in the RNA-Seq experiment. Additional alleles were generated via direct *Mutator* transposon tagging experiments via Crosses 1: *Gl3*/*Gl3* (*Mu* stock) x *gl3-ref*/*gl3-ref.* Two existing EMS- (ethylmethane sulphonate) induced alleles of *gl3* (*gl3-AEW-A632-363-EMS* and *gl3-94-1001-326-EMS*) generated by Gerry Neuffer were used for verification of the candidate *gl3* gene. Confirmed *gl3* mutant alleles will be deposited in the maize genetics stock center.

### RNA Isolation and RNA-Seq

F_2_ seeds were grown at 25°C for six days (2-leaf stage), at which time the lower leaves of 32 mutants (*gl3-ref*/*gl3-ref*) and 31 non-mutant siblings (*gl3-ref/Gl3-B73* or *Gl3-B73/Gl3-B73*) were collected and separately pooled for RNA extraction (RNeasy mini kit, Qiagen, Chatsworth, CA) followed by treatment with DNase I. Sequencing libraries were constructed using the Illumina mRNA-Seq sample preparation kit (Solexa/Illumina, Catalog no. RS-100-0801). The resulting libraries were sequenced on an Illumina Genome Analyzer II with 75 cycles, resulting in 75 bp single end reads (GenBank accession no. SRA049037).

### Mapping RNA-Seq Reads

Raw RNA-Seq reads were trimmed to remove low-quality nucleotides via an in-house trimming script. GSNAP (Genomic Short-read Nucleotide Alignment Program, version 2010-03-09) [49], which allows gap alignment including intron-spanning alignment, was used to map trimmed reads to the B73 reference genomes (B73ref_v1) [37], the mitochondrial genome (Genbank acc#: AY506529.1) and the chloroplast genome (Genbank acc#: X86563.2). Reads that uniquely mapped to B73ref_v1 with ≤2 mismatches every 36 bp (a site with insertions or deletions was counted as a mismatch) were used for further analyses. The read number of each gene model (Refgen1, 4a.53) [37] was computed based on the coordinates of mapped reads. A read was counted if any portion of that read’s coordinates were included within a gene model.

### SNP Calling and Filtration

Sequence variants identified by GSNAP were further filtered to identify SNPs for BSR-Seq. The alignments of uniquely mapped reads passing the filtering criteria from the mutant and non-mutant data sets were merged for SNP discovery using the following rules. Validated SNP site must have two and only two SNP-types. Reads from these two SNP-types must account for ≥90% of the total reads that align to this SNP site. Each SNP-type must have ≥3 reads (quality score of SNP base ≥15) and the reads account for ≥20% of the total reads on that SNP site, which stringently controls for potential false SNP discovery derived from sequencing errors or paralogs. The SNP discovery pipeline is downloadable (http://schnablelab.plantgenomics.iastate.edu/software). In addition, we previously identified a set of genomic sites that are either sequencing errors in the B73 reference genome or paramorphisms [50] (data not shown). These sites were further filtered from discovered SNPs, followed by the allele-specific quantification on these filtered SNP sites for both mutants and non-mutants. To get a set of SNPs for the BSR-Seq analysis, at each SNP we required that at least five sequencing reads in both the mutant pool and the non-mutant pool and both alleles have at least one read in the non-mutant pool.

### Identification of SNP Markers Tightly Linked to the Mutant Gene

An empirical Bayesian approach was used to estimate, for each SNP, the conditional probability of no recombination between the SNP marker and the causal gene in the mutant pool, given the SNP allele-specific counts.

Using Bayes’ theorem, we can write this conditional probability as
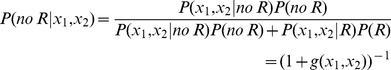
(1)where “no R” and “R” denote no recombination and recombination, respectively, between the SNP and the causal gene in the mutant pool; *x_1_* and *x_2_* denote the SNP allele counts in the mutant pool; and



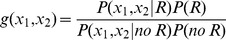
(2)In equations (1) and (2), 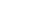 is the prior probability that there is no recombination between a randomly selected SNP and the causal gene in the mutant pool, and 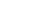 is the prior probability that there is recombination between a randomly selected SNP and the causal gene in the mutant pool. Let *d* denote the distance in Morgans between any randomly selected SNP and the causal gene. If we assume a uniform prior distribution across the genome for the causal gene location, it follows that the prior for *d* is uniform for values of *d* near zero. For a given distance *d*, we use Haldane’s mapping function to compute the prior probability of no recombination between the SNP and the causal gene as
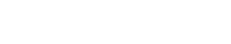
where *N* denotes the number of plants in the mutant pool. Note that 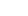 quickly converges to zero as *d* moves away from zero. Thus, we can find the approximate expected value of 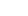 by integrating the product of 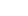 and the prior density of 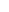 in a neighborhood of zero (0 to 20 cM, for example). This expected value serves as 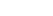 the prior probability of no recombination between the SNP and the causal gene in the mutant pool. The prior probability of some recombination between a randomly selected SNP and the causal gene is given by 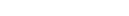.

Next consider 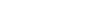, the conditional probability of the allele counts at a SNP in the mutant pool, given no recombination between the SNP and the causal gene in the mutant pool. Because each plant in the mutant pool contains two copies of the mutant allele and zero copies of the wildtype allele, no recombination implies that all SNP alleles coupled with the mutant allele. Thus, 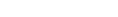 if 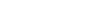
and 0 otherwise, where 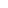 denotes the total number of reads for the SNP. By expression (1), it is clear that 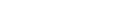 if 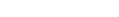 i.e., if 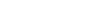.

The final probability needed for the computation of (1) is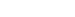. To compute the probability, we condition on the total number of reads for the SNP 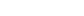 and assume that 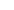 has a binomial distribution with 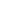 trials and success probability 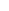. Given that there is some recombination between the SNP and the causal gene, we know that it is possible for RNA sequences in the mutant pool to contain both SNP alleles. However, we cannot know precisely the relative probability of each allele. In other words, the success probability 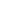 is unknown and likely to vary from SNP to SNP. Thus, we require a prior distribution for 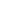 to provide an adequate representation of the possible values for the relative frequency of each allele. While a variety of choices are possible, we choose to take advantage of the large quantity of SNP data in the non-mutant pool to generate an empirical prior distribution. In particular, we use the observed non-zero relative frequencies of alleles at each SNP in the non-mutant pool to obtain the prior distribution for 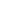. It is then straightforward to compute 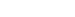 as the expected value of 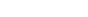 with respect to this empirical prior distribution.

Once all the component probabilities have been obtained, it is straightforward to compute
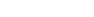
for each SNP in the mutant pool. These posterior probabilities can then be used to identify regions likely to contain the causal gene. However, the computation of these posterior probabilities makes little use of the data from the non-mutant pool. We can gain additional information about the likely location of the causal gene by more fully utilizing the non-mutant data.

In traditional BSA, DNA sequences that completely linked to the causal gene are expected to exhibit 1∶2 ratio of mutant to wildtype alleles in the non-mutant pool. However, in BSR-Seq allele frequencies are measured at the RNA level. There is no guarantee that the relative frequency observed in RNA-Seq reads will match the relative frequency at the DNA level. In addition, the read counts themselves are subject to biological and technical variation. To address these issues, we use the RNA-Seq data to estimate, for each SNP, 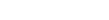, where 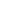 is the proportion of a mutant allele read at the SNP in the non-mutant pool, 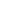 is the observed number of reads in the non-mutant pool that match the mutant allele, and 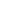 is the total number of reads at the SNP in the non-mutant pool. The value of 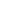 is selected here because we believe it is reasonable to assume that the mutant allele will be less probable than the wildtype allele for a SNP near the causal gene in the non-mutant pool, given that the expected relative frequency of the mutant allele at the DNA level is only half that of the wildtype allele.

To compute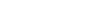, we again use Bayes theorem to obtain
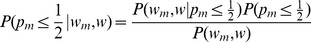
(3)


The identity of the mutant allele is determined by the most frequent allele in the mutant pool. This will be correct for SNPs near the causal gene and irrelevant for other SNPs that will be ruled out as candidates by our calculation of 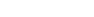. As in our calculation of 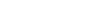, we use the observed relative frequencies of alleles at each SNP in the non-mutant pool to obtain a prior distribution for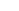. Each of the probabilities in (3) can be easily obtained by computing expectation with respect to this empirical prior.

Once we have computed 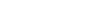 and 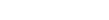 for each SNP, we compute the product of these probabilities (final probabilities) for each SNP to identify SNPs likely to be tightly linked to the causal gene. Only SNPs with high values of both 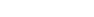 and 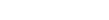 will have a large product. SNPs for which either 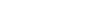
or 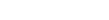 is small will be ruled out.

To obtain the genomic region(s) that are more likely linked to the causal gene, we scanned the whole genome by sliding windows with fixed number of SNPs (N = 50) and with a step size of 5 SNPs. In each window, a median of the final probabilities of all the SNPs was determined as the “window linkage probability”. The windows with the highest “window linkage probability” are the regions close to the causal genes.

#### Identify differentially expressed genes via Fisher’s exact test

The Fisher’ exact test was used to test the null hypothesis that the proportions of reads of a given gene among the total reads uniquely mapped to the reference genome are not different between the mutants and the non-mutants. Only genes with at least 40 total reads from both genotypes were used to perform the Fisher’ exact test. Absolute value of log2 mutant/non-mutant fold change greater than 0.8 was used to further filter DE genes. The transcripts were quantified by using normalized read counts plus 1. The total number of uniquely mapped reads of each data set was used for the normalization. The significantly expressed genes were obtained with the additional false discovery control (false discovery rate, FDR = 0.1%) to account for multiple tests [51]. Because this comparison did not include biological replication, statistically significant variation can be a consequence of either biological or technical variation in gene expression between the two samples.

### Identification of Maize Genes Involved in the Epicuticular Wax Pathway

The protein sequences of 13 Arabidopsis genes involved in biosynthesis and secretion of plant cuticular wax were BLAST to the maize protein database. The BLAST alignments of E-value < e−50, >50% identity and >30% coverage were extracted. For each Arabidopsis gene, we kept at most three best hits. Three Arabidopsis genes (wsd1, mah1 and cer2) did not have homologs identified in maize with the criteria. The best hit was extracted as the homologous gene of each of these three. In total, 25 maize homologous candidates were identified. With adding cloned maize glossy genes that are not in the Arabidopsis homologous list, 30 maize genes that might involve in the epicuticular wax pathway were obtained.

## Supporting Information

Figure S1 Histogram of p-values for differential expression tests. Fisher’s exact test was used to test the null hypothesis that expression of a given gene is not different between the two groups. A p-value was obtained for each informative gene. The distribution of p-values under the null hypothesis (no differential genes existed) is a uniform distribution in the range of 0–1. More than the expected number of p-values with small values indicates significantly differentially expressed genes could be statistically identified.
(DOC)

Figure S2 MA-similar plot. The MA-similar plot provides an overview of the differential level between groups of the comparison. Log2 fold change (y-axis) of each informative gene was plotted against log2 of mean of expression (x-axis). Significantly differentially expressed genes are highlighted in red.
(DOC)

Figure S3 Volcano plot. The volcano plot compares gene expression patterns between two groups. Negative log10 p-values from the differential expression test were plotted against the log2 fold change for each informative gene. Each dot represents a gene, plotting with 20% transparency. The horizontal dash line indicates the 0.1% FDR cutoff. The vertical green lines indicate the cutoffs of log2 wildtype/mutant ratios equaling to -0.8 and 0.8.
(DOC)

Figure S4 Overview of differential expression in the metabolic pathway in MapMan. MapMan (mapman.gabipd.org) provides a useful tool to visualize the alteration of gene expression in the comparison. Differential expression in the metabolic pathway was shown as an example. Each square represents a transcript. The squares were color-coded by log2 fold change between the *gl3* non-mutant pool and the mutant pool from the RNA-Seq data. The up- and down-regulated genes in the mutant pool relative to the non-mutant pool were highlighted in red and blue, respectively. More pathways can be explored by feeding the data of Table S3 to the MapMan software.
(DOC)

Table S1 Summary of RNA-Seq data and alignments. (DOC)

Table S2 Result of differential expression tests. Table S2 provides the detailed differential expression test result of genes (4a53). Test was performed on the informative genes that are those genes with at least 20 average reads across the two samples. Description of each column in this table: • **GeneID:** gene ID; • **Ref:** version of the reference genome; • **Chr:** chromosome; • **Ori:** gene orientation (either forward (+) or reverse (−) strand); • **Start:** the first physical position of the gene on the chromosome; • **End:** the last physical position of the gene on the chromosome; • **ExonSize:** total length of all the gene’s annotated exons; • **gl3mut:** raw read counts for the gene of the sample of the gl3 mutant pool; • **gl3wt:** raw read counts for the gene of the sample of the gl3 non-mutant pool; • **gl3mut.RPKM:** normalized read counts of a given gene (“RPKM” means reads per kb exonic sequence per million uniquely mapped reads) in the sample of the gl3 mutant pool; • **gl3wt.RPKM:** normalized read counts of a given gene in the sample of the gl3 non-mutant pool; • **gl3wt:mut_log2FC:** log2 of fold change between the gl3 non-mutant pool and the mutant pool; • **gl3wt:mut_pvalue:** p_value of the statistical test for differential expression of this gene between the gl3 non-mutant pool and the mutant pool; • **gl3wt:mut_qvalue:** the corrected p-values (q_values) for differential expression of this gene after correcting for multiple testing; • **gl3wt:mut_sig:** the answer to the question “Is this gene significantly expressed?” The gene with the q_value smaller than 0.001 (FDR 0.1%) and having an absolute log2 fold change of >0.8 was labeled with “yes”. • **description:** description of genes.
(XLS)

Table S3 The expression test data to feed MapMan for the pathway visualization. Table S3 contains two columns. The first column is the transcript name; the second column is the value of log2(fold-change). The transcripts without significantly differential expression were assigned a value of zero.
(DOC)

Table S4 Summary of SNP discovery. (DOC)

Table S5 Allele counts in the mutant pool and the non-mutant pool. Table S5 provides the read counting information for each of both alleles in the mutant and the non-mutant.
(XLS)

Table S6 Expression summary of genes involved in the biosynthesis of very-long-chain fatty acids. (DOC)

Table S7 Number of differentially expressed genes in each chromosome. (DOC)
