# Supplementary material for: Gene Mapping via Bulked Segregant RNA-Seq (BSR-Seq)
Source: PLoS One. 2012 May 7;7(5):e36406. doi: 10.1371/journal.pone.0036406 (PMC3346754; doi:10.1371/journal.pone.0036406)
Supplement: Figure S1 — Fisher’s exact test was used to test the null hypothesis that expression of a given gene is not different between the two groups. A p-value was obtained for each informative gene. The distribution of p-values under the null hypothesis (no differential genes existed) is a uniform distribution in the range of 0–1. More than the expected number of p-values with small values indicates significantly differentially expressed genes could be statistically identified. (DOC) [file pone.0036406.s001.doc]

**Figure S1:** Histogram of p-values for differential expression tests

Fisher’s exact test was used to test the null hypothesis that expression of a given gene is not different between the two groups. A p-value was obtained for each informative gene. The distribution of p-values under the null hypothesis (no differential genes existed) is a uniform distribution in the range of 0-1. More than the expected number of p-values with small values indicates significantly differentially expressed genes could be statistically identified.
