# Supplementary material for: Gene Mapping via Bulked Segregant RNA-Seq (BSR-Seq)
Source: PLoS One. 2012 May 7;7(5):e36406. doi: 10.1371/journal.pone.0036406 (PMC3346754; doi:10.1371/journal.pone.0036406)
Supplement: Figure S2 — The MA-similar plot provides an overview of the differential level between groups of the comparison. Log2 fold change (y-axis) of each informative gene was plotted against log2 of mean of expression (x-axis). Significantly differentially expressed genes are highlighted in red. (DOC) [file pone.0036406.s002.doc]

**Figure S2:** MA-similar plot

The MA-similar plot provides an overview of the differential level between groups of the comparison. Log2 fold change (y-axis) of each informative gene was plotted against log2 of mean of expression (x-axis). Significantly differentially expressed genes are highlighted in red.
