# Supplementary material for: Gene Mapping via Bulked Segregant RNA-Seq (BSR-Seq)
Source: PLoS One. 2012 May 7;7(5):e36406. doi: 10.1371/journal.pone.0036406 (PMC3346754; doi:10.1371/journal.pone.0036406)
Supplement: Figure S3 — The volcano plot compares gene expression patterns between two groups. Negative log10 p-values from the differential expression test were plotted against the log2 fold change for each informative gene. Each dot represents a gene, plotting with 20% transparency. The horizontal dash line indicates the 0.1% FDR cutoff. The vertical green lines indicate the cutoffs of log2 wildtype/mutant ratios equaling to -0.8 and 0.8. (DOC) [file pone.0036406.s003.doc]

**Figure S3:** Volcano plot

The volcano plot compares gene expression patterns between two groups. Negative log10 p-values from the differential expression test were plotted against the log2 fold change for each informative gene. Each dot represents a gene, plotting with 20% transparency. The horizontal dash line indicates the 0.1% FDR cutoff. The vertical green lines indicate the cutoffs of log2 wildtype/mutant ratios equaling to -0.8 and 0.8.
