# Supplementary material for: Gene Mapping via Bulked Segregant RNA-Seq (BSR-Seq)
Source: PLoS One. 2012 May 7;7(5):e36406. doi: 10.1371/journal.pone.0036406 (PMC3346754; doi:10.1371/journal.pone.0036406)
Supplement: Figure S4 — MapMan (mapman.gabipd.org) provides a useful tool to visualize the alteration of gene expression in the comparison. Differential expression in the metabolic pathway was shown as an example. Each square represents a transcript. The squares were color-coded by log2 fold change between the gl3 non-mutant pool and the mutant pool from the RNA-Seq data. The up- and down-regulated genes in the mutant pool relative to the non-mutant pool were highlighted in red and blue, respectively. More pathways can be explored by feeding the data of Table S3 to the MapMan software. (DOC) [file pone.0036406.s004.doc]

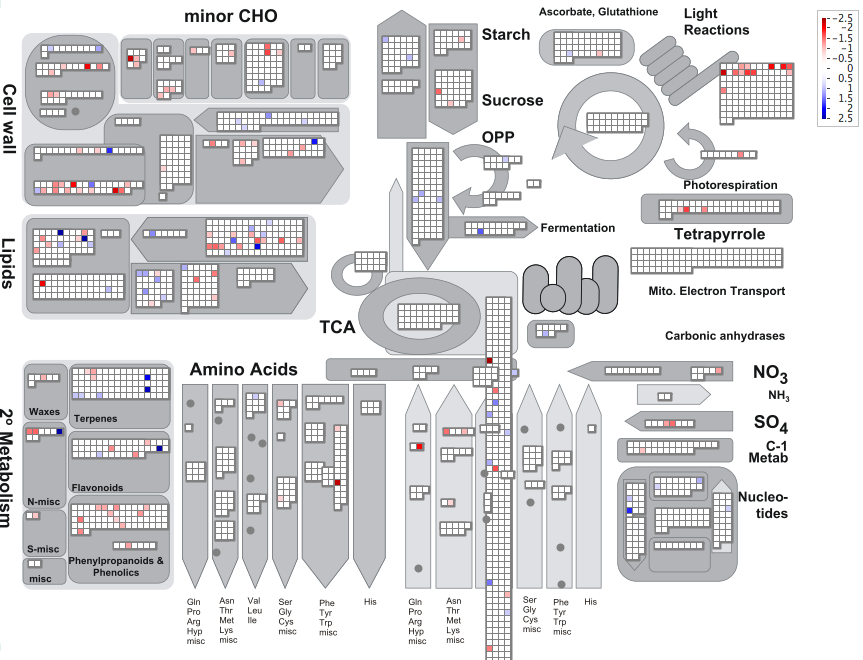


**Figure S4:** Overview of differential expression in the metabolic pathway in MapMan

MapMan (mapman.gabipd.org) provides a useful tool to visualize the alteration of gene expression in the comparison. Differential expression in the metabolic pathway was shown as an example. Each square represents a transcript. The squares were color-coded by log2 fold change between the *gl3* non-mutant pool and the mutant pool from the RNA-Seq data. The up- and down-regulated genes in the mutant pool relative to the non-mutant pool were highlighted in red and blue, respectively. More pathways can be explored by feeding the data of Table S3 to the MapMan software.
