# Supplementary material for: Gene Mapping via Bulked Segregant RNA-Seq (BSR-Seq)
Source: PLoS One. 2012 May 7;7(5):e36406. doi: 10.1371/journal.pone.0036406 (PMC3346754; doi:10.1371/journal.pone.0036406)
Supplement: Table S1 — (DOC) [file pone.0036406.s005.doc]

Table S1. Summary of RNA-Seq data and alignments

| **Sample** | **No. individuals in the pool** | **No. Reads** | **No. Reads (%, trimmed/raw)** | **Uniquely aligned (%, unique/trimmed)** | **Unique Reads in Gene Space (%, gene/unique)** |
| --- | --- | --- | --- | --- | --- |
| mutants | 32 | 13,700,533 | 12,526,899 (91.4%) | 6,672,038 (53.3%) | 6,037,550 (90.5%) |
| non-mutants | 31 | 13,078,023 | 11,945,873 (91.3%) | 6,561,160 (54.9%) | 5,874,438 (89.5%) |
