# Supplementary material for: Gene Mapping via Bulked Segregant RNA-Seq (BSR-Seq)
Source: PLoS One. 2012 May 7;7(5):e36406. doi: 10.1371/journal.pone.0036406 (PMC3346754; doi:10.1371/journal.pone.0036406)
Supplement: Table S3 — contains two columns. The first column is the transcript name; the second column is the value of log2(fold-change). The transcripts without significantly differential expression were assigned a value of zero. (DOC) [file pone.0036406.s007.doc]

Table S3. The expression test data to feed MapMan for the pathway visualization

Table S3 contains two columns. The first column is the transcript name; the second column is the value of log2(fold-change). The transcripts without significantly differential expression were assigned a value of zero.

Transcript gl3wt:mut_log2FC

ac147602.5_fgt003 0

ac147602.5_fgt004 0

ac147602.5_fgt005 0

ac148152.3_fgt005 -0.92

ac148152.3_fgt008 0

ac148167.6_fgt001 0

ac149475.2_fgt002 0

ac149475.2_fgt003 0

ac149475.2_fgt005 0

ac149475.2_fgt007 0

ac149633.4_fgt002 0

ac149633.4_fgt005 0

ac149810.2_fgt008 0

ac149818.2_fgt001 0

ac149818.2_fgt006 0

ac149818.2_fgt008 0

ac149828.2_fgt002 0

ac149829.2_fgt004 0

ac149829.2_fgt006 0

ac150739.3_fgt001 0

ac152495.1_fgt002 1.57

ac152495.1_fgt010 0

ac152495.1_fgt018 0

ac155376.2_fgt005 0

ac155377.1_fgt001 0

ac155377.1_fgt002 0

ac155417.2_fgt009 0

ac155434.2_fgt001 0

ac155434.2_fgt004 0

ac155434.2_fgt006 0

ac155496.2_fgt008 0

ac155610.2_fgt004 0

ac155610.2_fgt007 -1.31

ac155622.2_fgt001 0

ac155624.2_fgt006 0

ac155624.2_fgt007 0

ac155624.2_fgt010 0

ac155624.2_fgt011 0

ac159612.1_fgt007 0

ac166636.1_fgt007 0

ac177870.3_fgt006 0

ac177894.2_fgt007 0

ac177897.2_fgt011 0

ac177899.2_fgt009 0

ac177908.3_fgt002 0

ac177924.2_fgt003 0

ac177946.2_fgt004 0

ac182418.6_fgt009 0

ac182418.6_fgt010 0

ac182482.3_fgt003 0

ac182617.3_fgt001 0

ac183315.4_fgt006 0

ac183932.3_fgt007 0

ac184839.3_fgt002 0

ac185226.4_fgt001 0

ac185300.4_fgt002 0

ac185415.3_fgt005 0

ac185421.3_fgt001 0

ac185444.3_fgt003 0

ac185467.3_fgt002 0

ac185474.3_fgt006 0

ac185612.3_fgt001 0

ac186147.3_fgt008 -1.68

ac186166.3_fgt008 0

ac186187.3_fgt009 -1.36

ac186231.4_fgt002 0

ac186319.4_fgt002 0

ac186337.3_fgt004 0

ac186433.4_fgt002 0

ac186661.3_fgt003 0

ac186795.3_fgt006 0

ac187098.4_fgt011 0

ac187154.5_fgt003 0

ac187157.4_fgt002 0

ac187157.4_fgt004 0

ac187157.4_fgt005 0

ac187551.3_fgt002 0

ac188023.3_fgt005 0

ac188752.3_fgt002 0

ac188756.2_fgt001 0

ac188759.3_fgt005 0

ac188981.3_fgt003 0

ac189677.4_fgt001 0

ac189750.4_fgt004 -1.23

ac189879.3_fgt003 0

ac190609.3_fgt002 0

ac190609.3_fgt003 0

ac190609.3_fgt004 0

ac190623.3_fgt001 0

ac190628.4_fgt007 0

ac190750.2_fgt014 0

ac190788.2_fgt006 0

ac190801.3_fgt007 0

ac190838.3_fgt004 0

ac190982.3_fgt008 0

ac190999.2_fgt004 0

ac191009.3_fgt004 0

ac191097.3_fgt007 0

ac191113.2_fgt002 0

ac191116.3_fgt001 0

ac191246.3_fgt006 0

ac191283.3_fgt004 0

ac191285.3_fgt001 0

ac191387.3_fgt008 0

ac191387.3_fgt009 0

ac191418.3_fgt006 1.71

ac191567.3_fgt004 0

ac191628.3_fgt002 0

ac191687.3_fgt005 0

ac191707.3_fgt002 0

ac191719.3_fgt002 0

ac192244.3_fgt001 0

ac192244.3_fgt007 0

ac192244.3_fgt008 0

ac192283.3_fgt005 0

ac192362.3_fgt003 0

ac192451.3_fgt001 0

ac193379.3_fgt001 0

ac193475.3_fgt008 0

ac193632.2_fgt002 0

ac194015.3_fgt003 0

ac194022.3_fgt013 0

ac194131.3_fgt001 0

ac194219.3_fgt001 0

ac194272.3_fgt003 0

ac194341.4_fgt002 0

ac194363.3_fgt005 0

ac194365.3_fgt002 0

ac194381.3_fgt011 0

ac194389.3_fgt001 0

ac194409.3_fgt005 0

ac194461.3_fgt004 0

ac194671.1_fgt004 0

ac194914.3_fgt002 0

ac194965.4_fgt001 0

ac194965.4_fgt002 0

ac194965.4_fgt004 0

ac194970.5_fgt001 -0.97

ac194970.5_fgt003 0

ac194970.5_fgt004 0

ac194970.5_fgt005 0

ac194970.5_fgt009 0

ac195154.3_fgt004 0

ac195235.3_fgt010 0

ac195340.3_fgt001 0

ac195340.3_fgt002 0

ac195340.3_fgt004 0

ac195347.4_fgt004 0

ac195366.3_fgt002 0

ac195395.3_fgt003 0

ac195864.3_fgt016 0

ac195874.2_fgt002 0

ac195940.2_fgt003 0

ac196053.3_fgt002 0

ac196059.3_fgt003 0

ac196066.3_fgt004 0

ac196090.3_fgt003 1.09

ac196090.3_fgt006 0

ac196125.3_fgt002 0

ac196156.3_fgt003 0

ac196161.3_fgt008 0

ac196268.3_fgt003 0

ac196292.3_fgt004 0

ac196426.3_fgt007 0

ac196433.2_fgt004 0

ac196475.3_fgt004 0

ac196489.3_fgt002 0

ac196971.3_fgt002 0

ac196978.5_fgt001 0

ac197056.3_fgt005 0

ac197118.3_fgt005 -0.89

ac197122.3_fgt003 0

ac197145.4_fgt003 0

ac197146.3_fgt002 0

ac197146.3_fgt003 0

ac197229.3_fgt004 0

ac197246.3_fgt001 0

ac197246.3_fgt002 -0.88

ac197246.3_fgt003 0

ac197264.3_fgt005 0

ac197545.3_fgt002 0

ac197555.3_fgt008 0

ac197578.4_fgt003 0

ac197672.3_fgt002 0

ac197699.3_fgt003 0

ac197705.4_fgt006 0

ac197705.4_fgt007 0

ac197705.4_fgt011 0

ac197717.3_fgt002 0

ac197779.3_fgt001 0

ac197847.3_fgt004 0

ac198131.4_fgt008 0

ac198169.4_fgt004 0

ac198175.3_fgt003 0

ac198206.5_fgt001 0

ac198353.5_fgt001 0

ac198353.5_fgt004 0

ac198361.3_fgt004 0

ac198518.3_fgt003 0

ac198518.3_fgt004 0

ac198608.3_fgt003 0

ac198725.4_fgt007 0

ac198937.4_fgt003 0

ac198937.4_fgt004 0

ac198937.4_fgt006 0

ac199054.3_fgt004 0

ac199173.3_fgt006 0

ac199193.3_fgt003 0

ac199315.4_fgt001 0

ac199363.4_fgt003 0

ac199364.3_fgt001 0

ac199371.3_fgt001 0

ac199526.5_fgt002 0

ac199526.5_fgt003 0

ac199526.5_fgt004 0

ac199539.3_fgt006 0

ac199541.4_fgt001 0

ac199633.4_fgt001 0

ac199705.3_fgt008 0

ac199768.4_fgt006 0

ac199782.5_fgt001 0

ac199782.5_fgt002 0

ac199858.4_fgt002 0

ac199873.4_fgt004 0

ac199922.3_fgt003 0.94

ac200057.4_fgt007 0

ac200109.4_fgt002 0

ac200184.3_fgt003 0

ac200238.4_fgt001 0

ac200753.3_fgt003 0

ac200852.4_fgt008 0

ac200874.4_fgt003 0

ac200881.4_fgt004 0

ac201757.3_fgt002 0

ac201769.3_fgt002 0

ac201771.3_fgt005 0

ac201815.4_fgt001 0

ac202035.4_fgt003 0

ac202076.3_fgt011 0

ac202185.4_fgt004 -1.15

ac202439.3_fgt003 0

ac202439.3_fgt005 0

ac202707.3_fgt001 0

ac202915.3_fgt002 0

ac202930.4_fgt003 0

ac203031.3_fgt002 0

ac203160.3_fgt002 0

ac203173.3_fgt004 0

ac203220.3_fgt004 -0.91

ac203278.4_fgt001 0.83

ac203287.3_fgt004 0

ac203369.3_fgt004 0

ac203392.3_fgt004 0

ac203535.4_fgt001 0

ac203535.4_fgt004 0

ac203745.3_fgt005 0

ac203761.3_fgt002 0

ac203811.3_fgt008 0

ac203812.3_fgt004 0

ac203841.3_fgt001 0

ac203841.3_fgt002 0

ac203841.3_fgt009 0

ac203843.4_fgt004 0

ac203862.4_fgt001 0

ac203862.4_fgt002 0

ac203862.4_fgt003 0

ac203862.4_fgt004 0

ac203862.4_fgt005 0

ac203865.3_fgt002 0

ac203957.3_fgt004 0

ac203966.5_fgt004 0

ac203966.5_fgt005 0

ac203971.3_fgt004 0

ac203972.3_fgt001 0

ac203985.4_fgt005 0

ac203989.4_fgt001 0

ac204212.4_fgt001 0

ac204292.4_fgt002 0

ac204352.3_fgt011 0

ac204359.3_fgt005 0

ac204418.3_fgt006 0

ac204502.4_fgt006 0

ac204517.3_fgt002 0

ac204530.4_fgt002 0

ac204530.4_fgt005 0

ac204530.4_fgt006 0

ac204539.4_fgt002 0

ac204604.3_fgt008 0

ac204619.3_fgt003 -1.41

ac204711.3_fgt002 0

ac204842.3_fgt003 0

ac204857.3_fgt001 0

ac204921.4_fgt011 0

ac205024.3_fgt001 -1.16

ac205057.3_fgt002 0

ac205072.3_fgt010 0

ac205122.4_fgt003 0

ac205122.4_fgt004 0

ac205142.3_fgt001 0

ac205314.3_fgt010 0

ac205362.4_fgt002 -1.54

ac205376.4_fgt003 0

ac205419.3_fgt001 0

ac205471.4_fgt007 -1.22

ac205502.4_fgt003 0

ac205521.3_fgt003 0

ac205536.3_fgt003 0

ac205568.3_fgt007 0

ac205574.3_fgt006 0

ac205608.4_fgt004 0

ac205677.3_fgt001 0

ac205677.3_fgt002 0

ac205677.3_fgt005 0

ac205703.4_fgt005 0

ac205703.4_fgt007 0

ac205703.4_fgt010 0

ac205725.3_fgt010 0

ac205730.3_fgt002 0

ac205735.3_fgt006 0

ac205820.3_fgt002 0

ac205886.3_fgt001 0

ac206030.4_fgt001 0

ac206031.3_fgt005 0

ac206060.3_fgt001 0

ac206223.3_fgt001 0

ac206223.3_fgt002 0

ac206223.3_fgt010 0

ac206259.3_fgt003 0

ac206283.3_fgt003 0

ac206514.2_fgt001 -1.28

ac206642.4_fgt001 0

ac206761.3_fgt002 0

ac206788.3_fgt015 0

ac206840.3_fgt002 0

ac206951.3_fgt017 -1.05

ac206968.3_fgt003 0

ac207225.3_fgt003 0

ac207265.3_fgt002 0

ac207342.3_fgt007 0

ac207342.3_fgt008 0

ac207347.3_fgt005 0

ac207394.3_fgt004 0

ac207466.3_fgt003 0

ac207559.3_fgt004 0

ac207628.4_fgt006 0

ac207628.4_fgt009 0

ac207628.4_fgt010 0

ac207628.4_fgt011 0

ac207652.3_fgt001 0

ac207652.3_fgt002 0

ac207652.3_fgt003 0

ac207656.3_fgt002 -1.27

ac207722.2_fgt009 -1.63

ac207732.3_fgt009 1.31

ac207755.3_fgt005 -1.41

ac207890.3_fgt002 0

ac208023.3_fgt001 -1.27

ac208110.2_fgt006 0

ac208110.2_fgt007 0

ac208113.3_fgt006 0

ac208125.3_fgt008 0

ac208201.3_fgt001 0

ac208201.3_fgt002 0

ac208201.3_fgt003 0

ac208221.3_fgt002 0

ac208327.4_fgt003 0

ac208327.4_fgt006 0

ac208341.4_fgt005 0

ac208341.4_fgt006 0

ac208346.3_fgt004 0

ac208348.3_fgt005 0

ac208348.3_fgt012 0

ac208358.3_fgt005 0

ac208363.3_fgt004 0

ac208415.3_fgt003 0

ac208440.3_fgt002 0

ac208440.3_fgt003 0

ac208571.4_fgt001 0

ac208803.3_fgt002 0

ac208833.3_fgt005 0

ac208897.3_fgt002 0

ac208897.3_fgt004 0.85

ac208912.2_fgt002 0

ac209050.3_fgt002 0

ac209057.2_fgt004 0

ac209206.3_fgt008 0

ac209206.3_fgt011 0

ac209208.3_fgt001 0

ac209208.3_fgt002 0

ac209208.3_fgt005 0

ac209242.3_fgt002 1.11

ac209242.3_fgt003 0

ac209257.4_fgt007 0

ac209257.4_fgt008 -0.99

ac209259.3_fgt005 0

ac209374.4_fgt001 0

ac209374.4_fgt002 0

ac209374.4_fgt003 0

ac209462.3_fgt003 0

ac209462.3_fgt004 0

ac209664.3_fgt002 0

ac209705.3_fgt003 0

ac209705.3_fgt004 0

ac209755.3_fgt002 0

ac209755.3_fgt003 0

ac209819.3_fgt011 0

ac209819.3_fgt012 0

ac209858.4_fgt002 0

ac209858.4_fgt004 0

ac209877.3_fgt002 0

ac209898.2_fgt004 0

ac209919.3_fgt002 0

ac209974.4_fgt002 0

ac209987.4_fgt002 0

ac209987.4_fgt010 0

ac209992.3_fgt001 0

ac210003.2_fgt004 0

ac210003.2_fgt015 -1.05

ac210013.4_fgt006 0

ac210013.4_fgt008 0

ac210013.4_fgt009 0

ac210013.4_fgt011 0

ac210013.4_fgt013 0

ac210013.4_fgt014 0

ac210013.4_fgt016 0

ac210013.4_fgt018 0

ac210013.4_fgt019 0

ac210050.3_fgt007 0

ac210169.3_fgt002 0

ac210173.4_fgt005 0

ac210191.2_fgt011 0

ac210193.4_fgt002 -1.01

ac210204.3_fgt002 0

ac210517.3_fgt003 0

ac210528.3_fgt003 0

ac210529.3_fgt004 0

ac210669.3_fgt001 -2.03

ac210691.2_fgt003 0

ac210780.3_fgt006 0

ac210993.3_fgt005 0

ac211164.5_fgt003 0

ac211175.3_fgt003 0

ac211175.3_fgt005 0

ac211276.4_fgt008 0

ac211390.3_fgt001 0

ac211394.4_fgt004 0

ac211401.4_fgt003 0

ac211401.4_fgt004 0

ac211474.3_fgt006 0

ac211689.3_fgt003 0

ac211737.3_fgt012 0

ac211762.4_fgt005 0

ac211892.4_fgt002 0

ac211955.4_fgt013 0

ac212023.4_fgt004 0

ac212068.4_fgt005 0

ac212112.4_fgt002 0

ac212190.4_fgt010 0

ac212190.4_fgt011 1.18

ac212219.3_fgt005 0

ac212353.4_fgt004 0

ac212390.3_fgt005 0

ac212414.3_fgt001 0

ac212449.4_fgt005 0

ac212565.3_fgt001 -1.3

ac212570.3_fgt006 0

ac212579.3_fgt002 0

ac212582.3_fgt006 0

ac212668.2_fgt010 0

ac212684.3_fgt012 0

ac212827.3_fgt003 -1.04

ac212835.3_fgt002 0

ac212835.3_fgt003 0

ac212835.3_fgt004 0

ac212835.3_fgt007 0

ac212835.3_fgt011 0

ac212859.3_fgt007 0

ac212859.3_fgt008 0

ac212873.3_fgt002 0

ac212873.3_fgt008 0

ac213050.3_fgt001 0

ac213099.3_fgt001 0

ac213099.3_fgt002 0

ac213373.3_fgt001 0

ac213463.3_fgt002 0

ac213463.3_fgt003 -0.84

ac213463.3_fgt007 0

ac213464.3_fgt012 -1.02

ac213521.3_fgt003 0

ac213521.3_fgt004 0

ac213521.3_fgt005 0

ac213600.3_fgt002 0

ac213612.3_fgt001 0

ac213612.3_fgt002 0

ac213621.5_fgt004 -2.09

ac213621.5_fgt005 0

ac213654.3_fgt001 0

ac213654.3_fgt002 0

ac213884.3_fgt001 0

ac214244.4_fgt002 0

ac214350.3_fgt007 0

ac214360.3_fgt006 0

ac214360.3_fgt007 0

ac214437.4_fgt004 0

ac214446.3_fgt002 0

ac214448.3_fgt007 0

ac214479.2_fgt001 0

ac214507.3_fgt001 0

ac214602.3_fgt012 0

ac214635.3_fgt002 0

ac214640.3_fgt004 0

ac214648.3_fgt003 0

ac214817.3_fgt004 1

ac215198.3_fgt002 0

ac215201.3_fgt005 0

ac215256.3_fgt002 0

ac215260.3_fgt001 0

ac215260.3_fgt003 0

ac215260.3_fgt004 0

ac215264.2_fgt005 -0.83

ac215309.3_fgt004 1.4

ac215690.3_fgt002 0

ac215914.3_fgt004 0

ac216010.3_fgt001 0

ac216067.3_fgt002 0

ac216871.3_fgt003 -1.71

ac216871.3_fgt004 0

ac217050.4_fgt001 0

ac217050.4_fgt004 0

ac217050.4_fgt006 0

ac217050.4_fgt007 0

ac217051.3_fgt002 0

ac217121.3_fgt006 0

ac217121.3_fgt007 0

ac217123.2_fgt004 0

ac217271.3_fgt002 0

ac217274.3_fgt006 0

ac217293.3_fgt007 0

ac217300.3_fgt005 0

ac217358.3_fgt003 0

ac217358.3_fgt004 0

ac217358.3_fgt005 0

ac217358.3_fgt010 0

ac217358.3_fgt011 0

ac217358.3_fgt012 0

ac217401.3_fgt001 0

ac217401.3_fgt002 0

ac217401.3_fgt003 0

ac217499.3_fgt001 0.82

ac217499.3_fgt003 0

ac217556.3_fgt003 0

ac217665.3_fgt005 0

ac217679.3_fgt004 0

ac217792.3_fgt005 0

ac217811.3_fgt003 0

ac217842.3_fgt001 0

ac217887.3_fgt004 0

ac217887.3_fgt005 0

ac217908.2_fgt003 0

ac217910.3_fgt001 0

ac217950.3_fgt001 0

ac217962.3_fgt005 0

ac217965.2_fgt012 -1.03

ac217975.3_fgt001 0

ac217977.3_fgt003 0

ac217977.3_fgt004 0

ac218148.2_fgt008 0

ac218457.2_fgt013 0

ac218972.3_fgt001 0

ac218972.3_fgt004 0

ac218998.2_fgt004 0

ac218998.2_fgt007 0

ac219061.2_fgt007 0

ac220927.3_fgt004 0

ac220927.3_fgt007 -1.66

ac220927.3_fgt011 0

ac220970.4_fgt002 0

ac220999.3_fgt006 0

ac225147.4_fgt002 0

ac225147.4_fgt003 0

ac225147.4_fgt004 0

ac225185.3_fgt004 0

ac225191.2_fgt009 0

ac225193.3_fgt003 0

ac225193.3_fgt004 0

ac225718.2_fgt006 -1.06

ac226227.2_fgt005 0

ac226227.2_fgt006 0

ac226230.2_fgt002 0

ac226235.2_fgt001 -1.35

ac226235.2_fgt002 0

ac226235.2_fgt003 0

ac226248.1_fgt002 0

ac226373.2_fgt010 0

ac229673.2_fgt006 0

ac230011.2_fgt002 0

ac230013.2_fgt002 0

ac230013.2_fgt003 0

ac230013.2_fgt007 0

ac231180.2_fgt001 0

ac231180.2_fgt006 0

ac231485.1_fgt008 0

ac231745.1_fgt003 -1.02

ac231745.1_fgt004 0

ac231747.1_fgt001 0

ac232238.2_fgt006 -1.57

ac232393.1_fgt009 0

ac233751.1_fgt001 0

ac233788.2_fgt006 0

ac233788.2_fgt007 -1.74

ac233850.1_fgt006 0

ac233851.1_fgt013 0

ac233851.1_fgt014 0

ac233853.1_fgt003 0

ac233856.1_fgt003 0

ac233859.1_fgt002 0

ac233859.1_fgt003 0

ac233859.1_fgt004 0

ac233859.1_fgt005 0

ac233865.1_fgt001 -1.63

ac233865.1_fgt006 0

ac233866.1_fgt006 0

ac233869.1_fgt002 0

ac233869.1_fgt003 0

ac233869.1_fgt004 0

ac233870.1_fgt003 0

ac233870.1_fgt004 0

ac233870.1_fgt005 0

ac233872.1_fgt001 0

ac233872.1_fgt003 0

ac233878.1_fgt004 0

ac233880.1_fgt006 0

ac233880.1_fgt008 1.12

ac233882.1_fgt003 0

ac233882.1_fgt004 0

ac233882.1_fgt005 0

ac233883.1_fgt003 0

ac233885.1_fgt001 0

ac233888.1_fgt002 0

ac233892.1_fgt001 0

ac233893.1_fgt001 0

ac233893.1_fgt003 -1.07

ac233893.1_fgt006 -0.87

ac233894.1_fgt005 0

ac233895.1_fgt001 0

ac233898.1_fgt004 0

ac233901.1_fgt001 0

ac233901.1_fgt005 0

ac233901.1_fgt010 0

ac233910.1_fgt001 0

ac233910.1_fgt006 0

ac233910.1_fgt007 0

ac233910.1_fgt010 0

ac233914.1_fgt001 -0.81

ac233914.1_fgt002 0

ac233920.1_fgt002 0

ac233922.1_fgt005 0

ac233922.1_fgt008 0

ac233926.1_fgt003 1.85

ac233935.1_fgt005 0

ac233939.1_fgt002 0

ac233939.1_fgt003 0

ac233942.1_fgt001 0

ac233942.1_fgt004 0

ac233945.1_fgt001 0

ac233948.1_fgt007 0

ac233948.1_fgt008 0

ac233949.1_fgt004 0

ac233952.1_fgt005 0

ac233953.1_fgt005 0

ac233955.1_fgt010 0

ac233955.1_fgt011 0

ac233958.1_fgt002 0

ac233959.1_fgt002 0

ac233961.1_fgt001 0

ac233966.1_fgt007 0

ac233967.1_fgt001 0

ac233979.1_fgt007 0

ac233979.1_fgt009 0

ac234091.1_fgt001 -1.05

ac234108.1_fgt003 0

ac234150.1_fgt007 0

ac234152.1_fgt001 0

ac234152.1_fgt002 -1.06

ac234154.1_fgt002 0

ac234154.1_fgt007 0

ac234154.1_fgt008 0

ac234154.1_fgt012 0

ac234155.1_fgt002 0

ac234156.1_fgt005 0

ac234156.1_fgt006 0

ac234157.1_fgt002 0

ac234157.1_fgt004 0

ac234158.1_fgt005 0

ac234159.1_fgt002 0

ac234159.1_fgt003 0

ac234160.1_fgt003 -1.63

ac234160.1_fgt006 0

ac234160.1_fgt009 0

ac234161.1_fgt010 0

ac234163.1_fgt004 -1.37

ac234164.1_fgt002 0

ac234165.1_fgt001 0

ac234165.1_fgt002 0

ac234169.1_fgt003 0

ac234171.1_fgt003 0

ac234171.1_fgt005 0

ac234175.1_fgt009 0

ac234180.1_fgt001 0

ac234180.1_fgt002 0

ac234185.1_fgt004 1.87

ac234185.1_fgt007 0

ac234192.1_fgt001 0

ac234196.1_fgt002 0

ac234196.1_fgt004 0

ac234197.1_fgt001 0

ac234201.1_fgt001 0

ac234201.1_fgt003 0

ac234203.1_fgt002 0

ac234203.1_fgt004 0

ac234203.1_fgt010 0

ac234203.1_fgt011 0

ac234203.1_fgt012 2.34

ac234203.1_fgt013 0

ac234203.1_fgt014 0

ac234204.1_fgt006 0

ac234515.1_fgt003 0

ac234519.1_fgt006 0

ac234520.1_fgt003 -1.93

ac234520.1_fgt004 0

ac234520.1_fgt005 0

ac234521.1_fgt005 0

ac234522.1_fgt005 0

ac234526.1_fgt005 0

ac234528.1_fgt001 0

ac234528.1_fgt004 0

ac234528.1_fgt005 0

ac234575.1_fgt006 0

ac235534.1_fgt002 0

ac235534.1_fgt008 0

ac235535.1_fgt001 0

ac235537.1_fgt001 0

ac235540.1_fgt002 0

ac235541.1_fgt002 0

ac235541.1_fgt003 0

ac235544.1_fgt008 0

ac235546.1_fgt001 0

ac235547.1_fgt002 0

ac235547.1_fgt005 1

af466202.2_fgt005 0

af466202.2_fgt008 0

af466202.2_fgt009 0

af466202.2_fgt011 0

af546187.1_fgt011 -0.9

ay542798.1_fgt003 0

ef517601.1_fgt010 0

ef517601.1_fgt012 0

ef517601.1_fgt015 0

ef517601.1_fgt021 0

ef517601.1_fgt025 0

grmzm2g000039_t01 0

grmzm2g000042_t01 0

grmzm2g000052_t03 0

grmzm2g000114_t03 0

grmzm2g000166_t01 0

grmzm2g000171_t02 0

grmzm2g000209_t02 0

grmzm2g000219_t01 0

grmzm2g000221_t01 0

grmzm2g000231_t01 0

grmzm2g000245_t02 0

grmzm2g000256_t01 0

grmzm2g000261_t01 0

grmzm2g000268_t02 0

grmzm2g000278_t02 0

grmzm2g000326_t01 0

grmzm2g000339_t01 0

grmzm2g000353_t01 0

grmzm2g000361_t02 0

grmzm2g000371_t03 0

grmzm2g000393_t01 0

grmzm2g000397_t01 0

grmzm2g000423_t03 0

grmzm2g000451_t01 0

grmzm2g000453_t01 0

grmzm2g000489_t01 0

grmzm2g000531_t03 0

grmzm2g000560_t01 0

grmzm2g000581_t01 0

grmzm2g000587_t01 0

grmzm2g000593_t04 0

grmzm2g000596_t01 0

grmzm2g000610_t01 0

grmzm2g000614_t01 0

grmzm2g000615_t01 0

grmzm2g000620_t02 0

grmzm2g000622_t01 0

grmzm2g000623_t01 0

grmzm2g000632_t01 0

grmzm2g000644_t01 0

grmzm2g000645_t01 0

grmzm2g000650_t01 0

grmzm2g000660_t01 0

grmzm2g000674_t01 0

grmzm2g000686_t01 0

grmzm2g000710_t01 0

grmzm2g000713_t03 0

grmzm2g000718_t01 0

grmzm2g000719_t01 0

grmzm2g000739_t02 0

grmzm2g000741_t01 0

grmzm2g000749_t02 0

grmzm2g000753_t02 0

grmzm2g000764_t01 0

grmzm2g000777_t01 0

grmzm2g000801_t01 0

grmzm2g000812_t03 0

grmzm2g000816_t04 0

grmzm2g000818_t02 -0.84

grmzm2g000823_t01 0

grmzm2g000874_t01 0

grmzm2g000909_t01 0

grmzm2g000923_t01 0

grmzm2g000936_t01 0

grmzm2g000937_t03 0

grmzm2g000973_t02 0

grmzm2g000980_t01 0

grmzm2g001024_t02 0

grmzm2g001048_t02 0

grmzm2g001079_t01 -1.01

grmzm2g001084_t02 0

grmzm2g001088_t02 0

grmzm2g001114_t02 0

grmzm2g001180_t04 0

grmzm2g001184_t02 0

grmzm2g001191_t01 0

grmzm2g001200_t01 0

grmzm2g001243_t02 0

grmzm2g001255_t01 0

grmzm2g001272_t01 0

grmzm2g001279_t01 0

grmzm2g001288_t02 0

grmzm2g001289_t03 0

grmzm2g001296_t02 0

grmzm2g001297_t01 0

grmzm2g001304_t01 0

grmzm2g001314_t01 1.04

grmzm2g001327_t01 0

grmzm2g001334_t06 0

grmzm2g001363_t01 0

grmzm2g001415_t02 0

grmzm2g001421_t02 0

grmzm2g001426_t01 0

grmzm2g001444_t02 0

grmzm2g001454_t01 0

grmzm2g001457_t01 0

grmzm2g001462_t01 0

grmzm2g001500_t01 0

grmzm2g001508_t01 0

grmzm2g001514_t01 0

grmzm2g001530_t01 0

grmzm2g001541_t01 0

grmzm2g001551_t01 0

grmzm2g001577_t02 0

grmzm2g001631_t01 0

grmzm2g001639_t02 0

grmzm2g001645_t02 0

grmzm2g001652_t01 0

grmzm2g001653_t01 0

grmzm2g001660_t01 0

grmzm2g001661_t01 0

grmzm2g001663_t01 0

grmzm2g001668_t01 0

grmzm2g001676_t01 0

grmzm2g001696_t02 0

grmzm2g001724_t03 0

grmzm2g001748_t04 0

grmzm2g001750_t03 0

grmzm2g001755_t01 0

grmzm2g001777_t03 0

grmzm2g001803_t02 0

grmzm2g001805_t01 0

grmzm2g001816_t01 0

grmzm2g001832_t01 0

grmzm2g001869_t01 0

grmzm2g001887_t01 0

grmzm2g001895_t01 0

grmzm2g001898_t01 0

grmzm2g001904_t01 0

grmzm2g001905_t03 0

grmzm2g001918_t02 0

grmzm2g001930_t01 0

grmzm2g001934_t02 0

grmzm2g001956_t01 0

grmzm2g001973_t03 0

grmzm2g001991_t01 0

grmzm2g002002_t03 0

grmzm2g002023_t02 0

grmzm2g002075_t01 0

grmzm2g002100_t02 0

grmzm2g002115_t02 1.21

grmzm2g002128_t02 0

grmzm2g002130_t02 0

grmzm2g002135_t01 0

grmzm2g002138_t01 0

grmzm2g002142_t01 0

grmzm2g002165_t03 0

grmzm2g002173_t01 0

grmzm2g002178_t01 0

grmzm2g002220_t01 0

grmzm2g002260_t01 -0.84

grmzm2g002276_t01 0

grmzm2g002286_t01 0

grmzm2g002297_t04 0

grmzm2g002304_t01 0

grmzm2g002347_t01 0

grmzm2g002361_t01 0

grmzm2g002391_t02 0

grmzm2g002416_t02 0

grmzm2g002427_t01 0

grmzm2g002440_t03 0

grmzm2g002492_t04 0

grmzm2g002499_t01 0

grmzm2g002520_t01 0

grmzm2g002523_t01 0

grmzm2g002531_t01 0

grmzm2g002558_t02 0

grmzm2g002559_t01 0

grmzm2g002578_t01 0

grmzm2g002603_t01 0

grmzm2g002606_t01 0

grmzm2g002616_t01 0

grmzm2g002617_t02 0

grmzm2g002642_t01 0

grmzm2g002646_t01 0

grmzm2g002652_t01 0

grmzm2g002656_t01 0

grmzm2g002699_t02 0

grmzm2g002754_t01 0

grmzm2g002756_t02 0

grmzm2g002765_t02 0

grmzm2g002786_t01 0

grmzm2g002807_t01 0

grmzm2g002825_t01 0

grmzm2g002828_t02 0

grmzm2g002851_t02 0

grmzm2g002859_t01 0

grmzm2g002874_t01 0

grmzm2g002879_t03 0

grmzm2g002894_t01 0

grmzm2g002959_t01 0

grmzm2g002978_t01 0

grmzm2g002993_t01 0

grmzm2g002999_t01 0

grmzm2g003002_t03 -1.32

grmzm2g003022_t01 0

grmzm2g003023_t01 0

grmzm2g003028_t02 0

grmzm2g003033_t01 0

grmzm2g003038_t03 0

grmzm2g003043_t02 0

grmzm2g003057_t03 0

grmzm2g003064_t01 0

grmzm2g003068_t01 0

grmzm2g003076_t02 0

grmzm2g003090_t03 0

grmzm2g003096_t04 0

grmzm2g003108_t01 0

grmzm2g003124_t04 0

grmzm2g003130_t04 0

grmzm2g003138_t01 -1.6

grmzm2g003157_t01 0

grmzm2g003165_t02 0

grmzm2g003172_t01 0

grmzm2g003179_t01 -0.88

grmzm2g003246_t01 0

grmzm2g003252_t02 0

grmzm2g003289_t01 0

grmzm2g003304_t01 0

grmzm2g003306_t01 -0.94

grmzm2g003307_t01 0

grmzm2g003318_t01 0

grmzm2g003331_t01 0.85

grmzm2g003354_t01 0

grmzm2g003377_t01 0

grmzm2g003384_t02 0

grmzm2g003385_t01 0

grmzm2g003406_t01 0

grmzm2g003411_t01 1.47

grmzm2g003417_t01 0

grmzm2g003424_t02 0

grmzm2g003426_t01 0

grmzm2g003452_t01 0

grmzm2g003461_t01 0

grmzm2g003488_t01 0

grmzm2g003501_t01 2.17

grmzm2g003506_t02 0

grmzm2g003509_t03 0

grmzm2g003518_t01 0

grmzm2g003558_t01 0

grmzm2g003563_t03 0

grmzm2g003565_t01 0

grmzm2g003595_t02 0

grmzm2g003602_t01 0

grmzm2g003635_t01 0

grmzm2g003638_t01 0

grmzm2g003640_t06 0

grmzm2g003642_t01 0

grmzm2g003656_t07 0

grmzm2g003662_t01 0

grmzm2g003663_t01 0

grmzm2g003682_t01 0

grmzm2g003715_t03 0.89

grmzm2g003720_t02 0

grmzm2g003724_t01 0

grmzm2g003725_t01 0

grmzm2g003732_t01 0

grmzm2g003734_t01 0

grmzm2g003742_t01 0

grmzm2g003750_t01 0

grmzm2g003754_t02 0

grmzm2g003762_t03 0

grmzm2g003765_t04 0

grmzm2g003769_t01 0

grmzm2g003794_t05 0

grmzm2g003814_t01 0

grmzm2g003852_t01 0

grmzm2g003853_t01 0

grmzm2g003861_t01 0

grmzm2g003869_t01 0

grmzm2g003875_t01 0

grmzm2g003883_t01 0

grmzm2g003897_t01 0

grmzm2g003930_t07 0

grmzm2g003937_t02 0

grmzm2g003963_t01 0

grmzm2g003970_t01 0

grmzm2g003988_t01 0

grmzm2g003992_t01 0

grmzm2g004006_t02 0

grmzm2g004023_t06 0

grmzm2g004040_t01 0

grmzm2g004057_t02 0

grmzm2g004060_t01 0

grmzm2g004083_t01 0

grmzm2g004092_t01 0

grmzm2g004111_t01 0

grmzm2g004128_t01 0

grmzm2g004131_t01 0

grmzm2g004138_t04 0

grmzm2g004140_t07 0

grmzm2g004161_t03 -1.36

grmzm2g004172_t01 0

grmzm2g004182_t02 0

grmzm2g004183_t01 0

grmzm2g004188_t01 0

grmzm2g004211_t01 0

grmzm2g004222_t03 0

grmzm2g004259_t01 0

grmzm2g004278_t01 0

grmzm2g004290_t01 0

grmzm2g004301_t01 0

grmzm2g004305_t02 1.28

grmzm2g004320_t01 0

grmzm2g004349_t03 0

grmzm2g004352_t01 0

grmzm2g004356_t01 0

grmzm2g004365_t01 0

grmzm2g004377_t01 0

grmzm2g004382_t01 0

grmzm2g004396_t01 0

grmzm2g004397_t01 0

grmzm2g004412_t01 0

grmzm2g004414_t03 0

grmzm2g004422_t02 0

grmzm2g004435_t02 0

grmzm2g004445_t01 0

grmzm2g004455_t02 0

grmzm2g004459_t02 0

grmzm2g004466_t01 0

grmzm2g004468_t04 0

grmzm2g004475_t01 0

grmzm2g004480_t01 0

grmzm2g004483_t01 0

grmzm2g004511_t01 0

grmzm2g004516_t01 0

grmzm2g004528_t03 0

grmzm2g004531_t01 1.21

grmzm2g004534_t01 0

grmzm2g004548_t01 0

grmzm2g004572_t01 0

grmzm2g004583_t01 0

grmzm2g004590_t01 0

grmzm2g004592_t02 0

grmzm2g004619_t02 0

grmzm2g004641_t07 0

grmzm2g004683_t01 0

grmzm2g004694_t01 0

grmzm2g004696_t01 0

grmzm2g004699_t04 -0.89

grmzm2g004703_t02 0

grmzm2g004709_t02 0

grmzm2g004715_t01 0

grmzm2g004732_t01 0

grmzm2g004736_t01 0

grmzm2g004741_t03 0

grmzm2g004748_t01 -1.51

grmzm2g004749_t01 0

grmzm2g004768_t01 0

grmzm2g004784_t02 0

grmzm2g004795_t01 0

grmzm2g004835_t02 0

grmzm2g004847_t01 0

grmzm2g004878_t01 0

grmzm2g004880_t01 0

grmzm2g004888_t01 0

grmzm2g004898_t01 0

grmzm2g004908_t01 0

grmzm2g004932_t01 0

grmzm2g004949_t01 0

grmzm2g004955_t01 0

grmzm2g004957_t01 0

grmzm2g004959_t04 0

grmzm2g004990_t03 0

grmzm2g004996_t02 0

grmzm2g005000_t02 0

grmzm2g005024_t02 0

grmzm2g005036_t03 0

grmzm2g005040_t01 0

grmzm2g005080_t01 0

grmzm2g005107_t02 0

grmzm2g005126_t03 0

grmzm2g005146_t01 0

grmzm2g005163_t01 0

grmzm2g005195_t03 0

grmzm2g005199_t02 0

grmzm2g005207_t01 0

grmzm2g005209_t01 0

grmzm2g005233_t01 0

grmzm2g005236_t01 0.85

grmzm2g005251_t01 0

grmzm2g005256_t01 0

grmzm2g005260_t01 0

grmzm2g005265_t03 0

grmzm2g005298_t01 1.02

grmzm2g005308_t01 0

grmzm2g005314_t01 0

grmzm2g005339_t01 0

grmzm2g005346_t03 0

grmzm2g005347_t01 0

grmzm2g005350_t01 0

grmzm2g005365_t06 0

grmzm2g005374_t01 0

grmzm2g005419_t01 0

grmzm2g005433_t02 0

grmzm2g005435_t01 0

grmzm2g005444_t01 0

grmzm2g005452_t02 0

grmzm2g005459_t01 0

grmzm2g005483_t01 0

grmzm2g005486_t01 0

grmzm2g005493_t01 0

grmzm2g005499_t01 0

grmzm2g005552_t01 0

grmzm2g005562_t04 0

grmzm2g005583_t01 0

grmzm2g005592_t01 0

grmzm2g005622_t03 0

grmzm2g005624_t01 0

grmzm2g005646_t01 0

grmzm2g005652_t01 0

grmzm2g005710_t01 0

grmzm2g005732_t03 0

grmzm2g005737_t02 0

grmzm2g005743_t01 0

grmzm2g005749_t01 0

grmzm2g005771_t02 0

grmzm2g005774_t01 0

grmzm2g005788_t01 0

grmzm2g005791_t02 0.89

grmzm2g005834_t01 0

grmzm2g005840_t01 0

grmzm2g005844_t04 0

grmzm2g005848_t01 0

grmzm2g005849_t02 0

grmzm2g005859_t02 0

grmzm2g005869_t01 0

grmzm2g005886_t02 0

grmzm2g005887_t09 0

grmzm2g005909_t01 0

grmzm2g005938_t01 0

grmzm2g005939_t02 -2.57

grmzm2g005947_t01 0

grmzm2g005954_t01 0

grmzm2g005973_t02 0

grmzm2g005980_t01 0

grmzm2g005984_t01 0

grmzm2g005990_t02 0

grmzm2g005991_t01 0

grmzm2g006006_t01 0

grmzm2g006042_t03 0

grmzm2g006047_t01 0

grmzm2g006069_t02 0

grmzm2g006071_t01 0

grmzm2g006080_t01 0

grmzm2g006083_t01 0

grmzm2g006085_t01 0

grmzm2g006107_t01 0

grmzm2g006117_t03 0

grmzm2g006121_t02 0

grmzm2g006130_t08 0

grmzm2g006144_t01 0

grmzm2g006178_t02 0

grmzm2g006216_t01 0

grmzm2g006229_t01 0

grmzm2g006246_t01 0

grmzm2g006287_t01 -0.96

grmzm2g006293_t01 0

grmzm2g006297_t03 0

grmzm2g006329_t01 0

grmzm2g006341_t01 0

grmzm2g006363_t02 0

grmzm2g006370_t02 0

grmzm2g006377_t01 0

grmzm2g006416_t06 0

grmzm2g006428_t02 0

grmzm2g006429_t01 0

grmzm2g006450_t01 0

grmzm2g006453_t06 0

grmzm2g006468_t01 0

grmzm2g006474_t01 0

grmzm2g006477_t01 0

grmzm2g006480_t01 0

grmzm2g006493_t05 0

grmzm2g006505_t01 0

grmzm2g006507_t05 0

grmzm2g006565_t01 0

grmzm2g006631_t01 -1.23

grmzm2g006661_t01 0

grmzm2g006673_t01 0

grmzm2g006676_t01 0

grmzm2g006678_t01 0

grmzm2g006704_t01 0

grmzm2g006707_t02 0

grmzm2g006736_t02 0

grmzm2g006745_t03 0

grmzm2g006752_t02 0

grmzm2g006763_t01 0

grmzm2g006765_t04 0

grmzm2g006780_t01 0

grmzm2g006781_t01 1.06

grmzm2g006790_t01 0

grmzm2g006791_t03 0

grmzm2g006806_t01 0

grmzm2g006894_t01 0

grmzm2g006937_t01 1.07

grmzm2g006942_t02 0

grmzm2g006948_t01 0.9

grmzm2g006953_t01 0

grmzm2g006958_t01 0

grmzm2g006964_t01 0

grmzm2g006977_t01 0

grmzm2g007012_t01 0

grmzm2g007025_t01 0

grmzm2g007038_t01 0

grmzm2g007055_t04 0

grmzm2g007060_t01 0

grmzm2g007063_t03 0

grmzm2g007080_t01 0

grmzm2g007103_t01 0

grmzm2g007120_t04 0

grmzm2g007122_t01 0

grmzm2g007140_t01 0

grmzm2g007146_t01 0

grmzm2g007151_t03 0

grmzm2g007157_t01 0

grmzm2g007160_t01 0

grmzm2g007188_t01 0

grmzm2g007195_t01 0

grmzm2g007201_t02 0.85

grmzm2g007206_t02 0

grmzm2g007229_t01 0

grmzm2g007248_t01 0

grmzm2g007249_t01 0

grmzm2g007256_t01 0

grmzm2g007260_t05 0

grmzm2g007263_t02 0

grmzm2g007277_t01 0

grmzm2g007283_t02 0

grmzm2g007288_t02 0

grmzm2g007300_t03 0

grmzm2g007324_t01 -1.79

grmzm2g007339_t01 0

grmzm2g007347_t01 0

grmzm2g007372_t01 0

grmzm2g007381_t01 0

grmzm2g007384_t01 0

grmzm2g007385_t01 0

grmzm2g007399_t01 0

grmzm2g007404_t01 0

grmzm2g007441_t01 0

grmzm2g007453_t03 0

grmzm2g007466_t02 0

grmzm2g007475_t01 0

grmzm2g007477_t01 0

grmzm2g007486_t01 0

grmzm2g007489_t01 0

grmzm2g007514_t01 0

grmzm2g007555_t01 0

grmzm2g007587_t01 0

grmzm2g007590_t02 0

grmzm2g007630_t01 0

grmzm2g007647_t01 0.9

grmzm2g007651_t01 0

grmzm2g007666_t02 -0.81

grmzm2g007675_t01 0

grmzm2g007683_t03 0

grmzm2g007695_t01 0

grmzm2g007721_t01 0

grmzm2g007734_t01 0

grmzm2g007791_t02 0

grmzm2g007801_t01 0

grmzm2g007810_t01 0

grmzm2g007835_t02 0

grmzm2g007848_t02 0

grmzm2g007854_t02 0

grmzm2g007867_t01 0

grmzm2g007871_t01 0

grmzm2g007885_t03 0

grmzm2g007907_t02 0

grmzm2g007914_t01 0

grmzm2g007928_t01 0

grmzm2g007933_t02 1.15

grmzm2g007939_t04 0

grmzm2g007957_t01 0

grmzm2g008058_t04 0

grmzm2g008060_t01 0

grmzm2g008061_t02 0

grmzm2g008072_t01 0

grmzm2g008095_t02 0

grmzm2g008106_t01 -1.02

grmzm2g008108_t01 0

grmzm2g008123_t01 0

grmzm2g008175_t01 0

grmzm2g008209_t03 0

grmzm2g008216_t03 0

grmzm2g008226_t01 0

grmzm2g008232_t01 0

grmzm2g008242_t01 0

grmzm2g008247_t02 0

grmzm2g008250_t01 -1.35

grmzm2g008259_t02 0

grmzm2g008263_t02 0

grmzm2g008273_t01 0

grmzm2g008290_t01 -1.32

grmzm2g008309_t01 0

grmzm2g008327_t01 0

grmzm2g008353_t01 -1.36

grmzm2g008356_t01 0

grmzm2g008410_t01 0

grmzm2g008425_t02 0

grmzm2g008456_t01 0

grmzm2g008464_t02 0

grmzm2g008490_t01 0

grmzm2g008497_t01 0

grmzm2g008501_t03 0

grmzm2g008507_t01 0

grmzm2g008513_t01 0

grmzm2g008528_t01 0

grmzm2g008558_t02 0

grmzm2g008607_t01 0

grmzm2g008622_t01 0

grmzm2g008623_t01 0

grmzm2g008643_t02 0

grmzm2g008647_t01 0

grmzm2g008649_t01 0

grmzm2g008687_t01 0

grmzm2g008710_t01 0.96

grmzm2g008714_t01 0

grmzm2g008728_t03 0

grmzm2g008731_t01 0

grmzm2g008748_t01 0

grmzm2g008751_t01 0

grmzm2g008765_t02 0

grmzm2g008859_t01 0.84

grmzm2g008862_t02 0.95

grmzm2g008892_t01 0

grmzm2g008919_t01 0

grmzm2g009021_t02 0

grmzm2g009048_t01 0

grmzm2g009070_t01 0

grmzm2g009091_t04 0.92

grmzm2g009103_t01 0

grmzm2g009136_t01 0

grmzm2g009144_t01 0

grmzm2g009163_t03 0

grmzm2g009184_t01 0

grmzm2g009188_t02 0

grmzm2g009196_t01 0

grmzm2g009208_t01 0

grmzm2g009223_t01 0

grmzm2g009232_t04 0

grmzm2g009253_t01 0

grmzm2g009265_t01 0

grmzm2g009282_t01 0

grmzm2g009289_t01 0

grmzm2g009320_t01 0

grmzm2g009323_t01 0

grmzm2g009326_t02 0

grmzm2g009335_t02 0

grmzm2g009344_t01 0

grmzm2g009353_t01 0

grmzm2g009365_t01 0

grmzm2g009368_t02 0

grmzm2g009387_t01 0

grmzm2g009406_t01 0

grmzm2g009412_t04 0

grmzm2g009438_t01 0

grmzm2g009443_t01 0

grmzm2g009448_t01 0

grmzm2g009464_t02 0

grmzm2g009465_t01 0

grmzm2g009478_t01 0

grmzm2g009479_t02 -1.4

grmzm2g009538_t01 0

grmzm2g009544_t01 0

grmzm2g009563_t01 0

grmzm2g009571_t03 0

grmzm2g009575_t02 0

grmzm2g009591_t01 0

grmzm2g009593_t01 0

grmzm2g009598_t01 0

grmzm2g009607_t04 1.03

grmzm2g009616_t01 0

grmzm2g009626_t02 0

grmzm2g009627_t01 0

grmzm2g009653_t01 0

grmzm2g009655_t01 0

grmzm2g009661_t01 0

grmzm2g009673_t01 0

grmzm2g009681_t02 0

grmzm2g009683_t01 0

grmzm2g009703_t01 0

grmzm2g009715_t01 0

grmzm2g009735_t01 0

grmzm2g009785_t05 0

grmzm2g009792_t01 0

grmzm2g009795_t01 0

grmzm2g009804_t01 0

grmzm2g009808_t01 0

grmzm2g009843_t02 0

grmzm2g009845_t02 0

grmzm2g009849_t01 0

grmzm2g009851_t01 0

grmzm2g009869_t01 0

grmzm2g009871_t01 0

grmzm2g009876_t01 0.82

grmzm2g009888_t03 0

grmzm2g009892_t01 0

grmzm2g009894_t01 0

grmzm2g009895_t01 0

grmzm2g009901_t01 0

grmzm2g009928_t02 0

grmzm2g009936_t02 0

grmzm2g009940_t03 0

grmzm2g009958_t02 0

grmzm2g009969_t01 0

grmzm2g009994_t01 0

grmzm2g009995_t02 0

grmzm2g010000_t03 -0.96

grmzm2g010011_t01 0

grmzm2g010017_t02 0

grmzm2g010034_t04 0

grmzm2g010037_t01 0

grmzm2g010044_t04 0

grmzm2g010054_t01 0

grmzm2g010056_t02 0

grmzm2g010065_t01 0

grmzm2g010074_t01 0

grmzm2g010075_t01 0

grmzm2g010085_t02 0

grmzm2g010091_t01 0

grmzm2g010093_t04 0

grmzm2g010095_t01 0

grmzm2g010136_t04 0

grmzm2g010142_t02 0

grmzm2g010146_t01 0

grmzm2g010152_t01 0

grmzm2g010176_t01 0

grmzm2g010196_t01 0

grmzm2g010199_t01 0

grmzm2g010202_t02 0

grmzm2g010221_t01 0

grmzm2g010238_t01 0.96

grmzm2g010257_t01 0

grmzm2g010290_t01 0

grmzm2g010298_t01 0

grmzm2g010302_t01 0

grmzm2g010315_t01 0

grmzm2g010321_t02 0

grmzm2g010323_t05 0

grmzm2g010328_t01 0

grmzm2g010342_t01 0

grmzm2g010348_t01 0

grmzm2g010349_t03 0

grmzm2g010353_t01 0

grmzm2g010357_t04 0

grmzm2g010362_t01 0

grmzm2g010363_t01 0

grmzm2g010389_t01 0

grmzm2g010406_t06 0

grmzm2g010422_t01 0

grmzm2g010435_t01 0

grmzm2g010447_t01 0

grmzm2g010452_t01 1.46

grmzm2g010460_t06 0

grmzm2g010468_t01 0

grmzm2g010490_t02 0

grmzm2g010491_t02 0

grmzm2g010551_t03 0

grmzm2g010555_t01 0

grmzm2g010596_t03 0

grmzm2g010599_t01 0

grmzm2g010628_t01 0

grmzm2g010637_t02 0

grmzm2g010649_t01 0

grmzm2g010693_t02 0

grmzm2g010731_t01 0

grmzm2g010743_t01 0

grmzm2g010754_t01 0

grmzm2g010765_t02 0

grmzm2g010775_t01 0

grmzm2g010783_t01 0

grmzm2g010797_t03 0

grmzm2g010801_t01 0

grmzm2g010823_t01 0

grmzm2g010834_t02 0

grmzm2g010836_t01 0

grmzm2g010863_t03 0.92

grmzm2g010868_t01 0

grmzm2g010871_t04 0

grmzm2g010884_t02 0

grmzm2g010909_t01 0

grmzm2g010920_t01 0

grmzm2g010929_t01 0

grmzm2g010933_t01 0

grmzm2g010937_t01 0

grmzm2g010941_t01 0

grmzm2g010944_t03 0

grmzm2g010953_t01 0

grmzm2g010960_t01 0

grmzm2g010973_t01 0

grmzm2g010987_t01 -0.99

grmzm2g010991_t02 0

grmzm2g011006_t01 0

grmzm2g011030_t05 0

grmzm2g011031_t02 0

grmzm2g011068_t01 0

grmzm2g011070_t01 0

grmzm2g011071_t03 -1.19

grmzm2g011078_t02 0

grmzm2g011079_t02 0

grmzm2g011085_t01 0

grmzm2g011129_t01 0

grmzm2g011140_t01 0

grmzm2g011144_t01 0

grmzm2g011169_t01 0

grmzm2g011173_t04 0

grmzm2g011213_t01 0

grmzm2g011219_t01 0

grmzm2g011222_t02 0

grmzm2g011253_t02 0

grmzm2g011269_t01 0

grmzm2g011314_t01 0

grmzm2g011319_t01 0

grmzm2g011347_t01 0

grmzm2g011355_t01 0

grmzm2g011357_t01 0

grmzm2g011364_t02 0

grmzm2g011373_t01 0

grmzm2g011404_t01 0

grmzm2g011434_t01 0

grmzm2g011437_t01 0

grmzm2g011463_t01 0

grmzm2g011469_t01 0

grmzm2g011473_t01 0

grmzm2g011479_t02 0

grmzm2g011483_t02 0

grmzm2g011491_t02 0

grmzm2g011507_t03 0

grmzm2g011513_t01 0

grmzm2g011518_t01 0

grmzm2g011520_t02 0

grmzm2g011523_t01 0

grmzm2g011526_t01 1.07

grmzm2g011541_t01 0

grmzm2g011588_t01 0

grmzm2g011590_t02 0

grmzm2g011592_t01 0

grmzm2g011608_t02 0

grmzm2g011624_t01 0

grmzm2g011627_t04 0

grmzm2g011631_t01 0

grmzm2g011636_t01 0

grmzm2g011655_t01 0

grmzm2g011662_t02 0

grmzm2g011731_t01 0

grmzm2g011742_t01 0

grmzm2g011746_t01 0

grmzm2g011777_t02 0

grmzm2g011800_t01 0

grmzm2g011858_t01 0

grmzm2g011878_t01 0

grmzm2g011888_t01 0

grmzm2g011896_t03 0

grmzm2g011912_t02 0

grmzm2g011915_t01 0

grmzm2g011923_t02 0

grmzm2g011932_t01 0

grmzm2g011980_t01 0

grmzm2g011998_t01 0

grmzm2g012021_t01 0

grmzm2g012030_t02 0

grmzm2g012031_t01 0

grmzm2g012041_t01 0

grmzm2g012044_t01 0

grmzm2g012046_t01 0

grmzm2g012088_t05 0

grmzm2g012102_t01 0

grmzm2g012119_t04 0

grmzm2g012123_t02 0

grmzm2g012126_t01 0

grmzm2g012140_t01 0

grmzm2g012143_t01 0

grmzm2g012148_t01 0

grmzm2g012156_t01 0

grmzm2g012160_t01 1.42

grmzm2g012174_t01 0

grmzm2g012176_t01 0

grmzm2g012178_t01 0

grmzm2g012183_t01 0

grmzm2g012200_t01 0

grmzm2g012209_t04 0

grmzm2g012213_t01 0

grmzm2g012216_t01 0

grmzm2g012229_t02 0

grmzm2g012233_t01 0

grmzm2g012238_t01 0

grmzm2g012260_t01 0

grmzm2g012262_t01 0

grmzm2g012269_t01 0

grmzm2g012276_t02 0

grmzm2g012280_t01 0

grmzm2g012284_t01 0.98

grmzm2g012302_t05 0

grmzm2g012306_t01 -2.39

grmzm2g012319_t02 0

grmzm2g012324_t01 0

grmzm2g012326_t01 0

grmzm2g012340_t01 0

grmzm2g012391_t01 0

grmzm2g012393_t01 0

grmzm2g012397_t01 0

grmzm2g012399_t01 0

grmzm2g012404_t01 0

grmzm2g012413_t01 0

grmzm2g012416_t01 0

grmzm2g012432_t01 0

grmzm2g012434_t01 0

grmzm2g012453_t01 0

grmzm2g012479_t01 0

grmzm2g012501_t01 0

grmzm2g012546_t01 0

grmzm2g012566_t01 0

grmzm2g012584_t01 0

grmzm2g012595_t02 0.81

grmzm2g012601_t01 0

grmzm2g012628_t01 0

grmzm2g012631_t01 0

grmzm2g012654_t02 0

grmzm2g012690_t01 0

grmzm2g012717_t01 -1.52

grmzm2g012724_t03 0

grmzm2g012737_t03 0

grmzm2g012758_t01 0

grmzm2g012761_t01 0.81

grmzm2g012814_t01 0

grmzm2g012841_t10 0

grmzm2g012861_t03 -1.3

grmzm2g012863_t02 0

grmzm2g012870_t05 0

grmzm2g012874_t01 0

grmzm2g012891_t01 0

grmzm2g012923_t01 0

grmzm2g012926_t02 0

grmzm2g012942_t01 0

grmzm2g012958_t02 0

grmzm2g012964_t01 0

grmzm2g012966_t02 0

grmzm2g012992_t01 0

grmzm2g012999_t01 0

grmzm2g013002_t02 0

grmzm2g013065_t01 0

grmzm2g013082_t01 -0.89

grmzm2g013100_t01 0

grmzm2g013114_t01 0

grmzm2g013152_t01 0

grmzm2g013158_t01 0

grmzm2g013166_t01 0

grmzm2g013176_t01 0

grmzm2g013201_t01 0

grmzm2g013206_t01 0

grmzm2g013236_t03 0

grmzm2g013255_t01 -0.88

grmzm2g013283_t01 0

grmzm2g013318_t01 0

grmzm2g013324_t02 0

grmzm2g013342_t01 0

grmzm2g013349_t01 0

grmzm2g013357_t02 0

grmzm2g013378_t01 0

grmzm2g013391_t01 0

grmzm2g013398_t01 0

grmzm2g013433_t02 0

grmzm2g013461_t03 0

grmzm2g013471_t05 0

grmzm2g013478_t01 0

grmzm2g013546_t01 0

grmzm2g013592_t01 0

grmzm2g013600_t02 0

grmzm2g013607_t03 0

grmzm2g013617_t02 0

grmzm2g013619_t01 0

grmzm2g013625_t02 0

grmzm2g013627_t01 0

grmzm2g013634_t01 0

grmzm2g013639_t02 0

grmzm2g013650_t02 0

grmzm2g013677_t03 0

grmzm2g013695_t01 0

grmzm2g013704_t01 0

grmzm2g013728_t01 0

grmzm2g013750_t01 0

grmzm2g013765_t01 0

grmzm2g013783_t02 0

grmzm2g013794_t01 0

grmzm2g013811_t01 0

grmzm2g013821_t01 0

grmzm2g013890_t01 0

grmzm2g013892_t01 0

grmzm2g013900_t01 0

grmzm2g013908_t03 0

grmzm2g013936_t01 0

grmzm2g013944_t03 0

grmzm2g013948_t02 0

grmzm2g013957_t02 0

grmzm2g013964_t01 0

grmzm2g013970_t02 0

grmzm2g013981_t02 0

grmzm2g013986_t01 -1.16

grmzm2g014004_t01 0

grmzm2g014055_t02 0

grmzm2g014066_t01 0

grmzm2g014069_t01 0

grmzm2g014071_t01 0

grmzm2g014076_t01 0

grmzm2g014089_t02 0

grmzm2g014091_t01 0

grmzm2g014099_t01 0

grmzm2g014106_t01 0

grmzm2g014116_t01 0

grmzm2g014136_t01 0

grmzm2g014154_t02 0

grmzm2g014170_t02 0

grmzm2g014180_t01 0

grmzm2g014193_t03 0

grmzm2g014240_t02 0

grmzm2g014276_t02 0

grmzm2g014295_t01 0

grmzm2g014300_t01 0

grmzm2g014313_t01 0

grmzm2g014341_t04 0

grmzm2g014376_t01 0

grmzm2g014382_t02 0

grmzm2g014387_t01 0

grmzm2g014392_t01 0

grmzm2g014395_t01 0

grmzm2g014397_t02 0

grmzm2g014400_t02 0

grmzm2g014409_t01 0

grmzm2g014419_t02 0

grmzm2g014441_t01 0

grmzm2g014444_t04 0

grmzm2g014452_t01 0.83

grmzm2g014454_t01 0

grmzm2g014499_t01 0

grmzm2g014508_t01 0

grmzm2g014558_t01 0

grmzm2g014580_t01 0

grmzm2g014590_t01 -1.3

grmzm2g014612_t01 0

grmzm2g014653_t01 0

grmzm2g014676_t01 1.16

grmzm2g014695_t02 0

grmzm2g014709_t01 0

grmzm2g014720_t01 0

grmzm2g014750_t01 0

grmzm2g014770_t02 -0.89

grmzm2g014788_t01 0

grmzm2g014793_t01 0.82

grmzm2g014805_t01 0

grmzm2g014813_t05 0

grmzm2g014826_t01 0

grmzm2g014832_t02 0

grmzm2g014833_t01 0

grmzm2g014836_t01 0

grmzm2g014854_t01 0

grmzm2g014872_t02 0

grmzm2g014914_t03 0

grmzm2g014917_t01 0

grmzm2g014955_t01 0

grmzm2g014975_t02 0

grmzm2g014979_t01 0

grmzm2g014994_t02 0

grmzm2g015033_t01 0

grmzm2g015040_t04 0

grmzm2g015067_t02 0

grmzm2g015073_t01 0

grmzm2g015080_t02 0

grmzm2g015090_t01 0

grmzm2g015097_t01 0

grmzm2g015100_t01 0

grmzm2g015126_t01 0

grmzm2g015132_t01 0

grmzm2g015136_t01 0

grmzm2g015159_t01 0

grmzm2g015190_t01 0

grmzm2g015200_t01 0

grmzm2g015219_t02 0

grmzm2g015226_t01 0

grmzm2g015258_t01 0

grmzm2g015267_t01 0

grmzm2g015285_t01 0

grmzm2g015287_t03 0.84

grmzm2g015289_t01 0

grmzm2g015291_t02 0

grmzm2g015295_t03 0

grmzm2g015314_t01 0

grmzm2g015324_t02 0

grmzm2g015344_t01 0

grmzm2g015361_t05 0

grmzm2g015384_t01 0

grmzm2g015401_t01 0

grmzm2g015406_t01 0

grmzm2g015409_t01 0

grmzm2g015419_t05 -1.18

grmzm2g015432_t01 0

grmzm2g015433_t01 0

grmzm2g015436_t04 0

grmzm2g015502_t02 0

grmzm2g015514_t02 0

grmzm2g015571_t01 0

grmzm2g015578_t01 0

grmzm2g015588_t01 0

grmzm2g015592_t01 0

grmzm2g015596_t01 0

grmzm2g015603_t01 0

grmzm2g015605_t01 0

grmzm2g015610_t01 0

grmzm2g015642_t02 0

grmzm2g015692_t01 0

grmzm2g015703_t01 0

grmzm2g015709_t01 0

grmzm2g015727_t01 0

grmzm2g015730_t04 0

grmzm2g015735_t01 0

grmzm2g015767_t03 0

grmzm2g015784_t04 0

grmzm2g015804_t03 0

grmzm2g015854_t02 0

grmzm2g015861_t02 0

grmzm2g015869_t02 0

grmzm2g015875_t02 0

grmzm2g015880_t01 0

grmzm2g015881_t04 0

grmzm2g015887_t01 0

grmzm2g015889_t01 0

grmzm2g015902_t01 0

grmzm2g015906_t03 0

grmzm2g015925_t01 0

grmzm2g015955_t02 0

grmzm2g015967_t01 0

grmzm2g015972_t04 0

grmzm2g015983_t01 0

grmzm2g015989_t03 0

grmzm2g016066_t02 0

grmzm2g016084_t01 0

grmzm2g016088_t01 0

grmzm2g016097_t02 0

grmzm2g016150_t01 0

grmzm2g016153_t01 0

grmzm2g016168_t01 0

grmzm2g016184_t03 0

grmzm2g016232_t01 0

grmzm2g016236_t02 0

grmzm2g016241_t01 0

grmzm2g016250_t01 0

grmzm2g016290_t05 0

grmzm2g016296_t02 0

grmzm2g016323_t02 0

grmzm2g016382_t02 0

grmzm2g016439_t02 0

grmzm2g016447_t01 0

grmzm2g016462_t01 0

grmzm2g016480_t01 0

grmzm2g016503_t04 0

grmzm2g016511_t02 0

grmzm2g016546_t01 0

grmzm2g016551_t01 0

grmzm2g016581_t01 0

grmzm2g016592_t02 0

grmzm2g016602_t03 0

grmzm2g016605_t05 0

grmzm2g016622_t01 0

grmzm2g016644_t04 0

grmzm2g016649_t02 0

grmzm2g016651_t01 0

grmzm2g016655_t01 0

grmzm2g016657_t01 0

grmzm2g016660_t02 0

grmzm2g016668_t01 -1.51

grmzm2g016671_t02 0

grmzm2g016677_t01 0

grmzm2g016705_t02 0

grmzm2g016734_t02 0

grmzm2g016749_t01 0

grmzm2g016756_t01 -2.02

grmzm2g016774_t01 0

grmzm2g016802_t02 -0.83

grmzm2g016803_t01 0

grmzm2g016805_t01 0

grmzm2g016819_t01 0

grmzm2g016827_t01 0

grmzm2g016836_t02 -1.35

grmzm2g016844_t01 0

grmzm2g016858_t02 0

grmzm2g016866_t01 0

grmzm2g016875_t02 0

grmzm2g016878_t01 0

grmzm2g016890_t04 0

grmzm2g016892_t01 0

grmzm2g016894_t01 0

grmzm2g016926_t02 0

grmzm2g016928_t02 0

grmzm2g016930_t03 0

grmzm2g016939_t01 0

grmzm2g016958_t03 0

grmzm2g017013_t01 0

grmzm2g017016_t02 0

grmzm2g017031_t01 0

grmzm2g017045_t01 0

grmzm2g017047_t02 0

grmzm2g017077_t02 0

grmzm2g017080_t01 0

grmzm2g017086_t05 0

grmzm2g017089_t02 0

grmzm2g017110_t03 0

grmzm2g017142_t01 0

grmzm2g017145_t03 0

grmzm2g017159_t01 0

grmzm2g017164_t01 0

grmzm2g017186_t02 0

grmzm2g017187_t01 0

grmzm2g017193_t01 0

grmzm2g017249_t01 0

grmzm2g017254_t03 0

grmzm2g017257_t02 0

grmzm2g017266_t01 0

grmzm2g017268_t01 0.82

grmzm2g017269_t01 0

grmzm2g017290_t01 0

grmzm2g017305_t02 0

grmzm2g017319_t01 0

grmzm2g017329_t01 0

grmzm2g017351_t01 0

grmzm2g017355_t01 0

grmzm2g017365_t01 0

grmzm2g017368_t01 0

grmzm2g017382_t01 0

grmzm2g017386_t02 0

grmzm2g017388_t01 0

grmzm2g017404_t01 0

grmzm2g017409_t01 0

grmzm2g017414_t04 0

grmzm2g017419_t02 0

grmzm2g017421_t01 0

grmzm2g017426_t01 0

grmzm2g017429_t01 0

grmzm2g017460_t01 0

grmzm2g017484_t01 0

grmzm2g017486_t01 -1.76

grmzm2g017520_t01 0

grmzm2g017522_t01 0

grmzm2g017525_t02 0

grmzm2g017532_t02 0

grmzm2g017536_t01 0

grmzm2g017537_t02 0

grmzm2g017555_t01 0

grmzm2g017578_t01 0

grmzm2g017584_t01 0

grmzm2g017586_t03 0

grmzm2g017593_t01 0

grmzm2g017603_t01 0

grmzm2g017616_t01 0

grmzm2g017624_t01 0

grmzm2g017636_t01 0

grmzm2g017647_t01 0

grmzm2g017671_t01 0

grmzm2g017678_t01 0

grmzm2g017682_t01 0

grmzm2g017739_t01 0

grmzm2g017741_t01 0

grmzm2g017789_t02 0

grmzm2g017792_t01 0

grmzm2g017804_t02 0

grmzm2g017805_t03 0

grmzm2g017815_t01 0

grmzm2g017821_t01 0

grmzm2g017831_t01 0

grmzm2g017845_t02 0

grmzm2g017852_t01 -1.27

grmzm2g017853_t03 0

grmzm2g017865_t01 0

grmzm2g017923_t02 0

grmzm2g017925_t01 0

grmzm2g017933_t01 0

grmzm2g017940_t01 0

grmzm2g017941_t02 0

grmzm2g017957_t01 0

grmzm2g017966_t01 0

grmzm2g017991_t01 0

grmzm2g018006_t01 0

grmzm2g018020_t02 0

grmzm2g018027_t01 0

grmzm2g018044_t01 0

grmzm2g018059_t02 0

grmzm2g018070_t01 -0.99

grmzm2g018074_t01 0

grmzm2g018082_t01 0

grmzm2g018103_t01 0

grmzm2g018105_t03 0

grmzm2g018126_t01 0

grmzm2g018177_t03 0

grmzm2g018189_t01 0

grmzm2g018197_t01 0

grmzm2g018217_t01 0

grmzm2g018223_t01 0

grmzm2g018228_t02 0

grmzm2g018229_t05 0

grmzm2g018238_t01 0

grmzm2g018241_t01 0

grmzm2g018251_t01 0

grmzm2g018275_t01 0

grmzm2g018280_t01 0

grmzm2g018281_t02 0

grmzm2g018356_t01 0

grmzm2g018369_t01 0

grmzm2g018375_t01 0

grmzm2g018398_t01 0

grmzm2g018403_t01 0

grmzm2g018414_t02 0

grmzm2g018416_t01 0

grmzm2g018441_t02 0

grmzm2g018447_t02 0

grmzm2g018455_t01 0

grmzm2g018462_t03 0

grmzm2g018484_t01 0

grmzm2g018491_t01 0

grmzm2g018508_t02 0

grmzm2g018573_t02 0

grmzm2g018579_t01 0

grmzm2g018586_t01 0

grmzm2g018595_t05 0

grmzm2g018607_t01 0

grmzm2g018619_t03 0

grmzm2g018631_t01 0

grmzm2g018678_t01 0

grmzm2g018689_t01 0

grmzm2g018695_t01 0

grmzm2g018697_t04 0

grmzm2g018706_t02 0

grmzm2g018724_t03 0

grmzm2g018728_t01 0

grmzm2g018760_t01 0

grmzm2g018770_t01 0

grmzm2g018771_t02 0

grmzm2g018775_t01 0

grmzm2g018786_t04 0

grmzm2g018820_t02 -0.93

grmzm2g018823_t02 0

grmzm2g018837_t01 0

grmzm2g018869_t01 0

grmzm2g018885_t01 0.82

grmzm2g018901_t01 0

grmzm2g018926_t01 0

grmzm2g018929_t01 0

grmzm2g018941_t02 0

grmzm2g018943_t01 0

grmzm2g018947_t02 0

grmzm2g018950_t01 0

grmzm2g018951_t03 0

grmzm2g018955_t01 0

grmzm2g018971_t01 0

grmzm2g018984_t01 1.05

grmzm2g019029_t01 0

grmzm2g019050_t01 0

grmzm2g019056_t01 0

grmzm2g019084_t01 1.32

grmzm2g019106_t01 0

grmzm2g019119_t01 0

grmzm2g019121_t01 0

grmzm2g019144_t01 0

grmzm2g019166_t01 0

grmzm2g019171_t03 0

grmzm2g019177_t01 0

grmzm2g019183_t01 -1.16

grmzm2g019185_t01 0

grmzm2g019200_t01 0

grmzm2g019236_t04 0

grmzm2g019246_t01 0

grmzm2g019251_t01 0

grmzm2g019257_t01 0

grmzm2g019260_t01 0

grmzm2g019266_t03 0

grmzm2g019267_t01 0

grmzm2g019284_t01 0

grmzm2g019291_t04 0

grmzm2g019317_t02 0

grmzm2g019325_t01 0

grmzm2g019335_t02 0

grmzm2g019356_t01 -2.03

grmzm2g019358_t06 0

grmzm2g019363_t01 0

grmzm2g019373_t01 0

grmzm2g019386_t01 0

grmzm2g019404_t03 0

grmzm2g019411_t02 0

grmzm2g019413_t01 0

grmzm2g019434_t02 0

grmzm2g019437_t03 0

grmzm2g019446_t01 0

grmzm2g019450_t04 1

grmzm2g019468_t01 0

grmzm2g019500_t02 0

grmzm2g019501_t02 0

grmzm2g019515_t01 0

grmzm2g019538_t02 0

grmzm2g019553_t02 0

grmzm2g019586_t01 0

grmzm2g019596_t01 0

grmzm2g019597_t01 0

grmzm2g019604_t02 0

grmzm2g019641_t02 0

grmzm2g019673_t05 0

grmzm2g019681_t01 0

grmzm2g019689_t01 0

grmzm2g019695_t01 0

grmzm2g019721_t02 0

grmzm2g019738_t01 0

grmzm2g019744_t01 0

grmzm2g019746_t02 0

grmzm2g019777_t01 0

grmzm2g019783_t01 0

grmzm2g019807_t01 0

grmzm2g019810_t01 0

grmzm2g019812_t02 0

grmzm2g019838_t01 0

grmzm2g019863_t01 0

grmzm2g019872_t01 0

grmzm2g019874_t01 0

grmzm2g019876_t01 0

grmzm2g019879_t01 0

grmzm2g019901_t01 0

grmzm2g019919_t05 0

grmzm2g019926_t01 0

grmzm2g019958_t04 0

grmzm2g019971_t01 0

grmzm2g019974_t01 -1.18

grmzm2g019986_t01 0

grmzm2g019991_t01 0

grmzm2g019999_t01 0

grmzm2g020002_t04 0

grmzm2g020008_t01 0

grmzm2g020034_t03 0

grmzm2g020040_t01 0

grmzm2g020043_t01 0

grmzm2g020081_t02 0

grmzm2g020096_t01 0

grmzm2g020098_t03 0

grmzm2g020104_t01 0

grmzm2g020126_t01 0

grmzm2g020140_t01 0

grmzm2g020142_t01 0

grmzm2g020146_t01 0

grmzm2g020148_t01 0

grmzm2g020150_t01 0

grmzm2g020156_t01 0

grmzm2g020187_t02 0

grmzm2g020196_t01 0

grmzm2g020201_t01 0

grmzm2g020255_t02 0

grmzm2g020275_t02 0

grmzm2g020281_t04 0

grmzm2g020285_t01 0

grmzm2g020291_t01 0

grmzm2g020295_t01 0

grmzm2g020320_t01 0

grmzm2g020366_t01 0

grmzm2g020401_t04 0

grmzm2g020409_t01 0

grmzm2g020429_t01 1.11

grmzm2g020446_t01 0

grmzm2g020450_t01 0

grmzm2g020461_t01 0

grmzm2g020474_t03 0

grmzm2g020484_t05 0

grmzm2g020500_t01 -0.82

grmzm2g020544_t01 0

grmzm2g020548_t02 0

grmzm2g020574_t02 1.24

grmzm2g020594_t01 0

grmzm2g020620_t01 0

grmzm2g020627_t01 0

grmzm2g020631_t01 0

grmzm2g020653_t03 0

grmzm2g020661_t02 0

grmzm2g020666_t01 0

grmzm2g020721_t01 0

grmzm2g020728_t02 0

grmzm2g020761_t01 0

grmzm2g020766_t01 0

grmzm2g020772_t01 0

grmzm2g020775_t01 0

grmzm2g020785_t01 0

grmzm2g020801_t02 0

grmzm2g020805_t01 0

grmzm2g020814_t01 0

grmzm2g020840_t01 0

grmzm2g020843_t02 0

grmzm2g020856_t01 0

grmzm2g020864_t01 0

grmzm2g020893_t01 -1.33

grmzm2g020898_t01 0

grmzm2g020912_t02 0

grmzm2g020920_t02 0

grmzm2g020928_t01 0

grmzm2g020934_t01 0

grmzm2g020938_t01 0

grmzm2g020940_t01 -0.89

grmzm2g020943_t01 0

grmzm2g020974_t01 0

grmzm2g020982_t01 0

grmzm2g020986_t01 0

grmzm2g020996_t01 0

grmzm2g021015_t01 0

grmzm2g021044_t01 0

grmzm2g021055_t02 0

grmzm2g021074_t02 0

grmzm2g021101_t02 0

grmzm2g021106_t01 0

grmzm2g021107_t01 0

grmzm2g021110_t01 0

grmzm2g021129_t01 0

grmzm2g021149_t01 0

grmzm2g021170_t01 0

grmzm2g021194_t01 0

grmzm2g021219_t02 0

grmzm2g021223_t05 1.71

grmzm2g021225_t03 0

grmzm2g021243_t01 0

grmzm2g021256_t01 0

grmzm2g021299_t01 0

grmzm2g021313_t01 0

grmzm2g021331_t01 0

grmzm2g021339_t03 0

grmzm2g021379_t01 0

grmzm2g021406_t02 0

grmzm2g021416_t02 0

grmzm2g021422_t01 0

grmzm2g021464_t01 0

grmzm2g021468_t01 0

grmzm2g021470_t01 0

grmzm2g021471_t01 0

grmzm2g021517_t01 0

grmzm2g021530_t02 0

grmzm2g021549_t01 0

grmzm2g021560_t01 0

grmzm2g021567_t01 0

grmzm2g021589_t04 1.63

grmzm2g021598_t01 0

grmzm2g021605_t02 1.08

grmzm2g021619_t01 0

grmzm2g021621_t02 0

grmzm2g021635_t01 0

grmzm2g021661_t02 0

grmzm2g021674_t01 0

grmzm2g021687_t01 0

grmzm2g021694_t01 0

grmzm2g021704_t01 0

grmzm2g021706_t01 0

grmzm2g021742_t01 0

grmzm2g021746_t01 -1.01

grmzm2g021777_t02 0

grmzm2g021784_t01 0

grmzm2g021794_t01 -1.39

grmzm2g021802_t05 0

grmzm2g021816_t01 0

grmzm2g021822_t01 0

grmzm2g021834_t01 0

grmzm2g021846_t02 0

grmzm2g021864_t01 0

grmzm2g021877_t02 0

grmzm2g021879_t01 0

grmzm2g021885_t01 0

grmzm2g021912_t01 0

grmzm2g022014_t01 0

grmzm2g022019_t01 0

grmzm2g022032_t01 0

grmzm2g022041_t01 0

grmzm2g022050_t01 0

grmzm2g022054_t04 0

grmzm2g022061_t01 0

grmzm2g022088_t02 0

grmzm2g022090_t02 0

grmzm2g022107_t01 0

grmzm2g022120_t04 0

grmzm2g022159_t01 0

grmzm2g022175_t01 0

grmzm2g022180_t01 0

grmzm2g022181_t01 -1.51

grmzm2g022192_t03 0

grmzm2g022206_t01 0

grmzm2g022213_t01 0

grmzm2g022224_t01 0

grmzm2g022229_t01 0

grmzm2g022242_t01 0

grmzm2g022248_t01 0

grmzm2g022258_t04 0

grmzm2g022266_t01 0

grmzm2g022269_t02 0

grmzm2g022275_t02 0

grmzm2g022279_t01 0

grmzm2g022283_t01 0

grmzm2g022298_t01 0.89

grmzm2g022308_t02 0

grmzm2g022310_t01 0

grmzm2g022313_t01 0

grmzm2g022318_t02 0

grmzm2g022359_t02 0

grmzm2g022365_t01 0

grmzm2g022368_t01 0

grmzm2g022375_t03 -1.37

grmzm2g022398_t01 0

grmzm2g022402_t01 0

grmzm2g022413_t01 0

grmzm2g022453_t01 0

grmzm2g022480_t01 0

grmzm2g022499_t01 -1.91

grmzm2g022504_t02 0

grmzm2g022506_t02 0

grmzm2g022514_t01 0

grmzm2g022538_t01 0

grmzm2g022547_t02 0

grmzm2g022558_t01 0

grmzm2g022563_t01 0

grmzm2g022603_t02 0

grmzm2g022619_t02 0

grmzm2g022625_t01 0

grmzm2g022629_t01 0

grmzm2g022642_t07 0

grmzm2g022645_t01 0

grmzm2g022659_t01 0

grmzm2g022679_t01 0

grmzm2g022686_t02 0

grmzm2g022694_t01 0

grmzm2g022711_t04 0

grmzm2g022730_t01 1.93

grmzm2g022763_t01 0

grmzm2g022768_t02 0

grmzm2g022777_t01 0

grmzm2g022782_t03 0

grmzm2g022787_t02 0

grmzm2g022793_t01 0

grmzm2g022799_t01 0

grmzm2g022856_t01 0

grmzm2g022859_t01 0

grmzm2g022861_t01 0

grmzm2g022866_t02 0

grmzm2g022876_t01 0

grmzm2g022884_t01 0

grmzm2g022897_t02 0

grmzm2g022915_t01 0

grmzm2g022921_t01 0

grmzm2g022926_t02 0

grmzm2g022931_t01 0

grmzm2g022947_t01 0

grmzm2g022958_t01 0

grmzm2g022987_t01 0.88

grmzm2g022997_t02 0

grmzm2g023008_t02 0

grmzm2g023020_t01 0

grmzm2g023023_t01 0

grmzm2g023049_t01 0

grmzm2g023059_t01 0

grmzm2g023080_t01 0

grmzm2g023105_t01 0

grmzm2g023110_t01 0

grmzm2g023133_t02 0

grmzm2g023152_t01 1.65

grmzm2g023163_t01 0

grmzm2g023194_t01 0

grmzm2g023204_t01 0

grmzm2g023220_t02 -1.29

grmzm2g023232_t01 0

grmzm2g023237_t01 0

grmzm2g023239_t02 0

grmzm2g023242_t01 0

grmzm2g023257_t01 0

grmzm2g023275_t01 0

grmzm2g023279_t01 0

grmzm2g023289_t02 0

grmzm2g023293_t01 0

grmzm2g023313_t01 0

grmzm2g023325_t01 0

grmzm2g023328_t04 0

grmzm2g023347_t02 0

grmzm2g023387_t02 0

grmzm2g023392_t01 0

grmzm2g023418_t01 0

grmzm2g023436_t01 0

grmzm2g023438_t01 0

grmzm2g023444_t02 0

grmzm2g023475_t02 0

grmzm2g023520_t01 0

grmzm2g023528_t04 0

grmzm2g023563_t03 0

grmzm2g023575_t01 0

grmzm2g023585_t01 0

grmzm2g023591_t02 0

grmzm2g023625_t01 0

grmzm2g023636_t01 0

grmzm2g023638_t02 0

grmzm2g023652_t02 0

grmzm2g023667_t02 0

grmzm2g023711_t02 0

grmzm2g023715_t02 0

grmzm2g023748_t02 0

grmzm2g023755_t03 2.27

grmzm2g023769_t01 0

grmzm2g023791_t01 0

grmzm2g023798_t02 0

grmzm2g023833_t01 0

grmzm2g023836_t01 0

grmzm2g023840_t01 0

grmzm2g023858_t02 0

grmzm2g023884_t01 0

grmzm2g023906_t01 0

grmzm2g023946_t01 0

grmzm2g023970_t03 0

grmzm2g023973_t01 0

grmzm2g023982_t01 0

grmzm2g023983_t01 0

grmzm2g023988_t01 0

grmzm2g023992_t03 1.25

grmzm2g023995_t01 0

grmzm2g024051_t01 0

grmzm2g024054_t07 0

grmzm2g024071_t01 0

grmzm2g024073_t01 0

grmzm2g024099_t01 -1.11

grmzm2g024104_t04 0

grmzm2g024145_t02 0

grmzm2g024150_t01 0

grmzm2g024151_t01 0

grmzm2g024159_t01 0

grmzm2g024180_t03 0

grmzm2g024211_t02 0

grmzm2g024231_t03 0

grmzm2g024247_t01 0

grmzm2g024260_t01 0

grmzm2g024264_t02 0

grmzm2g024267_t01 0

grmzm2g024293_t03 0

grmzm2g024303_t01 0

grmzm2g024310_t02 0

grmzm2g024315_t02 0

grmzm2g024335_t01 0

grmzm2g024348_t01 0

grmzm2g024354_t01 0

grmzm2g024389_t03 0

grmzm2g024391_t01 0

grmzm2g024395_t01 0

grmzm2g024437_t01 0

grmzm2g024451_t01 -0.94

grmzm2g024466_t01 0

grmzm2g024477_t01 0

grmzm2g024482_t01 0

grmzm2g024517_t01 -1.56

grmzm2g024530_t01 0

grmzm2g024533_t01 0

grmzm2g024550_t01 0

grmzm2g024551_t01 0

grmzm2g024563_t01 0

grmzm2g024576_t02 0

grmzm2g024607_t01 0

grmzm2g024612_t01 0

grmzm2g024615_t03 0

grmzm2g024626_t02 0

grmzm2g024647_t07 0

grmzm2g024655_t01 0

grmzm2g024657_t01 0

grmzm2g024668_t02 -1.27

grmzm2g024686_t01 0

grmzm2g024690_t01 0

grmzm2g024693_t01 0

grmzm2g024708_t01 0

grmzm2g024730_t01 0

grmzm2g024733_t01 0

grmzm2g024738_t02 0

grmzm2g024739_t03 0

grmzm2g024785_t01 0

grmzm2g024799_t01 0

grmzm2g024806_t01 0

grmzm2g024808_t01 0

grmzm2g024811_t01 0

grmzm2g024823_t02 0

grmzm2g024838_t01 0

grmzm2g024851_t01 0

grmzm2g024865_t01 0

grmzm2g024882_t01 0

grmzm2g024910_t04 0

grmzm2g024973_t01 -1.29

grmzm2g024976_t01 0

grmzm2g024992_t01 0

grmzm2g025014_t01 0

grmzm2g025024_t01 0

grmzm2g025031_t03 0

grmzm2g025037_t01 0

grmzm2g025054_t01 0

grmzm2g025059_t01 0

grmzm2g025072_t01 0

grmzm2g025074_t01 0

grmzm2g025078_t01 0

grmzm2g025109_t01 0

grmzm2g025113_t03 0

grmzm2g025123_t01 0

grmzm2g025126_t01 0

grmzm2g025127_t02 0

grmzm2g025154_t02 0

grmzm2g025164_t01 0

grmzm2g025171_t01 0

grmzm2g025182_t02 -0.98

grmzm2g025214_t03 0

grmzm2g025215_t01 0

grmzm2g025227_t02 0

grmzm2g025231_t02 0

grmzm2g025236_t01 0

grmzm2g025242_t01 0

grmzm2g025243_t02 0.82

grmzm2g025248_t03 0

grmzm2g025255_t01 0

grmzm2g025281_t03 0

grmzm2g025294_t01 0

grmzm2g025322_t01 0

grmzm2g025329_t01 0

grmzm2g025340_t03 0

grmzm2g025356_t02 0

grmzm2g025366_t01 0

grmzm2g025387_t03 0

grmzm2g025409_t02 0

grmzm2g025414_t01 0

grmzm2g025451_t01 0

grmzm2g025459_t02 0

grmzm2g025470_t01 0

grmzm2g025480_t03 0

grmzm2g025488_t01 0

grmzm2g025528_t06 0

grmzm2g025531_t01 0

grmzm2g025536_t01 0

grmzm2g025552_t04 0

grmzm2g025592_t01 0

grmzm2g025594_t02 0

grmzm2g025598_t02 0

grmzm2g025611_t01 0

grmzm2g025646_t02 0

grmzm2g025648_t02 0

grmzm2g025659_t01 0.88

grmzm2g025671_t01 0

grmzm2g025703_t02 0

grmzm2g025731_t01 0

grmzm2g025742_t01 0

grmzm2g025783_t01 0

grmzm2g025806_t05 0

grmzm2g025812_t03 -1.04

grmzm2g025832_t01 0

grmzm2g025854_t03 0

grmzm2g025855_t01 0

grmzm2g025860_t01 0

grmzm2g025867_t01 0

grmzm2g025870_t03 0

grmzm2g025882_t01 0

grmzm2g025885_t01 0

grmzm2g025906_t02 0

grmzm2g025924_t01 0

grmzm2g025939_t01 0

grmzm2g025954_t02 0

grmzm2g025966_t01 0

grmzm2g025977_t04 0

grmzm2g025992_t01 0

grmzm2g026013_t01 0

grmzm2g026015_t04 0

grmzm2g026024_t01 0

grmzm2g026043_t01 0

grmzm2g026050_t02 0

grmzm2g026065_t01 0

grmzm2g026085_t01 0

grmzm2g026095_t01 0

grmzm2g026117_t03 0

grmzm2g026143_t01 0

grmzm2g026147_t04 -1.16

grmzm2g026151_t01 0

grmzm2g026180_t01 0

grmzm2g026203_t01 1.32

grmzm2g026216_t03 0

grmzm2g026231_t01 0

grmzm2g026301_t01 0

grmzm2g026309_t02 0

grmzm2g026311_t02 0

grmzm2g026371_t02 0

grmzm2g026391_t02 0

grmzm2g026417_t01 0

grmzm2g026442_t01 0

grmzm2g026447_t01 0

grmzm2g026459_t01 -1.32

grmzm2g026470_t01 0

grmzm2g026490_t01 0

grmzm2g026521_t01 0

grmzm2g026523_t01 0

grmzm2g026576_t01 0

grmzm2g026614_t03 0

grmzm2g026639_t01 0

grmzm2g026643_t01 0

grmzm2g026654_t01 0

grmzm2g026656_t01 0

grmzm2g026672_t01 0

grmzm2g026702_t01 0

grmzm2g026742_t01 0

grmzm2g026758_t01 0

grmzm2g026767_t01 0

grmzm2g026780_t01 0

grmzm2g026793_t01 0

grmzm2g026800_t01 0

grmzm2g026807_t01 0

grmzm2g026833_t01 0

grmzm2g026835_t01 0

grmzm2g026839_t01 0

grmzm2g026855_t03 0.84

grmzm2g026868_t01 0

grmzm2g026869_t02 0

grmzm2g026881_t01 0

grmzm2g026889_t01 0

grmzm2g026892_t01 0

grmzm2g026910_t01 -1.74

grmzm2g026922_t01 0

grmzm2g026927_t01 0

grmzm2g026930_t01 0

grmzm2g026943_t02 0.81

grmzm2g026952_t01 0

grmzm2g026962_t02 0

grmzm2g026969_t01 0

grmzm2g026980_t02 0

grmzm2g026991_t01 0

grmzm2g027019_t01 0

grmzm2g027021_t03 0

grmzm2g027043_t01 0

grmzm2g027047_t03 0

grmzm2g027049_t01 0

grmzm2g027059_t01 0

grmzm2g027068_t02 0

grmzm2g027075_t01 0

grmzm2g027098_t01 0

grmzm2g027105_t01 0

grmzm2g027115_t01 0

grmzm2g027120_t01 0

grmzm2g027131_t01 0

grmzm2g027166_t01 0

grmzm2g027183_t02 0

grmzm2g027193_t01 0

grmzm2g027209_t01 0

grmzm2g027219_t01 0

grmzm2g027232_t03 0

grmzm2g027241_t01 0

grmzm2g027272_t01 0

grmzm2g027282_t01 0

grmzm2g027331_t07 0

grmzm2g027333_t02 0

grmzm2g027344_t01 0

grmzm2g027375_t01 0

grmzm2g027378_t01 0

grmzm2g027392_t01 0

grmzm2g027420_t01 0

grmzm2g027431_t01 0

grmzm2g027437_t01 0

grmzm2g027441_t01 0

grmzm2g027451_t05 0

grmzm2g027462_t02 0

grmzm2g027478_t03 0

grmzm2g027497_t01 0

grmzm2g027499_t01 0

grmzm2g027535_t02 0

grmzm2g027546_t03 0

grmzm2g027571_t01 0

grmzm2g027592_t01 0

grmzm2g027603_t01 0

grmzm2g027640_t01 0

grmzm2g027663_t02 0

grmzm2g027665_t01 0

grmzm2g027673_t01 0

grmzm2g027695_t01 0

grmzm2g027723_t01 0

grmzm2g027726_t01 0

grmzm2g027728_t02 0

grmzm2g027737_t01 0

grmzm2g027750_t01 0

grmzm2g027756_t01 0

grmzm2g027794_t01 0

grmzm2g027821_t02 0

grmzm2g027825_t01 0

grmzm2g027835_t01 0

grmzm2g027839_t01 0

grmzm2g027851_t02 0

grmzm2g027860_t02 0

grmzm2g027875_t01 0

grmzm2g027886_t01 0

grmzm2g027891_t01 0

grmzm2g027932_t01 0

grmzm2g027955_t01 0

grmzm2g027958_t01 0

grmzm2g027972_t01 1.11

grmzm2g027991_t01 0

grmzm2g027995_t02 0

grmzm2g028004_t01 0

grmzm2g028005_t01 0

grmzm2g028007_t01 0

grmzm2g028016_t02 0

grmzm2g028036_t01 0

grmzm2g028037_t02 0

grmzm2g028039_t02 0

grmzm2g028054_t04 0

grmzm2g028070_t02 0

grmzm2g028089_t01 0

grmzm2g028096_t03 0

grmzm2g028104_t01 0

grmzm2g028108_t02 0

grmzm2g028110_t01 0

grmzm2g028129_t03 0

grmzm2g028132_t02 0

grmzm2g028134_t01 0

grmzm2g028136_t01 0

grmzm2g028139_t01 0

grmzm2g028156_t01 0

grmzm2g028183_t04 0

grmzm2g028190_t04 0

grmzm2g028216_t02 0

grmzm2g028218_t01 0

grmzm2g028234_t01 0

grmzm2g028249_t01 0

grmzm2g028258_t02 0

grmzm2g028266_t01 0

grmzm2g028286_t03 0

grmzm2g028302_t01 0

grmzm2g028307_t01 0

grmzm2g028313_t01 0

grmzm2g028325_t02 0

grmzm2g028346_t01 0

grmzm2g028353_t01 0

grmzm2g028369_t01 0

grmzm2g028379_t02 0

grmzm2g028393_t02 -1.35

grmzm2g028413_t02 0

grmzm2g028432_t01 0

grmzm2g028467_t01 0

grmzm2g028492_t01 0.93

grmzm2g028500_t01 0

grmzm2g028501_t01 0

grmzm2g028516_t02 0

grmzm2g028521_t01 0

grmzm2g028556_t03 0

grmzm2g028568_t02 0

grmzm2g028587_t01 0

grmzm2g028594_t03 0

grmzm2g028604_t01 -1.04

grmzm2g028609_t01 0

grmzm2g028637_t02 0

grmzm2g028640_t01 0

grmzm2g028656_t01 -1.35

grmzm2g028665_t03 0

grmzm2g028676_t02 0

grmzm2g028690_t01 0

grmzm2g028700_t01 0

grmzm2g028709_t01 0

grmzm2g028718_t02 0

grmzm2g028730_t01 0

grmzm2g028758_t02 0

grmzm2g028763_t02 0

grmzm2g028766_t01 0

grmzm2g028796_t01 0

grmzm2g028821_t02 0

grmzm2g028834_t01 0

grmzm2g028855_t01 0

grmzm2g028883_t01 0

grmzm2g028900_t01 0

grmzm2g028902_t02 0

grmzm2g028905_t01 0

grmzm2g028914_t03 0

grmzm2g028921_t02 0

grmzm2g028924_t01 0

grmzm2g028926_t01 0

grmzm2g028928_t02 0

grmzm2g028929_t01 0

grmzm2g028945_t03 0

grmzm2g028955_t01 0

grmzm2g028980_t01 0

grmzm2g028986_t03 0

grmzm2g028988_t01 0

grmzm2g029001_t01 0

grmzm2g029027_t03 0

grmzm2g029029_t01 0

grmzm2g029039_t01 0

grmzm2g029048_t01 0

grmzm2g029055_t02 0

grmzm2g029058_t01 0

grmzm2g029077_t01 0

grmzm2g029082_t01 0

grmzm2g029087_t03 0

grmzm2g029101_t01 0

grmzm2g029107_t02 0

grmzm2g029113_t01 0

grmzm2g029135_t01 0

grmzm2g029148_t01 0

grmzm2g029153_t01 0

grmzm2g029186_t04 0

grmzm2g029194_t01 0

grmzm2g029211_t01 0

grmzm2g029219_t02 1.02

grmzm2g029242_t01 0

grmzm2g029258_t01 0

grmzm2g029262_t02 0

grmzm2g029307_t04 0

grmzm2g029323_t01 0

grmzm2g029356_t01 0

grmzm2g029370_t01 0

grmzm2g029385_t01 0

grmzm2g029396_t02 0

grmzm2g029407_t01 0

grmzm2g029455_t01 0

grmzm2g029478_t04 0

grmzm2g029486_t01 0

grmzm2g029514_t01 0

grmzm2g029519_t01 0

grmzm2g029527_t01 0

grmzm2g029530_t01 0

grmzm2g029543_t01 0

grmzm2g029547_t03 0

grmzm2g029559_t01 0

grmzm2g029566_t03 0

grmzm2g029573_t03 0

grmzm2g029583_t03 0

grmzm2g029587_t01 0

grmzm2g029617_t01 0

grmzm2g029632_t01 0

grmzm2g029641_t02 0

grmzm2g029685_t01 0

grmzm2g029692_t03 0

grmzm2g029698_t02 0

grmzm2g029713_t01 0

grmzm2g029731_t01 0

grmzm2g029824_t03 0

grmzm2g029845_t01 0

grmzm2g029856_t01 0

grmzm2g029863_t02 -0.94

grmzm2g029879_t01 0

grmzm2g029912_t01 -1.1

grmzm2g029933_t01 0

grmzm2g029951_t01 0

grmzm2g029974_t01 0

grmzm2g030016_t02 0

grmzm2g030038_t01 0

grmzm2g030072_t03 0

grmzm2g030080_t01 0

grmzm2g030099_t02 0

grmzm2g030125_t01 0

grmzm2g030138_t01 -1.62

grmzm2g030139_t02 0

grmzm2g030144_t02 0

grmzm2g030165_t01 0

grmzm2g030167_t01 0

grmzm2g030169_t03 0

grmzm2g030189_t01 0

grmzm2g030203_t01 0

grmzm2g030216_t01 0

grmzm2g030223_t01 0

grmzm2g030228_t02 0

grmzm2g030235_t05 0

grmzm2g030240_t01 0

grmzm2g030241_t01 0

grmzm2g030252_t01 0

grmzm2g030272_t01 0

grmzm2g030275_t03 0

grmzm2g030280_t02 0

grmzm2g030293_t01 0

grmzm2g030299_t02 0

grmzm2g030325_t01 0

grmzm2g030341_t02 0

grmzm2g030350_t01 0

grmzm2g030384_t02 0

grmzm2g030408_t01 0

grmzm2g030422_t01 0

grmzm2g030494_t02 0

grmzm2g030510_t01 0

grmzm2g030529_t01 0

grmzm2g030542_t01 0

grmzm2g030557_t01 0

grmzm2g030567_t02 0

grmzm2g030570_t01 0

grmzm2g030578_t01 0

grmzm2g030594_t01 0

grmzm2g030596_t01 0

grmzm2g030598_t01 0

grmzm2g030606_t03 0

grmzm2g030628_t01 0

grmzm2g030636_t02 0

grmzm2g030646_t02 0

grmzm2g030659_t01 0

grmzm2g030673_t01 0

grmzm2g030692_t01 0.86

grmzm2g030710_t01 0

grmzm2g030712_t01 0

grmzm2g030713_t01 0

grmzm2g030731_t01 0

grmzm2g030744_t03 0

grmzm2g030762_t02 -1.28

grmzm2g030784_t01 0

grmzm2g030794_t01 0

grmzm2g030805_t01 0

grmzm2g030809_t01 0

grmzm2g030823_t01 0

grmzm2g030839_t07 0

grmzm2g030858_t01 0

grmzm2g030873_t01 0

grmzm2g030880_t01 0

grmzm2g030882_t01 -0.94

grmzm2g030902_t04 0

grmzm2g030915_t09 0

grmzm2g030955_t01 0

grmzm2g031001_t01 0

grmzm2g031022_t01 0

grmzm2g031028_t02 0

grmzm2g031043_t01 0

grmzm2g031053_t01 0

grmzm2g031057_t01 0

grmzm2g031065_t01 0

grmzm2g031107_t03 0

grmzm2g031117_t04 0

grmzm2g031138_t01 0

grmzm2g031143_t06 -1.25

grmzm2g031169_t01 0

grmzm2g031177_t03 0

grmzm2g031200_t02 0

grmzm2g031210_t02 0

grmzm2g031213_t04 0

grmzm2g031239_t01 0

grmzm2g031261_t02 0

grmzm2g031263_t01 0.91

grmzm2g031298_t02 0

grmzm2g031308_t02 0

grmzm2g031311_t01 0

grmzm2g031317_t02 0

grmzm2g031326_t01 -1.32

grmzm2g031338_t01 -1.47

grmzm2g031352_t01 0

grmzm2g031354_t01 0

grmzm2g031360_t01 0

grmzm2g031370_t01 0

grmzm2g031398_t02 0

grmzm2g031400_t01 0

grmzm2g031420_t01 0

grmzm2g031435_t01 0

grmzm2g031447_t01 0

grmzm2g031461_t01 0

grmzm2g031496_t01 0

grmzm2g031501_t04 0

grmzm2g031504_t01 0

grmzm2g031523_t02 0

grmzm2g031529_t01 0

grmzm2g031536_t01 0

grmzm2g031545_t04 0

grmzm2g031568_t01 0

grmzm2g031572_t01 0

grmzm2g031580_t01 0

grmzm2g031581_t01 0

grmzm2g031584_t01 0

grmzm2g031586_t02 0

grmzm2g031591_t01 0

grmzm2g031609_t01 0

grmzm2g031613_t02 -0.99

grmzm2g031625_t01 0

grmzm2g031637_t01 -1.65

grmzm2g031656_t01 0

grmzm2g031660_t01 0

grmzm2g031718_t01 0

grmzm2g031721_t03 0

grmzm2g031761_t04 0

grmzm2g031780_t01 0

grmzm2g031790_t01 -1.26

grmzm2g031802_t01 0

grmzm2g031824_t01 0

grmzm2g031825_t05 0

grmzm2g031827_t01 0.93

grmzm2g031846_t01 0

grmzm2g031850_t01 0

grmzm2g031859_t02 0

grmzm2g031904_t01 0

grmzm2g031917_t01 0

grmzm2g031930_t02 0

grmzm2g031938_t01 0

grmzm2g031941_t01 0

grmzm2g031952_t01 0

grmzm2g031954_t01 0

grmzm2g031981_t01 0

grmzm2g031983_t01 0

grmzm2g032003_t02 0

grmzm2g032022_t01 0

grmzm2g032028_t01 0

grmzm2g032044_t01 0

grmzm2g032049_t01 0

grmzm2g032071_t01 0

grmzm2g032107_t01 0

grmzm2g032110_t03 0

grmzm2g032132_t01 0

grmzm2g032142_t01 0

grmzm2g032160_t01 0

grmzm2g032163_t02 0

grmzm2g032182_t02 0

grmzm2g032190_t01 0

grmzm2g032214_t01 0

grmzm2g032218_t01 0

grmzm2g032219_t02 0

grmzm2g032222_t03 0

grmzm2g032225_t01 0

grmzm2g032252_t01 0

grmzm2g032253_t01 0

grmzm2g032258_t01 0

grmzm2g032260_t03 0

grmzm2g032266_t01 0

grmzm2g032276_t01 1.64

grmzm2g032280_t01 0

grmzm2g032282_t01 0

grmzm2g032293_t01 0

grmzm2g032295_t01 0

grmzm2g032314_t01 0

grmzm2g032315_t02 0

grmzm2g032336_t01 0

grmzm2g032337_t02 0

grmzm2g032348_t06 0

grmzm2g032351_t02 0

grmzm2g032367_t04 0

grmzm2g032376_t03 0

grmzm2g032409_t01 0

grmzm2g032423_t01 0

grmzm2g032430_t01 0

grmzm2g032447_t01 0

grmzm2g032475_t01 0

grmzm2g032484_t01 0

grmzm2g032496_t01 0

grmzm2g032505_t01 0

grmzm2g032564_t01 0

grmzm2g032594_t01 0

grmzm2g032602_t01 0

grmzm2g032619_t04 0

grmzm2g032648_t01 -1.6

grmzm2g032684_t02 0

grmzm2g032694_t03 0

grmzm2g032699_t02 0

grmzm2g032711_t01 0

grmzm2g032763_t01 0

grmzm2g032766_t01 0

grmzm2g032807_t01 0

grmzm2g032821_t01 0

grmzm2g032840_t01 0

grmzm2g032847_t01 0

grmzm2g032852_t03 0

grmzm2g032856_t01 0

grmzm2g032896_t01 0

grmzm2g032910_t05 0

grmzm2g032936_t01 0

grmzm2g032944_t01 0

grmzm2g032955_t01 0

grmzm2g032958_t01 0

grmzm2g033022_t01 0

grmzm2g033029_t01 0

grmzm2g033074_t01 0

grmzm2g033093_t01 0

grmzm2g033098_t01 0

grmzm2g033117_t01 0

grmzm2g033130_t05 0

grmzm2g033135_t01 0

grmzm2g033138_t06 0

grmzm2g033198_t01 0

grmzm2g033199_t04 0

grmzm2g033208_t02 0

grmzm2g033219_t01 0

grmzm2g033245_t03 0

grmzm2g033267_t01 0

grmzm2g033283_t01 0

grmzm2g033348_t01 0

grmzm2g033352_t02 0

grmzm2g033356_t01 0

grmzm2g033379_t01 0

grmzm2g033430_t02 0

grmzm2g033478_t03 0

grmzm2g033489_t02 0

grmzm2g033491_t02 0

grmzm2g033493_t02 0

grmzm2g033521_t01 0

grmzm2g033526_t01 0

grmzm2g033555_t01 0

grmzm2g033570_t01 0

grmzm2g033592_t02 0

grmzm2g033619_t02 0

grmzm2g033626_t04 0

grmzm2g033630_t01 0

grmzm2g033634_t04 0

grmzm2g033641_t01 0

grmzm2g033649_t01 -1.21

grmzm2g033653_t01 0

grmzm2g033661_t01 0

grmzm2g033671_t02 0

grmzm2g033680_t01 0

grmzm2g033694_t01 0

grmzm2g033724_t02 0

grmzm2g033746_t06 0

grmzm2g033787_t01 -1.44

grmzm2g033799_t01 0

grmzm2g033805_t01 0

grmzm2g033820_t01 0

grmzm2g033829_t01 0

grmzm2g033846_t01 0

grmzm2g033867_t01 0

grmzm2g033876_t01 0

grmzm2g033885_t02 0

grmzm2g033894_t02 0

grmzm2g033926_t04 0

grmzm2g033930_t01 0

grmzm2g033931_t01 0

grmzm2g033937_t01 0

grmzm2g033962_t01 0

grmzm2g033971_t01 0

grmzm2g034005_t01 0

grmzm2g034013_t01 0

grmzm2g034015_t01 0

grmzm2g034019_t01 0

grmzm2g034025_t01 0

grmzm2g034043_t01 0

grmzm2g034052_t01 0

grmzm2g034061_t01 0

grmzm2g034065_t01 0

grmzm2g034069_t01 0

grmzm2g034083_t01 0

grmzm2g034096_t02 0

grmzm2g034110_t01 0

grmzm2g034120_t02 0

grmzm2g034122_t01 0

grmzm2g034143_t03 0

grmzm2g034152_t01 0

grmzm2g034183_t01 0

grmzm2g034217_t01 0

grmzm2g034225_t03 0

grmzm2g034243_t01 0

grmzm2g034260_t03 0

grmzm2g034276_t03 0

grmzm2g034288_t01 0

grmzm2g034302_t02 0

grmzm2g034313_t01 0

grmzm2g034318_t01 0

grmzm2g034326_t01 0

grmzm2g034342_t01 0

grmzm2g034360_t01 0

grmzm2g034362_t01 0

grmzm2g034383_t02 0

grmzm2g034410_t03 0

grmzm2g034417_t02 0

grmzm2g034430_t01 0

grmzm2g034453_t01 0

grmzm2g034471_t01 0

grmzm2g034485_t01 0

grmzm2g034503_t04 0

grmzm2g034511_t04 0

grmzm2g034526_t02 0

grmzm2g034536_t03 0

grmzm2g034550_t01 -1.22

grmzm2g034551_t01 0

grmzm2g034572_t02 0

grmzm2g034573_t01 0

grmzm2g034575_t04 0

grmzm2g034598_t01 0

grmzm2g034611_t01 0

grmzm2g034622_t03 0

grmzm2g034626_t03 0

grmzm2g034631_t01 0

grmzm2g034638_t01 -1

grmzm2g034639_t01 0

grmzm2g034645_t01 0

grmzm2g034684_t01 0

grmzm2g034690_t01 0

grmzm2g034697_t02 0

grmzm2g034724_t03 0

grmzm2g034764_t01 0

grmzm2g034794_t02 0

grmzm2g034804_t08 0

grmzm2g034843_t01 0

grmzm2g034855_t03 0

grmzm2g034882_t01 0

grmzm2g034943_t02 0

grmzm2g034975_t01 0

grmzm2g034985_t02 0

grmzm2g035008_t02 0

grmzm2g035017_t01 0

grmzm2g035042_t01 0

grmzm2g035068_t02 1.05

grmzm2g035073_t01 0

grmzm2g035103_t01 0

grmzm2g035118_t01 0

grmzm2g035131_t02 0

grmzm2g035134_t05 0

grmzm2g035150_t03 0

grmzm2g035153_t01 0

grmzm2g035202_t01 0

grmzm2g035213_t02 0

grmzm2g035217_t01 0

grmzm2g035222_t01 0

grmzm2g035243_t01 0

grmzm2g035256_t01 0

grmzm2g035268_t01 0

grmzm2g035278_t01 0

grmzm2g035282_t01 0

grmzm2g035285_t01 0

grmzm2g035325_t01 0

grmzm2g035338_t01 0

grmzm2g035341_t01 0

grmzm2g035347_t01 0

grmzm2g035356_t01 0

grmzm2g035395_t01 0

grmzm2g035401_t01 0

grmzm2g035405_t02 0

grmzm2g035417_t01 0

grmzm2g035421_t01 0

grmzm2g035430_t01 0

grmzm2g035444_t01 0

grmzm2g035445_t01 0

grmzm2g035461_t01 0

grmzm2g035465_t05 0

grmzm2g035477_t01 0

grmzm2g035502_t01 0

grmzm2g035503_t01 -1.15

grmzm2g035517_t01 0

grmzm2g035520_t02 0

grmzm2g035526_t02 0

grmzm2g035528_t02 0

grmzm2g035531_t01 0

grmzm2g035533_t01 0

grmzm2g035557_t01 0

grmzm2g035574_t01 0

grmzm2g035579_t01 0

grmzm2g035584_t04 0

grmzm2g035594_t02 0

grmzm2g035595_t01 0

grmzm2g035599_t01 0

grmzm2g035601_t01 0

grmzm2g035620_t01 0

grmzm2g035632_t01 0

grmzm2g035665_t01 0

grmzm2g035708_t07 0

grmzm2g035709_t02 0.92

grmzm2g035719_t02 0

grmzm2g035726_t01 0

grmzm2g035741_t04 0

grmzm2g035749_t01 -1.04

grmzm2g035779_t01 0

grmzm2g035785_t01 0

grmzm2g035807_t01 0

grmzm2g035809_t02 0

grmzm2g035820_t01 0

grmzm2g035821_t02 0

grmzm2g035843_t01 0

grmzm2g035849_t01 0

grmzm2g035899_t03 0

grmzm2g035922_t01 0

grmzm2g035928_t01 0

grmzm2g035933_t01 0

grmzm2g035944_t01 0

grmzm2g035948_t01 0

grmzm2g035960_t01 0

grmzm2g035985_t01 0

grmzm2g035997_t03 0

grmzm2g036007_t03 0

grmzm2g036019_t02 0

grmzm2g036034_t01 0

grmzm2g036050_t01 0

grmzm2g036086_t01 0

grmzm2g036092_t02 0

grmzm2g036099_t03 0

grmzm2g036134_t01 0

grmzm2g036169_t01 0

grmzm2g036186_t02 0

grmzm2g036217_t01 0

grmzm2g036290_t01 0

grmzm2g036340_t01 0

grmzm2g036351_t01 0

grmzm2g036409_t01 0

grmzm2g036418_t02 0

grmzm2g036427_t01 0

grmzm2g036448_t04 0

grmzm2g036455_t01 0

grmzm2g036464_t02 0

grmzm2g036505_t02 0

grmzm2g036534_t02 0

grmzm2g036543_t01 0

grmzm2g036564_t01 0

grmzm2g036567_t01 0

grmzm2g036590_t01 0

grmzm2g036596_t03 0

grmzm2g036609_t01 0

grmzm2g036640_t01 0

grmzm2g036650_t04 0

grmzm2g036685_t02 0

grmzm2g036697_t01 0

grmzm2g036708_t01 0

grmzm2g036720_t02 0

grmzm2g036765_t01 0

grmzm2g036826_t01 0

grmzm2g036829_t02 0

grmzm2g036837_t01 0

grmzm2g036861_t04 0

grmzm2g036872_t01 0

grmzm2g036880_t01 0

grmzm2g036908_t03 -0.93

grmzm2g036916_t02 0

grmzm2g036918_t01 0

grmzm2g036921_t01 0

grmzm2g036927_t01 0

grmzm2g036940_t01 0

grmzm2g036966_t02 0

grmzm2g036991_t03 0

grmzm2g036996_t01 0

grmzm2g037015_t01 0

grmzm2g037048_t01 0

grmzm2g037064_t03 0

grmzm2g037104_t02 0

grmzm2g037128_t02 0

grmzm2g037130_t02 0

grmzm2g037140_t03 0

grmzm2g037146_t01 0

grmzm2g037150_t01 0

grmzm2g037152_t01 0

grmzm2g037164_t01 0

grmzm2g037177_t01 0

grmzm2g037185_t01 0

grmzm2g037189_t01 -0.88

grmzm2g037193_t01 0

grmzm2g037200_t01 0

grmzm2g037204_t02 0

grmzm2g037209_t04 0

grmzm2g037226_t01 0

grmzm2g037255_t01 0

grmzm2g037265_t04 -1.05

grmzm2g037284_t01 0

grmzm2g037308_t01 0

grmzm2g037322_t01 -0.84

grmzm2g037335_t01 0

grmzm2g037342_t01 0

grmzm2g037350_t01 0

grmzm2g037368_t01 0

grmzm2g037378_t01 0

grmzm2g037379_t02 0

grmzm2g037386_t02 0

grmzm2g037422_t01 0

grmzm2g037431_t01 0

grmzm2g037444_t01 0

grmzm2g037452_t03 0.89

grmzm2g037469_t01 0

grmzm2g037585_t02 0

grmzm2g037614_t01 0

grmzm2g037624_t02 0

grmzm2g037627_t01 0

grmzm2g037644_t01 0

grmzm2g037650_t01 0

grmzm2g037655_t01 0

grmzm2g037683_t01 0

grmzm2g037685_t02 0

grmzm2g037694_t01 0

grmzm2g037698_t01 0

grmzm2g037725_t01 0

grmzm2g037743_t01 0

grmzm2g037770_t01 0

grmzm2g037781_t01 0

grmzm2g037792_t01 0

grmzm2g037823_t01 0

grmzm2g037865_t02 0

grmzm2g037875_t03 0

grmzm2g037923_t03 0

grmzm2g037926_t01 0

grmzm2g037927_t01 0

grmzm2g038003_t01 0

grmzm2g038032_t01 0

grmzm2g038050_t02 0

grmzm2g038066_t02 0

grmzm2g038095_t01 0

grmzm2g038108_t01 0

grmzm2g038126_t01 0

grmzm2g038137_t01 0

grmzm2g038153_t01 0

grmzm2g038158_t01 0

grmzm2g038162_t02 0

grmzm2g038182_t01 0

grmzm2g038183_t01 0.89

grmzm2g038195_t01 0

grmzm2g038217_t01 0

grmzm2g038243_t03 0

grmzm2g038246_t01 0

grmzm2g038279_t01 0

grmzm2g038281_t01 0

grmzm2g038284_t03 0

grmzm2g038301_t01 0

grmzm2g038303_t01 0

grmzm2g038309_t01 0

grmzm2g038313_t01 0

grmzm2g038338_t02 0

grmzm2g038356_t01 0

grmzm2g038365_t01 0

grmzm2g038374_t01 0

grmzm2g038375_t01 0

grmzm2g038384_t01 0

grmzm2g038388_t01 0

grmzm2g038394_t01 0

grmzm2g038401_t01 0

grmzm2g038412_t01 0

grmzm2g038487_t01 0

grmzm2g038494_t01 0

grmzm2g038512_t04 0

grmzm2g038519_t04 0

grmzm2g038532_t01 0

grmzm2g038536_t01 0

grmzm2g038588_t02 0

grmzm2g038598_t06 0

grmzm2g038606_t02 0

grmzm2g038623_t01 0

grmzm2g038636_t01 0

grmzm2g038643_t02 0

grmzm2g038667_t02 1.26

grmzm2g038691_t02 0

grmzm2g038780_t01 0

grmzm2g038783_t01 0

grmzm2g038791_t06 0

grmzm2g038801_t01 0

grmzm2g038820_t01 0

grmzm2g038833_t01 0

grmzm2g038848_t01 0

grmzm2g038882_t01 0.96

grmzm2g038898_t01 -0.95

grmzm2g038922_t02 0

grmzm2g038953_t01 0

grmzm2g038964_t01 0

grmzm2g038988_t01 0

grmzm2g039011_t01 -0.9

grmzm2g039089_t01 0

grmzm2g039106_t01 0

grmzm2g039126_t01 0

grmzm2g039155_t05 0

grmzm2g039173_t01 0

grmzm2g039186_t01 0

grmzm2g039214_t01 0

grmzm2g039238_t02 0

grmzm2g039246_t01 0

grmzm2g039251_t01 0

grmzm2g039254_t01 0

grmzm2g039263_t02 0

grmzm2g039280_t01 0

grmzm2g039312_t01 -1.69

grmzm2g039325_t01 0

grmzm2g039345_t01 0

grmzm2g039365_t03 0

grmzm2g039373_t01 0

grmzm2g039385_t05 1.06

grmzm2g039396_t01 0

grmzm2g039419_t01 0

grmzm2g039445_t03 0

grmzm2g039454_t01 0

grmzm2g039455_t01 0

grmzm2g039505_t01 0

grmzm2g039532_t01 0

grmzm2g039536_t01 0

grmzm2g039542_t01 0

grmzm2g039583_t03 0

grmzm2g039586_t01 0

grmzm2g039588_t01 0

grmzm2g039592_t01 0

grmzm2g039600_t01 0

grmzm2g039618_t02 0

grmzm2g039622_t01 0

grmzm2g039639_t01 -1.45

grmzm2g039650_t03 0

grmzm2g039671_t01 0

grmzm2g039683_t01 0

grmzm2g039696_t03 0

grmzm2g039711_t01 0

grmzm2g039725_t01 0

grmzm2g039746_t01 0

grmzm2g039757_t01 0

grmzm2g039792_t01 0

grmzm2g039799_t01 0

grmzm2g039811_t01 0

grmzm2g039824_t02 0

grmzm2g039826_t01 0

grmzm2g039828_t01 0

grmzm2g039841_t02 0

grmzm2g039886_t01 0

grmzm2g039889_t04 0

grmzm2g039891_t01 0

grmzm2g039895_t01 0

grmzm2g039900_t03 0

grmzm2g039906_t02 0

grmzm2g039919_t03 0

grmzm2g039922_t01 -1.71

grmzm2g039954_t03 0

grmzm2g039961_t01 0

grmzm2g039978_t01 0

grmzm2g039982_t02 0

grmzm2g039983_t01 0

grmzm2g039993_t01 1.02

grmzm2g039996_t01 -1.25

grmzm2g040017_t01 0

grmzm2g040024_t03 0

grmzm2g040030_t01 0

grmzm2g040069_t01 0

grmzm2g040079_t01 0

grmzm2g040090_t02 0

grmzm2g040095_t01 -1.32

grmzm2g040100_t01 0

grmzm2g040131_t01 0

grmzm2g040145_t01 0

grmzm2g040152_t02 0.97

grmzm2g040158_t03 0

grmzm2g040161_t01 0

grmzm2g040164_t01 0

grmzm2g040182_t01 0

grmzm2g040207_t02 0

grmzm2g040209_t02 0

grmzm2g040247_t01 0

grmzm2g040296_t01 0

grmzm2g040300_t01 0

grmzm2g040309_t02 0

grmzm2g040320_t01 0

grmzm2g040349_t01 0

grmzm2g040382_t01 0

grmzm2g040387_t01 0

grmzm2g040389_t02 0

grmzm2g040397_t01 0

grmzm2g040401_t03 0

grmzm2g040424_t01 0

grmzm2g040441_t04 0

grmzm2g040452_t03 0

grmzm2g040467_t01 0

grmzm2g040477_t01 0

grmzm2g040493_t01 0

grmzm2g040511_t01 0

grmzm2g040513_t02 0

grmzm2g040515_t01 0

grmzm2g040559_t01 0

grmzm2g040561_t01 0

grmzm2g040587_t01 0

grmzm2g040613_t02 0

grmzm2g040618_t01 0

grmzm2g040627_t02 0

grmzm2g040634_t03 0

grmzm2g040642_t01 0

grmzm2g040672_t03 0

grmzm2g040692_t01 0

grmzm2g040702_t02 0

grmzm2g040706_t01 0

grmzm2g040720_t01 0

grmzm2g040728_t01 0

grmzm2g040734_t02 0

grmzm2g040736_t01 0

grmzm2g040743_t01 0

grmzm2g040750_t01 0

grmzm2g040762_t01 0

grmzm2g040785_t01 -1.6

grmzm2g040803_t01 0

grmzm2g040828_t01 0

grmzm2g040843_t02 0

grmzm2g040878_t01 0

grmzm2g040887_t02 0

grmzm2g040890_t01 0

grmzm2g040902_t02 0

grmzm2g040920_t02 0

grmzm2g040933_t02 0

grmzm2g040945_t01 0

grmzm2g040947_t01 0

grmzm2g040954_t02 0

grmzm2g040964_t01 0

grmzm2g040968_t01 0

grmzm2g040970_t01 0

grmzm2g040991_t03 0

grmzm2g040995_t01 0

grmzm2g041015_t02 0

grmzm2g041022_t01 0.83

grmzm2g041028_t01 0

grmzm2g041041_t01 0

grmzm2g041048_t01 0

grmzm2g041050_t08 0

grmzm2g041058_t01 0

grmzm2g041060_t01 0

grmzm2g041061_t02 0

grmzm2g041068_t01 1.42

grmzm2g041127_t01 -1.19

grmzm2g041159_t02 0

grmzm2g041175_t02 0

grmzm2g041181_t01 0

grmzm2g041223_t01 0

grmzm2g041231_t01 0

grmzm2g041238_t01 0

grmzm2g041258_t01 0

grmzm2g041269_t01 0

grmzm2g041275_t01 0

grmzm2g041312_t01 0

grmzm2g041314_t01 0

grmzm2g041328_t02 0

grmzm2g041381_t02 0

grmzm2g041394_t01 0

grmzm2g041415_t01 0

grmzm2g041462_t01 0

grmzm2g041463_t01 0

grmzm2g041472_t04 0

grmzm2g041506_t01 0

grmzm2g041518_t01 0

grmzm2g041549_t01 0

grmzm2g041561_t01 0

grmzm2g041633_t02 0

grmzm2g041697_t01 0

grmzm2g041699_t01 0

grmzm2g041701_t02 1.37

grmzm2g041714_t02 0

grmzm2g041732_t01 0

grmzm2g041770_t01 0

grmzm2g041797_t01 0

grmzm2g041799_t01 0

grmzm2g041818_t01 0

grmzm2g041822_t02 0

grmzm2g041831_t04 0

grmzm2g041842_t01 0

grmzm2g041847_t01 0

grmzm2g041866_t01 0

grmzm2g041876_t01 0

grmzm2g041881_t01 0

grmzm2g041885_t02 0

grmzm2g041955_t01 0

grmzm2g041959_t01 0

grmzm2g041961_t01 0

grmzm2g041980_t01 0

grmzm2g041989_t01 0

grmzm2g041991_t01 0

grmzm2g041994_t01 0

grmzm2g042006_t02 0

grmzm2g042008_t01 0

grmzm2g042027_t01 0

grmzm2g042032_t03 0

grmzm2g042036_t01 1.43

grmzm2g042040_t02 0

grmzm2g042046_t01 0

grmzm2g042047_t02 0

grmzm2g042055_t01 0

grmzm2g042061_t01 0

grmzm2g042074_t02 0

grmzm2g042078_t03 0

grmzm2g042080_t03 0

grmzm2g042089_t02 0.96

grmzm2g042099_t01 0

grmzm2g042118_t01 0

grmzm2g042127_t01 0

grmzm2g042136_t02 0

grmzm2g042143_t01 0

grmzm2g042146_t01 0

grmzm2g042164_t01 0

grmzm2g042171_t03 0

grmzm2g042179_t01 0

grmzm2g042181_t01 0

grmzm2g042195_t02 0

grmzm2g042198_t01 0

grmzm2g042208_t03 0

grmzm2g042231_t01 0

grmzm2g042245_t01 0

grmzm2g042253_t01 0

grmzm2g042269_t01 0

grmzm2g042278_t01 0

grmzm2g042292_t01 0.91

grmzm2g042295_t01 0

grmzm2g042313_t01 0

grmzm2g042343_t01 0

grmzm2g042347_t01 0

grmzm2g042356_t01 0

grmzm2g042371_t01 0

grmzm2g042380_t01 0

grmzm2g042398_t02 0

grmzm2g042443_t01 0

grmzm2g042488_t02 0

grmzm2g042492_t01 0

grmzm2g042502_t01 0

grmzm2g042516_t02 0

grmzm2g042541_t02 0

grmzm2g042552_t01 0

grmzm2g042572_t01 0

grmzm2g042582_t01 0

grmzm2g042592_t01 0

grmzm2g042598_t01 0

grmzm2g042599_t04 0

grmzm2g042602_t01 0

grmzm2g042604_t01 0

grmzm2g042607_t01 0

grmzm2g042612_t01 0

grmzm2g042615_t03 0

grmzm2g042623_t01 0

grmzm2g042636_t03 0

grmzm2g042639_t08 0

grmzm2g042660_t01 0

grmzm2g042664_t03 0

grmzm2g042666_t01 0

grmzm2g042683_t01 0

grmzm2g042698_t04 0

grmzm2g042704_t05 0

grmzm2g042712_t01 0

grmzm2g042758_t01 2.08

grmzm2g042782_t01 0

grmzm2g042789_t01 -0.95

grmzm2g042806_t01 0

grmzm2g042807_t01 0

grmzm2g042818_t01 0

grmzm2g042855_t01 0

grmzm2g042865_t01 -1.95

grmzm2g042879_t01 0

grmzm2g042881_t03 0

grmzm2g042889_t01 0

grmzm2g042893_t02 0

grmzm2g042895_t01 0

grmzm2g042897_t03 0

grmzm2g042981_t01 0

grmzm2g042992_t01 0

grmzm2g043030_t01 0

grmzm2g043049_t01 0

grmzm2g043056_t01 0

grmzm2g043075_t01 0

grmzm2g043109_t01 0

grmzm2g043117_t01 0

grmzm2g043119_t01 0

grmzm2g043127_t01 0

grmzm2g043147_t01 0

grmzm2g043150_t03 1.24

grmzm2g043162_t01 0

grmzm2g043174_t01 0

grmzm2g043182_t01 0

grmzm2g043193_t02 0

grmzm2g043212_t03 0

grmzm2g043226_t01 0

grmzm2g043240_t01 0

grmzm2g043242_t01 0

grmzm2g043275_t01 0

grmzm2g043277_t02 0

grmzm2g043279_t02 0

grmzm2g043291_t06 0

grmzm2g043295_t01 0

grmzm2g043300_t01 0

grmzm2g043310_t01 0

grmzm2g043331_t01 0

grmzm2g043336_t01 -1.53

grmzm2g043338_t01 0

grmzm2g043350_t01 0

grmzm2g043383_t02 0

grmzm2g043414_t01 0.98

grmzm2g043435_t02 0

grmzm2g043453_t02 0

grmzm2g043456_t01 0

grmzm2g043464_t01 0

grmzm2g043470_t02 0

grmzm2g043484_t01 0

grmzm2g043489_t01 0

grmzm2g043493_t04 0

grmzm2g043500_t01 0

grmzm2g043501_t01 0

grmzm2g043509_t01 0

grmzm2g043530_t01 0

grmzm2g043584_t01 0

grmzm2g043602_t03 0

grmzm2g043632_t03 0

grmzm2g043682_t02 0

grmzm2g043724_t01 0

grmzm2g043749_t02 0

grmzm2g043764_t01 0

grmzm2g043773_t01 0

grmzm2g043776_t01 0.94

grmzm2g043782_t01 0

grmzm2g043783_t01 0

grmzm2g043799_t01 0

grmzm2g043819_t02 0

grmzm2g043822_t03 0

grmzm2g043843_t01 0

grmzm2g043857_t01 0

grmzm2g043862_t01 0

grmzm2g043879_t01 0

grmzm2g043903_t01 0

grmzm2g043921_t01 0

grmzm2g043932_t01 0

grmzm2g043948_t01 0

grmzm2g043955_t01 0

grmzm2g043980_t05 0

grmzm2g043983_t03 0

grmzm2g043995_t02 0

grmzm2g044004_t01 0

grmzm2g044011_t01 0

grmzm2g044023_t02 0

grmzm2g044027_t01 0

grmzm2g044038_t01 0

grmzm2g044044_t02 0.84

grmzm2g044055_t01 0

grmzm2g044060_t02 0

grmzm2g044062_t01 0

grmzm2g044070_t01 0

grmzm2g044074_t01 0

grmzm2g044085_t01 0

grmzm2g044096_t01 0

grmzm2g044107_t01 0

grmzm2g044116_t01 0

grmzm2g044126_t01 0.96

grmzm2g044128_t01 0

grmzm2g044132_t02 0

grmzm2g044137_t01 0

grmzm2g044143_t01 0

grmzm2g044174_t01 0

grmzm2g044175_t01 0

grmzm2g044180_t03 0

grmzm2g044237_t01 0

grmzm2g044247_t01 0

grmzm2g044265_t01 0

grmzm2g044281_t01 0

grmzm2g044301_t01 0

grmzm2g044337_t02 0

grmzm2g044343_t01 0

grmzm2g044348_t03 0

grmzm2g044354_t04 0

grmzm2g044359_t02 -1.54

grmzm2g044382_t01 0

grmzm2g044388_t02 0

grmzm2g044398_t09 0

grmzm2g044422_t01 0

grmzm2g044423_t01 0

grmzm2g044442_t01 0

grmzm2g044448_t01 0

grmzm2g044457_t01 0

grmzm2g044460_t02 0

grmzm2g044495_t01 0

grmzm2g044498_t01 0

grmzm2g044501_t01 0

grmzm2g044512_t01 0

grmzm2g044527_t01 0

grmzm2g044531_t01 0

grmzm2g044552_t01 0

grmzm2g044569_t05 0

grmzm2g044581_t01 -0.82

grmzm2g044584_t02 0

grmzm2g044591_t01 -0.84

grmzm2g044629_t01 0

grmzm2g044681_t01 0

grmzm2g044684_t01 0

grmzm2g044697_t03 0

grmzm2g044724_t01 0

grmzm2g044733_t01 0

grmzm2g044744_t01 0

grmzm2g044752_t02 0

grmzm2g044762_t03 0

grmzm2g044771_t01 0

grmzm2g044775_t01 0

grmzm2g044800_t03 0

grmzm2g044824_t01 0

grmzm2g044832_t01 0

grmzm2g044862_t01 0

grmzm2g044866_t01 0

grmzm2g044875_t01 0

grmzm2g044900_t01 0

grmzm2g044902_t01 -1.37

grmzm2g044908_t02 0

grmzm2g044915_t01 0

grmzm2g044943_t01 0

grmzm2g044947_t04 0

grmzm2g044963_t01 0

grmzm2g044968_t01 0

grmzm2g044997_t03 0

grmzm2g045005_t04 0.91

grmzm2g045019_t01 0

grmzm2g045027_t01 0

grmzm2g045049_t01 0

grmzm2g045057_t02 0

grmzm2g045070_t02 0

grmzm2g045084_t01 0

grmzm2g045088_t01 0

grmzm2g045090_t01 0

grmzm2g045102_t01 0

grmzm2g045135_t01 0

grmzm2g045171_t01 -0.96

grmzm2g045178_t05 0

grmzm2g045183_t05 0

grmzm2g045185_t01 0

grmzm2g045188_t01 0

grmzm2g045192_t02 0

grmzm2g045215_t01 -1.23

grmzm2g045236_t01 0

grmzm2g045241_t02 0

grmzm2g045259_t01 0

grmzm2g045270_t02 0

grmzm2g045275_t01 0

grmzm2g045280_t01 0

grmzm2g045286_t01 0

grmzm2g045287_t01 0

grmzm2g045294_t02 0

grmzm2g045303_t01 0

grmzm2g045314_t02 0

grmzm2g045330_t01 0

grmzm2g045331_t05 0

grmzm2g045366_t01 0

grmzm2g045398_t01 0

grmzm2g045404_t02 0

grmzm2g045421_t01 0

grmzm2g045428_t01 0

grmzm2g045430_t01 0

grmzm2g045433_t01 0

grmzm2g045435_t01 0

grmzm2g045467_t01 0

grmzm2g045503_t01 0

grmzm2g045507_t01 0

grmzm2g045544_t01 0

grmzm2g045581_t01 0

grmzm2g045596_t03 -0.9

grmzm2g045638_t01 0

grmzm2g045664_t01 0

grmzm2g045668_t01 0

grmzm2g045675_t02 0

grmzm2g045688_t04 0

grmzm2g045690_t01 0

grmzm2g045704_t01 0

grmzm2g045706_t01 0

grmzm2g045714_t01 1.18

grmzm2g045716_t01 0

grmzm2g045732_t02 0

grmzm2g045748_t01 0

grmzm2g045753_t08 0

grmzm2g045779_t01 0

grmzm2g045820_t01 0

grmzm2g045849_t01 0

grmzm2g045854_t01 0.92

grmzm2g045921_t01 0

grmzm2g045970_t01 0

grmzm2g045976_t01 0

grmzm2g045987_t02 0

grmzm2g046005_t02 0

grmzm2g046011_t01 0

grmzm2g046021_t03 0

grmzm2g046024_t01 0

grmzm2g046025_t01 0.89

grmzm2g046055_t02 0

grmzm2g046061_t01 0

grmzm2g046065_t01 0

grmzm2g046070_t02 0

grmzm2g046088_t03 0

grmzm2g046098_t05 0

grmzm2g046101_t02 0

grmzm2g046143_t02 0

grmzm2g046186_t01 0

grmzm2g046191_t02 0

grmzm2g046231_t01 0

grmzm2g046244_t01 0

grmzm2g046274_t01 0

grmzm2g046284_t01 0

grmzm2g046288_t02 0

grmzm2g046290_t01 0

grmzm2g046293_t01 0

grmzm2g046297_t02 0

grmzm2g046317_t01 0

grmzm2g046348_t01 0

grmzm2g046355_t02 -1.15

grmzm2g046382_t01 0

grmzm2g046402_t01 0

grmzm2g046407_t01 0

grmzm2g046430_t02 0

grmzm2g046459_t01 0

grmzm2g046472_t01 0

grmzm2g046474_t03 0

grmzm2g046498_t01 0

grmzm2g046529_t03 0

grmzm2g046537_t01 0

grmzm2g046558_t01 0

grmzm2g046574_t01 1.67

grmzm2g046576_t01 0

grmzm2g046583_t01 0.81

grmzm2g046587_t03 0

grmzm2g046601_t02 0

grmzm2g046615_t01 0

grmzm2g046618_t01 0

grmzm2g046658_t01 0

grmzm2g046681_t03 0

grmzm2g046729_t02 0

grmzm2g046743_t02 0

grmzm2g046748_t02 0

grmzm2g046750_t01 0

grmzm2g046782_t01 0

grmzm2g046784_t01 0

grmzm2g046804_t05 0

grmzm2g046816_t04 0

grmzm2g046824_t04 0

grmzm2g046838_t02 0

grmzm2g046841_t01 0

grmzm2g046846_t01 0

grmzm2g046849_t01 0

grmzm2g046861_t01 0

grmzm2g046870_t01 0

grmzm2g046900_t04 0

grmzm2g046909_t01 0

grmzm2g046911_t01 0

grmzm2g046916_t01 0

grmzm2g046919_t01 0

grmzm2g046924_t01 0

grmzm2g046932_t01 0

grmzm2g046952_t02 0

grmzm2g046966_t01 0

grmzm2g046968_t02 0

grmzm2g047002_t02 0

grmzm2g047018_t02 0

grmzm2g047019_t01 0

grmzm2g047028_t01 1.16

grmzm2g047038_t01 0

grmzm2g047042_t03 0

grmzm2g047055_t03 0

grmzm2g047093_t02 0

grmzm2g047095_t04 0

grmzm2g047097_t01 0

grmzm2g047124_t01 0

grmzm2g047129_t01 0

grmzm2g047139_t01 0

grmzm2g047143_t01 0

grmzm2g047152_t01 0

grmzm2g047161_t01 0

grmzm2g047167_t01 0

grmzm2g047178_t01 0

grmzm2g047187_t01 0

grmzm2g047204_t02 0

grmzm2g047214_t01 0

grmzm2g047219_t01 0

grmzm2g047223_t01 0

grmzm2g047238_t01 0

grmzm2g047255_t01 -0.99

grmzm2g047274_t01 0

grmzm2g047298_t01 0

grmzm2g047299_t02 0

grmzm2g047310_t04 0

grmzm2g047316_t02 0

grmzm2g047319_t01 0

grmzm2g047321_t01 0

grmzm2g047347_t02 0

grmzm2g047365_t01 0

grmzm2g047370_t02 0

grmzm2g047372_t01 0

grmzm2g047376_t03 0

grmzm2g047377_t01 0

grmzm2g047384_t04 -1.89

grmzm2g047404_t01 0

grmzm2g047412_t02 0

grmzm2g047414_t01 0

grmzm2g047419_t04 0

grmzm2g047456_t01 -0.89

grmzm2g047457_t01 0

grmzm2g047474_t02 0

grmzm2g047476_t01 0

grmzm2g047486_t04 0

grmzm2g047509_t01 0

grmzm2g047512_t01 0

grmzm2g047513_t01 0

grmzm2g047564_t05 0

grmzm2g047577_t01 0

grmzm2g047588_t01 0

grmzm2g047590_t02 0

grmzm2g047592_t01 0

grmzm2g047607_t01 0

grmzm2g047626_t01 0

grmzm2g047695_t02 0

grmzm2g047705_t05 0

grmzm2g047720_t01 0

grmzm2g047727_t01 0

grmzm2g047732_t01 0

grmzm2g047774_t01 0

grmzm2g047777_t01 0

grmzm2g047781_t01 0

grmzm2g047813_t01 0

grmzm2g047815_t05 0

grmzm2g047855_t01 0

grmzm2g047860_t02 1.32

grmzm2g047867_t01 0

grmzm2g047894_t03 0

grmzm2g047949_t01 0

grmzm2g047954_t02 0

grmzm2g047961_t01 0

grmzm2g047968_t04 0

grmzm2g047971_t01 0

grmzm2g047995_t02 0

grmzm2g048006_t01 0

grmzm2g048008_t02 0

grmzm2g048013_t01 0

grmzm2g048022_t01 0

grmzm2g048045_t01 0

grmzm2g048067_t02 0

grmzm2g048085_t04 0

grmzm2g048106_t01 0

grmzm2g048117_t01 0

grmzm2g048121_t02 0

grmzm2g048129_t02 0

grmzm2g048136_t02 0

grmzm2g048140_t01 0

grmzm2g048147_t01 0

grmzm2g048175_t01 0

grmzm2g048192_t02 -1.34

grmzm2g048200_t02 -0.93

grmzm2g048205_t02 0.83

grmzm2g048210_t01 0

grmzm2g048225_t01 0

grmzm2g048243_t02 0

grmzm2g048257_t02 0

grmzm2g048276_t02 0

grmzm2g048277_t04 0

grmzm2g048281_t01 0

grmzm2g048294_t01 0

grmzm2g048305_t01 0

grmzm2g048313_t01 0

grmzm2g048324_t01 0

grmzm2g048363_t01 0

grmzm2g048371_t02 0

grmzm2g048392_t01 0

grmzm2g048411_t01 0

grmzm2g048430_t01 0

grmzm2g048434_t08 0

grmzm2g048435_t01 0

grmzm2g048450_t01 0

grmzm2g048455_t02 0

grmzm2g048472_t01 0

grmzm2g048474_t01 0

grmzm2g048475_t01 0

grmzm2g048482_t01 0

grmzm2g048494_t03 0

grmzm2g048497_t01 0

grmzm2g048537_t01 0

grmzm2g048549_t02 -0.83

grmzm2g048559_t01 0

grmzm2g048561_t01 0

grmzm2g048582_t01 1.01

grmzm2g048591_t01 0

grmzm2g048596_t02 0

grmzm2g048611_t01 0

grmzm2g048616_t01 0

grmzm2g048644_t01 0

grmzm2g048661_t01 0

grmzm2g048665_t01 0

grmzm2g048672_t01 0

grmzm2g048680_t01 0

grmzm2g048703_t01 0

grmzm2g048733_t01 0

grmzm2g048740_t02 0

grmzm2g048806_t01 0

grmzm2g048819_t02 0

grmzm2g048821_t01 0

grmzm2g048843_t03 0

grmzm2g048846_t01 0

grmzm2g048851_t01 0

grmzm2g048883_t01 0

grmzm2g048904_t02 0

grmzm2g048907_t01 0

grmzm2g048912_t03 0

grmzm2g048928_t01 0

grmzm2g049013_t01 0

grmzm2g049021_t03 0

grmzm2g049031_t01 0

grmzm2g049039_t09 0

grmzm2g049041_t02 0

grmzm2g049055_t02 0

grmzm2g049076_t02 0

grmzm2g049077_t02 0

grmzm2g049088_t01 0

grmzm2g049091_t03 0

grmzm2g049117_t03 0

grmzm2g049141_t01 0

grmzm2g049155_t02 0

grmzm2g049159_t01 0

grmzm2g049177_t01 0

grmzm2g049185_t02 0

grmzm2g049190_t02 0

grmzm2g049201_t02 0

grmzm2g049229_t01 0

grmzm2g049240_t02 0

grmzm2g049269_t02 0

grmzm2g049288_t01 0

grmzm2g049296_t01 0

grmzm2g049322_t02 0

grmzm2g049329_t01 0

grmzm2g049340_t01 0

grmzm2g049342_t01 0

grmzm2g049346_t01 0

grmzm2g049349_t01 0

grmzm2g049351_t01 0

grmzm2g049364_t01 0

grmzm2g049373_t02 0

grmzm2g049378_t01 0

grmzm2g049382_t03 0

grmzm2g049401_t01 0

grmzm2g049416_t01 0

grmzm2g049422_t01 0

grmzm2g049429_t02 0

grmzm2g049484_t01 0

grmzm2g049487_t01 0

grmzm2g049495_t01 0

grmzm2g049510_t01 0

grmzm2g049525_t01 0

grmzm2g049536_t01 0

grmzm2g049538_t01 0

grmzm2g049547_t01 0

grmzm2g049549_t02 0

grmzm2g049568_t01 0

grmzm2g049588_t03 0

grmzm2g049609_t01 0

grmzm2g049636_t01 0

grmzm2g049641_t02 0

grmzm2g049672_t01 0

grmzm2g049675_t01 0

grmzm2g049687_t01 0

grmzm2g049693_t01 0

grmzm2g049695_t01 -1.56

grmzm2g049759_t01 0

grmzm2g049766_t01 0

grmzm2g049790_t03 0

grmzm2g049798_t01 0

grmzm2g049811_t01 0

grmzm2g049823_t02 0

grmzm2g049839_t01 0

grmzm2g049866_t01 0

grmzm2g049877_t01 0

grmzm2g049888_t01 0

grmzm2g049895_t02 0

grmzm2g049902_t01 0

grmzm2g049912_t01 0

grmzm2g049915_t01 0

grmzm2g049954_t01 0

grmzm2g050072_t01 0

grmzm2g050076_t01 0

grmzm2g050077_t01 0

grmzm2g050089_t03 0

grmzm2g050108_t01 0

grmzm2g050118_t01 0

grmzm2g050159_t01 0

grmzm2g050166_t01 0

grmzm2g050174_t02 0

grmzm2g050177_t01 -2.35

grmzm2g050186_t01 0

grmzm2g050193_t01 0

grmzm2g050216_t01 0

grmzm2g050218_t01 0

grmzm2g050270_t01 0

grmzm2g050305_t01 0

grmzm2g050309_t01 0

grmzm2g050325_t02 0

grmzm2g050329_t01 0.86

grmzm2g050333_t02 0

grmzm2g050371_t02 0

grmzm2g050375_t02 0

grmzm2g050391_t01 0

grmzm2g050394_t01 0

grmzm2g050412_t03 0

grmzm2g050435_t02 0

grmzm2g050450_t01 0

grmzm2g050460_t01 0

grmzm2g050467_t01 0

grmzm2g050484_t01 0

grmzm2g050485_t01 0

grmzm2g050501_t01 0

grmzm2g050512_t01 0

grmzm2g050514_t03 0

grmzm2g050548_t01 -2.35

grmzm2g050550_t01 0

grmzm2g050553_t03 0

grmzm2g050570_t01 0

grmzm2g050583_t01 0

grmzm2g050590_t01 0

grmzm2g050596_t02 0

grmzm2g050625_t01 0

grmzm2g050628_t01 0

grmzm2g050641_t01 0

grmzm2g050647_t01 0

grmzm2g050649_t04 0

grmzm2g050658_t03 0

grmzm2g050693_t04 0

grmzm2g050697_t01 0

grmzm2g050701_t02 0

grmzm2g050705_t02 0

grmzm2g050714_t01 0

grmzm2g050730_t02 0

grmzm2g050734_t01 0

grmzm2g050765_t01 0

grmzm2g050774_t01 0

grmzm2g050803_t01 0

grmzm2g050810_t01 0

grmzm2g050833_t01 0

grmzm2g050842_t02 0

grmzm2g050845_t01 0

grmzm2g050861_t01 0

grmzm2g050885_t01 0

grmzm2g050890_t01 0

grmzm2g050912_t01 0

grmzm2g050914_t01 0

grmzm2g050915_t02 0

grmzm2g050925_t01 0

grmzm2g050933_t04 0

grmzm2g050939_t07 0

grmzm2g050946_t01 0

grmzm2g050961_t03 0

grmzm2g050971_t02 0

grmzm2g050982_t01 0

grmzm2g050984_t02 0

grmzm2g051004_t01 0

grmzm2g051005_t01 0

grmzm2g051043_t01 0

grmzm2g051050_t01 0

grmzm2g051059_t01 0

grmzm2g051090_t01 0

grmzm2g051101_t01 0

grmzm2g051103_t01 0

grmzm2g051129_t01 0

grmzm2g051141_t04 0

grmzm2g051151_t01 0

grmzm2g051174_t01 0

grmzm2g051179_t01 0

grmzm2g051185_t01 -1.01

grmzm2g051208_t01 -1.39

grmzm2g051219_t01 0

grmzm2g051228_t03 0

grmzm2g051247_t03 0

grmzm2g051256_t01 -0.84

grmzm2g051262_t02 0

grmzm2g051270_t01 0

grmzm2g051276_t01 0

grmzm2g051288_t01 0

grmzm2g051355_t03 0

grmzm2g051367_t01 0

grmzm2g051403_t02 0

grmzm2g051426_t02 0

grmzm2g051458_t01 0

grmzm2g051502_t01 1.03

grmzm2g051534_t01 0

grmzm2g051541_t01 0

grmzm2g051545_t02 0

grmzm2g051577_t01 0

grmzm2g051613_t03 0

grmzm2g051619_t01 -1.76

grmzm2g051622_t01 0

grmzm2g051626_t03 0

grmzm2g051630_t02 0

grmzm2g051637_t01 0

grmzm2g051677_t05 0

grmzm2g051683_t01 -1.79

grmzm2g051697_t01 0

grmzm2g051724_t01 0

grmzm2g051750_t01 0

grmzm2g051752_t01 0

grmzm2g051753_t03 0

grmzm2g051764_t03 0

grmzm2g051767_t04 0

grmzm2g051771_t01 0

grmzm2g051782_t02 0

grmzm2g051785_t01 0

grmzm2g051790_t02 0

grmzm2g051792_t01 0

grmzm2g051793_t01 0

grmzm2g051806_t01 0

grmzm2g051808_t01 0

grmzm2g051842_t01 0

grmzm2g051848_t01 0

grmzm2g051866_t01 0

grmzm2g051879_t06 0

grmzm2g051890_t01 0

grmzm2g051902_t01 0

grmzm2g051917_t01 0

grmzm2g051932_t01 0

grmzm2g051943_t01 0

grmzm2g051949_t03 0

grmzm2g051952_t05 0

grmzm2g051955_t01 0

grmzm2g051974_t01 0

grmzm2g051984_t02 0

grmzm2g052011_t02 0

grmzm2g052034_t02 0

grmzm2g052036_t01 0

grmzm2g052078_t01 0

grmzm2g052088_t03 0

grmzm2g052100_t01 0

grmzm2g052129_t02 0

grmzm2g052142_t01 0

grmzm2g052148_t01 0

grmzm2g052149_t01 0

grmzm2g052166_t01 0

grmzm2g052178_t02 0

grmzm2g052200_t03 0

grmzm2g052266_t01 0

grmzm2g052285_t02 0

grmzm2g052328_t01 0

grmzm2g052336_t02 0

grmzm2g052339_t01 0

grmzm2g052365_t01 0.96

grmzm2g052373_t01 0

grmzm2g052389_t02 0

grmzm2g052416_t01 0

grmzm2g052418_t01 0

grmzm2g052422_t01 -1.09

grmzm2g052434_t01 0

grmzm2g052435_t01 0

grmzm2g052471_t03 0

grmzm2g052474_t01 0

grmzm2g052479_t01 0

grmzm2g052483_t03 0

grmzm2g052507_t01 0

grmzm2g052509_t01 0

grmzm2g052515_t01 1.36

grmzm2g052544_t01 0

grmzm2g052546_t04 0

grmzm2g052562_t03 0

grmzm2g052569_t01 0

grmzm2g052581_t01 0

grmzm2g052586_t03 0

grmzm2g052610_t02 0

grmzm2g052616_t01 0

grmzm2g052630_t01 0

grmzm2g052650_t01 -0.93

grmzm2g052654_t01 0

grmzm2g052658_t01 0

grmzm2g052666_t01 0

grmzm2g052667_t02 0

grmzm2g052671_t01 0

grmzm2g052685_t01 0

grmzm2g052690_t01 0

grmzm2g052699_t01 0

grmzm2g052720_t01 0

grmzm2g052740_t01 0

grmzm2g052750_t01 0

grmzm2g052812_t01 0

grmzm2g052817_t01 0

grmzm2g052821_t01 0

grmzm2g052823_t01 0

grmzm2g052844_t02 0

grmzm2g052855_t01 0

grmzm2g052869_t01 0

grmzm2g052875_t01 0

grmzm2g052893_t01 0

grmzm2g052908_t01 0

grmzm2g052926_t01 0

grmzm2g052935_t02 0

grmzm2g052989_t01 0

grmzm2g053008_t03 0

grmzm2g053019_t01 0

grmzm2g053023_t02 0

grmzm2g053027_t02 0

grmzm2g053047_t02 0

grmzm2g053066_t01 0

grmzm2g053079_t01 0

grmzm2g053083_t01 0

grmzm2g053140_t01 0

grmzm2g053150_t01 0

grmzm2g053170_t01 0

grmzm2g053196_t01 0

grmzm2g053206_t01 0

grmzm2g053210_t01 0

grmzm2g053236_t01 0

grmzm2g053239_t01 0

grmzm2g053261_t03 0

grmzm2g053273_t01 0

grmzm2g053298_t02 0

grmzm2g053299_t01 0

grmzm2g053303_t01 0

grmzm2g053315_t01 -0.87

grmzm2g053319_t01 0

grmzm2g053322_t01 0

grmzm2g053384_t01 0

grmzm2g053396_t01 0

grmzm2g053397_t03 0

grmzm2g053434_t02 0

grmzm2g053500_t01 0

grmzm2g053514_t01 0

grmzm2g053531_t01 0

grmzm2g053554_t02 0

grmzm2g053557_t03 0

grmzm2g053574_t01 0

grmzm2g053600_t02 0

grmzm2g053610_t02 0

grmzm2g053652_t01 0

grmzm2g053669_t01 0

grmzm2g053676_t01 0

grmzm2g053707_t01 0

grmzm2g053720_t02 -2.02

grmzm2g053722_t01 0

grmzm2g053726_t01 0

grmzm2g053766_t01 0

grmzm2g053767_t03 0

grmzm2g053779_t01 0

grmzm2g053781_t01 0

grmzm2g053790_t01 0

grmzm2g053801_t01 0

grmzm2g053803_t03 0

grmzm2g053831_t01 0

grmzm2g053869_t01 0

grmzm2g053898_t02 0

grmzm2g053908_t01 0

grmzm2g053909_t01 0

grmzm2g053916_t05 0

grmzm2g053921_t01 0

grmzm2g053925_t01 0

grmzm2g053934_t03 0

grmzm2g053946_t01 0

grmzm2g053953_t02 0

grmzm2g053977_t01 0

grmzm2g053987_t03 0

grmzm2g053999_t01 0

grmzm2g054012_t01 0

grmzm2g054013_t01 0

grmzm2g054020_t02 0

grmzm2g054028_t03 0

grmzm2g054040_t01 0

grmzm2g054050_t01 -1.04

grmzm2g054056_t01 0

grmzm2g054065_t01 0

grmzm2g054070_t01 0

grmzm2g054076_t01 0

grmzm2g054109_t02 0

grmzm2g054115_t02 -1.08

grmzm2g054123_t01 0

grmzm2g054136_t08 0

grmzm2g054145_t02 0

grmzm2g054149_t02 0

grmzm2g054152_t01 0

grmzm2g054162_t03 0

grmzm2g054201_t02 0

grmzm2g054210_t01 0

grmzm2g054221_t06 0

grmzm2g054225_t01 0

grmzm2g054227_t02 0

grmzm2g054233_t01 0

grmzm2g054247_t01 0

grmzm2g054250_t02 0

grmzm2g054252_t03 0

grmzm2g054267_t01 0

grmzm2g054277_t01 0

grmzm2g054300_t01 0

grmzm2g054332_t01 0

grmzm2g054350_t01 0

grmzm2g054354_t02 0

grmzm2g054378_t04 0

grmzm2g054380_t05 0

grmzm2g054383_t01 0

grmzm2g054387_t01 0

grmzm2g054393_t03 0

grmzm2g054405_t02 0

grmzm2g054418_t01 0

grmzm2g054465_t01 0

grmzm2g054468_t02 0

grmzm2g054470_t01 0

grmzm2g054481_t01 0

grmzm2g054509_t01 0

grmzm2g054537_t01 0

grmzm2g054559_t03 0

grmzm2g054591_t01 0

grmzm2g054615_t01 0

grmzm2g054621_t03 0

grmzm2g054678_t02 0

grmzm2g054703_t01 0

grmzm2g054706_t01 0

grmzm2g054715_t06 0

grmzm2g054765_t01 0

grmzm2g054807_t01 0

grmzm2g054821_t01 0

grmzm2g054827_t01 0

grmzm2g054830_t02 0

grmzm2g054860_t01 0

grmzm2g054870_t01 0

grmzm2g054896_t01 1.04

grmzm2g054900_t01 0

grmzm2g054905_t01 0

grmzm2g054916_t01 0

grmzm2g054936_t01 0

grmzm2g055020_t01 0

grmzm2g055025_t01 0

grmzm2g055089_t04 0

grmzm2g055107_t01 0

grmzm2g055116_t01 0

grmzm2g055135_t06 0

grmzm2g055141_t01 0

grmzm2g055172_t01 0

grmzm2g055180_t01 0

grmzm2g055204_t01 0

grmzm2g055216_t04 0

grmzm2g055217_t01 0

grmzm2g055219_t01 0

grmzm2g055243_t01 0

grmzm2g055251_t01 0

grmzm2g055255_t01 0

grmzm2g055273_t01 0.87

grmzm2g055276_t03 0

grmzm2g055309_t02 0

grmzm2g055318_t04 0

grmzm2g055320_t01 0

grmzm2g055331_t08 0

grmzm2g055334_t01 0

grmzm2g055413_t01 0

grmzm2g055437_t01 0

grmzm2g055450_t01 0

grmzm2g055452_t01 0

grmzm2g055458_t02 0

grmzm2g055462_t01 0

grmzm2g055467_t01 2.59

grmzm2g055469_t01 0

grmzm2g055489_t04 0

grmzm2g055507_t01 0

grmzm2g055520_t01 0

grmzm2g055527_t01 0

grmzm2g055538_t03 0

grmzm2g055557_t01 0

grmzm2g055575_t01 0

grmzm2g055585_t02 0

grmzm2g055607_t01 0

grmzm2g055619_t01 0

grmzm2g055643_t01 0

grmzm2g055657_t02 0

grmzm2g055678_t04 0

grmzm2g055682_t01 0

grmzm2g055684_t01 0

grmzm2g055698_t02 0

grmzm2g055699_t01 0

grmzm2g055704_t02 -1.37

grmzm2g055705_t01 0

grmzm2g055717_t03 0

grmzm2g055724_t01 0

grmzm2g055752_t01 0

grmzm2g055754_t01 0

grmzm2g055756_t01 0

grmzm2g055785_t01 0

grmzm2g055802_t01 0

grmzm2g055807_t02 0

grmzm2g055809_t03 0

grmzm2g055826_t01 0

grmzm2g055834_t01 0

grmzm2g055844_t01 0

grmzm2g055857_t02 0

grmzm2g055880_t01 0

grmzm2g055883_t03 0

grmzm2g055898_t01 0

grmzm2g055899_t06 0

grmzm2g055905_t02 0

grmzm2g055917_t03 0

grmzm2g055936_t01 0

grmzm2g055957_t03 0

grmzm2g055960_t01 0

grmzm2g055970_t02 0

grmzm2g055973_t01 0

grmzm2g055982_t01 0

grmzm2g055992_t01 0

grmzm2g055993_t02 0

grmzm2g055999_t01 0

grmzm2g056014_t01 0

grmzm2g056036_t04 0

grmzm2g056038_t08 0

grmzm2g056039_t01 0

grmzm2g056056_t01 0

grmzm2g056068_t04 0

grmzm2g056075_t01 0

grmzm2g056079_t03 0

grmzm2g056081_t02 0

grmzm2g056093_t01 0

grmzm2g056116_t01 0

grmzm2g056120_t01 0

grmzm2g056143_t01 0

grmzm2g056145_t01 0

grmzm2g056166_t01 0

grmzm2g056236_t01 0

grmzm2g056247_t03 0.81

grmzm2g056252_t02 0

grmzm2g056270_t03 0

grmzm2g056335_t01 0

grmzm2g056350_t03 0

grmzm2g056357_t03 0

grmzm2g056393_t01 0

grmzm2g056400_t01 0

grmzm2g056407_t01 -3.74

grmzm2g056414_t02 0

grmzm2g056419_t01 0

grmzm2g056424_t01 0

grmzm2g056431_t03 0

grmzm2g056442_t01 0

grmzm2g056462_t01 0

grmzm2g056495_t02 0

grmzm2g056501_t05 0

grmzm2g056505_t02 0

grmzm2g056513_t02 0

grmzm2g056547_t01 0

grmzm2g056564_t01 0

grmzm2g056569_t01 0

grmzm2g056572_t05 0

grmzm2g056573_t01 0

grmzm2g056598_t01 0

grmzm2g056600_t01 0

grmzm2g056608_t01 0

grmzm2g056610_t01 0

grmzm2g056612_t01 0

grmzm2g056632_t01 0

grmzm2g056638_t01 1.94

grmzm2g056686_t05 0

grmzm2g056695_t01 0

grmzm2g056702_t02 0

grmzm2g056729_t01 0

grmzm2g056732_t01 0

grmzm2g056756_t01 0

grmzm2g056762_t03 0

grmzm2g056766_t01 0

grmzm2g056772_t01 -1.1

grmzm2g056773_t01 0

grmzm2g056786_t01 0

grmzm2g056815_t01 0

grmzm2g056849_t01 0

grmzm2g056870_t02 0

grmzm2g056903_t02 0

grmzm2g056916_t02 0

grmzm2g056920_t02 0

grmzm2g056929_t01 0

grmzm2g056961_t01 0

grmzm2g056975_t01 0

grmzm2g056986_t01 0

grmzm2g056989_t01 0

grmzm2g056996_t01 0

grmzm2g057000_t05 0

grmzm2g057006_t01 0

grmzm2g057023_t01 0

grmzm2g057026_t01 0

grmzm2g057031_t01 0

grmzm2g057037_t02 0

grmzm2g057044_t01 0

grmzm2g057056_t02 0

grmzm2g057057_t02 0

grmzm2g057067_t04 0

grmzm2g057075_t01 0

grmzm2g057078_t02 0

grmzm2g057086_t01 -2.57

grmzm2g057091_t02 0

grmzm2g057113_t01 0

grmzm2g057129_t04 0

grmzm2g057131_t03 0

grmzm2g057150_t01 0

grmzm2g057158_t01 0

grmzm2g057159_t04 0

grmzm2g057184_t01 0

grmzm2g057186_t02 0.88

grmzm2g057231_t01 0

grmzm2g057237_t03 0

grmzm2g057243_t01 0

grmzm2g057247_t01 0

grmzm2g057251_t02 0

grmzm2g057258_t04 0

grmzm2g057281_t02 -1.8

grmzm2g057296_t01 0

grmzm2g057305_t03 0

grmzm2g057328_t01 0

grmzm2g057329_t01 0

grmzm2g057369_t01 0

grmzm2g057374_t01 0

grmzm2g057380_t01 0

grmzm2g057388_t01 0

grmzm2g057394_t02 0

grmzm2g057402_t01 0

grmzm2g057408_t04 0

grmzm2g057410_t02 0

grmzm2g057436_t01 0

grmzm2g057437_t01 0

grmzm2g057441_t01 0

grmzm2g057448_t01 0

grmzm2g057450_t03 0

grmzm2g057451_t02 0

grmzm2g057466_t01 0

grmzm2g057475_t02 0

grmzm2g057491_t01 0

grmzm2g057508_t01 0

grmzm2g057509_t01 0

grmzm2g057525_t01 0

grmzm2g057529_t01 0

grmzm2g057535_t01 0

grmzm2g057554_t01 0

grmzm2g057557_t01 0

grmzm2g057576_t02 0

grmzm2g057608_t03 0

grmzm2g057616_t01 0

grmzm2g057618_t02 0

grmzm2g057623_t01 0

grmzm2g057630_t01 0

grmzm2g057642_t02 0

grmzm2g057672_t01 0

grmzm2g057674_t01 0

grmzm2g057699_t01 0

grmzm2g057709_t02 0

grmzm2g057733_t01 0

grmzm2g057743_t01 0

grmzm2g057768_t02 0

grmzm2g057779_t01 0

grmzm2g057789_t01 2.05

grmzm2g057820_t01 0

grmzm2g057823_t01 0

grmzm2g057829_t01 0

grmzm2g057852_t01 0

grmzm2g057853_t01 0

grmzm2g057865_t02 0

grmzm2g057879_t01 0

grmzm2g057907_t01 0

grmzm2g057910_t01 0

grmzm2g057930_t01 0

grmzm2g057935_t01 0

grmzm2g057942_t02 0

grmzm2g057950_t01 0

grmzm2g057959_t01 1.06

grmzm2g057973_t01 0

grmzm2g057983_t01 0

grmzm2g058013_t01 0

grmzm2g058021_t01 0

grmzm2g058026_t01 0

grmzm2g058032_t02 -0.83

grmzm2g058039_t01 0

grmzm2g058057_t01 0

grmzm2g058078_t01 1.23

grmzm2g058089_t02 0

grmzm2g058095_t02 0

grmzm2g058098_t01 0

grmzm2g058120_t01 0

grmzm2g058128_t01 0

grmzm2g058133_t01 0

grmzm2g058138_t01 0

grmzm2g058149_t01 0

grmzm2g058158_t01 0

grmzm2g058197_t01 0

grmzm2g058227_t01 0

grmzm2g058250_t01 0

grmzm2g058252_t01 0

grmzm2g058261_t01 0

grmzm2g058276_t01 0

grmzm2g058289_t01 0

grmzm2g058292_t01 0

grmzm2g058300_t01 1.11

grmzm2g058305_t01 0

grmzm2g058310_t02 0

grmzm2g058314_t01 0

grmzm2g058345_t01 0

grmzm2g058366_t01 0

grmzm2g058374_t01 0

grmzm2g058402_t01 0

grmzm2g058404_t01 0

grmzm2g058407_t01 0

grmzm2g058421_t01 0

grmzm2g058432_t01 0

grmzm2g058444_t01 0

grmzm2g058447_t03 0

grmzm2g058450_t01 0

grmzm2g058451_t03 0

grmzm2g058456_t02 0

grmzm2g058463_t02 0

grmzm2g058472_t02 0

grmzm2g058479_t02 0

grmzm2g058496_t01 0

grmzm2g058498_t01 0

grmzm2g058511_t01 0

grmzm2g058518_t01 0

grmzm2g058522_t03 0

grmzm2g058525_t01 0

grmzm2g058528_t02 0

grmzm2g058560_t01 0

grmzm2g058568_t03 0

grmzm2g058573_t07 0

grmzm2g058584_t01 0

grmzm2g058612_t01 0

grmzm2g058655_t03 0

grmzm2g058659_t02 0

grmzm2g058675_t01 0

grmzm2g058681_t06 0

grmzm2g058702_t01 0

grmzm2g058734_t01 0.81

grmzm2g058745_t01 0

grmzm2g058760_t01 0

grmzm2g058866_t01 0

grmzm2g058870_t01 0

grmzm2g058872_t01 0

grmzm2g058900_t01 0

grmzm2g058910_t02 0

grmzm2g058923_t01 0

grmzm2g058925_t01 0

grmzm2g058948_t01 0

grmzm2g058954_t01 2.23

grmzm2g058966_t01 0

grmzm2g059013_t01 0

grmzm2g059015_t01 0

grmzm2g059018_t02 0

grmzm2g059021_t03 0

grmzm2g059026_t01 0

grmzm2g059037_t02 0

grmzm2g059042_t01 0

grmzm2g059073_t01 0

grmzm2g059083_t03 0

grmzm2g059102_t02 0

grmzm2g059106_t02 0

grmzm2g059110_t01 0

grmzm2g059117_t01 0

grmzm2g059121_t01 0

grmzm2g059129_t01 0

grmzm2g059132_t01 0

grmzm2g059138_t01 -2.9

grmzm2g059151_t01 0

grmzm2g059167_t01 0

grmzm2g059179_t01 0

grmzm2g059191_t01 0

grmzm2g059214_t01 0

grmzm2g059225_t01 0

grmzm2g059252_t01 0

grmzm2g059266_t01 0

grmzm2g059282_t01 0

grmzm2g059285_t01 0

grmzm2g059306_t02 0

grmzm2g059308_t02 0

grmzm2g059314_t03 0

grmzm2g059325_t02 0

grmzm2g059353_t01 0

grmzm2g059363_t01 0

grmzm2g059381_t02 0

grmzm2g059392_t01 -1.04

grmzm2g059393_t01 0

grmzm2g059432_t01 0

grmzm2g059449_t01 0

grmzm2g059502_t05 -0.92

grmzm2g059517_t01 0

grmzm2g059556_t01 0

grmzm2g059565_t02 0

grmzm2g059580_t01 0

grmzm2g059590_t01 0

grmzm2g059618_t04 0

grmzm2g059622_t02 0

grmzm2g059634_t01 0

grmzm2g059637_t01 0

grmzm2g059669_t02 0

grmzm2g059671_t04 0

grmzm2g059693_t01 0

grmzm2g059702_t03 0

grmzm2g059703_t03 0

grmzm2g059706_t01 0

grmzm2g059740_t01 0

grmzm2g059753_t01 0

grmzm2g059801_t01 0

grmzm2g059825_t01 0

grmzm2g059836_t01 0

grmzm2g059845_t01 0

grmzm2g059851_t02 -1.02

grmzm2g059865_t01 0

grmzm2g059887_t03 0

grmzm2g059891_t01 0

grmzm2g059922_t02 0

grmzm2g059937_t01 0.81

grmzm2g059958_t01 0

grmzm2g059964_t01 0

grmzm2g059965_t01 0

grmzm2g059974_t02 0

grmzm2g059981_t01 0

grmzm2g059985_t01 0

grmzm2g060000_t02 0

grmzm2g060011_t01 0

grmzm2g060027_t03 0

grmzm2g060029_t01 0

grmzm2g060045_t01 0

grmzm2g060057_t01 0

grmzm2g060061_t04 0

grmzm2g060070_t01 0

grmzm2g060079_t04 0

grmzm2g060109_t01 0

grmzm2g060114_t01 0

grmzm2g060148_t01 0

grmzm2g060156_t01 0

grmzm2g060160_t02 0

grmzm2g060163_t01 0

grmzm2g060167_t01 0

grmzm2g060183_t01 0

grmzm2g060185_t01 0

grmzm2g060210_t02 0

grmzm2g060213_t01 0

grmzm2g060216_t02 0

grmzm2g060257_t04 0

grmzm2g060265_t01 0

grmzm2g060276_t01 0

grmzm2g060284_t03 0

grmzm2g060296_t05 0

grmzm2g060337_t01 1.21

grmzm2g060349_t03 0

grmzm2g060355_t01 0

grmzm2g060357_t01 0

grmzm2g060373_t04 0

grmzm2g060451_t02 0

grmzm2g060467_t03 0

grmzm2g060470_t04 0

grmzm2g060481_t01 0

grmzm2g060485_t01 0

grmzm2g060505_t03 0

grmzm2g060507_t01 0

grmzm2g060509_t05 0

grmzm2g060511_t01 0

grmzm2g060516_t01 0

grmzm2g060522_t01 0

grmzm2g060536_t01 0

grmzm2g060540_t01 0

grmzm2g060554_t01 0

grmzm2g060561_t01 0

grmzm2g060564_t01 0

grmzm2g060567_t01 0

grmzm2g060583_t01 0

grmzm2g060594_t02 0

grmzm2g060611_t01 0

grmzm2g060659_t02 0

grmzm2g060669_t05 0

grmzm2g060690_t03 0

grmzm2g060702_t01 0

grmzm2g060720_t01 0

grmzm2g060726_t03 0

grmzm2g060742_t01 0

grmzm2g060760_t01 0

grmzm2g060762_t01 0

grmzm2g060765_t05 0

grmzm2g060798_t01 0

grmzm2g060800_t01 0

grmzm2g060811_t01 0

grmzm2g060817_t01 0

grmzm2g060824_t01 0

grmzm2g060837_t04 -2.22

grmzm2g060842_t02 0

grmzm2g060856_t01 0

grmzm2g060857_t03 0

grmzm2g060866_t01 0

grmzm2g060868_t01 0

grmzm2g060870_t03 0

grmzm2g060872_t04 0

grmzm2g060886_t03 0

grmzm2g060896_t02 0

grmzm2g060906_t01 0

grmzm2g060919_t02 0

grmzm2g060922_t01 0

grmzm2g060924_t01 0

grmzm2g060947_t01 0

grmzm2g060952_t01 -0.91

grmzm2g060977_t02 0

grmzm2g060987_t03 0

grmzm2g061018_t01 0

grmzm2g061023_t05 0

grmzm2g061043_t01 0

grmzm2g061078_t02 0

grmzm2g061096_t02 0

grmzm2g061099_t01 0

grmzm2g061105_t01 0

grmzm2g061127_t01 0

grmzm2g061135_t02 0

grmzm2g061156_t01 0

grmzm2g061184_t01 0

grmzm2g061186_t02 0

grmzm2g061187_t01 0

grmzm2g061202_t01 0

grmzm2g061206_t01 0

grmzm2g061230_t02 -1.59

grmzm2g061234_t03 0

grmzm2g061277_t01 0

grmzm2g061283_t02 0

grmzm2g061321_t01 0

grmzm2g061398_t01 0

grmzm2g061442_t01 0

grmzm2g061446_t01 0

grmzm2g061447_t04 0

grmzm2g061465_t03 0

grmzm2g061469_t01 -1.41

grmzm2g061472_t01 0

grmzm2g061487_t01 0

grmzm2g061515_t01 0

grmzm2g061537_t01 0

grmzm2g061542_t01 0

grmzm2g061562_t01 0

grmzm2g061596_t03 -0.85

grmzm2g061602_t01 0.82

grmzm2g061620_t03 0

grmzm2g061624_t01 0

grmzm2g061629_t01 0

grmzm2g061634_t01 0

grmzm2g061662_t03 0

grmzm2g061663_t03 0

grmzm2g061672_t01 0.81

grmzm2g061681_t02 0

grmzm2g061702_t06 0

grmzm2g061723_t03 0

grmzm2g061732_t01 0

grmzm2g061735_t01 0

grmzm2g061745_t01 0

grmzm2g061751_t01 -2.07

grmzm2g061783_t04 0

grmzm2g061798_t01 0

grmzm2g061806_t01 0

grmzm2g061830_t01 0

grmzm2g061876_t01 0

grmzm2g061885_t01 0

grmzm2g061890_t01 0

grmzm2g061900_t01 0

grmzm2g061906_t02 0

grmzm2g061910_t01 0

grmzm2g061912_t02 0

grmzm2g061922_t01 0

grmzm2g061928_t01 0

grmzm2g061932_t01 0

grmzm2g061938_t02 0

grmzm2g061950_t01 0

grmzm2g061969_t01 0

grmzm2g061980_t01 0

grmzm2g061988_t02 0

grmzm2g061990_t01 0

grmzm2g061996_t02 0

grmzm2g062024_t01 0

grmzm2g062029_t01 0

grmzm2g062045_t01 -1.26

grmzm2g062056_t01 -1.11

grmzm2g062069_t03 0

grmzm2g062100_t01 -1.56

grmzm2g062129_t01 0

grmzm2g062151_t01 -1.04

grmzm2g062154_t02 0

grmzm2g062156_t01 0

grmzm2g062158_t01 -1.07

grmzm2g062201_t02 0

grmzm2g062210_t02 0

grmzm2g062230_t01 0

grmzm2g062262_t02 0

grmzm2g062342_t02 0

grmzm2g062354_t03 0

grmzm2g062373_t02 0

grmzm2g062391_t02 0

grmzm2g062396_t01 3.13

grmzm2g062397_t01 0

grmzm2g062420_t01 0

grmzm2g062425_t04 0

grmzm2g062452_t01 0

grmzm2g062458_t01 0

grmzm2g062470_t01 0

grmzm2g062471_t01 0

grmzm2g062476_t01 0

grmzm2g062481_t01 0

grmzm2g062488_t01 0.95

grmzm2g062531_t01 0

grmzm2g062541_t01 0

grmzm2g062552_t02 0

grmzm2g062554_t03 0

grmzm2g062555_t01 0

grmzm2g062569_t01 0

grmzm2g062591_t01 0

grmzm2g062613_t01 0

grmzm2g062641_t01 0

grmzm2g062673_t01 0

grmzm2g062724_t02 0

grmzm2g062726_t01 0

grmzm2g062738_t01 0

grmzm2g062761_t02 0

grmzm2g062785_t01 0

grmzm2g062788_t01 0

grmzm2g062826_t01 0

grmzm2g062841_t01 0

grmzm2g062848_t01 0

grmzm2g062860_t02 0

grmzm2g062885_t01 0

grmzm2g062910_t01 0

grmzm2g062914_t01 0

grmzm2g062946_t01 0

grmzm2g062953_t01 0

grmzm2g063024_t01 0

grmzm2g063042_t01 0

grmzm2g063048_t01 0

grmzm2g063060_t02 0

grmzm2g063069_t02 0

grmzm2g063084_t01 0

grmzm2g063133_t01 0

grmzm2g063151_t01 0

grmzm2g063156_t01 0

grmzm2g063162_t01 0

grmzm2g063163_t01 0

grmzm2g063188_t03 0

grmzm2g063192_t01 0

grmzm2g063216_t02 0

grmzm2g063220_t01 0

grmzm2g063223_t01 0

grmzm2g063244_t01 0

grmzm2g063253_t03 0

grmzm2g063258_t01 0

grmzm2g063262_t01 0

grmzm2g063263_t01 0

grmzm2g063276_t02 0

grmzm2g063298_t02 0

grmzm2g063316_t01 0

grmzm2g063322_t01 0

grmzm2g063328_t01 0

grmzm2g063340_t01 0

grmzm2g063342_t01 0

grmzm2g063363_t01 0

grmzm2g063369_t05 0

grmzm2g063380_t01 0

grmzm2g063394_t01 0

grmzm2g063420_t01 0

grmzm2g063431_t02 0

grmzm2g063438_t01 0

grmzm2g063462_t02 0

grmzm2g063468_t01 0

grmzm2g063473_t01 0

grmzm2g063492_t01 0

grmzm2g063503_t02 0

grmzm2g063511_t03 0

grmzm2g063517_t02 0

grmzm2g063519_t01 0

grmzm2g063524_t02 0

grmzm2g063533_t01 0

grmzm2g063550_t01 0

grmzm2g063566_t01 0

grmzm2g063575_t01 0

grmzm2g063592_t01 0

grmzm2g063603_t01 0

grmzm2g063617_t02 0

grmzm2g063643_t02 0

grmzm2g063657_t01 0

grmzm2g063676_t01 0

grmzm2g063679_t01 0

grmzm2g063684_t01 0

grmzm2g063688_t04 0

grmzm2g063693_t01 0

grmzm2g063700_t03 0

grmzm2g063723_t01 0

grmzm2g063729_t04 0

grmzm2g063747_t02 0

grmzm2g063754_t04 0

grmzm2g063756_t01 -1.06

grmzm2g063775_t02 0

grmzm2g063792_t06 0

grmzm2g063806_t01 0

grmzm2g063850_t01 0

grmzm2g063851_t02 0

grmzm2g063875_t01 0

grmzm2g063882_t03 0

grmzm2g063896_t02 0

grmzm2g063897_t01 0

grmzm2g063899_t02 0

grmzm2g063917_t01 0

grmzm2g063931_t02 0

grmzm2g063942_t04 0

grmzm2g063949_t01 0

grmzm2g063961_t01 0

grmzm2g063972_t02 0

grmzm2g063975_t01 0

grmzm2g064001_t01 0

grmzm2g064005_t01 0

grmzm2g064023_t03 0

grmzm2g064031_t02 0

grmzm2g064056_t01 0

grmzm2g064091_t01 0

grmzm2g064096_t01 0

grmzm2g064111_t01 0

grmzm2g064133_t02 0

grmzm2g064136_t01 0

grmzm2g064145_t01 0

grmzm2g064159_t01 0

grmzm2g064163_t01 0

grmzm2g064193_t01 0

grmzm2g064197_t02 0

grmzm2g064202_t01 0

grmzm2g064212_t02 0

grmzm2g064255_t01 0

grmzm2g064268_t01 1.02

grmzm2g064302_t01 0

grmzm2g064328_t01 0

grmzm2g064330_t02 0

grmzm2g064336_t01 0

grmzm2g064366_t01 0

grmzm2g064371_t01 0

grmzm2g064374_t02 0

grmzm2g064382_t01 0

grmzm2g064386_t04 0

grmzm2g064390_t02 0

grmzm2g064426_t03 0

grmzm2g064437_t01 0

grmzm2g064466_t01 0

grmzm2g064467_t01 0

grmzm2g064473_t01 0.84

grmzm2g064501_t01 0

grmzm2g064537_t01 0

grmzm2g064541_t01 0

grmzm2g064547_t01 0

grmzm2g064558_t01 0

grmzm2g064563_t03 0.97

grmzm2g064580_t03 0

grmzm2g064584_t01 0

grmzm2g064590_t01 -1.09

grmzm2g064600_t01 0

grmzm2g064605_t01 0

grmzm2g064608_t01 0

grmzm2g064612_t01 0

grmzm2g064630_t01 0

grmzm2g064640_t02 0

grmzm2g064655_t01 0

grmzm2g064663_t01 0

grmzm2g064679_t01 0

grmzm2g064695_t03 0

grmzm2g064701_t01 0

grmzm2g064708_t01 0

grmzm2g064712_t02 0

grmzm2g064725_t02 0

grmzm2g064732_t02 0

grmzm2g064753_t01 0

grmzm2g064767_t02 0

grmzm2g064799_t02 0

grmzm2g064804_t02 0

grmzm2g064807_t01 0

grmzm2g064822_t01 0

grmzm2g064853_t02 0

grmzm2g064868_t02 0

grmzm2g064870_t01 0

grmzm2g064873_t01 0

grmzm2g064875_t01 0

grmzm2g064898_t04 0

grmzm2g064905_t01 0

grmzm2g064936_t01 0

grmzm2g064954_t01 0

grmzm2g064960_t01 0

grmzm2g064962_t06 0

grmzm2g064967_t02 0

grmzm2g064984_t01 0

grmzm2g064988_t02 0

grmzm2g064993_t02 0

grmzm2g065012_t01 0

grmzm2g065030_t01 0

grmzm2g065040_t01 0

grmzm2g065066_t01 0

grmzm2g065076_t02 0

grmzm2g065088_t01 0

grmzm2g065105_t01 0

grmzm2g065125_t02 0

grmzm2g065154_t01 0

grmzm2g065194_t01 0

grmzm2g065210_t06 0

grmzm2g065214_t04 0

grmzm2g065225_t01 0

grmzm2g065244_t02 0

grmzm2g065245_t01 0

grmzm2g065276_t04 0

grmzm2g065292_t01 0

grmzm2g065355_t01 0

grmzm2g065374_t01 0

grmzm2g065394_t01 -1.19

grmzm2g065451_t01 0

grmzm2g065461_t01 0

grmzm2g065494_t01 0

grmzm2g065498_t01 0

grmzm2g065532_t01 0

grmzm2g065557_t01 0

grmzm2g065566_t01 0

grmzm2g065585_t01 0

grmzm2g065593_t05 0

grmzm2g065598_t03 -1.21

grmzm2g065599_t01 0

grmzm2g065612_t02 0

grmzm2g065622_t01 0

grmzm2g065632_t03 0

grmzm2g065635_t04 0

grmzm2g065640_t01 0

grmzm2g065655_t04 0

grmzm2g065669_t01 0

grmzm2g065694_t01 0

grmzm2g065696_t01 0

grmzm2g065718_t01 -0.93

grmzm2g065734_t02 0

grmzm2g065757_t02 0

grmzm2g065804_t02 0

grmzm2g065806_t01 0

grmzm2g065822_t01 0

grmzm2g065829_t03 0

grmzm2g065839_t01 0

grmzm2g065854_t01 0

grmzm2g065868_t03 0

grmzm2g065884_t01 0

grmzm2g065888_t01 0

grmzm2g065893_t01 0

grmzm2g065896_t01 0

grmzm2g065899_t01 0.88

grmzm2g065908_t08 0

grmzm2g065913_t03 0

grmzm2g065928_t01 0

grmzm2g065950_t02 0

grmzm2g065956_t01 0

grmzm2g065970_t01 0

grmzm2g065971_t02 0

grmzm2g066024_t01 0

grmzm2g066041_t01 0

grmzm2g066051_t01 0

grmzm2g066059_t02 0

grmzm2g066080_t01 0

grmzm2g066107_t01 0

grmzm2g066153_t01 0

grmzm2g066165_t02 0

grmzm2g066169_t01 0

grmzm2g066191_t03 0

grmzm2g066213_t01 0

grmzm2g066219_t01 -0.95

grmzm2g066222_t03 0

grmzm2g066225_t01 0

grmzm2g066290_t01 0

grmzm2g066293_t01 0

grmzm2g066316_t01 0

grmzm2g066326_t01 0

grmzm2g066329_t03 0

grmzm2g066341_t01 0

grmzm2g066362_t01 0

grmzm2g066369_t01 0

grmzm2g066400_t02 0

grmzm2g066413_t01 0

grmzm2g066426_t03 0

grmzm2g066428_t01 0

grmzm2g066440_t01 0

grmzm2g066441_t01 0

grmzm2g066456_t01 0

grmzm2g066460_t02 0

grmzm2g066469_t01 0

grmzm2g066485_t02 0

grmzm2g066489_t03 0

grmzm2g066496_t01 0

grmzm2g066500_t01 1.85

grmzm2g066516_t02 -1.54

grmzm2g066555_t01 0

grmzm2g066561_t01 0

grmzm2g066612_t02 0

grmzm2g066614_t01 0

grmzm2g066615_t01 0

grmzm2g066618_t01 0

grmzm2g066650_t01 0

grmzm2g066672_t02 0

grmzm2g066749_t01 0

grmzm2g066755_t01 0

grmzm2g066815_t01 0

grmzm2g066820_t01 0

grmzm2g066867_t01 0

grmzm2g066876_t01 1.23

grmzm2g066885_t03 0

grmzm2g066902_t01 0

grmzm2g066923_t01 0.91

grmzm2g066981_t04 0

grmzm2g066996_t01 0

grmzm2g067000_t01 -0.94

grmzm2g067019_t01 0

grmzm2g067028_t01 0

grmzm2g067036_t01 0

grmzm2g067063_t04 0

grmzm2g067067_t02 0

grmzm2g067080_t01 0

grmzm2g067122_t02 -1.03

grmzm2g067156_t03 0

grmzm2g067168_t01 0

grmzm2g067171_t01 0

grmzm2g067176_t01 0

grmzm2g067183_t02 0

grmzm2g067225_t01 0

grmzm2g067235_t01 0

grmzm2g067242_t01 0

grmzm2g067265_t01 0

grmzm2g067277_t02 0

grmzm2g067299_t06 0

grmzm2g067303_t04 0

grmzm2g067306_t01 0

grmzm2g067315_t01 0

grmzm2g067371_t01 0

grmzm2g067417_t01 0

grmzm2g067421_t01 0

grmzm2g067426_t01 0

grmzm2g067453_t02 0

grmzm2g067456_t02 0

grmzm2g067460_t01 0

grmzm2g067476_t02 0

grmzm2g067489_t01 1.22

grmzm2g067511_t01 0

grmzm2g067514_t01 0

grmzm2g067520_t01 0

grmzm2g067522_t01 0

grmzm2g067546_t01 0

grmzm2g067575_t01 0

grmzm2g067581_t01 0

grmzm2g067600_t01 0

grmzm2g067601_t03 0

grmzm2g067638_t02 0

grmzm2g067646_t01 0

grmzm2g067675_t01 0

grmzm2g067702_t01 -1.04

grmzm2g067707_t02 0

grmzm2g067727_t01 0

grmzm2g067735_t01 0

grmzm2g067747_t01 0

grmzm2g067752_t03 0

grmzm2g067756_t01 0

grmzm2g067764_t02 0

grmzm2g067777_t01 0

grmzm2g067789_t01 0

grmzm2g067853_t01 0

grmzm2g067877_t01 0

grmzm2g067908_t02 0

grmzm2g067910_t01 0

grmzm2g067915_t01 0

grmzm2g067964_t02 0

grmzm2g067985_t13 0

grmzm2g067992_t01 0

grmzm2g068024_t01 0

grmzm2g068028_t01 0

grmzm2g068059_t01 0

grmzm2g068091_t01 0

grmzm2g068095_t02 0

grmzm2g068112_t01 0

grmzm2g068117_t01 0

grmzm2g068128_t03 0

grmzm2g068151_t01 0

grmzm2g068158_t01 0

grmzm2g068177_t01 0

grmzm2g068179_t02 0

grmzm2g068192_t01 0

grmzm2g068193_t01 0

grmzm2g068212_t01 0

grmzm2g068217_t01 0

grmzm2g068244_t02 0

grmzm2g068255_t02 0

grmzm2g068259_t01 -1.11

grmzm2g068277_t01 0

grmzm2g068294_t01 0

grmzm2g068316_t02 0

grmzm2g068323_t02 0

grmzm2g068328_t01 0

grmzm2g068330_t02 0

grmzm2g068340_t01 0

grmzm2g068350_t01 0

grmzm2g068361_t01 0

grmzm2g068378_t01 0

grmzm2g068382_t01 0

grmzm2g068392_t07 0

grmzm2g068436_t01 0.82

grmzm2g068465_t01 0

grmzm2g068471_t02 0

grmzm2g068479_t02 0

grmzm2g068489_t02 0

grmzm2g068496_t02 0

grmzm2g068506_t03 0

grmzm2g068519_t02 0

grmzm2g068557_t01 0

grmzm2g068566_t01 0

grmzm2g068575_t01 0

grmzm2g068586_t01 0

grmzm2g068590_t02 0

grmzm2g068657_t01 0

grmzm2g068665_t01 0

grmzm2g068690_t01 0

grmzm2g068703_t01 0

grmzm2g068707_t01 0

grmzm2g068710_t07 0

grmzm2g068714_t04 1.01

grmzm2g068715_t01 0

grmzm2g068755_t03 0

grmzm2g068808_t02 0

grmzm2g068862_t01 0

grmzm2g068904_t01 0

grmzm2g068913_t01 0

grmzm2g068917_t01 0

grmzm2g068943_t02 0

grmzm2g068947_t01 0

grmzm2g068952_t01 0

grmzm2g068955_t01 0

grmzm2g068959_t01 0

grmzm2g068963_t01 0

grmzm2g068973_t02 0

grmzm2g068984_t01 0

grmzm2g069005_t01 0

grmzm2g069008_t01 0

grmzm2g069009_t02 0

grmzm2g069018_t01 0

grmzm2g069024_t07 0

grmzm2g069035_t01 0

grmzm2g069061_t01 0

grmzm2g069078_t01 0

grmzm2g069085_t01 0

grmzm2g069092_t01 0

grmzm2g069095_t01 0

grmzm2g069098_t01 -1.37

grmzm2g069099_t05 0

grmzm2g069102_t01 0

grmzm2g069106_t01 0

grmzm2g069146_t01 -2.65

grmzm2g069162_t02 0

grmzm2g069174_t02 0

grmzm2g069177_t02 0

grmzm2g069195_t01 0

grmzm2g069198_t06 0

grmzm2g069203_t01 0

grmzm2g069208_t01 0

grmzm2g069215_t01 0

grmzm2g069262_t02 0

grmzm2g069295_t01 0

grmzm2g069298_t01 0

grmzm2g069316_t01 0

grmzm2g069317_t01 0

grmzm2g069325_t01 0

grmzm2g069408_t01 0

grmzm2g069410_t01 0

grmzm2g069424_t01 0

grmzm2g069458_t01 0

grmzm2g069476_t04 0

grmzm2g069486_t04 0

grmzm2g069518_t01 0

grmzm2g069523_t01 0

grmzm2g069528_t01 0

grmzm2g069542_t01 0

grmzm2g069569_t01 0

grmzm2g069594_t01 0

grmzm2g069596_t01 0

grmzm2g069603_t02 0

grmzm2g069618_t01 0

grmzm2g069631_t01 0

grmzm2g069649_t01 0

grmzm2g069651_t05 0

grmzm2g069656_t01 0

grmzm2g069676_t02 0

grmzm2g069678_t01 0

grmzm2g069687_t01 0

grmzm2g069694_t01 -1.82

grmzm2g069708_t01 0

grmzm2g069713_t03 -0.91

grmzm2g069722_t01 0

grmzm2g069726_t02 0

grmzm2g069758_t01 0

grmzm2g069762_t02 0.85

grmzm2g069765_t05 0

grmzm2g069772_t01 0

grmzm2g069773_t01 0

grmzm2g069805_t01 0

grmzm2g069807_t01 0

grmzm2g069816_t01 0

grmzm2g069841_t02 0

grmzm2g069856_t02 0

grmzm2g069865_t01 0

grmzm2g069886_t01 0

grmzm2g069905_t01 0

grmzm2g069911_t02 -1.82

grmzm2g069916_t02 0

grmzm2g069922_t01 0

grmzm2g069923_t01 0

grmzm2g069928_t01 0

grmzm2g069970_t02 0

grmzm2g069976_t01 1.06

grmzm2g070011_t01 0

grmzm2g070015_t01 0

grmzm2g070038_t01 0

grmzm2g070045_t01 0

grmzm2g070047_t01 0

grmzm2g070061_t01 0

grmzm2g070068_t01 0

grmzm2g070075_t01 0

grmzm2g070092_t01 0

grmzm2g070111_t03 0

grmzm2g070126_t02 0

grmzm2g070127_t02 0

grmzm2g070138_t01 0

grmzm2g070144_t02 0

grmzm2g070163_t01 0

grmzm2g070167_t01 0

grmzm2g070178_t01 -0.98

grmzm2g070199_t01 0

grmzm2g070211_t01 0

grmzm2g070218_t03 0

grmzm2g070239_t04 0

grmzm2g070252_t01 0

grmzm2g070259_t02 0

grmzm2g070264_t01 0

grmzm2g070271_t01 0

grmzm2g070284_t02 0

grmzm2g070292_t01 0

grmzm2g070305_t01 0

grmzm2g070312_t03 0

grmzm2g070315_t02 0

grmzm2g070322_t01 0

grmzm2g070323_t01 0

grmzm2g070360_t01 0

grmzm2g070375_t01 0

grmzm2g070381_t01 0

grmzm2g070389_t06 0

grmzm2g070422_t03 0

grmzm2g070429_t01 0

grmzm2g070446_t01 0

grmzm2g070487_t04 0

grmzm2g070500_t01 0

grmzm2g070508_t02 0

grmzm2g070520_t02 0

grmzm2g070542_t03 0

grmzm2g070553_t04 0

grmzm2g070555_t01 0

grmzm2g070605_t01 0

grmzm2g070620_t01 -2.34

grmzm2g070633_t01 0

grmzm2g070649_t01 0

grmzm2g070659_t02 0

grmzm2g070693_t02 0

grmzm2g070708_t04 0

grmzm2g070716_t04 0

grmzm2g070723_t02 0

grmzm2g070744_t01 0

grmzm2g070765_t01 0

grmzm2g070780_t01 0

grmzm2g070797_t01 0

grmzm2g070804_t01 0

grmzm2g070807_t01 0

grmzm2g070810_t02 0

grmzm2g070825_t01 0

grmzm2g070847_t01 0

grmzm2g070863_t01 0

grmzm2g070881_t01 0

grmzm2g070885_t01 0

grmzm2g070890_t01 0

grmzm2g070898_t01 0

grmzm2g070978_t02 0

grmzm2g071010_t04 0

grmzm2g071015_t01 0

grmzm2g071021_t03 0

grmzm2g071034_t02 0

grmzm2g071042_t01 -1.02

grmzm2g071059_t01 0

grmzm2g071071_t01 0

grmzm2g071076_t01 0

grmzm2g071087_t02 0

grmzm2g071089_t01 0

grmzm2g071100_t01 0

grmzm2g071113_t01 0

grmzm2g071119_t01 0

grmzm2g071147_t01 0

grmzm2g071154_t03 0

grmzm2g071162_t01 0

grmzm2g071163_t01 0

grmzm2g071166_t02 0

grmzm2g071172_t01 0

grmzm2g071208_t03 0

grmzm2g071249_t02 0

grmzm2g071253_t01 0

grmzm2g071268_t01 0

grmzm2g071272_t01 -1.21

grmzm2g071288_t02 0

grmzm2g071294_t02 0

grmzm2g071304_t01 0

grmzm2g071307_t01 0

grmzm2g071310_t01 0

grmzm2g071319_t02 0

grmzm2g071333_t01 0

grmzm2g071343_t01 -1.58

grmzm2g071347_t01 0

grmzm2g071349_t01 0

grmzm2g071360_t02 0

grmzm2g071370_t02 1.15

grmzm2g071378_t02 0

grmzm2g071423_t01 0

grmzm2g071441_t03 0

grmzm2g071448_t01 0

grmzm2g071450_t01 0

grmzm2g071478_t01 -1.77

grmzm2g071491_t01 0

grmzm2g071518_t01 0

grmzm2g071573_t01 0

grmzm2g071582_t02 0

grmzm2g071589_t04 0

grmzm2g071602_t01 0

grmzm2g071613_t02 0

grmzm2g071626_t01 0

grmzm2g071638_t01 -0.98

grmzm2g071705_t01 -0.86

grmzm2g071714_t01 0

grmzm2g071720_t01 -1.13

grmzm2g071745_t02 0

grmzm2g071759_t04 0

grmzm2g071768_t03 0

grmzm2g071790_t02 0

grmzm2g071832_t01 0

grmzm2g071844_t01 0

grmzm2g071869_t02 0

grmzm2g071871_t01 0

grmzm2g071877_t01 0

grmzm2g071907_t02 0

grmzm2g071959_t01 0

grmzm2g071970_t01 0

grmzm2g071977_t01 0

grmzm2g071986_t01 0

grmzm2g071987_t01 0

grmzm2g071996_t01 0

grmzm2g071997_t02 0

grmzm2g072018_t01 0

grmzm2g072029_t02 0

grmzm2g072041_t01 0

grmzm2g072054_t01 0

grmzm2g072065_t02 0

grmzm2g072071_t01 0

grmzm2g072080_t03 0

grmzm2g072088_t01 0

grmzm2g072091_t01 0

grmzm2g072107_t03 0

grmzm2g072117_t03 0

grmzm2g072142_t01 0

grmzm2g072146_t01 0

grmzm2g072156_t01 0

grmzm2g072171_t02 0

grmzm2g072174_t01 0

grmzm2g072210_t01 0

grmzm2g072231_t01 0

grmzm2g072238_t02 0

grmzm2g072264_t02 0

grmzm2g072280_t01 -1.08

grmzm2g072298_t01 5.89

grmzm2g072300_t01 0

grmzm2g072315_t02 0

grmzm2g072337_t03 0

grmzm2g072339_t02 0

grmzm2g072350_t01 0

grmzm2g072365_t01 0

grmzm2g072383_t01 0

grmzm2g072388_t01 0

grmzm2g072406_t02 0

grmzm2g072415_t01 0

grmzm2g072462_t01 0

grmzm2g072501_t02 0

grmzm2g072506_t01 0

grmzm2g072513_t02 0

grmzm2g072518_t01 0.82

grmzm2g072529_t01 0

grmzm2g072550_t01 0

grmzm2g072569_t01 0

grmzm2g072573_t02 0

grmzm2g072578_t01 0

grmzm2g072584_t02 0

grmzm2g072612_t01 0

grmzm2g072658_t01 0

grmzm2g072669_t01 0

grmzm2g072682_t04 0

grmzm2g072690_t04 1.58

grmzm2g072700_t05 0

grmzm2g072729_t03 0

grmzm2g072744_t01 0

grmzm2g072755_t01 0

grmzm2g072806_t01 0

grmzm2g072808_t01 0

grmzm2g072820_t01 0

grmzm2g072853_t01 0

grmzm2g072855_t02 0

grmzm2g072865_t05 0

grmzm2g072868_t01 0

grmzm2g072892_t02 0

grmzm2g072894_t01 0.85

grmzm2g072909_t03 0

grmzm2g072911_t01 0

grmzm2g073017_t01 0

grmzm2g073023_t03 0

grmzm2g073045_t03 0

grmzm2g073054_t01 0

grmzm2g073059_t01 0

grmzm2g073077_t01 0

grmzm2g073123_t01 0

grmzm2g073150_t01 0

grmzm2g073192_t01 0

grmzm2g073197_t02 0

grmzm2g073199_t02 0

grmzm2g073223_t01 0

grmzm2g073228_t02 0

grmzm2g073250_t02 0

grmzm2g073275_t02 -1.01

grmzm2g073310_t01 0

grmzm2g073312_t01 0

grmzm2g073351_t01 -0.88

grmzm2g073376_t01 0

grmzm2g073377_t03 0

grmzm2g073396_t01 0

grmzm2g073399_t02 0

grmzm2g073404_t03 0

grmzm2g073427_t05 -1.02

grmzm2g073429_t01 0

grmzm2g073460_t01 0

grmzm2g073462_t01 -1.03

grmzm2g073465_t01 0

grmzm2g073495_t03 0

grmzm2g073498_t01 0

grmzm2g073504_t01 0

grmzm2g073505_t01 0

grmzm2g073510_t03 0

grmzm2g073511_t02 0

grmzm2g073521_t01 0

grmzm2g073535_t02 0

grmzm2g073551_t01 0

grmzm2g073555_t01 0

grmzm2g073567_t01 1.16

grmzm2g073571_t03 0

grmzm2g073581_t01 0

grmzm2g073609_t01 0

grmzm2g073622_t05 0

grmzm2g073628_t02 0

grmzm2g073630_t01 0

grmzm2g073668_t01 0

grmzm2g073693_t02 -1

grmzm2g073725_t01 0

grmzm2g073741_t01 0

grmzm2g073743_t02 0

grmzm2g073750_t01 0

grmzm2g073754_t02 0

grmzm2g073755_t02 0

grmzm2g073764_t01 0

grmzm2g073770_t01 0

grmzm2g073788_t01 0

grmzm2g073814_t01 -1.02

grmzm2g073826_t01 0

grmzm2g073836_t01 0

grmzm2g073860_t01 0

grmzm2g073861_t02 0

grmzm2g073888_t02 0

grmzm2g073892_t01 0

grmzm2g073912_t02 0

grmzm2g073928_t01 0

grmzm2g073934_t04 0

grmzm2g073942_t01 0

grmzm2g073943_t01 0

grmzm2g073950_t01 0

grmzm2g073954_t01 0

grmzm2g073959_t01 0

grmzm2g073979_t02 0

grmzm2g074015_t03 0

grmzm2g074024_t03 0

grmzm2g074028_t01 0

grmzm2g074037_t01 0

grmzm2g074040_t02 0

grmzm2g074053_t01 0

grmzm2g074082_t01 0

grmzm2g074083_t01 0

grmzm2g074085_t03 0

grmzm2g074087_t01 0

grmzm2g074097_t01 -0.82

grmzm2g074099_t02 0

grmzm2g074102_t02 0

grmzm2g074103_t02 0

grmzm2g074107_t02 -0.85

grmzm2g074114_t01 0

grmzm2g074122_t02 0

grmzm2g074138_t01 0

grmzm2g074158_t01 0

grmzm2g074169_t04 0

grmzm2g074173_t01 1.37

grmzm2g074193_t01 0

grmzm2g074238_t06 0

grmzm2g074245_t01 0

grmzm2g074252_t01 0

grmzm2g074254_t01 0

grmzm2g074262_t01 0

grmzm2g074267_t01 0

grmzm2g074270_t01 0

grmzm2g074278_t01 0

grmzm2g074280_t02 0

grmzm2g074282_t01 0

grmzm2g074300_t02 0

grmzm2g074314_t01 0

grmzm2g074317_t02 0

grmzm2g074323_t02 0

grmzm2g074351_t03 0

grmzm2g074373_t01 0

grmzm2g074377_t01 -0.87

grmzm2g074386_t07 0

grmzm2g074393_t01 0

grmzm2g074401_t01 0

grmzm2g074404_t01 0

grmzm2g074414_t01 0

grmzm2g074436_t01 0

grmzm2g074454_t01 0

grmzm2g074466_t01 0

grmzm2g074472_t01 0

grmzm2g074479_t05 0

grmzm2g074489_t02 0

grmzm2g074496_t01 0

grmzm2g074514_t02 0

grmzm2g074517_t01 0

grmzm2g074530_t01 0

grmzm2g074531_t03 0

grmzm2g074549_t02 0

grmzm2g074567_t01 0

grmzm2g074569_t01 0

grmzm2g074572_t01 0

grmzm2g074589_t01 0

grmzm2g074599_t01 0

grmzm2g074604_t02 -0.84

grmzm2g074631_t01 -1.65

grmzm2g074634_t06 0

grmzm2g074645_t01 0

grmzm2g074687_t01 0

grmzm2g074689_t03 0

grmzm2g074718_t01 0

grmzm2g074735_t02 -1.16

grmzm2g074742_t01 -1.45

grmzm2g074759_t01 0

grmzm2g074787_t01 0

grmzm2g074790_t01 0

grmzm2g074792_t02 0

grmzm2g074804_t01 0

grmzm2g074805_t01 0

grmzm2g074819_t02 0

grmzm2g074821_t03 0

grmzm2g074850_t01 0

grmzm2g074857_t02 0

grmzm2g074871_t01 0

grmzm2g074896_t02 -0.88

grmzm2g074898_t04 0

grmzm2g074914_t01 -0.93

grmzm2g074956_t01 0

grmzm2g074957_t02 0

grmzm2g075002_t02 0

grmzm2g075003_t01 0

grmzm2g075027_t04 0

grmzm2g075058_t01 0

grmzm2g075074_t02 0

grmzm2g075092_t01 0

grmzm2g075101_t01 0

grmzm2g075104_t01 0

grmzm2g075117_t01 0

grmzm2g075124_t02 0

grmzm2g075132_t01 0

grmzm2g075136_t02 0

grmzm2g075140_t01 0

grmzm2g075150_t01 0

grmzm2g075153_t01 0

grmzm2g075158_t01 0

grmzm2g075196_t01 0

grmzm2g075223_t01 0

grmzm2g075227_t01 0

grmzm2g075250_t01 0

grmzm2g075255_t02 0

grmzm2g075257_t02 0

grmzm2g075265_t01 0

grmzm2g075268_t02 0

grmzm2g075269_t01 0

grmzm2g075294_t01 0

grmzm2g075295_t01 0

grmzm2g075315_t02 0

grmzm2g075320_t01 0

grmzm2g075336_t01 0

grmzm2g075368_t01 0

grmzm2g075384_t01 0

grmzm2g075408_t02 0

grmzm2g075431_t02 0

grmzm2g075438_t01 1.18

grmzm2g075470_t01 0

grmzm2g075488_t02 0

grmzm2g075496_t04 0

grmzm2g075502_t01 1.06

grmzm2g075513_t01 0

grmzm2g075528_t03 0

grmzm2g075551_t03 0

grmzm2g075562_t02 0

grmzm2g075582_t01 0

grmzm2g075584_t01 0

grmzm2g075594_t03 0

grmzm2g075619_t01 0

grmzm2g075622_t01 0

grmzm2g075624_t02 0

grmzm2g075628_t01 0

grmzm2g075630_t01 0

grmzm2g075637_t01 1.21

grmzm2g075651_t01 0

grmzm2g075655_t01 0

grmzm2g075676_t04 0

grmzm2g075680_t01 0

grmzm2g075683_t02 0

grmzm2g075690_t05 0

grmzm2g075701_t01 -1.22

grmzm2g075712_t01 0

grmzm2g075719_t02 0

grmzm2g075744_t01 0

grmzm2g075767_t05 0

grmzm2g075775_t02 0

grmzm2g075796_t01 0

grmzm2g075837_t01 0

grmzm2g075839_t01 0

grmzm2g075844_t02 0

grmzm2g075851_t01 0

grmzm2g075884_t01 0

grmzm2g075900_t01 0

grmzm2g075942_t01 0

grmzm2g075956_t01 0

grmzm2g075958_t01 0

grmzm2g075974_t01 0

grmzm2g075992_t01 0

grmzm2g076006_t02 0

grmzm2g076029_t01 0

grmzm2g076062_t02 0

grmzm2g076075_t01 0

grmzm2g076084_t01 0

grmzm2g076087_t02 0

grmzm2g076104_t02 0

grmzm2g076145_t05 0

grmzm2g076152_t01 0

grmzm2g076225_t02 -0.95

grmzm2g076257_t01 0

grmzm2g076263_t01 0

grmzm2g076272_t02 0

grmzm2g076276_t03 0

grmzm2g076279_t01 0

grmzm2g076303_t01 0

grmzm2g076307_t01 -1.27

grmzm2g076348_t01 0

grmzm2g076351_t01 0

grmzm2g076370_t01 0

grmzm2g076387_t01 0

grmzm2g076392_t03 0

grmzm2g076399_t02 0

grmzm2g076415_t03 0

grmzm2g076423_t01 0

grmzm2g076435_t01 0

grmzm2g076450_t01 0

grmzm2g076484_t04 0

grmzm2g076524_t01 0

grmzm2g076526_t01 0

grmzm2g076537_t05 0.99

grmzm2g076539_t02 0

grmzm2g076542_t02 0

grmzm2g076544_t01 0

grmzm2g076597_t01 0

grmzm2g076602_t01 0

grmzm2g076613_t01 0

grmzm2g076630_t01 0

grmzm2g076631_t01 0

grmzm2g076636_t01 0

grmzm2g076657_t01 0

grmzm2g076673_t01 0

grmzm2g076676_t01 0

grmzm2g076683_t01 0

grmzm2g076705_t01 0

grmzm2g076730_t02 0

grmzm2g076755_t01 0

grmzm2g076771_t01 0

grmzm2g076802_t02 0

grmzm2g076810_t01 0

grmzm2g076826_t01 0

grmzm2g076827_t01 0

grmzm2g076841_t01 0

grmzm2g076844_t02 0

grmzm2g076868_t01 0

grmzm2g076885_t01 0

grmzm2g076946_t02 0

grmzm2g076950_t01 0

grmzm2g076962_t01 0

grmzm2g076985_t01 0

grmzm2g076987_t01 0

grmzm2g077002_t02 0

grmzm2g077015_t01 0

grmzm2g077024_t01 0

grmzm2g077036_t01 0

grmzm2g077054_t01 0

grmzm2g077068_t01 0

grmzm2g077079_t01 1.26

grmzm2g077088_t01 0

grmzm2g077125_t01 0

grmzm2g077127_t01 0

grmzm2g077131_t01 0

grmzm2g077147_t01 0

grmzm2g077150_t01 0

grmzm2g077181_t03 0

grmzm2g077187_t01 0

grmzm2g077202_t01 0

grmzm2g077206_t01 0

grmzm2g077208_t02 0

grmzm2g077212_t01 0

grmzm2g077214_t01 0

grmzm2g077215_t01 0

grmzm2g077222_t01 0

grmzm2g077253_t01 0

grmzm2g077256_t01 0

grmzm2g077258_t03 0

grmzm2g077259_t01 0

grmzm2g077279_t01 0

grmzm2g077293_t01 0

grmzm2g077295_t01 0

grmzm2g077299_t02 0

grmzm2g077307_t01 0

grmzm2g077316_t02 0

grmzm2g077320_t01 0

grmzm2g077333_t02 0

grmzm2g077356_t01 0

grmzm2g077361_t01 0.87

grmzm2g077401_t01 0

grmzm2g077404_t01 0

grmzm2g077406_t01 0

grmzm2g077415_t02 0

grmzm2g077420_t01 0

grmzm2g077436_t02 0

grmzm2g077458_t01 0

grmzm2g077460_t01 -2.35

grmzm2g077463_t01 0.96

grmzm2g077486_t01 0

grmzm2g077488_t01 0

grmzm2g077490_t02 1.18

grmzm2g077503_t01 0

grmzm2g077525_t01 0

grmzm2g077531_t01 0

grmzm2g077541_t01 0

grmzm2g077546_t01 0

grmzm2g077553_t01 0

grmzm2g077596_t01 0

grmzm2g077607_t04 0

grmzm2g077632_t01 0

grmzm2g077655_t01 0

grmzm2g077659_t01 0

grmzm2g077662_t02 0

grmzm2g077669_t01 0

grmzm2g077673_t01 0

grmzm2g077744_t01 0

grmzm2g077752_t01 0

grmzm2g077755_t01 0

grmzm2g077757_t01 0

grmzm2g077760_t01 0

grmzm2g077769_t01 0

grmzm2g077780_t02 0

grmzm2g077784_t02 0

grmzm2g077789_t01 0

grmzm2g077797_t02 0

grmzm2g077809_t01 0

grmzm2g077811_t01 0

grmzm2g077823_t02 0

grmzm2g077837_t05 0

grmzm2g077844_t02 0

grmzm2g077851_t02 0

grmzm2g077863_t02 0

grmzm2g077895_t01 0

grmzm2g077897_t01 0

grmzm2g077914_t02 0

grmzm2g077937_t01 0

grmzm2g077942_t01 0

grmzm2g077960_t01 0

grmzm2g077981_t04 0

grmzm2g077989_t01 0

grmzm2g077991_t01 0

grmzm2g078022_t04 0

grmzm2g078024_t01 0

grmzm2g078033_t03 0

grmzm2g078052_t01 0

grmzm2g078090_t01 0

grmzm2g078092_t01 0

grmzm2g078118_t02 0

grmzm2g078124_t01 0

grmzm2g078135_t01 0

grmzm2g078143_t01 0

grmzm2g078154_t04 0

grmzm2g078157_t01 0

grmzm2g078164_t01 0

grmzm2g078174_t01 0

grmzm2g078178_t02 0

grmzm2g078180_t03 0

grmzm2g078198_t01 0

grmzm2g078200_t01 0

grmzm2g078204_t01 0

grmzm2g078210_t01 -1.67

grmzm2g078238_t01 0

grmzm2g078252_t02 0

grmzm2g078267_t01 0

grmzm2g078272_t02 0

grmzm2g078274_t03 0

grmzm2g078275_t02 0

grmzm2g078283_t01 0

grmzm2g078292_t01 0

grmzm2g078314_t01 0

grmzm2g078324_t01 0

grmzm2g078360_t02 0

grmzm2g078362_t01 0

grmzm2g078373_t01 0

grmzm2g078379_t01 0

grmzm2g078396_t01 0

grmzm2g078412_t03 0

grmzm2g078416_t01 0

grmzm2g078440_t03 0

grmzm2g078458_t05 0

grmzm2g078465_t01 2.54

grmzm2g078480_t01 0

grmzm2g078500_t01 0

grmzm2g078501_t03 0

grmzm2g078508_t03 0

grmzm2g078517_t01 0

grmzm2g078526_t01 0

grmzm2g078541_t01 0

grmzm2g078566_t01 0

grmzm2g078569_t02 0

grmzm2g078576_t03 0

grmzm2g078595_t01 0

grmzm2g078623_t01 0

grmzm2g078636_t01 -0.96

grmzm2g078638_t05 0

grmzm2g078641_t02 0

grmzm2g078650_t02 -0.83

grmzm2g078656_t02 0

grmzm2g078691_t01 0

grmzm2g078723_t01 0

grmzm2g078725_t01 0

grmzm2g078754_t03 1.12

grmzm2g078756_t02 0

grmzm2g078757_t01 0

grmzm2g078781_t01 0

grmzm2g078806_t01 0

grmzm2g078820_t01 0

grmzm2g078826_t01 0

grmzm2g078832_t01 0

grmzm2g078839_t01 0

grmzm2g078876_t02 0

grmzm2g078887_t01 0

grmzm2g078890_t01 0

grmzm2g078894_t01 0

grmzm2g078895_t02 0

grmzm2g078924_t02 0

grmzm2g078926_t01 0

grmzm2g078933_t01 0

grmzm2g078941_t01 0

grmzm2g078959_t01 0

grmzm2g078963_t02 0

grmzm2g078985_t02 0

grmzm2g078995_t01 0

grmzm2g079013_t02 0

grmzm2g079031_t01 0

grmzm2g079034_t01 0

grmzm2g079066_t01 0

grmzm2g079083_t01 0

grmzm2g079089_t01 0

grmzm2g079109_t01 0

grmzm2g079112_t02 0

grmzm2g079127_t01 0

grmzm2g079143_t02 0

grmzm2g079196_t01 0

grmzm2g079200_t01 0

grmzm2g079226_t01 0

grmzm2g079231_t01 0

grmzm2g079236_t01 0

grmzm2g079256_t02 0

grmzm2g079257_t01 0

grmzm2g079281_t01 0

grmzm2g079306_t01 0

grmzm2g079308_t02 1.13

grmzm2g079323_t01 1.62

grmzm2g079342_t01 0

grmzm2g079348_t04 0

grmzm2g079352_t01 0

grmzm2g079353_t01 0

grmzm2g079365_t01 0

grmzm2g079381_t04 0

grmzm2g079389_t01 0

grmzm2g079397_t03 0

grmzm2g079403_t01 0

grmzm2g079409_t01 0

grmzm2g079428_t01 0

grmzm2g079436_t01 1.14

grmzm2g079452_t01 0

grmzm2g079468_t02 0

grmzm2g079470_t01 0

grmzm2g079471_t02 0

grmzm2g079487_t02 0

grmzm2g079490_t01 0

grmzm2g079538_t02 0

grmzm2g079545_t01 0

grmzm2g079554_t01 -0.98

grmzm2g079583_t01 0

grmzm2g079616_t01 0

grmzm2g079625_t01 0

grmzm2g079632_t02 -0.87

grmzm2g079653_t01 0

grmzm2g079658_t01 0

grmzm2g079668_t01 0

grmzm2g079682_t01 0

grmzm2g079723_t01 0

grmzm2g079746_t02 0

grmzm2g079759_t01 0

grmzm2g079772_t01 0

grmzm2g079777_t01 0

grmzm2g079782_t01 0

grmzm2g079784_t05 0

grmzm2g079796_t01 0

grmzm2g079807_t01 0

grmzm2g079817_t01 0

grmzm2g079823_t02 0

grmzm2g079832_t01 0

grmzm2g079839_t01 0

grmzm2g079850_t02 0

grmzm2g079873_t02 0

grmzm2g079888_t01 0

grmzm2g079889_t01 0.9

grmzm2g079904_t01 0

grmzm2g079908_t01 0

grmzm2g079915_t01 0

grmzm2g079938_t02 0

grmzm2g079944_t01 0

grmzm2g079949_t01 -0.86

grmzm2g079956_t01 -1.02

grmzm2g079957_t01 -1.83

grmzm2g079980_t01 0

grmzm2g080001_t01 0

grmzm2g080034_t02 0

grmzm2g080041_t01 0

grmzm2g080044_t02 0

grmzm2g080045_t02 0

grmzm2g080048_t02 0

grmzm2g080079_t02 1.58

grmzm2g080107_t01 0

grmzm2g080120_t02 0

grmzm2g080129_t01 0

grmzm2g080139_t01 1

grmzm2g080156_t04 0

grmzm2g080168_t03 0

grmzm2g080178_t03 0

grmzm2g080183_t01 0

grmzm2g080222_t03 0

grmzm2g080231_t02 -0.82

grmzm2g080247_t01 0

grmzm2g080256_t01 0

grmzm2g080270_t01 0

grmzm2g080274_t03 -1.18

grmzm2g080307_t03 0

grmzm2g080320_t02 -0.81

grmzm2g080355_t01 0

grmzm2g080375_t05 0

grmzm2g080380_t01 0

grmzm2g080439_t02 0

grmzm2g080450_t01 0

grmzm2g080462_t05 0

grmzm2g080466_t04 0

grmzm2g080487_t01 0

grmzm2g080497_t08 0.95

grmzm2g080499_t02 0

grmzm2g080503_t01 0

grmzm2g080509_t03 0

grmzm2g080516_t01 -1.44

grmzm2g080519_t01 0

grmzm2g080521_t01 0

grmzm2g080524_t01 0

grmzm2g080530_t01 0

grmzm2g080537_t01 1.02

grmzm2g080565_t02 0

grmzm2g080567_t01 0

grmzm2g080583_t01 0

grmzm2g080588_t01 0

grmzm2g080603_t03 0

grmzm2g080608_t01 0

grmzm2g080642_t01 0

grmzm2g080644_t02 0

grmzm2g080650_t01 0

grmzm2g080652_t01 0

grmzm2g080663_t02 0

grmzm2g080722_t02 0

grmzm2g080725_t04 0

grmzm2g080746_t01 0

grmzm2g080764_t01 0

grmzm2g080772_t01 0

grmzm2g080775_t01 0

grmzm2g080816_t04 0

grmzm2g080842_t01 0

grmzm2g080851_t01 0

grmzm2g080871_t02 0

grmzm2g080889_t01 1.13

grmzm2g080898_t01 0

grmzm2g080912_t01 0

grmzm2g080917_t02 -0.82

grmzm2g080930_t01 0

grmzm2g080937_t02 0

grmzm2g080940_t01 0

grmzm2g080992_t01 1.37

grmzm2g081012_t01 0

grmzm2g081013_t01 0

grmzm2g081032_t01 0

grmzm2g081037_t01 0

grmzm2g081048_t01 0

grmzm2g081075_t01 0

grmzm2g081077_t01 0

grmzm2g081090_t01 0

grmzm2g081102_t03 0

grmzm2g081112_t01 0

grmzm2g081114_t01 0

grmzm2g081127_t03 0

grmzm2g081144_t02 0

grmzm2g081155_t01 0.89

grmzm2g081158_t02 0

grmzm2g081172_t01 0

grmzm2g081175_t02 0

grmzm2g081180_t01 0

grmzm2g081188_t01 0

grmzm2g081192_t01 0

grmzm2g081221_t01 0

grmzm2g081239_t01 0

grmzm2g081268_t02 0

grmzm2g081310_t01 0

grmzm2g081322_t01 0

grmzm2g081350_t01 0

grmzm2g081406_t01 0

grmzm2g081429_t01 0

grmzm2g081474_t01 0

grmzm2g081504_t09 0

grmzm2g081511_t01 0

grmzm2g081519_t03 0

grmzm2g081521_t02 0

grmzm2g081529_t02 0

grmzm2g081541_t02 0

grmzm2g081554_t03 0

grmzm2g081571_t05 0

grmzm2g081580_t03 0

grmzm2g081582_t02 0

grmzm2g081589_t01 0

grmzm2g081603_t01 0

grmzm2g081615_t01 0

grmzm2g081622_t01 0

grmzm2g081626_t01 0

grmzm2g081639_t01 0

grmzm2g081642_t01 0

grmzm2g081652_t01 -0.83

grmzm2g081653_t03 0

grmzm2g081666_t02 0

grmzm2g081668_t01 0

grmzm2g081671_t01 0

grmzm2g081676_t01 0

grmzm2g081682_t01 0

grmzm2g081719_t01 0

grmzm2g081729_t01 0

grmzm2g081745_t02 0

grmzm2g081774_t01 0

grmzm2g081782_t01 0

grmzm2g081790_t01 0

grmzm2g081803_t01 0

grmzm2g081812_t01 0

grmzm2g081816_t01 0

grmzm2g081826_t02 0

grmzm2g081829_t02 0

grmzm2g081838_t01 0

grmzm2g081841_t01 0

grmzm2g081843_t01 0

grmzm2g081857_t01 0

grmzm2g081883_t01 0

grmzm2g081886_t01 0

grmzm2g081888_t01 0

grmzm2g081892_t01 -0.99

grmzm2g081915_t01 0

grmzm2g081919_t01 0

grmzm2g081924_t01 0

grmzm2g081935_t02 0

grmzm2g081943_t04 -1.3

grmzm2g081949_t01 -0.92

grmzm2g081955_t01 0

grmzm2g081957_t02 -1.26

grmzm2g081965_t01 0

grmzm2g081970_t01 0

grmzm2g081977_t03 -0.91

grmzm2g082007_t01 0

grmzm2g082019_t01 0

grmzm2g082032_t01 -1.08

grmzm2g082037_t01 0

grmzm2g082087_t01 0

grmzm2g082131_t01 0

grmzm2g082149_t01 0

grmzm2g082167_t01 0

grmzm2g082169_t01 0

grmzm2g082180_t01 0

grmzm2g082181_t01 0

grmzm2g082184_t02 1.41

grmzm2g082185_t01 0

grmzm2g082191_t01 0

grmzm2g082198_t01 0

grmzm2g082199_t01 0

grmzm2g082203_t02 0

grmzm2g082205_t01 0

grmzm2g082207_t04 0

grmzm2g082214_t01 0

grmzm2g082216_t04 0

grmzm2g082222_t01 0

grmzm2g082227_t01 0

grmzm2g082249_t01 0

grmzm2g082257_t03 0

grmzm2g082260_t04 0

grmzm2g082264_t01 0

grmzm2g082271_t01 0

grmzm2g082302_t01 0

grmzm2g082312_t01 0

grmzm2g082322_t02 0

grmzm2g082330_t02 0

grmzm2g082335_t01 0

grmzm2g082346_t01 0

grmzm2g082372_t01 -1

grmzm2g082376_t01 -0.88

grmzm2g082387_t01 0

grmzm2g082390_t01 0

grmzm2g082434_t03 0

grmzm2g082448_t01 0

grmzm2g082468_t02 0

grmzm2g082484_t01 0

grmzm2g082502_t01 0

grmzm2g082508_t01 0

grmzm2g082520_t01 0

grmzm2g082522_t03 0

grmzm2g082529_t02 0

grmzm2g082544_t01 1.01

grmzm2g082564_t01 0

grmzm2g082580_t01 0

grmzm2g082581_t01 0

grmzm2g082608_t01 0

grmzm2g082613_t01 0

grmzm2g082633_t01 0

grmzm2g082640_t03 0

grmzm2g082642_t02 0

grmzm2g082664_t01 0

grmzm2g082670_t01 0

grmzm2g082683_t03 0

grmzm2g082707_t01 0

grmzm2g082721_t01 0

grmzm2g082745_t01 0

grmzm2g082792_t02 0

grmzm2g082809_t01 0

grmzm2g082836_t01 0

grmzm2g082853_t01 0

grmzm2g082855_t05 0

grmzm2g082874_t02 0.96

grmzm2g082894_t01 0

grmzm2g082906_t01 0

grmzm2g082916_t01 0

grmzm2g082924_t02 0

grmzm2g082931_t01 0

grmzm2g082940_t01 -1.28

grmzm2g082941_t01 0

grmzm2g082962_t01 0

grmzm2g082974_t02 0

grmzm2g082976_t01 0

grmzm2g082998_t02 0

grmzm2g083016_t02 0

grmzm2g083058_t01 0

grmzm2g083068_t02 0

grmzm2g083076_t01 0

grmzm2g083081_t03 0

grmzm2g083091_t01 0

grmzm2g083095_t01 0

grmzm2g083102_t05 0

grmzm2g083117_t02 0

grmzm2g083130_t01 0

grmzm2g083150_t01 0

grmzm2g083173_t01 0

grmzm2g083176_t02 0

grmzm2g083195_t02 -1.26

grmzm2g083243_t02 0

grmzm2g083253_t05 0

grmzm2g083262_t01 0

grmzm2g083284_t02 0

grmzm2g083309_t03 0

grmzm2g083344_t02 0

grmzm2g083346_t02 0

grmzm2g083347_t02 0

grmzm2g083367_t01 0

grmzm2g083374_t01 0

grmzm2g083382_t01 0

grmzm2g083402_t01 0

grmzm2g083410_t02 0

grmzm2g083418_t01 0

grmzm2g083427_t04 0

grmzm2g083437_t01 0

grmzm2g083453_t01 0

grmzm2g083459_t01 0

grmzm2g083497_t02 0

grmzm2g083504_t03 0

grmzm2g083518_t01 0

grmzm2g083526_t02 0

grmzm2g083538_t01 0

grmzm2g083546_t01 0

grmzm2g083551_t03 0

grmzm2g083555_t01 0

grmzm2g083580_t02 0

grmzm2g083620_t02 0

grmzm2g083642_t01 0

grmzm2g083655_t02 0

grmzm2g083677_t01 0

grmzm2g083689_t02 0

grmzm2g083711_t01 0

grmzm2g083716_t01 0

grmzm2g083725_t01 0

grmzm2g083745_t04 0

grmzm2g083749_t04 0

grmzm2g083755_t02 0

grmzm2g083759_t01 0

grmzm2g083763_t01 0

grmzm2g083783_t02 0

grmzm2g083788_t01 0

grmzm2g083797_t01 0

grmzm2g083812_t01 0

grmzm2g083813_t01 0

grmzm2g083836_t03 0

grmzm2g083841_t02 0

grmzm2g083847_t05 0

grmzm2g083875_t01 0

grmzm2g083886_t01 0

grmzm2g083894_t01 -0.96

grmzm2g083932_t01 0

grmzm2g083935_t01 0

grmzm2g083950_t02 0

grmzm2g083972_t01 0

grmzm2g083998_t01 0

grmzm2g084014_t01 0

grmzm2g084021_t01 0

grmzm2g084046_t04 0

grmzm2g084062_t01 0

grmzm2g084063_t02 0

grmzm2g084134_t01 0

grmzm2g084149_t02 0

grmzm2g084164_t03 0

grmzm2g084181_t01 0

grmzm2g084183_t01 0

grmzm2g084192_t01 0

grmzm2g084195_t03 0

grmzm2g084252_t01 0

grmzm2g084274_t01 0

grmzm2g084279_t01 -0.84

grmzm2g084325_t01 0

grmzm2g084327_t01 0

grmzm2g084369_t02 0

grmzm2g084406_t02 0

grmzm2g084407_t01 0

grmzm2g084429_t01 0

grmzm2g084445_t01 -1.36

grmzm2g084463_t03 0

grmzm2g084465_t03 0

grmzm2g084477_t01 0

grmzm2g084486_t01 0

grmzm2g084491_t01 0

grmzm2g084513_t04 0

grmzm2g084515_t01 0

grmzm2g084521_t02 0

grmzm2g084525_t02 0

grmzm2g084540_t01 0

grmzm2g084547_t02 0

grmzm2g084570_t01 0

grmzm2g084583_t01 -1.27

grmzm2g084586_t01 0

grmzm2g084587_t01 0

grmzm2g084604_t01 0

grmzm2g084606_t03 0.85

grmzm2g084609_t01 0

grmzm2g084647_t03 0

grmzm2g084684_t02 0

grmzm2g084694_t01 0

grmzm2g084719_t01 0

grmzm2g084727_t02 0

grmzm2g084729_t01 0

grmzm2g084739_t03 0

grmzm2g084757_t01 0

grmzm2g084767_t01 0

grmzm2g084783_t01 0

grmzm2g084791_t02 0

grmzm2g084802_t01 0

grmzm2g084806_t02 0

grmzm2g084812_t01 0

grmzm2g084819_t01 0

grmzm2g084859_t01 0

grmzm2g084863_t01 0

grmzm2g084868_t01 0

grmzm2g084881_t02 0

grmzm2g084928_t02 0

grmzm2g084935_t01 0

grmzm2g084940_t01 0

grmzm2g084942_t01 0

grmzm2g084944_t01 0

grmzm2g084958_t04 -1.75

grmzm2g084984_t01 0

grmzm2g085000_t04 0

grmzm2g085005_t04 0

grmzm2g085019_t01 0

grmzm2g085027_t01 0

grmzm2g085038_t01 0

grmzm2g085042_t01 0

grmzm2g085049_t01 0

grmzm2g085054_t01 0

grmzm2g085078_t01 0

grmzm2g085086_t01 -1.42

grmzm2g085089_t01 0

grmzm2g085111_t01 0

grmzm2g085116_t01 0

grmzm2g085117_t01 0

grmzm2g085153_t02 0

grmzm2g085189_t01 0

grmzm2g085195_t01 0

grmzm2g085198_t01 0

grmzm2g085236_t03 0

grmzm2g085246_t01 0

grmzm2g085248_t01 0

grmzm2g085249_t01 0

grmzm2g085254_t03 0

grmzm2g085266_t01 0

grmzm2g085301_t01 0

grmzm2g085320_t01 0

grmzm2g085336_t01 0

grmzm2g085353_t01 0

grmzm2g085378_t01 0

grmzm2g085381_t03 -0.85

grmzm2g085392_t01 0

grmzm2g085402_t01 0

grmzm2g085411_t01 0

grmzm2g085438_t03 0

grmzm2g085467_t01 0

grmzm2g085469_t01 0

grmzm2g085474_t03 0.81

grmzm2g085483_t01 0

grmzm2g085513_t01 0

grmzm2g085530_t01 0

grmzm2g085547_t01 0.84

grmzm2g085550_t02 0

grmzm2g085568_t01 0

grmzm2g085573_t02 0

grmzm2g085577_t01 0

grmzm2g085582_t01 0

grmzm2g085587_t01 0

grmzm2g085600_t01 0

grmzm2g085602_t01 0

grmzm2g085604_t02 0

grmzm2g085613_t01 0.89

grmzm2g085630_t01 0

grmzm2g085641_t01 0

grmzm2g085646_t02 0

grmzm2g085661_t02 0

grmzm2g085670_t02 0

grmzm2g085675_t01 0

grmzm2g085678_t01 0

grmzm2g085711_t01 0

grmzm2g085713_t01 0

grmzm2g085718_t01 0

grmzm2g085733_t01 0

grmzm2g085747_t04 0

grmzm2g085750_t01 0

grmzm2g085812_t01 0

grmzm2g085825_t01 0

grmzm2g085827_t02 0

grmzm2g085833_t01 0

grmzm2g085845_t01 0

grmzm2g085849_t02 0

grmzm2g085854_t01 0

grmzm2g085856_t01 0

grmzm2g085872_t01 0

grmzm2g085885_t01 0

grmzm2g085909_t01 0

grmzm2g085924_t01 0

grmzm2g085926_t04 0

grmzm2g085932_t02 0

grmzm2g085939_t01 0

grmzm2g085945_t01 0

grmzm2g085948_t01 0

grmzm2g085960_t02 0

grmzm2g085967_t01 0

grmzm2g085970_t01 0

grmzm2g086003_t01 0

grmzm2g086030_t01 0

grmzm2g086032_t01 0

grmzm2g086052_t02 0

grmzm2g086069_t01 0

grmzm2g086072_t01 0

grmzm2g086088_t03 0

grmzm2g086093_t03 0

grmzm2g086116_t01 0

grmzm2g086123_t01 0

grmzm2g086132_t01 0

grmzm2g086138_t01 0

grmzm2g086163_t01 0

grmzm2g086202_t01 0

grmzm2g086236_t02 0

grmzm2g086237_t01 0

grmzm2g086242_t01 0

grmzm2g086258_t01 0

grmzm2g086267_t03 0

grmzm2g086269_t01 0

grmzm2g086277_t01 0

grmzm2g086287_t01 0

grmzm2g086371_t02 0

grmzm2g086389_t02 0

grmzm2g086403_t01 0

grmzm2g086418_t03 0

grmzm2g086464_t01 0

grmzm2g086484_t01 0

grmzm2g086486_t01 0

grmzm2g086489_t01 0

grmzm2g086496_t01 0

grmzm2g086497_t01 0

grmzm2g086553_t01 0

grmzm2g086577_t01 0

grmzm2g086587_t01 -1.38

grmzm2g086604_t01 0

grmzm2g086636_t01 0

grmzm2g086648_t02 0

grmzm2g086669_t02 0

grmzm2g086714_t01 0

grmzm2g086733_t01 0

grmzm2g086750_t02 0

grmzm2g086757_t01 0

grmzm2g086763_t03 0

grmzm2g086766_t01 0

grmzm2g086779_t01 0

grmzm2g086788_t01 0

grmzm2g086789_t01 0

grmzm2g086801_t02 0

grmzm2g086805_t02 0

grmzm2g086835_t01 0

grmzm2g086841_t01 0

grmzm2g086845_t03 0

grmzm2g086869_t01 0

grmzm2g086876_t01 0

grmzm2g086882_t01 0

grmzm2g086887_t02 0

grmzm2g086904_t01 0

grmzm2g086906_t01 0

grmzm2g086920_t01 0

grmzm2g086925_t01 0

grmzm2g086943_t02 0

grmzm2g086964_t01 0

grmzm2g086992_t01 0

grmzm2g086994_t01 0

grmzm2g086997_t01 0

grmzm2g087032_t01 0

grmzm2g087041_t01 0

grmzm2g087063_t06 0

grmzm2g087068_t01 0

grmzm2g087069_t01 0

grmzm2g087079_t01 0

grmzm2g087094_t01 0

grmzm2g087103_t03 0

grmzm2g087105_t01 0

grmzm2g087117_t01 0

grmzm2g087137_t01 0

grmzm2g087144_t01 0

grmzm2g087146_t01 0

grmzm2g087150_t01 0

grmzm2g087161_t01 0

grmzm2g087172_t01 0

grmzm2g087192_t01 0

grmzm2g087196_t01 0

grmzm2g087201_t01 0.9

grmzm2g087206_t01 0

grmzm2g087207_t01 0

grmzm2g087212_t01 0

grmzm2g087226_t04 0

grmzm2g087233_t02 0

grmzm2g087243_t01 0

grmzm2g087245_t01 -1.25

grmzm2g087254_t01 -1.53

grmzm2g087259_t01 1.08

grmzm2g087275_t01 0

grmzm2g087291_t02 0

grmzm2g087312_t01 0

grmzm2g087323_t01 0

grmzm2g087326_t01 0

grmzm2g087395_t02 0

grmzm2g087431_t04 0

grmzm2g087435_t01 0

grmzm2g087451_t01 0

grmzm2g087459_t04 0

grmzm2g087479_t01 0

grmzm2g087495_t01 0

grmzm2g087507_t01 -1.55

grmzm2g087513_t01 0

grmzm2g087531_t01 0

grmzm2g087549_t01 0

grmzm2g087570_t01 0

grmzm2g087582_t01 0

grmzm2g087590_t05 0

grmzm2g087598_t01 0

grmzm2g087600_t01 0

grmzm2g087612_t01 0

grmzm2g087622_t01 0

grmzm2g087625_t01 0

grmzm2g087628_t02 0

grmzm2g087635_t01 0

grmzm2g087651_t01 0

grmzm2g087662_t02 0

grmzm2g087671_t01 0

grmzm2g087675_t01 0

grmzm2g087678_t01 0

grmzm2g087692_t01 0

grmzm2g087712_t02 0.94

grmzm2g087714_t01 0

grmzm2g087719_t01 0

grmzm2g087758_t03 0

grmzm2g087787_t01 0

grmzm2g087804_t01 0

grmzm2g087806_t01 0

grmzm2g087817_t02 0

grmzm2g087850_t01 0

grmzm2g087851_t01 0

grmzm2g087870_t01 0

grmzm2g087896_t03 0

grmzm2g087901_t01 0

grmzm2g087918_t01 0

grmzm2g087920_t02 0

grmzm2g087924_t02 0

grmzm2g087955_t01 0

grmzm2g088053_t01 0

grmzm2g088060_t01 0

grmzm2g088083_t01 0

grmzm2g088088_t02 0

grmzm2g088114_t02 0

grmzm2g088138_t05 0

grmzm2g088196_t01 0

grmzm2g088212_t01 0

grmzm2g088218_t03 0

grmzm2g088225_t01 0

grmzm2g088235_t01 0

grmzm2g088261_t01 0

grmzm2g088299_t02 0

grmzm2g088320_t01 0

grmzm2g088356_t01 0

grmzm2g088361_t01 0

grmzm2g088396_t01 0

grmzm2g088397_t01 0

grmzm2g088436_t01 0.96

grmzm2g088469_t01 0

grmzm2g088482_t03 0

grmzm2g088487_t01 0

grmzm2g088501_t02 0

grmzm2g088511_t01 0

grmzm2g088524_t01 0

grmzm2g088539_t01 0

grmzm2g088549_t01 0

grmzm2g088565_t02 0

grmzm2g088590_t02 0

grmzm2g088601_t03 1.11

grmzm2g088627_t01 0

grmzm2g088648_t01 0

grmzm2g088653_t04 0

grmzm2g088669_t01 0

grmzm2g088689_t01 0

grmzm2g088727_t01 0

grmzm2g088737_t01 0

grmzm2g088753_t01 0

grmzm2g088828_t01 0

grmzm2g088834_t03 0

grmzm2g088843_t02 0

grmzm2g088849_t01 0

grmzm2g088880_t03 0

grmzm2g088921_t01 0

grmzm2g088930_t03 0

grmzm2g088951_t01 0

grmzm2g088961_t01 0

grmzm2g088974_t01 0

grmzm2g089010_t01 0

grmzm2g089021_t01 0

grmzm2g089050_t01 0

grmzm2g089056_t01 0

grmzm2g089058_t01 -1.92

grmzm2g089075_t01 0

grmzm2g089092_t02 0

grmzm2g089107_t01 0

grmzm2g089119_t01 0

grmzm2g089121_t01 0

grmzm2g089132_t01 0

grmzm2g089136_t01 0

grmzm2g089140_t01 0

grmzm2g089147_t01 0

grmzm2g089149_t01 0

grmzm2g089159_t02 0

grmzm2g089163_t01 0

grmzm2g089193_t01 0

grmzm2g089244_t01 2.1

grmzm2g089259_t03 0

grmzm2g089282_t01 0

grmzm2g089296_t02 0

grmzm2g089317_t03 0

grmzm2g089351_t02 0

grmzm2g089355_t02 0

grmzm2g089361_t01 0

grmzm2g089365_t01 0

grmzm2g089400_t01 1.27

grmzm2g089406_t01 0

grmzm2g089421_t02 0

grmzm2g089448_t01 0

grmzm2g089454_t01 0

grmzm2g089461_t01 0

grmzm2g089466_t01 0

grmzm2g089484_t01 0

grmzm2g089491_t03 0

grmzm2g089493_t01 -1.34

grmzm2g089498_t05 0

grmzm2g089501_t01 0.81

grmzm2g089507_t01 0.99

grmzm2g089528_t01 -0.81

grmzm2g089562_t02 0

grmzm2g089565_t01 0

grmzm2g089596_t06 -1.15

grmzm2g089622_t01 0

grmzm2g089630_t02 0

grmzm2g089631_t05 0

grmzm2g089640_t01 0

grmzm2g089673_t01 0

grmzm2g089676_t01 0

grmzm2g089684_t01 0

grmzm2g089688_t01 0

grmzm2g089696_t01 0

grmzm2g089713_t04 0

grmzm2g089736_t02 0

grmzm2g089783_t01 0

grmzm2g089812_t02 0

grmzm2g089836_t02 0

grmzm2g089850_t01 -1.29

grmzm2g089854_t03 0

grmzm2g089857_t01 0.86

grmzm2g089860_t01 0

grmzm2g089876_t01 1.88

grmzm2g089895_t01 0

grmzm2g089944_t01 0

grmzm2g089948_t01 0

grmzm2g089952_t01 -1.17

grmzm2g089959_t01 0

grmzm2g089962_t01 0

grmzm2g089974_t01 0

grmzm2g089976_t01 0

grmzm2g089992_t01 0

grmzm2g089995_t01 0

grmzm2g090010_t01 0

grmzm2g090018_t01 0

grmzm2g090028_t01 0

grmzm2g090029_t01 0

grmzm2g090034_t01 0

grmzm2g090037_t05 0

grmzm2g090061_t01 0

grmzm2g090068_t01 0

grmzm2g090070_t02 0

grmzm2g090086_t01 0

grmzm2g090087_t04 0

grmzm2g090104_t02 0

grmzm2g090112_t01 0

grmzm2g090124_t01 0

grmzm2g090156_t01 0

grmzm2g090172_t02 0

grmzm2g090177_t01 0

grmzm2g090187_t01 0

grmzm2g090190_t01 0

grmzm2g090213_t02 0

grmzm2g090217_t03 0

grmzm2g090226_t01 0

grmzm2g090241_t01 0

grmzm2g090271_t01 0

grmzm2g090274_t01 0.88

grmzm2g090279_t02 0

grmzm2g090300_t01 0

grmzm2g090338_t01 0

grmzm2g090374_t01 0

grmzm2g090419_t01 -1.09

grmzm2g090422_t02 0

grmzm2g090432_t01 0

grmzm2g090435_t01 -0.81

grmzm2g090480_t05 0

grmzm2g090487_t01 0

grmzm2g090493_t01 0

grmzm2g090500_t01 0

grmzm2g090505_t01 0

grmzm2g090542_t01 0

grmzm2g090559_t02 0

grmzm2g090568_t01 0

grmzm2g090576_t01 0

grmzm2g090584_t01 0

grmzm2g090594_t01 0

grmzm2g090595_t01 0

grmzm2g090609_t02 0

grmzm2g090619_t01 0

grmzm2g090647_t04 0

grmzm2g090675_t01 0

grmzm2g090718_t01 0

grmzm2g090722_t03 0

grmzm2g090728_t01 0

grmzm2g090733_t02 0

grmzm2g090736_t01 0

grmzm2g090738_t01 0

grmzm2g090747_t01 0

grmzm2g090779_t02 0

grmzm2g090824_t04 0

grmzm2g090865_t01 -1.88

grmzm2g090868_t03 0

grmzm2g090869_t01 0

grmzm2g090873_t02 0

grmzm2g090887_t01 0

grmzm2g090904_t01 0

grmzm2g090905_t01 0

grmzm2g090908_t01 0

grmzm2g090928_t05 0

grmzm2g090935_t02 0

grmzm2g090963_t02 0

grmzm2g090980_t02 0

grmzm2g090981_t01 0

grmzm2g091006_t02 1.49

grmzm2g091047_t01 0

grmzm2g091058_t01 0

grmzm2g091119_t01 0

grmzm2g091124_t01 0

grmzm2g091143_t02 0

grmzm2g091151_t01 0

grmzm2g091155_t04 0

grmzm2g091189_t03 0

grmzm2g091226_t01 -1.41

grmzm2g091228_t01 0

grmzm2g091232_t01 0

grmzm2g091233_t02 0

grmzm2g091245_t01 0

grmzm2g091258_t06 0

grmzm2g091265_t06 0

grmzm2g091267_t01 0.9

grmzm2g091276_t02 0

grmzm2g091293_t01 0

grmzm2g091302_t03 0

grmzm2g091331_t03 0

grmzm2g091362_t01 0

grmzm2g091383_t01 0

grmzm2g091397_t02 0

grmzm2g091419_t01 0

grmzm2g091433_t02 0

grmzm2g091435_t01 0

grmzm2g091449_t01 0

grmzm2g091456_t04 0

grmzm2g091481_t02 0

grmzm2g091487_t01 0

grmzm2g091494_t02 -1.02

grmzm2g091503_t03 0

grmzm2g091540_t02 0

grmzm2g091558_t04 0

grmzm2g091560_t02 0

grmzm2g091563_t01 0

grmzm2g091578_t01 0

grmzm2g091581_t03 0

grmzm2g091586_t04 0

grmzm2g091632_t03 0

grmzm2g091696_t01 0

grmzm2g091715_t03 0

grmzm2g091756_t01 0

grmzm2g091766_t01 0

grmzm2g091811_t01 0

grmzm2g091825_t01 0

grmzm2g091839_t01 -1.11

grmzm2g091845_t03 0

grmzm2g091857_t01 0

grmzm2g091895_t01 0

grmzm2g091900_t01 0

grmzm2g091921_t01 0

grmzm2g091964_t03 0

grmzm2g091973_t01 0

grmzm2g091989_t01 1.18

grmzm2g091995_t02 0

grmzm2g092000_t01 0

grmzm2g092018_t01 0

grmzm2g092021_t01 0

grmzm2g092051_t01 0

grmzm2g092085_t01 0

grmzm2g092101_t01 0

grmzm2g092107_t01 0

grmzm2g092109_t01 0

grmzm2g092112_t01 0

grmzm2g092120_t01 0

grmzm2g092123_t01 0

grmzm2g092125_t02 0

grmzm2g092129_t01 0

grmzm2g092131_t01 0

grmzm2g092137_t01 0.83

grmzm2g092147_t02 1.01

grmzm2g092152_t01 0

grmzm2g092154_t01 0

grmzm2g092160_t02 0

grmzm2g092165_t02 0

grmzm2g092174_t01 0

grmzm2g092182_t01 0

grmzm2g092190_t02 0

grmzm2g092198_t01 0

grmzm2g092214_t01 0

grmzm2g092244_t01 0

grmzm2g092256_t01 0

grmzm2g092258_t05 0

grmzm2g092281_t01 0

grmzm2g092284_t03 0

grmzm2g092296_t02 0

grmzm2g092311_t03 0

grmzm2g092325_t02 0

grmzm2g092327_t01 0

grmzm2g092365_t02 0

grmzm2g092371_t01 0

grmzm2g092379_t01 0

grmzm2g092409_t01 0

grmzm2g092427_t03 0

grmzm2g092429_t05 0

grmzm2g092433_t01 0

grmzm2g092447_t01 0

grmzm2g092459_t02 0

grmzm2g092468_t01 0

grmzm2g092474_t01 0

grmzm2g092481_t02 0

grmzm2g092493_t01 0

grmzm2g092497_t01 0

grmzm2g092535_t01 0

grmzm2g092545_t04 0

grmzm2g092571_t04 0

grmzm2g092581_t02 -1.23

grmzm2g092586_t01 0

grmzm2g092588_t03 0

grmzm2g092595_t01 0.96

grmzm2g092604_t01 0

grmzm2g092607_t01 0

grmzm2g092609_t01 0

grmzm2g092616_t01 0

grmzm2g092632_t01 0

grmzm2g092648_t06 0

grmzm2g092661_t01 0

grmzm2g092663_t02 0

grmzm2g092669_t01 0

grmzm2g092678_t01 0

grmzm2g092719_t01 0

grmzm2g092723_t01 0

grmzm2g092739_t01 0

grmzm2g092741_t02 0

grmzm2g092743_t01 0

grmzm2g092749_t02 0

grmzm2g092776_t08 0

grmzm2g092780_t01 0

grmzm2g092793_t01 0

grmzm2g092813_t01 -1.15

grmzm2g092817_t01 0

grmzm2g092834_t01 0

grmzm2g092888_t01 0

grmzm2g092895_t01 0

grmzm2g092910_t01 0

grmzm2g092923_t02 0

grmzm2g092947_t01 0

grmzm2g092959_t01 0

grmzm2g092975_t01 0

grmzm2g092981_t01 0.82

grmzm2g092988_t01 0

grmzm2g092991_t02 0

grmzm2g093006_t01 0

grmzm2g093020_t01 0

grmzm2g093032_t01 0

grmzm2g093038_t02 0

grmzm2g093050_t01 0

grmzm2g093065_t01 0

grmzm2g093101_t01 0

grmzm2g093104_t02 0

grmzm2g093119_t03 0

grmzm2g093125_t01 0

grmzm2g093139_t01 0

grmzm2g093146_t01 0

grmzm2g093186_t01 0

grmzm2g093195_t01 0

grmzm2g093197_t01 0

grmzm2g093210_t01 -0.97

grmzm2g093254_t02 0

grmzm2g093256_t06 0

grmzm2g093270_t01 0

grmzm2g093272_t01 0

grmzm2g093276_t01 0

grmzm2g093286_t01 0

grmzm2g093288_t01 0

grmzm2g093291_t01 0

grmzm2g093305_t01 0

grmzm2g093316_t01 0

grmzm2g093323_t01 0

grmzm2g093325_t03 0

grmzm2g093347_t01 0

grmzm2g093359_t01 0

grmzm2g093391_t01 0

grmzm2g093399_t01 0

grmzm2g093405_t02 0

grmzm2g093436_t01 0

grmzm2g093441_t02 0

grmzm2g093490_t01 0

grmzm2g093514_t02 0

grmzm2g093526_t01 0.89

grmzm2g093557_t01 0

grmzm2g093574_t03 1.07

grmzm2g093598_t01 0

grmzm2g093603_t01 0

grmzm2g093623_t01 0

grmzm2g093638_t01 0

grmzm2g093643_t01 0

grmzm2g093666_t02 0

grmzm2g093708_t01 0

grmzm2g093710_t01 0

grmzm2g093716_t01 0

grmzm2g093720_t01 0

grmzm2g093731_t03 0

grmzm2g093736_t01 0

grmzm2g093744_t02 0

grmzm2g093761_t04 0

grmzm2g093776_t01 0

grmzm2g093789_t01 1.04

grmzm2g093809_t01 0

grmzm2g093815_t02 0

grmzm2g093826_t02 0

grmzm2g093828_t01 0

grmzm2g093849_t01 -1.43

grmzm2g093855_t02 0

grmzm2g093895_t01 0

grmzm2g093900_t01 0

grmzm2g093902_t04 0.9

grmzm2g093908_t01 0

grmzm2g093945_t01 0

grmzm2g093950_t01 0

grmzm2g093962_t05 0

grmzm2g093997_t02 0

grmzm2g094017_t01 0

grmzm2g094039_t04 0

grmzm2g094047_t01 0

grmzm2g094050_t01 0

grmzm2g094051_t01 0

grmzm2g094072_t02 0

grmzm2g094074_t03 0

grmzm2g094083_t07 1.99

grmzm2g094090_t01 0

grmzm2g094165_t02 0

grmzm2g094168_t01 0

grmzm2g094224_t01 0

grmzm2g094255_t01 0

grmzm2g094304_t01 -1.53

grmzm2g094352_t03 -1.82

grmzm2g094388_t01 0

grmzm2g094390_t01 0

grmzm2g094428_t01 0

grmzm2g094452_t01 0

grmzm2g094490_t01 0

grmzm2g094497_t01 0

grmzm2g094500_t04 0

grmzm2g094526_t02 0

grmzm2g094532_t01 0

grmzm2g094541_t01 0

grmzm2g094543_t01 0

grmzm2g094558_t01 0

grmzm2g094579_t01 0

grmzm2g094581_t01 0

grmzm2g094586_t01 0

grmzm2g094589_t01 0

grmzm2g094595_t02 0

grmzm2g094599_t01 0

grmzm2g094616_t01 0

grmzm2g094632_t02 2.08

grmzm2g094655_t02 0

grmzm2g094698_t01 0.92

grmzm2g094699_t02 0

grmzm2g094712_t04 0

grmzm2g094742_t01 0

grmzm2g094792_t01 0

grmzm2g094831_t01 0

grmzm2g094867_t01 0

grmzm2g094871_t01 0

grmzm2g094879_t02 0

grmzm2g094884_t02 0

grmzm2g094892_t01 0

grmzm2g094898_t01 0

grmzm2g094928_t03 0

grmzm2g094951_t02 0

grmzm2g094959_t05 0

grmzm2g094978_t02 0

grmzm2g094990_t01 1.5

grmzm2g095039_t01 0

grmzm2g095043_t01 0

grmzm2g095082_t02 0

grmzm2g095104_t02 0

grmzm2g095114_t02 0

grmzm2g095124_t01 0

grmzm2g095126_t03 0

grmzm2g095141_t05 0

grmzm2g095144_t01 0

grmzm2g095147_t02 0

grmzm2g095164_t01 0

grmzm2g095185_t01 0

grmzm2g095206_t01 0

grmzm2g095211_t01 0

grmzm2g095219_t01 0

grmzm2g095239_t02 0

grmzm2g095287_t01 0

grmzm2g095299_t01 0

grmzm2g095302_t01 0

grmzm2g095308_t01 0

grmzm2g095323_t01 0

grmzm2g095326_t01 0

grmzm2g095333_t01 0

grmzm2g095348_t03 0

grmzm2g095363_t01 4.4

grmzm2g095392_t02 0

grmzm2g095400_t01 0

grmzm2g095404_t01 0

grmzm2g095492_t01 0

grmzm2g095510_t02 0

grmzm2g095511_t01 0

grmzm2g095534_t06 1.14

grmzm2g095552_t03 0

grmzm2g095556_t01 0

grmzm2g095562_t01 0

grmzm2g095579_t02 0

grmzm2g095588_t01 0

grmzm2g095595_t02 0

grmzm2g095598_t01 0

grmzm2g095611_t02 0

grmzm2g095631_t01 0

grmzm2g095634_t01 0

grmzm2g095655_t01 0

grmzm2g095657_t01 0

grmzm2g095670_t02 0

grmzm2g095725_t03 -1.57

grmzm2g095727_t03 0

grmzm2g095763_t01 0

grmzm2g095778_t02 0

grmzm2g095782_t04 0

grmzm2g095786_t01 -2.7

grmzm2g095806_t01 0

grmzm2g095807_t01 0

grmzm2g095810_t01 0

grmzm2g095811_t04 -1.41

grmzm2g095826_t01 0

grmzm2g095865_t03 0

grmzm2g095868_t04 0

grmzm2g095892_t02 0

grmzm2g095898_t02 0

grmzm2g095905_t01 0

grmzm2g095921_t01 0.87

grmzm2g095931_t01 0

grmzm2g095945_t02 0

grmzm2g095964_t01 0

grmzm2g095968_t02 -0.9

grmzm2g095969_t02 0

grmzm2g096010_t01 0

grmzm2g096020_t01 0

grmzm2g096037_t02 0

grmzm2g096051_t04 0

grmzm2g096070_t01 0

grmzm2g096090_t01 0

grmzm2g096092_t01 0

grmzm2g096107_t01 0

grmzm2g096153_t02 0

grmzm2g096165_t01 0

grmzm2g096169_t01 0

grmzm2g096171_t02 0

grmzm2g096228_t02 0

grmzm2g096240_t02 0

grmzm2g096247_t01 0

grmzm2g096261_t02 0

grmzm2g096269_t01 -1.11

grmzm2g096303_t01 0

grmzm2g096348_t03 0

grmzm2g096352_t03 0

grmzm2g096355_t01 0

grmzm2g096363_t01 0

grmzm2g096365_t01 0

grmzm2g096370_t01 0

grmzm2g096389_t02 0

grmzm2g096391_t01 0

grmzm2g096407_t01 0

grmzm2g096409_t01 0

grmzm2g096412_t01 0

grmzm2g096470_t04 0

grmzm2g096486_t01 0

grmzm2g096487_t02 0

grmzm2g096491_t03 0

grmzm2g096546_t01 0

grmzm2g096548_t02 0

grmzm2g096585_t06 0

grmzm2g096591_t02 0

grmzm2g096596_t02 0

grmzm2g096602_t01 0

grmzm2g096604_t03 0

grmzm2g096622_t01 0

grmzm2g096655_t01 0

grmzm2g096663_t01 0

grmzm2g096682_t01 0

grmzm2g096683_t01 0

grmzm2g096690_t01 0

grmzm2g096691_t01 -0.88

grmzm2g096693_t01 0

grmzm2g096695_t02 0

grmzm2g096705_t01 0

grmzm2g096709_t01 0

grmzm2g096764_t01 0

grmzm2g096802_t01 0

grmzm2g096806_t01 0

grmzm2g096814_t02 0

grmzm2g096815_t01 0

grmzm2g096824_t01 0

grmzm2g096877_t02 0

grmzm2g096904_t01 0

grmzm2g096909_t01 0

grmzm2g096920_t01 0

grmzm2g096946_t01 0

grmzm2g096958_t01 0

grmzm2g096962_t03 0

grmzm2g096972_t01 0

grmzm2g097015_t01 0

grmzm2g097021_t01 0

grmzm2g097032_t02 0

grmzm2g097040_t01 0

grmzm2g097043_t02 0

grmzm2g097046_t01 -0.92

grmzm2g097068_t01 0

grmzm2g097103_t02 0

grmzm2g097106_t01 0

grmzm2g097109_t01 0

grmzm2g097129_t01 0

grmzm2g097137_t01 0

grmzm2g097141_t01 0

grmzm2g097144_t01 0

grmzm2g097164_t01 0

grmzm2g097207_t01 0

grmzm2g097226_t01 0

grmzm2g097249_t01 0

grmzm2g097258_t01 0

grmzm2g097275_t01 0

grmzm2g097277_t01 0

grmzm2g097282_t01 0

grmzm2g097283_t02 0

grmzm2g097289_t02 0

grmzm2g097297_t01 2.97

grmzm2g097349_t01 0

grmzm2g097364_t03 0

grmzm2g097395_t01 0

grmzm2g097421_t02 0

grmzm2g097426_t03 0

grmzm2g097434_t02 0

grmzm2g097457_t03 0

grmzm2g097499_t01 0

grmzm2g097502_t02 0

grmzm2g097504_t01 0

grmzm2g097568_t05 0

grmzm2g097573_t01 0

grmzm2g097593_t01 0

grmzm2g097605_t01 0

grmzm2g097640_t02 0

grmzm2g097641_t02 0

grmzm2g097662_t01 0

grmzm2g097674_t03 0

grmzm2g097704_t01 0

grmzm2g097726_t01 0

grmzm2g097728_t03 0.83

grmzm2g097738_t01 0

grmzm2g097739_t01 0

grmzm2g097746_t02 0

grmzm2g097747_t01 0

grmzm2g097802_t01 0

grmzm2g097805_t01 0

grmzm2g097812_t01 0

grmzm2g097820_t03 0

grmzm2g097821_t01 0

grmzm2g097827_t01 -1.86

grmzm2g097841_t01 0

grmzm2g097843_t01 0

grmzm2g097851_t01 0

grmzm2g097854_t02 0

grmzm2g097896_t01 0

grmzm2g097898_t03 0

grmzm2g097900_t01 0

grmzm2g097935_t01 0

grmzm2g097959_t01 0

grmzm2g097967_t01 0

grmzm2g097977_t01 0

grmzm2g097995_t01 0

grmzm2g098011_t01 0

grmzm2g098015_t01 0

grmzm2g098039_t01 0

grmzm2g098046_t01 0

grmzm2g098058_t02 -1.16

grmzm2g098076_t02 0

grmzm2g098078_t02 0

grmzm2g098079_t01 0

grmzm2g098084_t06 0

grmzm2g098088_t01 0

grmzm2g098129_t01 0

grmzm2g098153_t02 0

grmzm2g098174_t01 0

grmzm2g098179_t01 0

grmzm2g098182_t01 0

grmzm2g098187_t01 0

grmzm2g098208_t02 0

grmzm2g098212_t01 0

grmzm2g098214_t01 0

grmzm2g098227_t01 0

grmzm2g098237_t01 0

grmzm2g098239_t01 0

grmzm2g098279_t01 0

grmzm2g098290_t04 0

grmzm2g098298_t01 0

grmzm2g098301_t01 0

grmzm2g098305_t01 0

grmzm2g098335_t01 0

grmzm2g098346_t01 0

grmzm2g098370_t02 0

grmzm2g098397_t03 0

grmzm2g098420_t04 0

grmzm2g098423_t01 0

grmzm2g098427_t04 0

grmzm2g098434_t01 0

grmzm2g098442_t01 -2.04

grmzm2g098460_t01 0

grmzm2g098494_t01 0

grmzm2g098496_t01 0

grmzm2g098517_t01 0

grmzm2g098520_t01 0

grmzm2g098527_t01 0

grmzm2g098545_t03 0

grmzm2g098557_t01 0

grmzm2g098565_t05 0

grmzm2g098569_t01 0

grmzm2g098577_t04 0

grmzm2g098594_t04 0

grmzm2g098603_t01 0

grmzm2g098637_t02 0

grmzm2g098643_t01 0

grmzm2g098674_t01 0

grmzm2g098676_t01 0

grmzm2g098679_t01 1.06

grmzm2g098697_t02 1.6

grmzm2g098712_t01 0

grmzm2g098721_t03 0

grmzm2g098731_t01 0

grmzm2g098751_t01 -1.14

grmzm2g098754_t01 0

grmzm2g098784_t01 0

grmzm2g098793_t02 0

grmzm2g098797_t01 0

grmzm2g098800_t02 0

grmzm2g098815_t03 0

grmzm2g098819_t01 0

grmzm2g098857_t04 0

grmzm2g098859_t01 0

grmzm2g098869_t01 0

grmzm2g098884_t01 0

grmzm2g098890_t01 0

grmzm2g098900_t02 0

grmzm2g098904_t02 0

grmzm2g098957_t01 0

grmzm2g098973_t02 0

grmzm2g098988_t01 0

grmzm2g098999_t01 0

grmzm2g099002_t02 0

grmzm2g099005_t01 0

grmzm2g099007_t01 0

grmzm2g099034_t01 0

grmzm2g099036_t01 0

grmzm2g099045_t01 0

grmzm2g099049_t01 -1.11

grmzm2g099052_t01 0

grmzm2g099056_t01 0

grmzm2g099066_t01 0

grmzm2g099080_t02 0

grmzm2g099086_t01 0

grmzm2g099088_t01 0

grmzm2g099130_t01 0

grmzm2g099160_t03 0

grmzm2g099167_t02 0

grmzm2g099183_t03 0

grmzm2g099186_t01 0

grmzm2g099187_t01 0

grmzm2g099191_t02 0

grmzm2g099207_t01 0

grmzm2g099238_t02 0

grmzm2g099239_t02 0

grmzm2g099253_t01 0

grmzm2g099255_t01 0

grmzm2g099270_t01 0

grmzm2g099285_t01 0

grmzm2g099295_t01 -1.22

grmzm2g099305_t02 1.32

grmzm2g099317_t03 0

grmzm2g099319_t01 0

grmzm2g099324_t01 0

grmzm2g099328_t04 0

grmzm2g099334_t01 0

grmzm2g099337_t01 0

grmzm2g099352_t05 0

grmzm2g099355_t01 0

grmzm2g099363_t03 0

grmzm2g099367_t01 0

grmzm2g099382_t02 0

grmzm2g099390_t01 0

grmzm2g099425_t01 0

grmzm2g099449_t01 0

grmzm2g099452_t01 0

grmzm2g099454_t01 0

grmzm2g099463_t02 0

grmzm2g099466_t01 0

grmzm2g099467_t01 0

grmzm2g099474_t01 0

grmzm2g099481_t01 0

grmzm2g099483_t01 -0.86

grmzm2g099487_t01 0

grmzm2g099491_t01 0

grmzm2g099529_t01 0

grmzm2g099542_t01 1.08

grmzm2g099547_t04 0

grmzm2g099598_t02 0

grmzm2g099604_t01 0

grmzm2g099615_t01 0

grmzm2g099626_t01 0

grmzm2g099628_t03 0

grmzm2g099630_t03 0

grmzm2g099642_t01 0

grmzm2g099648_t01 0

grmzm2g099657_t05 0

grmzm2g099666_t01 0

grmzm2g099678_t01 -1.02

grmzm2g099696_t01 0

grmzm2g099729_t02 0

grmzm2g099754_t04 0

grmzm2g099758_t01 0

grmzm2g099767_t02 0

grmzm2g099780_t01 0

grmzm2g099797_t03 0

grmzm2g099860_t03 0

grmzm2g099862_t02 0

grmzm2g099875_t01 0

grmzm2g099891_t03 0

grmzm2g099907_t01 0

grmzm2g099909_t01 0

grmzm2g099914_t01 0.83

grmzm2g099944_t02 0

grmzm2g099960_t01 0

grmzm2g099967_t01 0

grmzm2g099980_t02 0

grmzm2g099981_t02 0

grmzm2g099984_t01 0

grmzm2g099987_t01 0

grmzm2g100020_t02 0

grmzm2g100043_t01 0

grmzm2g100084_t01 0

grmzm2g100090_t01 0

grmzm2g100107_t02 0

grmzm2g100118_t01 0

grmzm2g100120_t05 0

grmzm2g100143_t01 0

grmzm2g100146_t02 0

grmzm2g100152_t01 0

grmzm2g100158_t01 0

grmzm2g100176_t01 0

grmzm2g100225_t01 0

grmzm2g100229_t01 0

grmzm2g100244_t01 0

grmzm2g100260_t03 0

grmzm2g100286_t02 2.56

grmzm2g100288_t01 0

grmzm2g100315_t01 0

grmzm2g100318_t01 0

grmzm2g100321_t01 0

grmzm2g100333_t01 0

grmzm2g100344_t01 0

grmzm2g100360_t01 0

grmzm2g100391_t01 -0.84

grmzm2g100402_t02 0

grmzm2g100419_t01 0

grmzm2g100431_t01 0

grmzm2g100448_t01 0

grmzm2g100454_t03 0

grmzm2g100462_t05 0

grmzm2g100467_t01 0

grmzm2g100484_t03 0

grmzm2g100497_t01 0

grmzm2g100511_t01 0

grmzm2g100518_t01 0

grmzm2g100568_t01 0

grmzm2g100579_t02 0

grmzm2g100583_t03 0

grmzm2g100620_t01 0

grmzm2g100641_t01 0

grmzm2g100652_t04 0

grmzm2g100707_t02 0

grmzm2g100714_t01 0

grmzm2g100716_t04 0

grmzm2g100726_t01 0

grmzm2g100741_t01 0

grmzm2g100747_t01 0

grmzm2g100754_t01 2.93

grmzm2g100794_t01 0

grmzm2g100809_t01 0

grmzm2g100815_t03 0

grmzm2g100858_t01 0

grmzm2g100864_t01 0

grmzm2g100872_t01 0

grmzm2g100881_t02 0

grmzm2g100889_t02 0

grmzm2g100898_t01 0

grmzm2g100920_t02 0

grmzm2g100946_t01 0

grmzm2g100955_t01 0

grmzm2g100976_t01 0

grmzm2g100988_t01 0

grmzm2g101004_t01 0

grmzm2g101020_t01 0

grmzm2g101024_t01 0

grmzm2g101042_t01 0

grmzm2g101053_t01 1.6

grmzm2g101058_t01 0

grmzm2g101062_t03 0

grmzm2g101076_t02 0

grmzm2g101105_t01 0

grmzm2g101116_t01 1.09

grmzm2g101117_t01 0

grmzm2g101125_t01 0

grmzm2g101130_t01 0

grmzm2g101142_t02 0

grmzm2g101179_t01 0

grmzm2g101181_t02 0

grmzm2g101217_t04 0

grmzm2g101244_t07 0

grmzm2g101264_t01 0

grmzm2g101270_t01 -1.36

grmzm2g101271_t01 0

grmzm2g101274_t02 0

grmzm2g101282_t01 0

grmzm2g101287_t01 0

grmzm2g101350_t01 0

grmzm2g101383_t01 0

grmzm2g101390_t02 0

grmzm2g101393_t01 0

grmzm2g101408_t02 0

grmzm2g101412_t01 0

grmzm2g101413_t02 0

grmzm2g101438_t05 0

grmzm2g101446_t01 0

grmzm2g101457_t01 0

grmzm2g101460_t02 0

grmzm2g101463_t02 0

grmzm2g101480_t06 0

grmzm2g101502_t03 0

grmzm2g101515_t02 0

grmzm2g101518_t01 0

grmzm2g101523_t02 0

grmzm2g101533_t01 1.05

grmzm2g101547_t03 0

grmzm2g101571_t01 0

grmzm2g101596_t01 0

grmzm2g101613_t01 0

grmzm2g101617_t01 0

grmzm2g101635_t01 0

grmzm2g101664_t01 0

grmzm2g101687_t01 0

grmzm2g101689_t01 0

grmzm2g101693_t02 0

grmzm2g101698_t01 0

grmzm2g101711_t01 0

grmzm2g101744_t02 0

grmzm2g101754_t01 0

grmzm2g101760_t01 0

grmzm2g101769_t01 0

grmzm2g101791_t01 0

grmzm2g101818_t01 0

grmzm2g101852_t02 0

grmzm2g101859_t01 0

grmzm2g101867_t01 0

grmzm2g101874_t01 0

grmzm2g101875_t03 1.6

grmzm2g101900_t01 0

grmzm2g101926_t02 0

grmzm2g101938_t02 0

grmzm2g101958_t01 0

grmzm2g101979_t01 0

grmzm2g102000_t01 0

grmzm2g102021_t02 0

grmzm2g102041_t01 0

grmzm2g102059_t01 0

grmzm2g102069_t01 0

grmzm2g102075_t01 0

grmzm2g102088_t01 0

grmzm2g102138_t01 -1.33

grmzm2g102163_t01 0

grmzm2g102167_t02 0

grmzm2g102174_t01 0

grmzm2g102183_t02 0

grmzm2g102189_t01 0

grmzm2g102196_t02 0

grmzm2g102216_t01 0

grmzm2g102230_t01 0

grmzm2g102231_t03 0

grmzm2g102238_t01 2.4

grmzm2g102243_t01 0

grmzm2g102255_t01 0

grmzm2g102271_t01 0

grmzm2g102322_t01 0

grmzm2g102346_t03 0

grmzm2g102347_t06 0

grmzm2g102349_t02 0

grmzm2g102356_t02 0

grmzm2g102365_t02 0

grmzm2g102404_t02 0

grmzm2g102421_t01 0

grmzm2g102447_t01 0

grmzm2g102471_t03 0

grmzm2g102475_t01 0

grmzm2g102497_t01 0

grmzm2g102499_t07 0

grmzm2g102502_t02 0

grmzm2g102514_t01 -1.79

grmzm2g102516_t03 0

grmzm2g102548_t01 0

grmzm2g102560_t01 0

grmzm2g102572_t03 0

grmzm2g102580_t02 0

grmzm2g102583_t03 0

grmzm2g102596_t01 0

grmzm2g102601_t01 0

grmzm2g102639_t02 0

grmzm2g102664_t01 0

grmzm2g102681_t01 0

grmzm2g102692_t01 0

grmzm2g102714_t02 0

grmzm2g102737_t01 0

grmzm2g102745_t01 0

grmzm2g102760_t01 0

grmzm2g102779_t01 0

grmzm2g102786_t02 0

grmzm2g102790_t01 0

grmzm2g102802_t04 0

grmzm2g102808_t01 -1.71

grmzm2g102829_t04 0

grmzm2g102838_t01 0

grmzm2g102845_t01 0

grmzm2g102858_t01 0

grmzm2g102862_t01 0

grmzm2g102878_t01 0

grmzm2g102891_t03 0

grmzm2g102903_t01 0

grmzm2g102912_t01 0

grmzm2g102915_t01 0

grmzm2g102921_t03 0

grmzm2g102923_t01 0

grmzm2g102938_t01 0

grmzm2g102946_t04 0

grmzm2g102959_t01 0

grmzm2g102964_t01 0

grmzm2g102968_t03 0

grmzm2g103013_t01 -3.46

grmzm2g103047_t01 0

grmzm2g103070_t02 0

grmzm2g103078_t01 0

grmzm2g103079_t02 0

grmzm2g103101_t01 0

grmzm2g103113_t01 0

grmzm2g103116_t01 0

grmzm2g103128_t01 0

grmzm2g103135_t01 0

grmzm2g103152_t01 0

grmzm2g103166_t02 0

grmzm2g103179_t01 0

grmzm2g103182_t01 0

grmzm2g103197_t01 0

grmzm2g103216_t01 0

grmzm2g103230_t02 0

grmzm2g103245_t02 0

grmzm2g103250_t01 0

grmzm2g103258_t07 0

grmzm2g103266_t07 0

grmzm2g103270_t01 0

grmzm2g103281_t01 0

grmzm2g103287_t01 0

grmzm2g103315_t01 0

grmzm2g103342_t01 -0.88

grmzm2g103345_t01 0

grmzm2g103357_t03 0

grmzm2g103382_t01 0

grmzm2g103430_t05 0

grmzm2g103458_t01 0

grmzm2g103465_t02 0

grmzm2g103490_t01 0

grmzm2g103512_t01 0

grmzm2g103526_t01 0

grmzm2g103544_t01 0

grmzm2g103579_t01 0

grmzm2g103617_t03 0

grmzm2g103628_t01 0

grmzm2g103647_t02 0

grmzm2g103657_t01 0

grmzm2g103662_t02 0

grmzm2g103672_t02 0

grmzm2g103713_t01 0

grmzm2g103721_t01 0

grmzm2g103740_t01 0

grmzm2g103773_t01 0

grmzm2g103783_t02 0

grmzm2g103785_t01 0

grmzm2g103805_t03 0

grmzm2g103812_t01 0

grmzm2g103843_t01 0

grmzm2g103847_t01 0

grmzm2g103864_t01 0

grmzm2g103873_t01 0

grmzm2g103884_t01 0

grmzm2g103896_t01 0

grmzm2g103897_t02 0

grmzm2g103900_t04 0

grmzm2g103902_t03 0

grmzm2g103909_t01 0

grmzm2g103914_t01 0

grmzm2g103929_t01 -1.03

grmzm2g103937_t05 0

grmzm2g103939_t02 0

grmzm2g103945_t03 -1.34

grmzm2g104012_t01 0

grmzm2g104013_t03 0

grmzm2g104017_t04 0

grmzm2g104025_t02 0

grmzm2g104041_t01 0

grmzm2g104045_t01 0

grmzm2g104047_t01 0

grmzm2g104070_t03 0

grmzm2g104081_t01 0

grmzm2g104088_t04 0

grmzm2g104118_t01 0

grmzm2g104125_t01 0

grmzm2g104133_t01 0

grmzm2g104147_t04 0

grmzm2g104166_t03 0

grmzm2g104168_t01 0

grmzm2g104179_t01 0

grmzm2g104226_t01 0.99

grmzm2g104231_t02 0

grmzm2g104237_t01 0

grmzm2g104254_t01 0

grmzm2g104262_t01 0

grmzm2g104269_t03 0

grmzm2g104288_t01 0

grmzm2g104294_t01 0

grmzm2g104299_t03 0

grmzm2g104310_t01 0

grmzm2g104316_t01 0

grmzm2g104325_t01 0

grmzm2g104347_t01 0

grmzm2g104357_t01 0.81

grmzm2g104373_t01 0

grmzm2g104375_t02 0

grmzm2g104384_t01 0

grmzm2g104390_t01 0

grmzm2g104394_t01 0

grmzm2g104397_t01 0

grmzm2g104400_t01 0

grmzm2g104410_t01 0

grmzm2g104419_t01 2.15

grmzm2g104430_t02 0

grmzm2g104443_t01 0

grmzm2g104449_t02 0

grmzm2g104456_t01 0

grmzm2g104464_t01 0

grmzm2g104468_t04 0

grmzm2g104501_t01 0

grmzm2g104504_t02 0

grmzm2g104511_t01 0

grmzm2g104534_t01 0

grmzm2g104538_t05 0

grmzm2g104542_t01 0

grmzm2g104546_t01 0

grmzm2g104551_t01 0

grmzm2g104575_t02 0

grmzm2g104584_t01 0

grmzm2g104608_t03 0

grmzm2g104613_t01 0

grmzm2g104626_t01 0

grmzm2g104632_t01 0

grmzm2g104639_t01 0

grmzm2g104649_t01 0

grmzm2g104655_t01 0

grmzm2g104658_t09 0

grmzm2g104704_t01 0

grmzm2g104730_t02 0

grmzm2g104732_t01 0

grmzm2g104754_t01 0

grmzm2g104760_t01 0

grmzm2g104769_t02 0

grmzm2g104783_t01 0

grmzm2g104833_t01 0

grmzm2g104836_t01 0

grmzm2g104843_t01 0

grmzm2g104847_t01 0

grmzm2g104876_t03 0

grmzm2g104882_t01 0

grmzm2g104898_t01 -1.24

grmzm2g104907_t01 0

grmzm2g104918_t02 0

grmzm2g104920_t01 0

grmzm2g104925_t01 0

grmzm2g104942_t02 -1.73

grmzm2g104955_t01 0

grmzm2g104958_t01 0

grmzm2g104983_t01 0

grmzm2g104999_t02 0

grmzm2g105004_t01 0

grmzm2g105005_t02 0

grmzm2g105019_t01 0

grmzm2g105167_t01 0

grmzm2g105184_t01 0

grmzm2g105189_t01 0

grmzm2g105221_t01 0

grmzm2g105229_t02 0

grmzm2g105253_t02 0

grmzm2g105283_t01 0

grmzm2g105297_t02 0

grmzm2g105302_t01 0

grmzm2g105307_t01 0

grmzm2g105317_t01 0

grmzm2g105330_t01 -0.97

grmzm2g105331_t01 0

grmzm2g105348_t01 0

grmzm2g105362_t01 0

grmzm2g105364_t01 0

grmzm2g105368_t01 0

grmzm2g105387_t04 0

grmzm2g105391_t01 0

grmzm2g105398_t01 0

grmzm2g105415_t01 0

grmzm2g105428_t01 0

grmzm2g105436_t01 0

grmzm2g105438_t02 0

grmzm2g105466_t01 0

grmzm2g105473_t02 0

grmzm2g105494_t01 0

grmzm2g105523_t02 0

grmzm2g105531_t01 0

grmzm2g105539_t02 0

grmzm2g105542_t01 0

grmzm2g105570_t01 0

grmzm2g105571_t01 0

grmzm2g105598_t02 0

grmzm2g105604_t02 0

grmzm2g105644_t01 -0.81

grmzm2g105682_t01 0

grmzm2g105712_t06 0

grmzm2g105741_t01 -1.19

grmzm2g105755_t01 0

grmzm2g105770_t01 0

grmzm2g105772_t04 0

grmzm2g105787_t02 1.66

grmzm2g105791_t02 0

grmzm2g105801_t01 0

grmzm2g105807_t03 0

grmzm2g105822_t01 0

grmzm2g105827_t03 0

grmzm2g105834_t01 0

grmzm2g105855_t01 0

grmzm2g105863_t07 0

grmzm2g105869_t02 0

grmzm2g105901_t01 0

grmzm2g105909_t03 0

grmzm2g105922_t01 0

grmzm2g105957_t02 0

grmzm2g105991_t01 0

grmzm2g105996_t01 0

grmzm2g106028_t01 0

grmzm2g106042_t02 0

grmzm2g106053_t01 0

grmzm2g106056_t01 0

grmzm2g106061_t01 0

grmzm2g106105_t02 0

grmzm2g106119_t01 0

grmzm2g106133_t02 -0.93

grmzm2g106140_t01 0

grmzm2g106141_t01 0

grmzm2g106164_t01 0

grmzm2g106165_t01 0

grmzm2g106190_t01 0

grmzm2g106204_t03 0

grmzm2g106213_t01 0

grmzm2g106216_t07 0

grmzm2g106218_t01 0

grmzm2g106222_t03 0

grmzm2g106233_t01 0

grmzm2g106245_t01 0

grmzm2g106250_t01 0

grmzm2g106263_t01 2.08

grmzm2g106283_t01 0

grmzm2g106288_t01 0

grmzm2g106293_t01 0

grmzm2g106308_t02 0

grmzm2g106331_t01 0

grmzm2g106363_t01 -1.6

grmzm2g106376_t01 0

grmzm2g106389_t01 0

grmzm2g106401_t01 0

grmzm2g106408_t01 0

grmzm2g106412_t03 0

grmzm2g106424_t01 0

grmzm2g106427_t01 0

grmzm2g106443_t01 0

grmzm2g106459_t02 0

grmzm2g106462_t01 0

grmzm2g106493_t01 0

grmzm2g106515_t01 0

grmzm2g106526_t02 0

grmzm2g106531_t01 0

grmzm2g106548_t01 0

grmzm2g106552_t01 0

grmzm2g106569_t02 0

grmzm2g106578_t01 0

grmzm2g106588_t01 -0.84

grmzm2g106590_t01 0

grmzm2g106600_t01 0

grmzm2g106604_t01 -1.6

grmzm2g106622_t01 1.95

grmzm2g106647_t02 0

grmzm2g106673_t06 0

grmzm2g106683_t04 0

grmzm2g106690_t01 0

grmzm2g106703_t01 0

grmzm2g106730_t01 0

grmzm2g106732_t02 0

grmzm2g106748_t02 0

grmzm2g106752_t01 0

grmzm2g106766_t01 0

grmzm2g106790_t01 0

grmzm2g106792_t01 0

grmzm2g106795_t03 0

grmzm2g106810_t01 0

grmzm2g106819_t03 0

grmzm2g106881_t01 0

grmzm2g106903_t01 0

grmzm2g106906_t02 0

grmzm2g106921_t01 0

grmzm2g106928_t01 0

grmzm2g106931_t01 0

grmzm2g106950_t01 0

grmzm2g106960_t01 0

grmzm2g106974_t01 0

grmzm2g107031_t01 0

grmzm2g107073_t01 0

grmzm2g107076_t01 0

grmzm2g107082_t01 0

grmzm2g107089_t02 0

grmzm2g107092_t01 0

grmzm2g107101_t05 0

grmzm2g107106_t05 0

grmzm2g107111_t01 0

grmzm2g107114_t01 0

grmzm2g107116_t02 0

grmzm2g107136_t01 0

grmzm2g107163_t01 0

grmzm2g107196_t02 0

grmzm2g107205_t01 0

grmzm2g107226_t01 -1.31

grmzm2g107289_t01 0

grmzm2g107299_t01 0

grmzm2g107304_t03 0

grmzm2g107306_t02 0

grmzm2g107309_t08 0

grmzm2g107336_t02 0

grmzm2g107362_t03 0

grmzm2g107373_t01 -1.06

grmzm2g107380_t01 0

grmzm2g107388_t01 0

grmzm2g107402_t01 0

grmzm2g107408_t01 0

grmzm2g107437_t01 0

grmzm2g107444_t01 0

grmzm2g107457_t01 0

grmzm2g107463_t02 0

grmzm2g107473_t01 0

grmzm2g107491_t02 0

grmzm2g107498_t01 0

grmzm2g107499_t02 0

grmzm2g107532_t01 0

grmzm2g107557_t01 0

grmzm2g107562_t01 0

grmzm2g107565_t02 0

grmzm2g107571_t01 0

grmzm2g107575_t01 0

grmzm2g107588_t04 0

grmzm2g107609_t01 0

grmzm2g107620_t01 0

grmzm2g107629_t05 0

grmzm2g107639_t02 0

grmzm2g107651_t01 0

grmzm2g107654_t01 0

grmzm2g107665_t01 0

grmzm2g107696_t01 0

grmzm2g107711_t01 0

grmzm2g107731_t01 0

grmzm2g107739_t03 0

grmzm2g107745_t01 0

grmzm2g107774_t02 0

grmzm2g107798_t01 0

grmzm2g107805_t01 0

grmzm2g107807_t02 0

grmzm2g107815_t01 0

grmzm2g107838_t02 0

grmzm2g107839_t02 0

grmzm2g107854_t01 0

grmzm2g107867_t03 0

grmzm2g107872_t01 0

grmzm2g107886_t01 0

grmzm2g107945_t01 -1.13

grmzm2g107968_t01 0

grmzm2g107984_t01 0

grmzm2g107985_t01 0

grmzm2g107987_t04 0

grmzm2g108008_t03 0

grmzm2g108027_t01 0

grmzm2g108032_t02 0

grmzm2g108084_t01 0

grmzm2g108085_t01 0

grmzm2g108087_t01 0

grmzm2g108103_t01 0

grmzm2g108115_t01 0

grmzm2g108125_t03 0

grmzm2g108133_t02 0

grmzm2g108135_t02 0

grmzm2g108138_t03 0

grmzm2g108144_t01 0

grmzm2g108147_t02 0

grmzm2g108149_t01 0

grmzm2g108158_t01 0

grmzm2g108221_t02 -0.82

grmzm2g108225_t01 0

grmzm2g108228_t02 0

grmzm2g108229_t02 0

grmzm2g108255_t01 0

grmzm2g108267_t01 0

grmzm2g108273_t01 0

grmzm2g108277_t02 0

grmzm2g108281_t04 0

grmzm2g108285_t01 0

grmzm2g108309_t02 0

grmzm2g108318_t01 0

grmzm2g108348_t02 0

grmzm2g108355_t03 0

grmzm2g108364_t02 0

grmzm2g108384_t01 0

grmzm2g108396_t01 0

grmzm2g108416_t02 0

grmzm2g108424_t02 0

grmzm2g108457_t02 0

grmzm2g108463_t01 0

grmzm2g108474_t04 0

grmzm2g108477_t01 0.85

grmzm2g108480_t01 0

grmzm2g108487_t01 0

grmzm2g108514_t01 -1.49

grmzm2g108530_t03 0

grmzm2g108537_t01 -1.87

grmzm2g108546_t02 0

grmzm2g108581_t01 0

grmzm2g108586_t01 0

grmzm2g108600_t02 0

grmzm2g108619_t01 0

grmzm2g108643_t01 0

grmzm2g108655_t01 0

grmzm2g108677_t01 0

grmzm2g108686_t01 0

grmzm2g108714_t01 0

grmzm2g108716_t02 0

grmzm2g108737_t01 0

grmzm2g108739_t02 0

grmzm2g108741_t01 0

grmzm2g108766_t01 0

grmzm2g108767_t01 0

grmzm2g108775_t01 0

grmzm2g108780_t05 0

grmzm2g108829_t01 0

grmzm2g108849_t02 0

grmzm2g108861_t01 0

grmzm2g108874_t03 0

grmzm2g108912_t01 0

grmzm2g108919_t03 0

grmzm2g108959_t01 0

grmzm2g108976_t02 0

grmzm2g108991_t03 0

grmzm2g108996_t01 0

grmzm2g109009_t02 0

grmzm2g109032_t02 0

grmzm2g109039_t01 0

grmzm2g109056_t01 0

grmzm2g109062_t02 0

grmzm2g109070_t02 0

grmzm2g109071_t02 -1.05

grmzm2g109121_t01 0

grmzm2g109130_t01 0

grmzm2g109165_t02 0

grmzm2g109201_t01 0

grmzm2g109221_t02 0

grmzm2g109225_t01 0

grmzm2g109244_t01 0

grmzm2g109268_t02 0

grmzm2g109274_t02 0

grmzm2g109284_t01 0

grmzm2g109315_t01 0

grmzm2g109326_t01 0

grmzm2g109348_t01 0

grmzm2g109352_t01 0

grmzm2g109354_t01 0

grmzm2g109383_t01 0

grmzm2g109405_t02 0

grmzm2g109425_t02 0

grmzm2g109429_t02 0

grmzm2g109431_t02 0

grmzm2g109448_t01 0

grmzm2g109464_t01 0

grmzm2g109472_t03 0

grmzm2g109509_t02 0

grmzm2g109526_t04 0

grmzm2g109527_t01 0

grmzm2g109560_t02 0

grmzm2g109566_t01 0

grmzm2g109589_t03 0

grmzm2g109595_t01 0

grmzm2g109624_t01 0

grmzm2g109639_t01 0

grmzm2g109651_t04 0

grmzm2g109668_t01 0

grmzm2g109674_t01 0.97

grmzm2g109677_t03 0

grmzm2g109680_t02 0

grmzm2g109720_t01 0

grmzm2g109725_t03 0

grmzm2g109731_t01 0

grmzm2g109743_t01 0

grmzm2g109753_t01 0

grmzm2g109783_t01 0

grmzm2g109785_t01 0

grmzm2g109818_t01 0

grmzm2g109821_t01 0

grmzm2g109823_t01 0

grmzm2g109831_t01 0

grmzm2g109843_t01 0

grmzm2g109865_t01 0

grmzm2g109869_t01 -0.94

grmzm2g109879_t02 0

grmzm2g109881_t01 0

grmzm2g109885_t01 0

grmzm2g109954_t02 0

grmzm2g109959_t02 -1.95

grmzm2g109967_t02 0

grmzm2g109976_t02 0

grmzm2g109977_t01 0

grmzm2g109987_t04 0

grmzm2g109997_t02 0

grmzm2g110004_t01 0

grmzm2g110023_t01 0

grmzm2g110027_t01 0

grmzm2g110076_t02 0

grmzm2g110109_t01 0

grmzm2g110116_t01 0

grmzm2g110135_t01 0

grmzm2g110141_t01 0

grmzm2g110143_t01 0

grmzm2g110145_t02 0

grmzm2g110158_t02 0

grmzm2g110175_t01 0

grmzm2g110192_t01 0

grmzm2g110198_t03 0

grmzm2g110201_t01 0

grmzm2g110216_t02 0

grmzm2g110233_t03 0

grmzm2g110236_t01 0

grmzm2g110258_t03 0

grmzm2g110277_t02 0

grmzm2g110287_t01 0

grmzm2g110289_t01 0

grmzm2g110294_t01 0

grmzm2g110298_t01 0

grmzm2g110304_t01 0

grmzm2g110309_t01 0

grmzm2g110328_t04 0

grmzm2g110333_t01 0

grmzm2g110378_t03 0

grmzm2g110381_t01 0

grmzm2g110398_t04 0

grmzm2g110402_t01 0

grmzm2g110406_t01 0

grmzm2g110408_t01 0

grmzm2g110412_t01 0

grmzm2g110500_t01 0

grmzm2g110504_t01 0

grmzm2g110509_t02 0

grmzm2g110511_t01 0

grmzm2g110541_t01 0

grmzm2g110548_t03 0

grmzm2g110558_t01 0

grmzm2g110567_t01 -2.61

grmzm2g110572_t01 0

grmzm2g110582_t01 0

grmzm2g110584_t02 0

grmzm2g110626_t01 0

grmzm2g110646_t02 0

grmzm2g110657_t01 0

grmzm2g110681_t01 -0.92

grmzm2g110685_t01 0

grmzm2g110714_t02 0

grmzm2g110726_t01 0

grmzm2g110735_t01 0

grmzm2g110822_t01 0

grmzm2g110834_t01 0

grmzm2g110843_t02 1.51

grmzm2g110862_t02 0

grmzm2g110878_t01 0

grmzm2g110881_t02 0

grmzm2g110897_t02 0

grmzm2g110908_t01 0

grmzm2g110922_t03 0

grmzm2g110932_t05 0

grmzm2g110952_t06 0

grmzm2g110960_t01 0

grmzm2g110983_t02 0

grmzm2g110993_t01 0

grmzm2g111014_t01 0

grmzm2g111017_t01 0

grmzm2g111022_t01 0

grmzm2g111138_t01 0

grmzm2g111143_t03 0

grmzm2g111146_t03 0

grmzm2g111164_t06 0

grmzm2g111172_t02 0

grmzm2g111200_t04 0

grmzm2g111204_t01 0

grmzm2g111208_t01 0

grmzm2g111214_t02 0

grmzm2g111216_t01 0

grmzm2g111224_t01 0

grmzm2g111225_t01 0

grmzm2g111232_t01 0

grmzm2g111238_t01 0

grmzm2g111247_t02 0

grmzm2g111269_t01 0

grmzm2g111271_t01 0

grmzm2g111300_t01 0

grmzm2g111304_t02 0

grmzm2g111309_t01 0

grmzm2g111354_t01 0

grmzm2g111411_t02 0

grmzm2g111472_t01 0

grmzm2g111475_t02 0

grmzm2g111491_t01 0

grmzm2g111501_t01 0

grmzm2g111510_t01 0

grmzm2g111511_t05 0

grmzm2g111521_t02 0

grmzm2g111529_t01 0

grmzm2g111533_t04 0

grmzm2g111566_t01 0

grmzm2g111579_t01 0

grmzm2g111593_t01 0

grmzm2g111611_t01 0

grmzm2g111614_t03 0

grmzm2g111632_t02 0

grmzm2g111637_t01 0

grmzm2g111642_t03 0

grmzm2g111657_t01 1.51

grmzm2g111666_t01 0

grmzm2g111672_t01 0

grmzm2g111679_t02 0

grmzm2g111697_t01 0

grmzm2g111713_t01 0

grmzm2g111720_t01 0

grmzm2g111756_t01 0

grmzm2g111760_t01 0

grmzm2g111780_t01 1.49

grmzm2g111782_t01 0

grmzm2g111818_t04 0

grmzm2g111837_t01 0

grmzm2g111846_t01 0

grmzm2g111872_t01 0

grmzm2g111886_t02 0

grmzm2g111906_t03 0

grmzm2g111907_t03 0

grmzm2g111912_t04 0

grmzm2g111922_t01 0

grmzm2g111923_t01 0

grmzm2g111928_t01 -1.68

grmzm2g111954_t02 0

grmzm2g111955_t01 0

grmzm2g111965_t01 0

grmzm2g111980_t01 0

grmzm2g111998_t01 0

grmzm2g112003_t01 0

grmzm2g112039_t02 0

grmzm2g112050_t01 0

grmzm2g112057_t01 0

grmzm2g112149_t01 0

grmzm2g112165_t01 0

grmzm2g112176_t01 0

grmzm2g112240_t01 0

grmzm2g112244_t01 0

grmzm2g112284_t01 -0.88

grmzm2g112285_t01 0

grmzm2g112296_t02 0

grmzm2g112309_t01 0

grmzm2g112329_t01 0

grmzm2g112336_t01 0

grmzm2g112337_t01 0

grmzm2g112352_t01 0

grmzm2g112366_t01 0

grmzm2g112392_t01 0

grmzm2g112425_t02 0

grmzm2g112429_t01 0

grmzm2g112456_t03 0

grmzm2g112470_t01 0

grmzm2g112488_t01 0

grmzm2g112530_t01 -1.13

grmzm2g112535_t01 0

grmzm2g112596_t01 0

grmzm2g112609_t01 0

grmzm2g112629_t01 0

grmzm2g112633_t01 0

grmzm2g112640_t01 0

grmzm2g112656_t01 0

grmzm2g112659_t01 0

grmzm2g112666_t04 0

grmzm2g112681_t01 0

grmzm2g112686_t01 0

grmzm2g112728_t02 0

grmzm2g112764_t01 0

grmzm2g112782_t01 0

grmzm2g112805_t01 0

grmzm2g112830_t04 0

grmzm2g112836_t01 -1.08

grmzm2g112839_t01 0

grmzm2g112853_t01 0

grmzm2g112865_t02 0

grmzm2g112895_t01 0

grmzm2g112903_t01 0

grmzm2g112912_t01 0

grmzm2g112914_t01 0

grmzm2g112968_t01 0

grmzm2g113002_t01 0

grmzm2g113016_t01 0

grmzm2g113017_t02 0

grmzm2g113033_t02 0

grmzm2g113052_t01 0

grmzm2g113056_t02 0

grmzm2g113060_t01 0

grmzm2g113062_t01 0

grmzm2g113073_t01 0

grmzm2g113093_t02 0

grmzm2g113096_t02 0

grmzm2g113099_t01 0

grmzm2g113127_t01 0

grmzm2g113129_t01 0

grmzm2g113135_t01 0

grmzm2g113137_t01 0

grmzm2g113139_t01 0

grmzm2g113156_t03 0

grmzm2g113158_t01 0

grmzm2g113159_t01 0

grmzm2g113163_t01 0

grmzm2g113181_t01 0

grmzm2g113200_t01 0

grmzm2g113202_t01 0

grmzm2g113203_t01 0

grmzm2g113205_t01 0

grmzm2g113216_t06 0

grmzm2g113228_t01 0

grmzm2g113232_t01 0

grmzm2g113239_t01 0

grmzm2g113241_t01 0

grmzm2g113244_t01 0

grmzm2g113250_t01 0

grmzm2g113252_t02 0

grmzm2g113253_t01 0

grmzm2g113262_t01 0

grmzm2g113264_t01 0

grmzm2g113267_t01 0

grmzm2g113276_t01 0

grmzm2g113287_t02 0

grmzm2g113295_t01 0

grmzm2g113325_t01 0

grmzm2g113332_t02 0

grmzm2g113340_t01 0

grmzm2g113349_t01 0

grmzm2g113351_t01 0

grmzm2g113355_t01 0

grmzm2g113364_t01 0

grmzm2g113372_t01 0

grmzm2g113373_t01 0

grmzm2g113382_t02 0

grmzm2g113391_t03 0

grmzm2g113396_t01 0

grmzm2g113401_t01 0

grmzm2g113408_t01 0

grmzm2g113409_t01 0

grmzm2g113414_t01 0

grmzm2g113415_t01 0

grmzm2g113418_t01 0

grmzm2g113420_t01 0

grmzm2g113421_t01 0

grmzm2g113423_t01 0

grmzm2g113432_t01 0

grmzm2g113439_t02 0

grmzm2g113453_t01 0

grmzm2g113470_t01 0

grmzm2g113473_t01 0

grmzm2g113476_t04 0

grmzm2g113480_t01 0

grmzm2g113495_t01 0

grmzm2g113506_t02 0

grmzm2g113512_t01 0

grmzm2g113513_t01 0

grmzm2g113532_t01 0

grmzm2g113569_t01 0

grmzm2g113585_t01 0

grmzm2g113592_t02 0

grmzm2g113596_t01 0

grmzm2g113613_t01 0.92

grmzm2g113618_t01 0

grmzm2g113619_t01 1.06

grmzm2g113629_t01 0

grmzm2g113655_t02 0

grmzm2g113668_t01 0

grmzm2g113682_t01 0

grmzm2g113696_t02 0

grmzm2g113720_t01 0

grmzm2g113726_t01 0

grmzm2g113742_t02 0

grmzm2g113750_t01 0

grmzm2g113761_t01 -1.39

grmzm2g113775_t01 0

grmzm2g113790_t01 0.9

grmzm2g113794_t01 0

grmzm2g113796_t01 0

grmzm2g113815_t01 0

grmzm2g113819_t01 0

grmzm2g113821_t01 0

grmzm2g113840_t02 0

grmzm2g113844_t01 0

grmzm2g113852_t01 0

grmzm2g113863_t01 0

grmzm2g113866_t01 0

grmzm2g113873_t03 0

grmzm2g113888_t01 0

grmzm2g113899_t01 0

grmzm2g113911_t02 0

grmzm2g113944_t01 0

grmzm2g113950_t01 0

grmzm2g113964_t01 0

grmzm2g113967_t01 0

grmzm2g113990_t01 0

grmzm2g113995_t04 0

grmzm2g114044_t02 0

grmzm2g114052_t01 0.88

grmzm2g114071_t01 -0.81

grmzm2g114093_t01 0

grmzm2g114098_t01 0

grmzm2g114107_t02 0

grmzm2g114126_t02 0

grmzm2g114127_t01 0

grmzm2g114131_t04 0

grmzm2g114137_t01 0

grmzm2g114140_t02 0

grmzm2g114153_t01 -1.27

grmzm2g114162_t01 0

grmzm2g114172_t02 0

grmzm2g114190_t01 0

grmzm2g114192_t01 0

grmzm2g114220_t02 0

grmzm2g114234_t01 0

grmzm2g114312_t02 0

grmzm2g114315_t02 0

grmzm2g114322_t02 0

grmzm2g114325_t01 0

grmzm2g114337_t01 0

grmzm2g114356_t01 0

grmzm2g114371_t03 0

grmzm2g114380_t01 0

grmzm2g114389_t01 0

grmzm2g114394_t01 0

grmzm2g114399_t04 0

grmzm2g114423_t01 0

grmzm2g114444_t01 0

grmzm2g114459_t02 0

grmzm2g114469_t03 0

grmzm2g114503_t01 0

grmzm2g114513_t02 0

grmzm2g114557_t01 0

grmzm2g114570_t03 0

grmzm2g114575_t02 0

grmzm2g114584_t03 0

grmzm2g114592_t01 0

grmzm2g114613_t02 0

grmzm2g114634_t02 0

grmzm2g114642_t03 0

grmzm2g114667_t01 0

grmzm2g114672_t05 0

grmzm2g114675_t01 0

grmzm2g114688_t01 0

grmzm2g114692_t01 0

grmzm2g114702_t03 0

grmzm2g114704_t02 0

grmzm2g114706_t01 0

grmzm2g114707_t01 0

grmzm2g114731_t01 0

grmzm2g114739_t01 0

grmzm2g114748_t01 0

grmzm2g114751_t02 0

grmzm2g114758_t01 0

grmzm2g114772_t01 0

grmzm2g114775_t01 0

grmzm2g114789_t01 0

grmzm2g114801_t01 0

grmzm2g114834_t02 0.88

grmzm2g114841_t01 0

grmzm2g114850_t01 0

grmzm2g114861_t01 0

grmzm2g114873_t02 -1.25

grmzm2g114888_t02 0

grmzm2g114895_t02 0

grmzm2g114906_t02 0

grmzm2g114911_t01 0

grmzm2g114954_t05 0

grmzm2g114988_t01 0

grmzm2g114998_t01 0

grmzm2g115000_t01 0

grmzm2g115013_t01 0

grmzm2g115047_t01 0

grmzm2g115105_t01 0

grmzm2g115131_t01 0

grmzm2g115156_t01 0

grmzm2g115162_t01 0

grmzm2g115176_t01 0

grmzm2g115182_t03 0

grmzm2g115198_t03 0

grmzm2g115243_t03 0

grmzm2g115257_t03 0

grmzm2g115304_t03 0

grmzm2g115342_t05 0

grmzm2g115357_t02 0

grmzm2g115372_t01 0

grmzm2g115388_t01 0

grmzm2g115390_t05 0

grmzm2g115395_t02 0

grmzm2g115398_t01 0

grmzm2g115400_t01 0

grmzm2g115420_t02 0

grmzm2g115422_t01 0

grmzm2g115424_t01 0

grmzm2g115437_t01 0.82

grmzm2g115442_t02 0

grmzm2g115451_t03 0

grmzm2g115456_t01 0

grmzm2g115491_t01 0

grmzm2g115504_t01 0

grmzm2g115516_t01 0

grmzm2g115518_t03 0

grmzm2g115564_t01 0

grmzm2g115579_t04 0

grmzm2g115598_t01 0

grmzm2g115612_t02 0

grmzm2g115615_t02 0

grmzm2g115621_t01 0

grmzm2g115633_t02 0

grmzm2g115635_t01 0

grmzm2g115646_t01 0

grmzm2g115658_t03 0

grmzm2g115660_t01 -1.05

grmzm2g115670_t01 0

grmzm2g115674_t01 0

grmzm2g115698_t01 0

grmzm2g115701_t02 0

grmzm2g115750_t01 0

grmzm2g115755_t01 0

grmzm2g115757_t03 0

grmzm2g115762_t01 0

grmzm2g115766_t02 0

grmzm2g115772_t02 0

grmzm2g115773_t01 0

grmzm2g115775_t03 0

grmzm2g115805_t02 0

grmzm2g115809_t01 0

grmzm2g115817_t01 0

grmzm2g115828_t05 0

grmzm2g115834_t03 0

grmzm2g115839_t01 1.41

grmzm2g115841_t01 0

grmzm2g115875_t01 0

grmzm2g115901_t01 0

grmzm2g115921_t01 0

grmzm2g115925_t01 0

grmzm2g115939_t01 0

grmzm2g115957_t01 0

grmzm2g115960_t01 0

grmzm2g115975_t01 0

grmzm2g115981_t01 0

grmzm2g116029_t01 0

grmzm2g116034_t01 0

grmzm2g116053_t01 0

grmzm2g116079_t06 0

grmzm2g116083_t01 0

grmzm2g116086_t01 0

grmzm2g116087_t03 0

grmzm2g116126_t01 0

grmzm2g116133_t05 0

grmzm2g116135_t03 0

grmzm2g116140_t01 0

grmzm2g116151_t01 0

grmzm2g116185_t01 0

grmzm2g116190_t01 0.99

grmzm2g116196_t02 0

grmzm2g116204_t01 0

grmzm2g116216_t01 0

grmzm2g116243_t03 0

grmzm2g116258_t01 0

grmzm2g116273_t01 0

grmzm2g116282_t03 0

grmzm2g116327_t03 0

grmzm2g116362_t01 0

grmzm2g116426_t01 0

grmzm2g116427_t01 0

grmzm2g116434_t01 0

grmzm2g116514_t01 0

grmzm2g116517_t02 0

grmzm2g116526_t01 0

grmzm2g116557_t03 0

grmzm2g116563_t01 0

grmzm2g116577_t01 0

grmzm2g116584_t06 0

grmzm2g116586_t01 0

grmzm2g116592_t01 0

grmzm2g116614_t02 0

grmzm2g116620_t01 0

grmzm2g116626_t01 0

grmzm2g116634_t02 0

grmzm2g116638_t01 0

grmzm2g116646_t02 0

grmzm2g116658_t01 0

grmzm2g116670_t01 0

grmzm2g116675_t03 0

grmzm2g116681_t02 0

grmzm2g116685_t01 0

grmzm2g116689_t01 0

grmzm2g116693_t01 0

grmzm2g116700_t02 0

grmzm2g116714_t02 1

grmzm2g116750_t01 0

grmzm2g116752_t01 0

grmzm2g116760_t01 0

grmzm2g116774_t01 0

grmzm2g116785_t02 0

grmzm2g116803_t01 0

grmzm2g116831_t01 0

grmzm2g116846_t02 0

grmzm2g116872_t01 0

grmzm2g116885_t01 0

grmzm2g116908_t01 0

grmzm2g116919_t01 0

grmzm2g116952_t05 0

grmzm2g116966_t01 0.84

grmzm2g116971_t01 0

grmzm2g117022_t01 0

grmzm2g117030_t02 0

grmzm2g117060_t02 0

grmzm2g117064_t01 0

grmzm2g117100_t01 0

grmzm2g117153_t01 0

grmzm2g117180_t01 0

grmzm2g117189_t02 0

grmzm2g117193_t04 -1.26

grmzm2g117198_t02 0

grmzm2g117207_t01 0

grmzm2g117222_t01 0

grmzm2g117240_t02 0

grmzm2g117263_t01 0

grmzm2g117278_t01 0

grmzm2g117281_t03 0

grmzm2g117298_t02 0

grmzm2g117300_t01 0

grmzm2g117344_t01 0

grmzm2g117346_t01 0

grmzm2g117357_t02 0

grmzm2g117369_t04 0

grmzm2g117388_t03 0

grmzm2g117401_t04 0

grmzm2g117405_t02 0

grmzm2g117410_t02 0

grmzm2g117412_t04 0

grmzm2g117439_t01 0

grmzm2g117441_t01 0

grmzm2g117458_t01 0

grmzm2g117459_t01 1.33

grmzm2g117465_t01 0

grmzm2g117497_t01 0

grmzm2g117507_t01 0

grmzm2g117513_t01 0

grmzm2g117544_t02 0

grmzm2g117558_t01 0

grmzm2g117582_t01 0

grmzm2g117593_t01 0

grmzm2g117609_t01 0

grmzm2g117612_t02 0

grmzm2g117614_t01 0

grmzm2g117615_t02 0

grmzm2g117619_t01 0

grmzm2g117633_t01 0

grmzm2g117642_t04 0

grmzm2g117644_t01 0

grmzm2g117680_t01 0

grmzm2g117715_t01 0

grmzm2g117742_t03 0

grmzm2g117746_t02 0

grmzm2g117754_t01 0

grmzm2g117755_t01 0

grmzm2g117763_t01 1.15

grmzm2g117771_t01 0

grmzm2g117786_t01 0

grmzm2g117796_t01 0

grmzm2g117804_t01 0

grmzm2g117811_t01 0

grmzm2g117823_t03 0

grmzm2g117836_t01 0

grmzm2g117854_t01 1.1

grmzm2g117865_t01 0

grmzm2g117870_t03 0

grmzm2g117900_t02 0

grmzm2g117935_t01 0

grmzm2g117956_t02 -1.63

grmzm2g117961_t01 0.96

grmzm2g117963_t01 0

grmzm2g117968_t01 0

grmzm2g117984_t01 0

grmzm2g117993_t01 0

grmzm2g118003_t04 0

grmzm2g118005_t01 0

grmzm2g118014_t05 0

grmzm2g118022_t01 0.89

grmzm2g118037_t01 0

grmzm2g118047_t02 0

grmzm2g118062_t01 0

grmzm2g118063_t01 0

grmzm2g118082_t02 0

grmzm2g118098_t01 0

grmzm2g118109_t01 0

grmzm2g118113_t01 0

grmzm2g118208_t01 0

grmzm2g118214_t02 0

grmzm2g118241_t04 0

grmzm2g118243_t01 0

grmzm2g118265_t07 0

grmzm2g118286_t01 0

grmzm2g118312_t01 0

grmzm2g118316_t02 0

grmzm2g118344_t02 0

grmzm2g118345_t01 0

grmzm2g118355_t04 -0.84

grmzm2g118362_t01 0

grmzm2g118363_t01 0

grmzm2g118366_t01 0

grmzm2g118385_t01 0

grmzm2g118393_t01 0

grmzm2g118403_t01 0

grmzm2g118409_t02 0

grmzm2g118433_t01 0

grmzm2g118441_t01 -1.44

grmzm2g118453_t01 0

grmzm2g118459_t01 0

grmzm2g118462_t01 0

grmzm2g118467_t01 0

grmzm2g118479_t01 0

grmzm2g118485_t01 0

grmzm2g118497_t02 0

grmzm2g118515_t03 0

grmzm2g118590_t02 0

grmzm2g118597_t03 0

grmzm2g118610_t01 0

grmzm2g118628_t01 0

grmzm2g118637_t01 0

grmzm2g118644_t02 0

grmzm2g118646_t02 0

grmzm2g118687_t03 0

grmzm2g118690_t02 0

grmzm2g118714_t01 0

grmzm2g118737_t01 0

grmzm2g118743_t01 0

grmzm2g118800_t02 0

grmzm2g118806_t04 0

grmzm2g118825_t01 0

grmzm2g118827_t01 0

grmzm2g118840_t02 0

grmzm2g118870_t02 0

grmzm2g118884_t01 0

grmzm2g118917_t01 0

grmzm2g118922_t01 0

grmzm2g118939_t02 0

grmzm2g118951_t01 0

grmzm2g118957_t03 0

grmzm2g118959_t01 0

grmzm2g118979_t02 0

grmzm2g119071_t01 0

grmzm2g119079_t02 0

grmzm2g119088_t01 0

grmzm2g119104_t01 0

grmzm2g119111_t01 0

grmzm2g119116_t02 0

grmzm2g119117_t01 0

grmzm2g119127_t01 0

grmzm2g119146_t02 0.83

grmzm2g119168_t01 0

grmzm2g119169_t01 0

grmzm2g119175_t01 0

grmzm2g119219_t02 0

grmzm2g119248_t01 0

grmzm2g119249_t01 0

grmzm2g119252_t01 0

grmzm2g119256_t01 0

grmzm2g119258_t02 0

grmzm2g119286_t04 0

grmzm2g119300_t03 0

grmzm2g119303_t01 1.18

grmzm2g119311_t01 0

grmzm2g119316_t01 0

grmzm2g119345_t02 0

grmzm2g119357_t01 0

grmzm2g119361_t03 0

grmzm2g119370_t01 1.44

grmzm2g119383_t01 0

grmzm2g119393_t01 0

grmzm2g119411_t01 0

grmzm2g119465_t02 0

grmzm2g119468_t01 0

grmzm2g119471_t03 0

grmzm2g119482_t01 0

grmzm2g119483_t01 0

grmzm2g119494_t01 0

grmzm2g119499_t02 0

grmzm2g119516_t04 0

grmzm2g119517_t01 0

grmzm2g119521_t01 0

grmzm2g119523_t04 0

grmzm2g119527_t01 0

grmzm2g119536_t01 0

grmzm2g119546_t02 0

grmzm2g119571_t01 0

grmzm2g119583_t01 0

grmzm2g119623_t02 -1.16

grmzm2g119627_t01 0

grmzm2g119638_t02 0

grmzm2g119640_t07 0

grmzm2g119650_t01 0

grmzm2g119657_t01 0

grmzm2g119675_t01 0

grmzm2g119691_t02 0

grmzm2g119696_t01 0

grmzm2g119703_t01 0

grmzm2g119705_t01 0

grmzm2g119714_t01 0

grmzm2g119722_t02 0

grmzm2g119725_t03 0

grmzm2g119736_t01 0

grmzm2g119740_t01 0

grmzm2g119745_t01 0

grmzm2g119749_t04 0

grmzm2g119755_t01 0

grmzm2g119759_t06 0

grmzm2g119761_t02 0

grmzm2g119766_t01 -1.65

grmzm2g119769_t02 0

grmzm2g119773_t03 0

grmzm2g119778_t01 0

grmzm2g119782_t01 0

grmzm2g119783_t01 0

grmzm2g119791_t03 1.02

grmzm2g119802_t01 0

grmzm2g119809_t03 0

grmzm2g119823_t01 0

grmzm2g119850_t01 0

grmzm2g119852_t01 0

grmzm2g119894_t01 0

grmzm2g119906_t01 0

grmzm2g119926_t05 0

grmzm2g119930_t01 0

grmzm2g119932_t01 0

grmzm2g119933_t04 0

grmzm2g119941_t01 0

grmzm2g119950_t01 0

grmzm2g119970_t01 0

grmzm2g119999_t02 1.23

grmzm2g120035_t02 0

grmzm2g120041_t02 0

grmzm2g120047_t01 0

grmzm2g120069_t01 0

grmzm2g120079_t02 0

grmzm2g120085_t02 -1.26

grmzm2g120114_t01 0

grmzm2g120115_t02 0

grmzm2g120136_t01 0

grmzm2g120151_t01 0

grmzm2g120163_t01 0

grmzm2g120167_t01 0

grmzm2g120173_t01 0

grmzm2g120175_t02 0

grmzm2g120202_t01 0

grmzm2g120257_t01 0

grmzm2g120271_t02 0

grmzm2g120296_t01 0

grmzm2g120300_t02 0

grmzm2g120302_t01 0

grmzm2g120304_t04 0

grmzm2g120320_t01 -0.84

grmzm2g120329_t02 1.08

grmzm2g120371_t01 0

grmzm2g120373_t01 0

grmzm2g120408_t01 0

grmzm2g120432_t01 0

grmzm2g120484_t01 0

grmzm2g120517_t03 0

grmzm2g120530_t01 0

grmzm2g120539_t01 0

grmzm2g120563_t05 0

grmzm2g120572_t01 0

grmzm2g120575_t03 0

grmzm2g120578_t03 0

grmzm2g120579_t01 0

grmzm2g120581_t01 0

grmzm2g120596_t01 0

grmzm2g120619_t01 -1.56

grmzm2g120652_t01 0

grmzm2g120674_t01 0

grmzm2g120724_t01 0

grmzm2g120750_t02 0

grmzm2g120784_t02 0

grmzm2g120810_t01 0

grmzm2g120816_t05 0

grmzm2g120857_t02 0

grmzm2g120870_t03 0

grmzm2g120897_t01 0

grmzm2g120922_t01 0

grmzm2g120933_t01 0

grmzm2g120942_t02 0

grmzm2g120971_t01 0

grmzm2g120973_t04 0

grmzm2g120975_t01 0

grmzm2g120983_t02 -1.09

grmzm2g120987_t04 0

grmzm2g121022_t04 0

grmzm2g121024_t01 0

grmzm2g121034_t01 0

grmzm2g121063_t02 0

grmzm2g121066_t01 0

grmzm2g121074_t01 0

grmzm2g121075_t01 0

grmzm2g121096_t01 0.98

grmzm2g121117_t01 0

grmzm2g121123_t02 0

grmzm2g121128_t01 0

grmzm2g121150_t01 0

grmzm2g121151_t01 0

grmzm2g121166_t03 0

grmzm2g121176_t01 0

grmzm2g121186_t01 0

grmzm2g121200_t01 0

grmzm2g121208_t02 0

grmzm2g121221_t03 -1.67

grmzm2g121223_t02 0

grmzm2g121237_t01 0

grmzm2g121269_t02 0

grmzm2g121293_t01 0

grmzm2g121303_t05 0

grmzm2g121309_t01 0

grmzm2g121312_t01 0

grmzm2g121333_t01 0

grmzm2g121354_t01 0

grmzm2g121360_t01 0

grmzm2g121404_t02 0

grmzm2g121452_t01 0

grmzm2g121456_t02 0

grmzm2g121468_t01 0

grmzm2g121486_t01 0

grmzm2g121494_t02 0

grmzm2g121495_t01 2.01

grmzm2g121510_t01 0

grmzm2g121514_t02 0

grmzm2g121516_t01 0

grmzm2g121565_t02 0

grmzm2g121573_t02 0

grmzm2g121612_t01 0

grmzm2g121621_t01 0

grmzm2g121625_t01 0

grmzm2g121683_t01 0

grmzm2g121700_t02 0

grmzm2g121704_t01 0

grmzm2g121715_t01 0

grmzm2g121743_t02 0

grmzm2g121753_t01 0

grmzm2g121776_t02 0

grmzm2g121785_t01 0

grmzm2g121790_t01 0

grmzm2g121820_t02 0

grmzm2g121826_t02 0

grmzm2g121840_t01 0

grmzm2g121843_t01 0

grmzm2g121851_t01 0

grmzm2g121868_t01 0

grmzm2g121871_t01 0

grmzm2g121878_t04 0

grmzm2g121905_t04 0

grmzm2g121942_t04 0

grmzm2g121948_t04 0

grmzm2g121960_t01 0

grmzm2g122038_t01 0

grmzm2g122045_t01 0

grmzm2g122061_t02 0

grmzm2g122064_t01 0

grmzm2g122072_t01 0

grmzm2g122073_t01 0

grmzm2g122076_t02 0

grmzm2g122108_t03 0

grmzm2g122116_t04 0

grmzm2g122126_t01 0

grmzm2g122135_t02 0

grmzm2g122139_t02 0

grmzm2g122145_t02 0

grmzm2g122148_t02 1.1

grmzm2g122164_t01 0

grmzm2g122187_t01 0

grmzm2g122198_t02 0

grmzm2g122199_t03 0

grmzm2g122203_t01 0

grmzm2g122231_t01 -1.04

grmzm2g122239_t03 0

grmzm2g122251_t01 0

grmzm2g122267_t01 0

grmzm2g122274_t01 0

grmzm2g122276_t01 0

grmzm2g122277_t01 -0.83

grmzm2g122290_t01 0

grmzm2g122296_t01 0

grmzm2g122302_t01 0

grmzm2g122306_t01 0

grmzm2g122324_t01 0

grmzm2g122327_t01 0

grmzm2g122330_t01 0

grmzm2g122335_t03 0

grmzm2g122337_t01 0

grmzm2g122344_t01 0

grmzm2g122362_t01 0

grmzm2g122431_t01 0

grmzm2g122437_t01 0

grmzm2g122443_t01 0

grmzm2g122447_t01 0

grmzm2g122453_t01 0

grmzm2g122476_t02 0

grmzm2g122479_t05 0

grmzm2g122481_t01 0

grmzm2g122543_t01 0

grmzm2g122569_t01 0

grmzm2g122584_t01 0

grmzm2g122607_t01 0

grmzm2g122618_t01 0

grmzm2g122631_t01 0

grmzm2g122666_t01 0

grmzm2g122715_t02 0

grmzm2g122750_t01 0

grmzm2g122767_t01 0

grmzm2g122780_t01 0

grmzm2g122787_t06 0

grmzm2g122793_t02 0

grmzm2g122805_t01 0

grmzm2g122810_t04 0

grmzm2g122811_t01 0

grmzm2g122843_t02 0

grmzm2g122848_t01 0

grmzm2g122853_t01 -0.81

grmzm2g122863_t01 0

grmzm2g122869_t01 0

grmzm2g122871_t04 0

grmzm2g122892_t03 0

grmzm2g122897_t01 0

grmzm2g122937_t02 0

grmzm2g122943_t01 0

grmzm2g122954_t01 0

grmzm2g122965_t01 0

grmzm2g122983_t02 0

grmzm2g122999_t01 0

grmzm2g123014_t01 0

grmzm2g123029_t01 0

grmzm2g123033_t05 0

grmzm2g123089_t05 0

grmzm2g123094_t01 0

grmzm2g123122_t01 0

grmzm2g123128_t01 0

grmzm2g123140_t04 1.71

grmzm2g123143_t01 0

grmzm2g123159_t02 0

grmzm2g123200_t01 0

grmzm2g123204_t01 0

grmzm2g123227_t03 0

grmzm2g123234_t01 0

grmzm2g123246_t01 0

grmzm2g123277_t01 0

grmzm2g123309_t01 0

grmzm2g123328_t01 0

grmzm2g123331_t01 0

grmzm2g123362_t01 0

grmzm2g123371_t01 0

grmzm2g123407_t01 0

grmzm2g123410_t01 0

grmzm2g123428_t01 0

grmzm2g123440_t01 0

grmzm2g123450_t01 0

grmzm2g123459_t01 0

grmzm2g123482_t04 0

grmzm2g123486_t02 0

grmzm2g123495_t02 0

grmzm2g123499_t01 0

grmzm2g123503_t03 0

grmzm2g123519_t02 0

grmzm2g123527_t02 0

grmzm2g123534_t01 0

grmzm2g123537_t02 0

grmzm2g123540_t01 0

grmzm2g123544_t01 0

grmzm2g123558_t01 0

grmzm2g123604_t02 0

grmzm2g123624_t02 0

grmzm2g123652_t02 0

grmzm2g123660_t02 0

grmzm2g123667_t07 -2.36

grmzm2g123709_t01 0

grmzm2g123714_t01 0

grmzm2g123719_t01 0

grmzm2g123732_t01 0

grmzm2g123762_t01 0

grmzm2g123787_t01 0

grmzm2g123790_t02 0

grmzm2g123791_t01 0

grmzm2g123796_t03 0

grmzm2g123807_t02 0

grmzm2g123815_t02 0

grmzm2g123831_t01 0

grmzm2g123876_t01 0

grmzm2g123886_t01 0

grmzm2g123887_t01 0

grmzm2g123896_t01 0

grmzm2g123900_t01 0

grmzm2g123922_t01 0

grmzm2g123940_t05 0

grmzm2g123966_t01 0

grmzm2g123977_t01 0

grmzm2g123987_t01 0

grmzm2g123996_t01 0

grmzm2g124026_t01 0

grmzm2g124042_t01 3.63

grmzm2g124047_t01 0

grmzm2g124051_t02 0

grmzm2g124059_t01 0

grmzm2g124066_t02 0

grmzm2g124070_t01 0

grmzm2g124079_t01 0

grmzm2g124096_t01 0

grmzm2g124103_t03 0

grmzm2g124143_t01 0

grmzm2g124151_t01 0

grmzm2g124175_t01 0

grmzm2g124203_t02 0

grmzm2g124209_t03 0

grmzm2g124243_t01 0

grmzm2g124254_t01 0

grmzm2g124288_t01 0

grmzm2g124307_t02 0

grmzm2g124313_t01 0

grmzm2g124319_t01 0

grmzm2g124321_t03 0

grmzm2g124335_t02 0

grmzm2g124353_t01 0

grmzm2g124365_t03 0

grmzm2g124371_t01 0

grmzm2g124377_t01 0

grmzm2g124402_t01 0

grmzm2g124411_t04 0

grmzm2g124416_t04 0

grmzm2g124421_t01 0

grmzm2g124434_t02 0

grmzm2g124441_t01 0

grmzm2g124455_t01 0

grmzm2g124460_t03 0

grmzm2g124466_t01 0

grmzm2g124473_t01 0

grmzm2g124476_t01 0

grmzm2g124495_t01 0

grmzm2g124502_t01 0

grmzm2g124509_t02 0

grmzm2g124530_t02 0

grmzm2g124532_t01 0

grmzm2g124540_t02 -1.41

grmzm2g124550_t02 0

grmzm2g124553_t01 0

grmzm2g124557_t01 0

grmzm2g124563_t01 0

grmzm2g124567_t01 0

grmzm2g124573_t01 0

grmzm2g124576_t02 0

grmzm2g124593_t03 0

grmzm2g124602_t01 0

grmzm2g124617_t01 0

grmzm2g124620_t01 0

grmzm2g124638_t01 0

grmzm2g124644_t01 0

grmzm2g124671_t01 0

grmzm2g124701_t02 0

grmzm2g124715_t02 0

grmzm2g124732_t02 0

grmzm2g124758_t03 0

grmzm2g124759_t01 0

grmzm2g124791_t03 0

grmzm2g124815_t01 0

grmzm2g124820_t01 0

grmzm2g124863_t01 -1.03

grmzm2g124872_t01 0

grmzm2g124883_t01 0

grmzm2g124886_t03 0

grmzm2g124911_t02 0

grmzm2g124915_t03 0

grmzm2g124918_t02 0

grmzm2g124963_t03 0

grmzm2g124965_t04 0

grmzm2g124974_t01 0

grmzm2g124975_t02 0

grmzm2g124983_t01 0

grmzm2g125001_t01 0

grmzm2g125034_t01 0

grmzm2g125044_t01 0

grmzm2g125054_t01 0

grmzm2g125072_t02 0

grmzm2g125083_t01 0

grmzm2g125138_t01 0

grmzm2g125148_t04 0

grmzm2g125149_t03 0

grmzm2g125175_t04 0

grmzm2g125193_t04 0

grmzm2g125233_t01 0

grmzm2g125239_t03 0

grmzm2g125241_t01 0

grmzm2g125263_t01 0.9

grmzm2g125266_t01 0

grmzm2g125268_t02 0

grmzm2g125271_t01 0

grmzm2g125285_t02 0

grmzm2g125294_t01 0

grmzm2g125300_t03 0

grmzm2g125304_t02 0

grmzm2g125308_t02 0

grmzm2g125310_t02 0

grmzm2g125314_t01 0

grmzm2g125320_t01 0

grmzm2g125342_t01 0

grmzm2g125352_t02 0

grmzm2g125411_t01 0

grmzm2g125420_t01 0

grmzm2g125424_t03 0

grmzm2g125432_t01 0

grmzm2g125441_t07 0

grmzm2g125455_t03 0

grmzm2g125482_t01 0

grmzm2g125487_t01 0

grmzm2g125494_t03 0

grmzm2g125495_t01 0

grmzm2g125507_t01 0

grmzm2g125512_t03 0

grmzm2g125522_t01 0

grmzm2g125527_t01 0

grmzm2g125529_t03 0

grmzm2g125531_t03 0

grmzm2g125544_t02 0

grmzm2g125552_t04 0

grmzm2g125556_t04 0

grmzm2g125557_t02 0

grmzm2g125617_t01 0

grmzm2g125635_t01 0

grmzm2g125642_t01 0

grmzm2g125653_t01 0

grmzm2g125656_t01 0

grmzm2g125728_t02 0

grmzm2g125762_t03 0

grmzm2g125775_t01 -1.12

grmzm2g125777_t01 0

grmzm2g125784_t01 0

grmzm2g125832_t01 0

grmzm2g125838_t01 0

grmzm2g125844_t04 0

grmzm2g125850_t01 0

grmzm2g125853_t01 0

grmzm2g125891_t01 0

grmzm2g125893_t01 0

grmzm2g125923_t01 0

grmzm2g125931_t01 0

grmzm2g125935_t01 0

grmzm2g125969_t03 0

grmzm2g125977_t02 0

grmzm2g126002_t01 0

grmzm2g126010_t02 0

grmzm2g126026_t01 0

grmzm2g126038_t01 0

grmzm2g126048_t07 0

grmzm2g126106_t02 0

grmzm2g126120_t02 0

grmzm2g126128_t01 0

grmzm2g126131_t02 0

grmzm2g126161_t01 0

grmzm2g126170_t05 0

grmzm2g126182_t01 0

grmzm2g126190_t03 0

grmzm2g126199_t01 0

grmzm2g126218_t01 0

grmzm2g126253_t01 0

grmzm2g126260_t02 0

grmzm2g126261_t03 0

grmzm2g126266_t01 0

grmzm2g126290_t02 0

grmzm2g126292_t01 0

grmzm2g126302_t01 0

grmzm2g126338_t01 0

grmzm2g126361_t01 0

grmzm2g126386_t01 -1.04

grmzm2g126413_t01 0

grmzm2g126435_t02 0

grmzm2g126447_t02 0

grmzm2g126453_t02 0

grmzm2g126468_t02 0

grmzm2g126471_t02 0

grmzm2g126484_t02 0

grmzm2g126496_t01 0

grmzm2g126505_t01 -1.97

grmzm2g126507_t01 0

grmzm2g126517_t03 0

grmzm2g126545_t06 0

grmzm2g126552_t01 0

grmzm2g126572_t01 0

grmzm2g126580_t01 0

grmzm2g126582_t01 0

grmzm2g126586_t02 0

grmzm2g126594_t04 0

grmzm2g126601_t01 0

grmzm2g126603_t01 0

grmzm2g126646_t01 -1.35

grmzm2g126656_t02 0

grmzm2g126664_t01 0

grmzm2g126682_t01 -1.06

grmzm2g126691_t01 0

grmzm2g126706_t03 0

grmzm2g126745_t01 0

grmzm2g126763_t02 0

grmzm2g126765_t02 0

grmzm2g126774_t01 0

grmzm2g126795_t01 0

grmzm2g126808_t02 0

grmzm2g126812_t01 0

grmzm2g126821_t02 0

grmzm2g126832_t03 0

grmzm2g126834_t01 0

grmzm2g126835_t01 0

grmzm2g126839_t02 0

grmzm2g126853_t04 0

grmzm2g126858_t02 0

grmzm2g126860_t01 0

grmzm2g126865_t01 0

grmzm2g126900_t02 -1.01

grmzm2g126920_t01 0

grmzm2g126928_t01 0

grmzm2g126956_t01 0

grmzm2g126972_t02 0

grmzm2g126975_t01 1.7

grmzm2g126988_t01 0

grmzm2g127031_t01 0

grmzm2g127034_t01 0

grmzm2g127050_t02 0

grmzm2g127067_t01 0

grmzm2g127072_t03 -1.11

grmzm2g127115_t01 0

grmzm2g127117_t01 0

grmzm2g127123_t01 0

grmzm2g127139_t01 0

grmzm2g127141_t01 0

grmzm2g127147_t03 0

grmzm2g127150_t02 0

grmzm2g127154_t01 0

grmzm2g127168_t01 0

grmzm2g127173_t01 0

grmzm2g127180_t01 0

grmzm2g127230_t02 0

grmzm2g127297_t02 0

grmzm2g127308_t02 -0.95

grmzm2g127312_t02 0

grmzm2g127340_t02 0

grmzm2g127350_t01 0

grmzm2g127361_t02 0

grmzm2g127374_t02 0

grmzm2g127386_t02 0

grmzm2g127393_t02 0

grmzm2g127396_t01 0

grmzm2g127404_t01 -1.04

grmzm2g127416_t01 0

grmzm2g127418_t01 -1.69

grmzm2g127421_t01 0

grmzm2g127426_t01 0

grmzm2g127453_t02 0

grmzm2g127457_t01 0

grmzm2g127481_t02 0

grmzm2g127490_t01 0

grmzm2g127510_t01 0

grmzm2g127537_t01 1.06

grmzm2g127546_t02 0

grmzm2g127548_t01 0

grmzm2g127559_t01 0

grmzm2g127562_t02 0

grmzm2g127581_t01 0

grmzm2g127591_t02 0

grmzm2g127598_t01 0

grmzm2g127608_t03 0

grmzm2g127609_t06 0

grmzm2g127623_t01 0

grmzm2g127632_t01 0

grmzm2g127635_t01 -1.68

grmzm2g127648_t01 0

grmzm2g127665_t01 0

grmzm2g127683_t04 0

grmzm2g127687_t01 0

grmzm2g127714_t02 0

grmzm2g127717_t01 0

grmzm2g127729_t03 0

grmzm2g127739_t03 0

grmzm2g127780_t01 0

grmzm2g127798_t01 0

grmzm2g127821_t01 0

grmzm2g127844_t02 0

grmzm2g127853_t01 0

grmzm2g127911_t03 0

grmzm2g127924_t01 0

grmzm2g127948_t04 0

grmzm2g127949_t02 0

grmzm2g127963_t01 0

grmzm2g127984_t02 0

grmzm2g128012_t01 0

grmzm2g128016_t02 0

grmzm2g128056_t02 0

grmzm2g128057_t01 0

grmzm2g128072_t02 0

grmzm2g128078_t01 0

grmzm2g128080_t02 0

grmzm2g128092_t02 0

grmzm2g128109_t01 0

grmzm2g128131_t04 0

grmzm2g128146_t01 0

grmzm2g128171_t01 0

grmzm2g128176_t01 0

grmzm2g128179_t01 0

grmzm2g128189_t01 0

grmzm2g128206_t02 0

grmzm2g128212_t01 0

grmzm2g128214_t01 0

grmzm2g128219_t01 0

grmzm2g128228_t01 0

grmzm2g128233_t01 0

grmzm2g128268_t03 0

grmzm2g128315_t01 0

grmzm2g128319_t01 0

grmzm2g128358_t01 -1.03

grmzm2g128399_t01 0

grmzm2g128421_t02 0

grmzm2g128426_t01 0

grmzm2g128432_t02 0

grmzm2g128434_t01 0

grmzm2g128444_t02 0

grmzm2g128454_t01 0

grmzm2g128466_t01 0

grmzm2g128477_t03 0

grmzm2g128485_t03 0

grmzm2g128487_t02 0

grmzm2g128491_t01 0

grmzm2g128504_t01 0

grmzm2g128515_t01 0

grmzm2g128518_t02 0

grmzm2g128564_t02 -0.87

grmzm2g128577_t02 0

grmzm2g128579_t03 0

grmzm2g128605_t01 0

grmzm2g128608_t01 0

grmzm2g128613_t01 0

grmzm2g128641_t01 0

grmzm2g128644_t01 0

grmzm2g128651_t01 0

grmzm2g128658_t01 0

grmzm2g128663_t01 0

grmzm2g128665_t01 0

grmzm2g128682_t01 0

grmzm2g128688_t01 0

grmzm2g128737_t01 0

grmzm2g128744_t02 0

grmzm2g128807_t04 0

grmzm2g128809_t01 0

grmzm2g128850_t01 0

grmzm2g128865_t01 0

grmzm2g128877_t04 0

grmzm2g128880_t01 0

grmzm2g128895_t01 0

grmzm2g128902_t02 0

grmzm2g128914_t01 0

grmzm2g128918_t01 0

grmzm2g128922_t03 0

grmzm2g128929_t01 0

grmzm2g128934_t03 0

grmzm2g128935_t01 -0.92

grmzm2g128969_t02 0

grmzm2g128971_t01 0

grmzm2g128981_t01 0

grmzm2g128987_t01 0

grmzm2g128992_t03 0

grmzm2g128995_t06 0

grmzm2g129007_t01 0

grmzm2g129015_t02 0

grmzm2g129018_t01 -1.58

grmzm2g129065_t01 0

grmzm2g129071_t01 0

grmzm2g129080_t01 0

grmzm2g129083_t01 0

grmzm2g129108_t02 0

grmzm2g129146_t03 0

grmzm2g129147_t02 -1.68

grmzm2g129150_t01 0

grmzm2g129155_t02 0

grmzm2g129169_t02 0

grmzm2g129189_t02 0

grmzm2g129208_t03 0

grmzm2g129209_t01 0

grmzm2g129218_t02 0

grmzm2g129238_t02 0

grmzm2g129243_t01 0

grmzm2g129246_t05 0

grmzm2g129261_t01 0

grmzm2g129266_t02 0

grmzm2g129268_t01 0

grmzm2g129278_t01 0

grmzm2g129288_t04 0

grmzm2g129291_t01 0

grmzm2g129302_t01 0

grmzm2g129304_t01 0

grmzm2g129354_t01 0

grmzm2g129357_t01 0

grmzm2g129375_t01 0

grmzm2g129405_t01 0

grmzm2g129428_t01 0

grmzm2g129431_t01 0

grmzm2g129444_t03 0

grmzm2g129448_t01 0

grmzm2g129451_t01 0

grmzm2g129453_t01 0

grmzm2g129457_t01 0

grmzm2g129493_t01 0

grmzm2g129513_t01 0

grmzm2g129554_t02 0

grmzm2g129569_t01 0

grmzm2g129585_t01 0

grmzm2g129615_t03 0

grmzm2g129620_t01 0

grmzm2g129642_t01 0

grmzm2g129675_t01 0

grmzm2g129700_t02 0

grmzm2g129777_t01 0

grmzm2g129781_t02 0

grmzm2g129783_t01 0

grmzm2g129804_t02 0

grmzm2g129806_t01 0

grmzm2g129817_t02 0

grmzm2g129860_t02 0

grmzm2g129865_t01 -0.96

grmzm2g129874_t02 0

grmzm2g129879_t01 0

grmzm2g129889_t01 0

grmzm2g129907_t02 0

grmzm2g129929_t01 0

grmzm2g129954_t01 0

grmzm2g129973_t01 0

grmzm2g129987_t01 0

grmzm2g130010_t01 0

grmzm2g130018_t02 0

grmzm2g130034_t03 0

grmzm2g130043_t02 0

grmzm2g130046_t01 0

grmzm2g130052_t01 0

grmzm2g130053_t02 0

grmzm2g130062_t01 0

grmzm2g130064_t02 0

grmzm2g130079_t01 0

grmzm2g130085_t01 0

grmzm2g130095_t01 0

grmzm2g130101_t02 0

grmzm2g130109_t01 0

grmzm2g130121_t01 0

grmzm2g130127_t01 0

grmzm2g130149_t01 0

grmzm2g130167_t02 0

grmzm2g130169_t01 0

grmzm2g130173_t04 0

grmzm2g130207_t06 0

grmzm2g130224_t01 0

grmzm2g130230_t02 0

grmzm2g130232_t03 0

grmzm2g130276_t01 0

grmzm2g130278_t02 0

grmzm2g130287_t01 0

grmzm2g130305_t01 0

grmzm2g130314_t01 0

grmzm2g130332_t01 0

grmzm2g130333_t01 0

grmzm2g130339_t01 0

grmzm2g130348_t01 0

grmzm2g130358_t01 0

grmzm2g130366_t01 0

grmzm2g130375_t05 0

grmzm2g130379_t01 0

grmzm2g130398_t01 0

grmzm2g130404_t01 0

grmzm2g130425_t01 0

grmzm2g130432_t01 0

grmzm2g130440_t05 0

grmzm2g130442_t01 0

grmzm2g130449_t01 0

grmzm2g130454_t02 0

grmzm2g130477_t01 0

grmzm2g130505_t03 0

grmzm2g130528_t01 0

grmzm2g130544_t03 0

grmzm2g130558_t01 0

grmzm2g130586_t03 0

grmzm2g130634_t02 0

grmzm2g130651_t01 0

grmzm2g130724_t01 0

grmzm2g130739_t01 0

grmzm2g130746_t01 0

grmzm2g130749_t02 0

grmzm2g130764_t01 0

grmzm2g130773_t02 0

grmzm2g130790_t01 0

grmzm2g130800_t02 0

grmzm2g130854_t01 0

grmzm2g130868_t01 0

grmzm2g130883_t01 0

grmzm2g130927_t03 0

grmzm2g130933_t01 0

grmzm2g130943_t02 0

grmzm2g130953_t04 0

grmzm2g130959_t03 0

grmzm2g130987_t04 0

grmzm2g131020_t01 0

grmzm2g131024_t02 0

grmzm2g131026_t01 0

grmzm2g131055_t01 1.61

grmzm2g131070_t01 0

grmzm2g131087_t02 0

grmzm2g131106_t01 0

grmzm2g131152_t01 0

grmzm2g131155_t01 0

grmzm2g131165_t01 0

grmzm2g131177_t01 0

grmzm2g131202_t01 0

grmzm2g131205_t06 -1.1

grmzm2g131221_t01 0

grmzm2g131243_t01 0

grmzm2g131245_t01 0

grmzm2g131249_t03 0

grmzm2g131254_t05 0

grmzm2g131280_t01 0

grmzm2g131305_t02 0

grmzm2g131321_t02 0

grmzm2g131324_t02 0

grmzm2g131329_t01 0

grmzm2g131332_t01 0

grmzm2g131334_t03 0

grmzm2g131340_t01 0

grmzm2g131431_t01 0

grmzm2g131434_t02 0

grmzm2g131443_t02 0

grmzm2g131448_t01 0

grmzm2g131467_t01 0

grmzm2g131473_t01 0

grmzm2g131476_t02 1.01

grmzm2g131482_t02 0

grmzm2g131489_t01 0

grmzm2g131516_t02 0

grmzm2g131539_t02 0

grmzm2g131575_t01 0

grmzm2g131577_t01 0

grmzm2g131591_t01 0

grmzm2g131595_t01 0

grmzm2g131611_t02 0

grmzm2g131623_t03 0

grmzm2g131667_t03 0

grmzm2g131683_t01 0

grmzm2g131697_t02 0

grmzm2g131699_t01 0

grmzm2g131708_t06 0

grmzm2g131723_t03 0

grmzm2g131727_t03 0

grmzm2g131733_t01 0

grmzm2g131749_t01 0

grmzm2g131756_t03 0.86

grmzm2g131785_t01 0

grmzm2g131793_t01 0

grmzm2g131815_t02 0

grmzm2g131817_t02 0

grmzm2g131820_t01 0

grmzm2g131836_t02 0

grmzm2g131839_t01 0

grmzm2g131853_t01 0

grmzm2g131907_t01 0

grmzm2g131912_t02 0

grmzm2g131939_t01 0

grmzm2g131943_t01 0

grmzm2g131957_t01 0

grmzm2g131961_t03 0

grmzm2g131969_t02 0

grmzm2g131982_t01 -1.36

grmzm2g131988_t03 0

grmzm2g131998_t05 0

grmzm2g132000_t01 0

grmzm2g132009_t01 0

grmzm2g132019_t03 0

grmzm2g132021_t04 0

grmzm2g132032_t03 0

grmzm2g132060_t02 0

grmzm2g132069_t02 0

grmzm2g132093_t03 0

grmzm2g132101_t01 0

grmzm2g132121_t01 0

grmzm2g132130_t01 0

grmzm2g132140_t01 0

grmzm2g132169_t02 0

grmzm2g132184_t03 0

grmzm2g132185_t01 0

grmzm2g132212_t01 0

grmzm2g132218_t02 0

grmzm2g132251_t01 0

grmzm2g132301_t02 0

grmzm2g132303_t01 -1

grmzm2g132331_t01 -1.35

grmzm2g132367_t02 -1.36

grmzm2g132373_t01 0

grmzm2g132403_t01 0

grmzm2g132431_t01 0

grmzm2g132442_t03 -1.23

grmzm2g132450_t01 0

grmzm2g132461_t01 -0.97

grmzm2g132464_t01 0

grmzm2g132465_t01 0

grmzm2g132504_t04 0

grmzm2g132506_t01 0

grmzm2g132519_t03 0

grmzm2g132547_t03 0

grmzm2g132568_t01 0

grmzm2g132577_t01 0

grmzm2g132591_t03 0

grmzm2g132593_t03 0

grmzm2g132607_t03 0

grmzm2g132623_t02 0

grmzm2g132628_t01 0

grmzm2g132633_t04 0

grmzm2g132636_t01 0

grmzm2g132640_t01 0

grmzm2g132644_t01 0

grmzm2g132653_t02 0

grmzm2g132686_t01 0

grmzm2g132704_t01 0

grmzm2g132748_t01 0

grmzm2g132749_t02 0

grmzm2g132759_t05 0

grmzm2g132774_t01 0

grmzm2g132777_t01 0

grmzm2g132780_t01 0

grmzm2g132794_t01 0

grmzm2g132796_t02 0

grmzm2g132804_t01 0

grmzm2g132811_t01 0

grmzm2g132847_t01 0

grmzm2g132854_t01 0

grmzm2g132857_t01 0

grmzm2g132866_t01 0

grmzm2g132898_t01 0

grmzm2g132903_t01 0

grmzm2g132924_t01 0

grmzm2g132929_t02 0

grmzm2g132934_t02 0

grmzm2g132936_t02 0

grmzm2g132944_t03 0

grmzm2g132956_t01 0

grmzm2g132958_t01 0

grmzm2g132968_t05 0

grmzm2g132971_t02 0

grmzm2g132978_t02 0

grmzm2g132991_t01 0

grmzm2g132994_t01 0

grmzm2g133012_t01 0

grmzm2g133016_t01 0

grmzm2g133018_t01 0

grmzm2g133021_t01 0

grmzm2g133023_t01 0

grmzm2g133028_t01 1.03

grmzm2g133029_t03 0

grmzm2g133048_t01 0

grmzm2g133050_t01 0

grmzm2g133055_t01 0

grmzm2g133082_t02 0

grmzm2g133121_t01 0

grmzm2g133173_t03 0

grmzm2g133175_t01 0

grmzm2g133189_t01 0

grmzm2g133203_t01 0

grmzm2g133213_t01 0

grmzm2g133226_t03 0

grmzm2g133282_t01 0

grmzm2g133302_t04 0

grmzm2g133308_t01 0

grmzm2g133314_t03 0

grmzm2g133358_t03 0

grmzm2g133359_t01 0

grmzm2g133396_t02 0

grmzm2g133400_t03 0

grmzm2g133407_t03 0

grmzm2g133421_t01 0

grmzm2g133428_t02 0

grmzm2g133430_t01 0

grmzm2g133434_t01 -0.83

grmzm2g133444_t01 0

grmzm2g133464_t02 0

grmzm2g133475_t01 1.77

grmzm2g133483_t01 0

grmzm2g133552_t01 0

grmzm2g133558_t01 -1.29

grmzm2g133568_t01 1.84

grmzm2g133620_t01 0

grmzm2g133621_t01 0

grmzm2g133624_t01 0

grmzm2g133629_t03 0

grmzm2g133631_t01 0

grmzm2g133646_t01 0

grmzm2g133652_t02 0.84

grmzm2g133653_t01 0

grmzm2g133675_t01 0

grmzm2g133684_t03 0

grmzm2g133720_t01 0

grmzm2g133749_t01 0

grmzm2g133756_t01 0

grmzm2g133757_t01 0

grmzm2g133764_t01 0

grmzm2g133802_t02 0

grmzm2g133819_t02 0

grmzm2g133844_t01 0

grmzm2g133854_t01 0

grmzm2g133885_t02 0

grmzm2g133919_t01 0

grmzm2g133926_t01 0

grmzm2g133937_t01 0

grmzm2g133941_t01 0

grmzm2g133943_t01 0

grmzm2g133958_t02 0

grmzm2g133959_t01 0

grmzm2g134020_t01 0

grmzm2g134027_t01 0

grmzm2g134044_t01 0

grmzm2g134045_t01 0

grmzm2g134054_t02 0

grmzm2g134062_t01 0

grmzm2g134067_t01 0

grmzm2g134073_t02 0

grmzm2g134104_t01 0

grmzm2g134107_t01 0

grmzm2g134109_t02 0

grmzm2g134130_t02 0

grmzm2g134134_t01 0

grmzm2g134149_t02 0

grmzm2g134176_t03 0

grmzm2g134182_t01 0

grmzm2g134183_t02 0

grmzm2g134214_t01 0

grmzm2g134227_t03 0

grmzm2g134230_t01 0

grmzm2g134234_t01 0

grmzm2g134235_t01 -1.34

grmzm2g134251_t01 0

grmzm2g134256_t02 0

grmzm2g134260_t02 -0.81

grmzm2g134264_t01 0

grmzm2g134270_t02 0.92

grmzm2g134284_t01 0

grmzm2g134290_t01 0

grmzm2g134295_t01 0

grmzm2g134308_t02 -0.85

grmzm2g134313_t01 0

grmzm2g134329_t01 0

grmzm2g134340_t01 0

grmzm2g134341_t02 0

grmzm2g134367_t01 0

grmzm2g134382_t06 0

grmzm2g134385_t01 0

grmzm2g134389_t02 0

grmzm2g134396_t01 0.93

grmzm2g134398_t01 0

grmzm2g134424_t01 0

grmzm2g134426_t01 0

grmzm2g134430_t01 0

grmzm2g134439_t01 0

grmzm2g134476_t02 -0.87

grmzm2g134480_t01 0

grmzm2g134508_t02 0

grmzm2g134539_t02 0

grmzm2g134544_t01 0

grmzm2g134545_t01 0

grmzm2g134563_t01 0

grmzm2g134613_t01 0

grmzm2g134628_t01 0

grmzm2g134668_t03 0

grmzm2g134671_t01 0

grmzm2g134703_t01 -1.1

grmzm2g134708_t01 0

grmzm2g134711_t01 0

grmzm2g134731_t01 0

grmzm2g134738_t01 0

grmzm2g134747_t02 0

grmzm2g134749_t02 0

grmzm2g134753_t02 0

grmzm2g134756_t02 0

grmzm2g134759_t01 0

grmzm2g134761_t01 0

grmzm2g134770_t03 0

grmzm2g134797_t04 0

grmzm2g134806_t02 0

grmzm2g134825_t03 -1.13

grmzm2g134844_t01 0

grmzm2g134846_t01 0

grmzm2g134862_t02 0

grmzm2g134863_t02 0

grmzm2g134866_t01 0

grmzm2g134878_t01 0

grmzm2g134889_t02 0

grmzm2g134901_t01 0

grmzm2g134917_t01 0

grmzm2g134930_t02 0

grmzm2g134957_t01 0

grmzm2g134976_t01 0

grmzm2g134980_t02 0

grmzm2g134982_t01 0

grmzm2g134985_t01 0

grmzm2g135002_t01 0

grmzm2g135013_t01 0

grmzm2g135027_t02 0.96

grmzm2g135029_t03 0

grmzm2g135038_t01 0

grmzm2g135044_t01 0

grmzm2g135045_t02 0

grmzm2g135052_t01 0

grmzm2g135054_t01 0

grmzm2g135091_t01 -1.15

grmzm2g135095_t02 0

grmzm2g135108_t01 0.99

grmzm2g135120_t01 0

grmzm2g135132_t01 0

grmzm2g135165_t01 0

grmzm2g135186_t04 0

grmzm2g135195_t02 0

grmzm2g135199_t02 0

grmzm2g135236_t01 0

grmzm2g135256_t01 0

grmzm2g135283_t04 0

grmzm2g135300_t01 0.88

grmzm2g135320_t01 0

grmzm2g135322_t01 0

grmzm2g135341_t01 0

grmzm2g135354_t01 0

grmzm2g135359_t01 0

grmzm2g135362_t01 0

grmzm2g135400_t01 0

grmzm2g135410_t01 0

grmzm2g135446_t02 0

grmzm2g135470_t01 0

grmzm2g135476_t04 0

grmzm2g135498_t01 0

grmzm2g135501_t01 0

grmzm2g135506_t02 0

grmzm2g135588_t03 0

grmzm2g135599_t01 0

grmzm2g135617_t02 0

grmzm2g135651_t01 0

grmzm2g135654_t04 0

grmzm2g135655_t01 0

grmzm2g135688_t03 0

grmzm2g135691_t01 0

grmzm2g135722_t01 1.37

grmzm2g135727_t05 0

grmzm2g135739_t01 0

grmzm2g135743_t02 0

grmzm2g135756_t02 0

grmzm2g135763_t01 0

grmzm2g135770_t01 0

grmzm2g135778_t01 0

grmzm2g135782_t01 0

grmzm2g135790_t04 0

grmzm2g135795_t01 0

grmzm2g135808_t01 0

grmzm2g135816_t01 0

grmzm2g135817_t01 0

grmzm2g135839_t01 0

grmzm2g135877_t01 0

grmzm2g135893_t02 0

grmzm2g135904_t02 0

grmzm2g135909_t01 0.87

grmzm2g135935_t01 0

grmzm2g135940_t01 0

grmzm2g135949_t03 0

grmzm2g135968_t01 0

grmzm2g135970_t01 0

grmzm2g135978_t01 0

grmzm2g135990_t03 0

grmzm2g136016_t03 0

grmzm2g136042_t01 0

grmzm2g136058_t01 0

grmzm2g136067_t01 0

grmzm2g136072_t01 0

grmzm2g136076_t01 0

grmzm2g136081_t01 0

grmzm2g136085_t01 0

grmzm2g136106_t02 0

grmzm2g136113_t01 0

grmzm2g136139_t03 0

grmzm2g136143_t01 0

grmzm2g136146_t01 0

grmzm2g136158_t03 -2

grmzm2g136178_t02 0

grmzm2g136237_t01 0

grmzm2g136250_t01 0

grmzm2g136262_t01 0

grmzm2g136266_t02 0

grmzm2g136268_t02 0

grmzm2g136283_t02 0

grmzm2g136293_t01 0

grmzm2g136296_t03 0

grmzm2g136300_t01 0

grmzm2g136306_t01 0

grmzm2g136311_t01 0

grmzm2g136341_t01 0

grmzm2g136353_t01 -2.32

grmzm2g136364_t02 0

grmzm2g136366_t01 0

grmzm2g136367_t02 0

grmzm2g136369_t01 0

grmzm2g136389_t01 0

grmzm2g136412_t01 0

grmzm2g136427_t01 0

grmzm2g136443_t01 0

grmzm2g136453_t01 0

grmzm2g136469_t01 0

grmzm2g136486_t01 0

grmzm2g136494_t01 0

grmzm2g136495_t02 0

grmzm2g136508_t01 0

grmzm2g136513_t01 -3.51

grmzm2g136522_t04 0

grmzm2g136534_t02 -1.58

grmzm2g136563_t01 0

grmzm2g136580_t01 -0.87

grmzm2g136599_t01 1

grmzm2g136624_t01 0

grmzm2g136644_t01 0

grmzm2g136662_t01 0

grmzm2g136665_t01 0

grmzm2g136680_t01 0

grmzm2g136700_t01 0

grmzm2g136712_t01 0

grmzm2g136765_t01 0

grmzm2g136769_t02 0

grmzm2g136794_t01 0

grmzm2g136830_t01 0

grmzm2g136857_t02 0

grmzm2g136859_t02 0

grmzm2g136884_t01 -1.74

grmzm2g136887_t01 0

grmzm2g136889_t01 0

grmzm2g136895_t04 0

grmzm2g136901_t01 0

grmzm2g136910_t01 0

grmzm2g136918_t05 0

grmzm2g136960_t01 0

grmzm2g137029_t01 0

grmzm2g137046_t01 0.87

grmzm2g137064_t01 0

grmzm2g137077_t01 0

grmzm2g137108_t01 -0.88

grmzm2g137118_t02 0

grmzm2g137120_t02 0

grmzm2g137139_t01 0

grmzm2g137151_t02 0

grmzm2g137161_t01 0

grmzm2g137211_t01 0

grmzm2g137236_t01 0

grmzm2g137286_t01 0

grmzm2g137288_t01 0

grmzm2g137293_t01 1.18

grmzm2g137312_t01 0

grmzm2g137321_t01 0

grmzm2g137329_t01 0

grmzm2g137338_t01 0

grmzm2g137348_t02 0

grmzm2g137352_t02 -0.84

grmzm2g137358_t02 0

grmzm2g137366_t01 0

grmzm2g137375_t01 0

grmzm2g137409_t01 0

grmzm2g137413_t01 0

grmzm2g137426_t01 0

grmzm2g137435_t03 0

grmzm2g137451_t01 0

grmzm2g137468_t02 0

grmzm2g137471_t01 0

grmzm2g137479_t01 0

grmzm2g137495_t01 0

grmzm2g137498_t01 0

grmzm2g137528_t01 0

grmzm2g137535_t02 0

grmzm2g137546_t01 0

grmzm2g137558_t01 0

grmzm2g137561_t01 0

grmzm2g137569_t03 0

grmzm2g137574_t02 0

grmzm2g137582_t01 0

grmzm2g137593_t01 0

grmzm2g137596_t01 0

grmzm2g137673_t02 0

grmzm2g137696_t02 1.51

grmzm2g137704_t01 0

grmzm2g137707_t01 0

grmzm2g137710_t03 1.51

grmzm2g137715_t01 0

grmzm2g137724_t01 0

grmzm2g137775_t01 0

grmzm2g137788_t01 -0.98

grmzm2g137802_t01 0

grmzm2g137816_t03 0

grmzm2g137839_t01 0

grmzm2g137849_t01 0

grmzm2g137868_t05 0

grmzm2g137909_t01 -1.23

grmzm2g137930_t05 0

grmzm2g137944_t02 0.83

grmzm2g137947_t02 0

grmzm2g137961_t01 0

grmzm2g137964_t02 0

grmzm2g137965_t01 0

grmzm2g137968_t02 1.01

grmzm2g137984_t01 0

grmzm2g137985_t01 0

grmzm2g138012_t01 0

grmzm2g138041_t01 1.08

grmzm2g138053_t01 0

grmzm2g138060_t02 0

grmzm2g138067_t01 0

grmzm2g138074_t05 0

grmzm2g138076_t03 0

grmzm2g138077_t01 0

grmzm2g138097_t01 0

grmzm2g138103_t01 0

grmzm2g138125_t01 0

grmzm2g138128_t03 0

grmzm2g138152_t01 0

grmzm2g138161_t01 0

grmzm2g138178_t01 0

grmzm2g138190_t02 0

grmzm2g138220_t01 0

grmzm2g138230_t02 0

grmzm2g138255_t01 0

grmzm2g138258_t01 0

grmzm2g138268_t01 0

grmzm2g138303_t01 0

grmzm2g138338_t01 0

grmzm2g138342_t01 0

grmzm2g138349_t01 0

grmzm2g138382_t01 0

grmzm2g138407_t01 0

grmzm2g138419_t02 0

grmzm2g138421_t01 0

grmzm2g138423_t01 0

grmzm2g138425_t01 0

grmzm2g138429_t04 0

grmzm2g138441_t01 0

grmzm2g138455_t01 0

grmzm2g138475_t01 0

grmzm2g138494_t02 0

grmzm2g138496_t02 0

grmzm2g138511_t01 0

grmzm2g138527_t01 0

grmzm2g138550_t01 0

grmzm2g138572_t01 0

grmzm2g138583_t02 0

grmzm2g138585_t01 0

grmzm2g138589_t01 0

grmzm2g138659_t01 0

grmzm2g138676_t05 0

grmzm2g138710_t01 0

grmzm2g138750_t01 -1.76

grmzm2g138770_t01 0

grmzm2g138800_t01 0

grmzm2g138809_t01 0

grmzm2g138814_t02 0

grmzm2g138819_t01 0

grmzm2g138840_t01 0

grmzm2g138842_t01 0

grmzm2g138881_t03 1.08

grmzm2g138907_t01 0

grmzm2g138964_t04 0

grmzm2g138987_t03 0

grmzm2g138995_t02 0

grmzm2g139018_t02 0

grmzm2g139022_t02 0

grmzm2g139031_t01 0

grmzm2g139035_t02 0

grmzm2g139038_t01 0

grmzm2g139041_t01 0

grmzm2g139047_t02 0

grmzm2g139066_t01 0

grmzm2g139085_t02 0

grmzm2g139090_t01 0

grmzm2g139093_t01 0

grmzm2g139123_t01 0

grmzm2g139141_t01 0

grmzm2g139157_t01 0

grmzm2g139160_t01 0

grmzm2g139166_t01 0

grmzm2g139175_t01 0

grmzm2g139193_t01 0

grmzm2g139198_t01 0

grmzm2g139210_t04 0

grmzm2g139250_t02 -0.9

grmzm2g139296_t02 0

grmzm2g139300_t01 0

grmzm2g139329_t02 0

grmzm2g139336_t02 0

grmzm2g139341_t01 0

grmzm2g139349_t01 0

grmzm2g139360_t04 0

grmzm2g139363_t01 0

grmzm2g139369_t01 0

grmzm2g139372_t05 0

grmzm2g139374_t03 0

grmzm2g139407_t02 0

grmzm2g139419_t01 0

grmzm2g139441_t04 0

grmzm2g139462_t01 0

grmzm2g139463_t04 0

grmzm2g139467_t01 0

grmzm2g139512_t03 0

grmzm2g139533_t01 0

grmzm2g139574_t03 0

grmzm2g139583_t01 0

grmzm2g139600_t02 -0.99

grmzm2g139614_t01 0

grmzm2g139617_t02 0

grmzm2g139643_t03 0

grmzm2g139657_t01 0

grmzm2g139680_t01 0

grmzm2g139683_t03 0

grmzm2g139688_t01 0

grmzm2g139689_t02 0.96

grmzm2g139691_t01 0

grmzm2g139710_t01 0

grmzm2g139714_t01 0

grmzm2g139744_t02 0

grmzm2g139760_t01 0

grmzm2g139786_t01 0

grmzm2g139803_t01 0

grmzm2g139815_t02 0

grmzm2g139822_t01 0

grmzm2g139828_t02 0

grmzm2g139837_t01 0

grmzm2g139852_t02 0

grmzm2g139855_t01 0

grmzm2g139858_t01 0

grmzm2g139861_t01 0

grmzm2g139874_t01 0

grmzm2g139880_t01 0

grmzm2g139882_t01 0

grmzm2g139892_t01 0

grmzm2g139894_t02 0

grmzm2g139900_t01 0

grmzm2g139903_t01 0

grmzm2g139920_t01 0

grmzm2g139931_t01 0

grmzm2g139941_t04 0

grmzm2g139952_t01 0

grmzm2g139973_t02 0

grmzm2g139977_t01 0

grmzm2g140011_t01 0

grmzm2g140047_t01 0

grmzm2g140051_t01 0

grmzm2g140070_t01 0

grmzm2g140078_t01 0

grmzm2g140082_t01 0

grmzm2g140095_t04 0

grmzm2g140101_t01 -1.52

grmzm2g140107_t01 0

grmzm2g140116_t01 0

grmzm2g140156_t01 0

grmzm2g140160_t01 0

grmzm2g140174_t01 0

grmzm2g140179_t01 0

grmzm2g140201_t01 0

grmzm2g140231_t01 0

grmzm2g140288_t03 0

grmzm2g140299_t01 0

grmzm2g140328_t02 0

grmzm2g140342_t01 0

grmzm2g140352_t01 0

grmzm2g140362_t01 0

grmzm2g140394_t04 0

grmzm2g140432_t01 0

grmzm2g140443_t01 0

grmzm2g140451_t01 0

grmzm2g140455_t01 0

grmzm2g140474_t02 0

grmzm2g140500_t01 0

grmzm2g140537_t01 0

grmzm2g140545_t02 0

grmzm2g140559_t01 0

grmzm2g140561_t01 0

grmzm2g140577_t01 0

grmzm2g140587_t02 0

grmzm2g140590_t02 0

grmzm2g140602_t02 0

grmzm2g140609_t01 0

grmzm2g140612_t01 0

grmzm2g140614_t02 0

grmzm2g140635_t02 0

grmzm2g140651_t01 -0.83

grmzm2g140667_t03 0

grmzm2g140669_t01 0

grmzm2g140721_t01 0

grmzm2g140726_t01 0

grmzm2g140737_t01 0

grmzm2g140754_t01 0

grmzm2g140758_t01 1.08

grmzm2g140763_t02 0

grmzm2g140782_t01 0

grmzm2g140799_t02 0

grmzm2g140805_t01 0

grmzm2g140809_t02 0

grmzm2g140811_t01 0

grmzm2g140817_t01 0

grmzm2g140832_t01 0

grmzm2g140837_t01 0

grmzm2g140867_t01 0

grmzm2g140885_t02 0

grmzm2g140893_t02 0

grmzm2g140908_t01 0

grmzm2g140909_t01 0

grmzm2g140915_t01 -1.11

grmzm2g140917_t01 0

grmzm2g140924_t01 0

grmzm2g140970_t02 0

grmzm2g140994_t02 0

grmzm2g140996_t01 0

grmzm2g141002_t01 1.08

grmzm2g141009_t01 0

grmzm2g141026_t01 0

grmzm2g141185_t01 0

grmzm2g141209_t01 0

grmzm2g141214_t01 0

grmzm2g141216_t01 0

grmzm2g141219_t04 0

grmzm2g141222_t01 0

grmzm2g141229_t01 0

grmzm2g141241_t01 0

grmzm2g141273_t01 0

grmzm2g141277_t01 -1.09

grmzm2g141288_t02 -1.33

grmzm2g141289_t01 0

grmzm2g141299_t03 0

grmzm2g141325_t01 -2.07

grmzm2g141332_t01 0

grmzm2g141353_t01 0

grmzm2g141355_t01 0

grmzm2g141379_t01 0

grmzm2g141399_t04 0

grmzm2g141411_t01 0

grmzm2g141432_t01 0

grmzm2g141473_t01 0

grmzm2g141499_t01 0

grmzm2g141503_t02 0

grmzm2g141510_t01 0

grmzm2g141517_t01 0

grmzm2g141535_t01 0

grmzm2g141584_t01 0

grmzm2g141585_t01 0

grmzm2g141587_t01 0

grmzm2g141596_t01 0

grmzm2g141600_t03 0

grmzm2g141607_t02 0

grmzm2g141634_t02 0

grmzm2g141636_t01 0

grmzm2g141647_t01 0

grmzm2g141664_t01 0

grmzm2g141665_t02 0

grmzm2g141679_t01 0

grmzm2g141704_t02 0

grmzm2g141707_t02 0

grmzm2g141731_t03 0

grmzm2g141735_t01 0

grmzm2g141760_t01 0

grmzm2g141776_t01 0

grmzm2g141784_t01 0

grmzm2g141799_t01 0

grmzm2g141818_t02 0

grmzm2g141848_t02 0

grmzm2g141856_t01 0

grmzm2g141858_t01 0

grmzm2g141873_t01 0

grmzm2g141903_t01 0

grmzm2g141922_t01 -1

grmzm2g141925_t01 0

grmzm2g141931_t02 -1.18

grmzm2g141940_t02 0

grmzm2g141941_t02 0

grmzm2g141948_t01 0

grmzm2g141955_t04 0

grmzm2g141975_t01 0

grmzm2g141998_t02 0

grmzm2g142013_t01 0

grmzm2g142019_t01 0

grmzm2g142030_t01 0

grmzm2g142039_t01 0.86

grmzm2g142043_t02 0

grmzm2g142051_t02 0

grmzm2g142063_t02 0

grmzm2g142068_t01 0

grmzm2g142072_t01 0

grmzm2g142097_t03 0

grmzm2g142111_t01 0

grmzm2g142150_t02 0

grmzm2g142168_t01 0

grmzm2g142207_t01 0

grmzm2g142217_t01 0

grmzm2g142249_t01 0

grmzm2g142315_t02 0

grmzm2g142334_t01 0

grmzm2g142336_t03 0

grmzm2g142345_t02 0

grmzm2g142357_t01 0

grmzm2g142363_t04 0

grmzm2g142366_t01 0

grmzm2g142379_t01 0

grmzm2g142386_t01 0

grmzm2g142409_t02 0

grmzm2g142413_t01 0

grmzm2g142437_t02 0

grmzm2g142443_t01 0

grmzm2g142456_t01 0

grmzm2g142502_t03 0

grmzm2g142507_t01 0

grmzm2g142544_t01 0

grmzm2g142549_t01 0

grmzm2g142553_t01 0

grmzm2g142557_t01 0

grmzm2g142565_t01 0

grmzm2g142597_t03 0

grmzm2g142609_t02 0

grmzm2g142620_t02 0

grmzm2g142640_t01 0

grmzm2g142660_t03 0

grmzm2g142661_t03 0

grmzm2g142664_t01 0

grmzm2g142667_t02 0

grmzm2g142690_t02 0

grmzm2g142705_t01 -0.97

grmzm2g142709_t01 0

grmzm2g142712_t01 0

grmzm2g142735_t01 0

grmzm2g142740_t01 0

grmzm2g142751_t01 0

grmzm2g142757_t01 0

grmzm2g142768_t01 0

grmzm2g142777_t02 0

grmzm2g142779_t01 0

grmzm2g142802_t01 0

grmzm2g142806_t01 0

grmzm2g142820_t01 0

grmzm2g142825_t05 0

grmzm2g142836_t01 0

grmzm2g142850_t02 0

grmzm2g142863_t01 0

grmzm2g142870_t03 0

grmzm2g142873_t02 0

grmzm2g142875_t01 0

grmzm2g142898_t01 0

grmzm2g142913_t01 0

grmzm2g142918_t01 0

grmzm2g142919_t01 0

grmzm2g142922_t01 0

grmzm2g142932_t03 0

grmzm2g142984_t02 0

grmzm2g143029_t01 0

grmzm2g143071_t02 0

grmzm2g143102_t01 0

grmzm2g143128_t01 0

grmzm2g143139_t01 0

grmzm2g143153_t01 0

grmzm2g143160_t01 0

grmzm2g143165_t01 0

grmzm2g143168_t01 0

grmzm2g143173_t01 0

grmzm2g143202_t02 0

grmzm2g143205_t03 0

grmzm2g143211_t02 0

grmzm2g143213_t02 1.15

grmzm2g143234_t03 0

grmzm2g143235_t01 0

grmzm2g143242_t01 0

grmzm2g143244_t02 1.2

grmzm2g143246_t01 0

grmzm2g143258_t02 -1.89

grmzm2g143330_t02 0

grmzm2g143354_t01 0

grmzm2g143357_t01 0

grmzm2g143373_t01 2.03

grmzm2g143377_t01 0

grmzm2g143392_t01 0

grmzm2g143400_t01 -1.78

grmzm2g143402_t03 0

grmzm2g143403_t01 0

grmzm2g143443_t04 0

grmzm2g143445_t01 0

grmzm2g143450_t01 0

grmzm2g143460_t01 0

grmzm2g143462_t05 0

grmzm2g143469_t01 0

grmzm2g143480_t04 0

grmzm2g143494_t01 -1.58

grmzm2g143499_t01 0

grmzm2g143502_t01 0

grmzm2g143512_t01 0

grmzm2g143525_t02 0

grmzm2g143535_t01 0

grmzm2g143568_t01 0

grmzm2g143588_t02 0

grmzm2g143590_t02 0

grmzm2g143591_t01 0

grmzm2g143602_t01 0

grmzm2g143613_t01 0

grmzm2g143625_t01 0

grmzm2g143627_t01 0

grmzm2g143644_t01 0

grmzm2g143646_t01 0

grmzm2g143651_t01 0

grmzm2g143655_t01 0

grmzm2g143666_t01 0

grmzm2g143703_t02 0

grmzm2g143718_t01 0

grmzm2g143723_t01 0

grmzm2g143725_t01 0

grmzm2g143747_t01 0

grmzm2g143756_t01 0

grmzm2g143765_t01 0

grmzm2g143767_t01 0

grmzm2g143777_t01 0.92

grmzm2g143780_t01 0

grmzm2g143782_t01 0

grmzm2g143788_t01 0

grmzm2g143854_t03 0

grmzm2g143862_t01 0

grmzm2g143870_t02 0

grmzm2g143883_t06 1.07

grmzm2g143917_t01 0

grmzm2g143955_t02 0

grmzm2g143998_t01 0

grmzm2g144008_t01 0

grmzm2g144020_t02 0

grmzm2g144021_t02 0

grmzm2g144028_t02 0

grmzm2g144030_t03 0

grmzm2g144042_t02 0

grmzm2g144051_t01 0

grmzm2g144083_t01 0

grmzm2g144088_t01 0

grmzm2g144097_t02 0

grmzm2g144101_t03 0

grmzm2g144109_t01 0

grmzm2g144146_t01 0

grmzm2g144155_t01 0

grmzm2g144166_t01 0

grmzm2g144172_t01 0

grmzm2g144173_t01 0

grmzm2g144180_t02 0

grmzm2g144196_t02 0

grmzm2g144224_t01 0

grmzm2g144254_t07 0

grmzm2g144273_t01 0

grmzm2g144275_t04 0

grmzm2g144282_t01 0

grmzm2g144346_t01 -1.48

grmzm2g144362_t04 0

grmzm2g144367_t01 0

grmzm2g144372_t03 0

grmzm2g144387_t02 0

grmzm2g144403_t01 0

grmzm2g144420_t01 0

grmzm2g144421_t01 -2.82

grmzm2g144483_t02 0

grmzm2g144501_t01 0

grmzm2g144581_t04 0

grmzm2g144615_t02 0

grmzm2g144618_t02 0

grmzm2g144621_t01 0

grmzm2g144635_t01 0

grmzm2g144638_t01 0

grmzm2g144645_t03 0

grmzm2g144648_t01 0

grmzm2g144653_t04 0

grmzm2g144665_t01 0

grmzm2g144668_t01 0

grmzm2g144705_t01 0

grmzm2g144707_t01 0

grmzm2g144716_t02 0

grmzm2g144726_t01 0

grmzm2g144730_t01 0

grmzm2g144742_t02 0

grmzm2g144744_t01 -0.97

grmzm2g144764_t01 0

grmzm2g144782_t01 -1.44

grmzm2g144815_t01 0

grmzm2g144818_t01 0

grmzm2g144821_t01 0

grmzm2g144841_t02 0

grmzm2g144843_t01 0

grmzm2g144857_t01 0

grmzm2g144868_t02 0

grmzm2g144873_t02 0

grmzm2g144890_t01 0

grmzm2g144950_t01 0

grmzm2g144971_t01 0

grmzm2g144982_t01 0

grmzm2g144985_t03 0

grmzm2g144995_t04 0

grmzm2g145008_t01 0

grmzm2g145024_t03 1.01

grmzm2g145027_t01 0

grmzm2g145029_t01 0

grmzm2g145034_t02 0

grmzm2g145041_t02 2.53

grmzm2g145063_t01 0

grmzm2g145088_t01 0

grmzm2g145101_t03 0

grmzm2g145104_t01 0

grmzm2g145107_t02 0

grmzm2g145112_t01 0

grmzm2g145130_t01 0

grmzm2g145133_t04 0

grmzm2g145146_t01 0

grmzm2g145152_t01 0

grmzm2g145175_t03 0

grmzm2g145201_t01 0

grmzm2g145213_t01 0

grmzm2g145226_t02 0

grmzm2g145236_t06 0

grmzm2g145242_t02 -1.36

grmzm2g145256_t01 0

grmzm2g145258_t01 0

grmzm2g145280_t01 0

grmzm2g145300_t01 0

grmzm2g145330_t01 0

grmzm2g145346_t01 0

grmzm2g145360_t01 0

grmzm2g145370_t01 0

grmzm2g145374_t01 0

grmzm2g145396_t02 0

grmzm2g145444_t01 -1.99

grmzm2g145449_t02 0

grmzm2g145451_t02 0

grmzm2g145460_t01 1.32

grmzm2g145461_t01 0.82

grmzm2g145473_t01 0

grmzm2g145482_t03 0

grmzm2g145496_t02 0

grmzm2g145500_t04 0

grmzm2g145504_t01 0

grmzm2g145518_t01 -1.2

grmzm2g145527_t01 0

grmzm2g145556_t01 0

grmzm2g145573_t01 0

grmzm2g145578_t02 0

grmzm2g145579_t01 -1.25

grmzm2g145589_t01 0

grmzm2g145594_t02 0

grmzm2g145595_t01 0

grmzm2g145626_t01 0

grmzm2g145632_t01 0

grmzm2g145633_t01 0

grmzm2g145651_t01 0

grmzm2g145662_t02 0

grmzm2g145666_t01 0

grmzm2g145690_t01 -1.21

grmzm2g145698_t01 0

grmzm2g145699_t01 0

grmzm2g145709_t01 0

grmzm2g145715_t01 0

grmzm2g145718_t02 0

grmzm2g145720_t02 0

grmzm2g145752_t02 0

grmzm2g145758_t01 0

grmzm2g145775_t01 0

grmzm2g145794_t03 0

grmzm2g145805_t01 0

grmzm2g145814_t04 0

grmzm2g145816_t02 0

grmzm2g145821_t01 0

grmzm2g145825_t02 0

grmzm2g145827_t02 0

grmzm2g145840_t01 0

grmzm2g145850_t01 0

grmzm2g145854_t01 0

grmzm2g145870_t01 0

grmzm2g145879_t01 0

grmzm2g145890_t01 0

grmzm2g145895_t03 0

grmzm2g145905_t02 0

grmzm2g145914_t02 0

grmzm2g145935_t01 0

grmzm2g145944_t02 0

grmzm2g145951_t01 0

grmzm2g145958_t04 0

grmzm2g145962_t01 0

grmzm2g145968_t02 0

grmzm2g145972_t02 0

grmzm2g145996_t01 0

grmzm2g146000_t06 0

grmzm2g146004_t01 0

grmzm2g146012_t01 0

grmzm2g146020_t01 0

grmzm2g146028_t01 0

grmzm2g146034_t01 0

grmzm2g146041_t03 0

grmzm2g146111_t06 0

grmzm2g146115_t03 0

grmzm2g146118_t01 0

grmzm2g146152_t01 0

grmzm2g146161_t01 -1.42

grmzm2g146173_t02 0

grmzm2g146190_t01 0

grmzm2g146192_t01 0

grmzm2g146206_t03 0

grmzm2g146207_t01 0

grmzm2g146219_t01 0

grmzm2g146240_t02 0

grmzm2g146246_t01 0

grmzm2g146267_t02 0

grmzm2g146278_t03 0

grmzm2g146280_t02 0

grmzm2g146286_t02 0.81

grmzm2g146292_t04 0

grmzm2g146313_t02 0

grmzm2g146331_t01 0

grmzm2g146337_t01 0

grmzm2g146354_t01 0

grmzm2g146358_t03 0

grmzm2g146374_t01 0

grmzm2g146378_t01 0

grmzm2g146395_t01 0

grmzm2g146416_t01 0.88

grmzm2g146437_t01 0

grmzm2g146446_t01 0

grmzm2g146463_t01 -1.36

grmzm2g146472_t01 0

grmzm2g146475_t01 0

grmzm2g146486_t01 0

grmzm2g146490_t01 0

grmzm2g146514_t03 0

grmzm2g146536_t01 0

grmzm2g146553_t04 0

grmzm2g146589_t05 0

grmzm2g146599_t05 0

grmzm2g146614_t01 0

grmzm2g146616_t01 1.06

grmzm2g146627_t02 -1.69

grmzm2g146670_t01 0

grmzm2g146673_t01 0

grmzm2g146677_t03 0

grmzm2g146697_t03 0

grmzm2g146720_t01 0

grmzm2g146750_t01 0

grmzm2g146760_t01 0

grmzm2g146761_t01 0

grmzm2g146786_t01 0

grmzm2g146819_t01 0

grmzm2g146847_t01 0

grmzm2g146862_t02 0

grmzm2g146878_t01 0

grmzm2g146885_t01 0

grmzm2g146887_t01 0

grmzm2g146951_t01 0

grmzm2g146965_t01 0

grmzm2g146981_t01 0

grmzm2g147014_t01 0

grmzm2g147046_t01 0

grmzm2g147051_t01 -1.34

grmzm2g147056_t01 0

grmzm2g147123_t01 0

grmzm2g147128_t01 0

grmzm2g147145_t03 0

grmzm2g147158_t01 0

grmzm2g147191_t01 0

grmzm2g147199_t01 0

grmzm2g147213_t01 0

grmzm2g147243_t02 0

grmzm2g147245_t01 0

grmzm2g147256_t02 0

grmzm2g147266_t01 0

grmzm2g147268_t02 0

grmzm2g147279_t01 -0.95

grmzm2g147288_t01 0

grmzm2g147355_t01 0

grmzm2g147377_t02 0

grmzm2g147390_t01 0

grmzm2g147402_t01 0

grmzm2g147418_t01 0

grmzm2g147420_t01 0

grmzm2g147422_t01 0

grmzm2g147430_t02 0

grmzm2g147446_t02 0

grmzm2g147450_t01 0

grmzm2g147454_t01 0

grmzm2g147459_t02 0

grmzm2g147481_t01 2.02

grmzm2g147500_t06 0

grmzm2g147534_t09 0

grmzm2g147544_t04 -0.9

grmzm2g147579_t01 0

grmzm2g147587_t01 0

grmzm2g147603_t01 0

grmzm2g147614_t05 0

grmzm2g147619_t03 0

grmzm2g147623_t02 0

grmzm2g147659_t01 0

grmzm2g147667_t01 0

grmzm2g147671_t01 0

grmzm2g147687_t01 0

grmzm2g147698_t01 0

grmzm2g147709_t01 0

grmzm2g147712_t01 0

grmzm2g147721_t02 0

grmzm2g147726_t02 0

grmzm2g147756_t01 -1.27

grmzm2g147766_t02 0

grmzm2g147770_t01 0

grmzm2g147772_t01 0

grmzm2g147775_t03 0

grmzm2g147780_t02 0

grmzm2g147787_t01 1.66

grmzm2g147800_t01 0

grmzm2g147809_t01 0

grmzm2g147811_t01 0

grmzm2g147814_t02 0

grmzm2g147819_t01 0

grmzm2g147844_t01 0

grmzm2g147849_t01 0

grmzm2g147856_t02 0

grmzm2g147857_t01 0

grmzm2g147862_t01 0

grmzm2g147867_t01 0

grmzm2g147869_t01 1.16

grmzm2g147880_t01 0

grmzm2g147882_t03 0

grmzm2g147884_t01 0

grmzm2g147885_t01 0

grmzm2g147894_t01 0

grmzm2g147920_t01 0

grmzm2g147925_t01 0

grmzm2g147942_t01 -0.99

grmzm2g147961_t01 1.57

grmzm2g147966_t01 -1.24

grmzm2g148022_t01 -0.87

grmzm2g148066_t01 0

grmzm2g148078_t02 -2.35

grmzm2g148090_t03 0

grmzm2g148098_t01 0.9

grmzm2g148099_t01 0

grmzm2g148106_t01 0

grmzm2g148115_t01 0

grmzm2g148118_t01 0

grmzm2g148130_t02 0

grmzm2g148133_t01 0

grmzm2g148158_t03 0

grmzm2g148176_t02 0

grmzm2g148180_t02 0

grmzm2g148194_t07 0

grmzm2g148198_t01 0

grmzm2g148200_t01 0

grmzm2g148213_t01 0

grmzm2g148216_t03 0

grmzm2g148229_t02 0

grmzm2g148270_t01 0

grmzm2g148281_t01 0

grmzm2g148301_t01 0

grmzm2g148323_t01 0

grmzm2g148333_t01 0

grmzm2g148370_t01 0

grmzm2g148387_t01 0

grmzm2g148400_t01 -1.46

grmzm2g148411_t01 0

grmzm2g148441_t01 0

grmzm2g148453_t01 0

grmzm2g148460_t01 -1.03

grmzm2g148492_t01 0

grmzm2g148538_t01 0

grmzm2g148555_t01 0

grmzm2g148586_t01 0

grmzm2g148594_t01 0

grmzm2g148621_t02 0

grmzm2g148633_t02 0

grmzm2g148675_t01 -1.06

grmzm2g148704_t01 0

grmzm2g148709_t01 0

grmzm2g148723_t01 0

grmzm2g148744_t01 0

grmzm2g148751_t01 0

grmzm2g148758_t01 0

grmzm2g148769_t01 0

grmzm2g148772_t01 0

grmzm2g148773_t01 0

grmzm2g148790_t02 0

grmzm2g148796_t01 1.1

grmzm2g148807_t01 0

grmzm2g148810_t03 0

grmzm2g148811_t01 0

grmzm2g148831_t01 0

grmzm2g148864_t01 0

grmzm2g148867_t02 0

grmzm2g148872_t01 0

grmzm2g148884_t02 -0.96

grmzm2g148896_t01 0

grmzm2g148904_t02 0

grmzm2g148908_t03 -1.48

grmzm2g148913_t02 0

grmzm2g148924_t01 0

grmzm2g148925_t01 0

grmzm2g148937_t01 0

grmzm2g148962_t02 0

grmzm2g148985_t01 0

grmzm2g149028_t01 0

grmzm2g149031_t01 0

grmzm2g149040_t02 0

grmzm2g149105_t02 0

grmzm2g149108_t02 0

grmzm2g149115_t01 0

grmzm2g149132_t01 0

grmzm2g149135_t01 0

grmzm2g149138_t01 0

grmzm2g149153_t01 0

grmzm2g149175_t07 0

grmzm2g149178_t01 0

grmzm2g149201_t01 0

grmzm2g149211_t02 0

grmzm2g149224_t01 0

grmzm2g149230_t01 0

grmzm2g149236_t01 0

grmzm2g149238_t01 0

grmzm2g149257_t01 0

grmzm2g149265_t02 0

grmzm2g149272_t01 0

grmzm2g149281_t01 0

grmzm2g149289_t01 0

grmzm2g149313_t01 0

grmzm2g149315_t01 0

grmzm2g149317_t02 0

grmzm2g149321_t01 0

grmzm2g149323_t01 0

grmzm2g149326_t01 -1.51

grmzm2g149383_t02 0

grmzm2g149406_t01 0

grmzm2g149414_t01 0

grmzm2g149422_t01 0

grmzm2g149428_t03 0

grmzm2g149442_t02 0

grmzm2g149480_t02 0

grmzm2g149520_t03 0

grmzm2g149534_t01 0

grmzm2g149535_t01 0

grmzm2g149543_t01 0

grmzm2g149553_t01 0

grmzm2g149556_t01 0

grmzm2g149567_t02 0

grmzm2g149576_t02 0

grmzm2g149580_t01 0

grmzm2g149617_t01 0

grmzm2g149619_t01 0

grmzm2g149636_t01 0

grmzm2g149639_t02 0

grmzm2g149649_t01 0

grmzm2g149662_t02 0

grmzm2g149704_t01 0

grmzm2g149708_t01 0

grmzm2g149717_t01 0

grmzm2g149739_t01 0

grmzm2g149751_t03 0

grmzm2g149756_t01 0

grmzm2g149761_t01 0

grmzm2g149768_t01 0

grmzm2g149775_t01 0

grmzm2g149786_t01 0

grmzm2g149790_t01 0

grmzm2g149796_t01 0

grmzm2g149798_t01 -1.02

grmzm2g149800_t01 0

grmzm2g149802_t03 1.41

grmzm2g149808_t02 0

grmzm2g149809_t01 -1.27

grmzm2g149837_t01 0

grmzm2g149843_t02 0

grmzm2g149847_t01 0

grmzm2g149865_t03 1.37

grmzm2g149894_t01 0

grmzm2g149903_t02 0

grmzm2g149905_t01 0

grmzm2g149932_t02 0

grmzm2g149935_t01 0

grmzm2g149946_t01 0

grmzm2g149952_t01 0

grmzm2g149958_t01 0

grmzm2g149975_t03 0

grmzm2g149977_t01 0

grmzm2g149979_t01 0

grmzm2g149994_t04 0

grmzm2g150004_t01 0

grmzm2g150014_t02 0

grmzm2g150024_t02 0

grmzm2g150030_t01 0

grmzm2g150058_t03 0

grmzm2g150098_t01 0

grmzm2g150160_t01 0

grmzm2g150166_t01 0

grmzm2g150169_t01 0

grmzm2g150183_t01 0

grmzm2g150187_t01 0

grmzm2g150190_t03 0

grmzm2g150193_t01 0

grmzm2g150201_t01 0

grmzm2g150209_t02 0

grmzm2g150212_t01 0

grmzm2g150213_t04 0

grmzm2g150248_t02 0

grmzm2g150251_t02 0

grmzm2g150256_t01 -0.98

grmzm2g150260_t01 0

grmzm2g150262_t01 0

grmzm2g150264_t01 0

grmzm2g150286_t04 0

grmzm2g150295_t01 0

grmzm2g150302_t01 0

grmzm2g150319_t01 0

grmzm2g150323_t02 0

grmzm2g150337_t02 0

grmzm2g150363_t01 -0.83

grmzm2g150367_t01 0

grmzm2g150374_t02 0

grmzm2g150383_t01 0

grmzm2g150406_t01 0

grmzm2g150448_t01 0

grmzm2g150450_t01 0

grmzm2g150471_t05 0

grmzm2g150478_t02 0

grmzm2g150484_t01 0

grmzm2g150485_t05 0

grmzm2g150496_t01 0

grmzm2g150503_t01 0

grmzm2g150521_t01 0

grmzm2g150541_t01 0

grmzm2g150598_t01 0

grmzm2g150608_t01 0

grmzm2g150616_t01 0

grmzm2g150631_t02 0

grmzm2g150648_t07 0

grmzm2g150656_t02 0

grmzm2g150681_t01 0

grmzm2g150683_t04 0

grmzm2g150691_t01 0

grmzm2g150697_t01 0

grmzm2g150714_t01 0

grmzm2g150726_t01 0

grmzm2g150736_t01 0

grmzm2g150754_t02 0

grmzm2g150755_t01 0

grmzm2g150758_t02 0

grmzm2g150762_t02 0

grmzm2g150772_t02 0

grmzm2g150791_t01 0

grmzm2g150796_t03 0

grmzm2g150806_t02 0

grmzm2g150813_t02 0

grmzm2g150834_t01 0

grmzm2g150838_t01 0

grmzm2g150841_t01 0

grmzm2g150864_t01 0

grmzm2g150866_t02 0

grmzm2g150867_t01 0

grmzm2g150893_t03 0

grmzm2g150901_t02 0.84

grmzm2g150906_t01 0

grmzm2g150912_t01 0

grmzm2g150932_t03 0

grmzm2g150934_t01 0

grmzm2g150941_t03 0

grmzm2g150950_t03 0

grmzm2g150962_t02 0

grmzm2g151009_t02 0

grmzm2g151041_t05 0

grmzm2g151044_t01 0

grmzm2g151087_t02 0

grmzm2g151125_t01 0

grmzm2g151169_t01 0

grmzm2g151193_t01 0

grmzm2g151195_t01 0

grmzm2g151200_t03 0

grmzm2g151223_t01 0

grmzm2g151227_t01 0

grmzm2g151236_t02 0

grmzm2g151245_t01 0

grmzm2g151252_t03 0

grmzm2g151254_t02 0

grmzm2g151257_t01 0

grmzm2g151285_t02 0

grmzm2g151295_t03 0

grmzm2g151299_t02 0

grmzm2g151319_t04 -1.13

grmzm2g151332_t01 0

grmzm2g151387_t01 0

grmzm2g151406_t01 0

grmzm2g151407_t03 0

grmzm2g151414_t01 0

grmzm2g151418_t01 0

grmzm2g151434_t01 0

grmzm2g151440_t01 0

grmzm2g151476_t01 -1.56

grmzm2g151496_t01 0

grmzm2g151516_t01 -1.17

grmzm2g151519_t02 0

grmzm2g151521_t01 0

grmzm2g151529_t01 0

grmzm2g151536_t01 0

grmzm2g151549_t02 0

grmzm2g151564_t01 0

grmzm2g151567_t01 0

grmzm2g151576_t01 0

grmzm2g151580_t01 0

grmzm2g151582_t01 0

grmzm2g151589_t02 0

grmzm2g151614_t04 0

grmzm2g151616_t01 0

grmzm2g151639_t01 0

grmzm2g151649_t01 0

grmzm2g151651_t02 0

grmzm2g151653_t02 0

grmzm2g151656_t01 0

grmzm2g151689_t04 0

grmzm2g151700_t01 0

grmzm2g151701_t01 0

grmzm2g151706_t02 0

grmzm2g151717_t01 0

grmzm2g151726_t01 0

grmzm2g151734_t01 0

grmzm2g151738_t01 0

grmzm2g151807_t03 0

grmzm2g151826_t02 0

grmzm2g151863_t04 0

grmzm2g151873_t02 0

grmzm2g151893_t02 0

grmzm2g151903_t03 0

grmzm2g151905_t01 0

grmzm2g151923_t03 0

grmzm2g151934_t01 0

grmzm2g151944_t02 0

grmzm2g151955_t01 0

grmzm2g151967_t01 0

grmzm2g151975_t01 0

grmzm2g151977_t01 0

grmzm2g151983_t01 0

grmzm2g151992_t01 -0.83

grmzm2g152007_t01 0

grmzm2g152025_t01 0

grmzm2g152040_t01 0

grmzm2g152056_t02 0

grmzm2g152057_t04 0

grmzm2g152059_t01 0

grmzm2g152105_t03 0

grmzm2g152111_t06 0

grmzm2g152120_t01 0

grmzm2g152126_t01 -1.3

grmzm2g152127_t01 0

grmzm2g152177_t01 0

grmzm2g152179_t01 0

grmzm2g152189_t01 0

grmzm2g152194_t01 0

grmzm2g152258_t01 0

grmzm2g152278_t01 0

grmzm2g152319_t01 0

grmzm2g152328_t02 0

grmzm2g152370_t01 0

grmzm2g152417_t01 0.97

grmzm2g152419_t01 0

grmzm2g152421_t01 0

grmzm2g152432_t01 0

grmzm2g152438_t05 0

grmzm2g152447_t01 0

grmzm2g152457_t02 0

grmzm2g152461_t01 0

grmzm2g152466_t03 0

grmzm2g152470_t02 0

grmzm2g152477_t03 0

grmzm2g152485_t06 0

grmzm2g152548_t01 0

grmzm2g152549_t01 0

grmzm2g152552_t02 0

grmzm2g152555_t02 0

grmzm2g152561_t01 0

grmzm2g152573_t01 0.81

grmzm2g152599_t02 0

grmzm2g152616_t01 0

grmzm2g152638_t01 0

grmzm2g152661_t01 0

grmzm2g152663_t01 0

grmzm2g152686_t03 0

grmzm2g152688_t02 0

grmzm2g152703_t01 0

grmzm2g152732_t03 0

grmzm2g152739_t01 0

grmzm2g152757_t01 0

grmzm2g152768_t01 0

grmzm2g152777_t02 0

grmzm2g152796_t03 -3.25

grmzm2g152801_t01 0.95

grmzm2g152808_t01 0

grmzm2g152827_t01 0

grmzm2g152853_t01 0

grmzm2g152877_t01 0

grmzm2g152888_t01 0

grmzm2g152889_t01 0

grmzm2g152895_t01 0

grmzm2g152908_t01 0

grmzm2g152919_t01 0

grmzm2g152921_t02 0

grmzm2g152925_t02 0

grmzm2g152929_t01 0

grmzm2g152955_t01 0

grmzm2g152962_t01 0

grmzm2g152963_t01 0

grmzm2g152975_t01 0

grmzm2g152984_t01 0

grmzm2g153017_t01 0

grmzm2g153058_t03 0

grmzm2g153068_t01 0

grmzm2g153075_t02 0

grmzm2g153087_t02 0

grmzm2g153103_t01 0

grmzm2g153119_t01 0

grmzm2g153138_t01 0

grmzm2g153144_t01 0

grmzm2g153150_t01 0

grmzm2g153162_t03 0

grmzm2g153178_t04 0

grmzm2g153181_t02 0

grmzm2g153184_t01 -1.05

grmzm2g153206_t01 0

grmzm2g153208_t01 0

grmzm2g153227_t01 0

grmzm2g153233_t01 0

grmzm2g153250_t01 0

grmzm2g153263_t02 0

grmzm2g153274_t02 0

grmzm2g153275_t01 0

grmzm2g153292_t08 0

grmzm2g153332_t01 0

grmzm2g153333_t01 0

grmzm2g153358_t01 0

grmzm2g153363_t02 0

grmzm2g153369_t02 0

grmzm2g153390_t01 0

grmzm2g153393_t03 0

grmzm2g153409_t04 0

grmzm2g153434_t03 0

grmzm2g153444_t01 0

grmzm2g153450_t01 0

grmzm2g153454_t01 0

grmzm2g153476_t05 0

grmzm2g153488_t03 0

grmzm2g153523_t01 0

grmzm2g153526_t04 0

grmzm2g153527_t01 -0.81

grmzm2g153536_t02 0

grmzm2g153541_t06 0

grmzm2g153569_t04 0

grmzm2g153582_t01 0

grmzm2g153594_t02 0

grmzm2g153602_t01 0

grmzm2g153611_t01 0

grmzm2g153615_t01 0

grmzm2g153648_t04 0

grmzm2g153672_t01 0

grmzm2g153675_t02 0

grmzm2g153683_t02 0

grmzm2g153704_t01 0

grmzm2g153706_t01 0

grmzm2g153714_t02 0

grmzm2g153722_t02 0

grmzm2g153745_t01 0

grmzm2g153760_t01 0

grmzm2g153766_t01 0

grmzm2g153769_t01 0

grmzm2g153792_t01 0

grmzm2g153797_t01 0

grmzm2g153815_t01 0

grmzm2g153823_t01 0

grmzm2g153877_t01 0

grmzm2g153887_t01 0

grmzm2g153899_t01 0

grmzm2g153920_t01 0

grmzm2g153924_t01 0

grmzm2g153945_t01 0

grmzm2g153949_t03 0

grmzm2g153969_t01 0

grmzm2g153991_t01 0

grmzm2g153999_t02 0

grmzm2g154007_t01 0

grmzm2g154029_t03 0

grmzm2g154036_t01 0

grmzm2g154063_t01 0

grmzm2g154078_t01 0

grmzm2g154087_t03 0

grmzm2g154090_t01 0

grmzm2g154093_t04 0

grmzm2g154124_t01 0

grmzm2g154149_t01 0

grmzm2g154156_t01 0

grmzm2g154169_t02 0

grmzm2g154178_t02 0

grmzm2g154211_t02 -0.93

grmzm2g154218_t01 0

grmzm2g154221_t02 0

grmzm2g154223_t02 0

grmzm2g154229_t02 0

grmzm2g154278_t01 0

grmzm2g154312_t03 0

grmzm2g154316_t03 0

grmzm2g154328_t01 0

grmzm2g154332_t01 0

grmzm2g154344_t02 0

grmzm2g154366_t01 0

grmzm2g154394_t01 0

grmzm2g154397_t01 0

grmzm2g154414_t01 0

grmzm2g154422_t01 0

grmzm2g154426_t01 0

grmzm2g154437_t01 0

grmzm2g154447_t01 0

grmzm2g154449_t02 -1.61

grmzm2g154460_t01 0

grmzm2g154463_t01 0

grmzm2g154487_t01 0

grmzm2g154489_t01 0

grmzm2g154499_t03 0

grmzm2g154505_t01 0

grmzm2g154508_t01 0

grmzm2g154509_t01 0

grmzm2g154523_t02 0

grmzm2g154532_t02 0

grmzm2g154549_t01 0

grmzm2g154565_t01 0

grmzm2g154574_t02 0

grmzm2g154578_t01 0

grmzm2g154595_t01 0

grmzm2g154621_t01 0

grmzm2g154628_t01 0

grmzm2g154641_t02 0

grmzm2g154648_t04 0

grmzm2g154664_t02 0

grmzm2g154678_t01 -1.65

grmzm2g154725_t01 0

grmzm2g154735_t01 0

grmzm2g154839_t01 0

grmzm2g154864_t01 0

grmzm2g154881_t02 0

grmzm2g154883_t02 0

grmzm2g154890_t03 0

grmzm2g154892_t01 0

grmzm2g154896_t01 0

grmzm2g154936_t05 0

grmzm2g154939_t02 0

grmzm2g154946_t01 0

grmzm2g154958_t01 0

grmzm2g154960_t01 0

grmzm2g154987_t01 0

grmzm2g155012_t01 0

grmzm2g155015_t01 0

grmzm2g155021_t01 0

grmzm2g155058_t01 0

grmzm2g155077_t01 0

grmzm2g155096_t08 0

grmzm2g155111_t03 0

grmzm2g155123_t04 0

grmzm2g155216_t01 -2.31

grmzm2g155217_t01 0

grmzm2g155220_t01 0

grmzm2g155232_t02 0

grmzm2g155242_t01 0

grmzm2g155253_t01 0

grmzm2g155281_t01 0

grmzm2g155285_t02 0

grmzm2g155312_t02 0

grmzm2g155317_t01 0

grmzm2g155321_t01 0

grmzm2g155323_t02 0

grmzm2g155329_t01 0

grmzm2g155332_t01 0

grmzm2g155337_t03 0

grmzm2g155341_t02 0

grmzm2g155348_t03 0

grmzm2g155357_t01 0.81

grmzm2g155370_t01 0

grmzm2g155375_t01 0

grmzm2g155384_t01 0

grmzm2g155387_t01 0

grmzm2g155422_t01 0

grmzm2g155437_t02 0

grmzm2g155512_t01 0

grmzm2g155543_t01 0

grmzm2g155546_t01 0

grmzm2g155580_t02 0

grmzm2g155593_t02 0

grmzm2g155626_t01 0

grmzm2g155642_t04 0

grmzm2g155662_t04 0

grmzm2g155709_t03 0

grmzm2g155729_t02 0

grmzm2g155743_t01 0

grmzm2g155753_t03 0

grmzm2g155762_t01 0

grmzm2g155767_t01 0

grmzm2g155776_t01 0

grmzm2g155783_t01 0

grmzm2g155836_t01 0

grmzm2g155837_t02 0

grmzm2g155849_t01 0

grmzm2g155868_t01 0

grmzm2g155877_t01 0

grmzm2g155889_t02 0

grmzm2g155899_t01 0

grmzm2g155911_t01 0

grmzm2g155931_t01 0

grmzm2g155935_t02 0

grmzm2g155943_t01 0

grmzm2g155954_t01 0

grmzm2g155974_t01 0

grmzm2g155991_t01 0

grmzm2g155998_t01 -1.04

grmzm2g156004_t01 0

grmzm2g156012_t01 0

grmzm2g156013_t02 0

grmzm2g156016_t01 0

grmzm2g156019_t01 0

grmzm2g156026_t01 0

grmzm2g156033_t01 0

grmzm2g156068_t02 0

grmzm2g156069_t01 0

grmzm2g156099_t01 0

grmzm2g156110_t02 0

grmzm2g156112_t01 0

grmzm2g156121_t01 0

grmzm2g156126_t01 0

grmzm2g156127_t01 0

grmzm2g156145_t01 0

grmzm2g156156_t01 0

grmzm2g156158_t02 0

grmzm2g156162_t03 0

grmzm2g156174_t01 0

grmzm2g156203_t02 0

grmzm2g156206_t01 0

grmzm2g156227_t01 0

grmzm2g156296_t01 0

grmzm2g156310_t01 0

grmzm2g156320_t01 0

grmzm2g156356_t01 0

grmzm2g156365_t05 0

grmzm2g156376_t02 0

grmzm2g156388_t02 0

grmzm2g156415_t06 0

grmzm2g156422_t01 0

grmzm2g156459_t01 0

grmzm2g156472_t03 0

grmzm2g156486_t03 0

grmzm2g156490_t01 0

grmzm2g156516_t03 0

grmzm2g156517_t01 0

grmzm2g156543_t01 0

grmzm2g156565_t01 0

grmzm2g156568_t01 0

grmzm2g156578_t02 0

grmzm2g156581_t02 0

grmzm2g156599_t01 0

grmzm2g156606_t01 0

grmzm2g156608_t06 0

grmzm2g156630_t03 0

grmzm2g156632_t01 0

grmzm2g156638_t01 0

grmzm2g156643_t01 0

grmzm2g156648_t05 0

grmzm2g156673_t02 0

grmzm2g156692_t01 0

grmzm2g156713_t01 0

grmzm2g156734_t01 0

grmzm2g156741_t01 0

grmzm2g156748_t01 2.37

grmzm2g156754_t01 0

grmzm2g156756_t01 0

grmzm2g156765_t02 0

grmzm2g156785_t01 0

grmzm2g156800_t02 0

grmzm2g156803_t01 0

grmzm2g156816_t01 0

grmzm2g156818_t01 0

grmzm2g156824_t02 0

grmzm2g156827_t01 0

grmzm2g156848_t04 0

grmzm2g156861_t02 -1.43

grmzm2g156879_t01 0

grmzm2g156890_t03 0

grmzm2g156904_t01 0

grmzm2g156905_t01 0

grmzm2g156910_t01 0

grmzm2g156937_t01 0

grmzm2g156950_t01 0

grmzm2g156956_t02 0

grmzm2g156960_t01 0

grmzm2g156986_t01 0

grmzm2g157007_t02 0

grmzm2g157010_t01 0

grmzm2g157018_t02 0

grmzm2g157019_t01 0

grmzm2g157027_t01 0

grmzm2g157034_t02 0.81

grmzm2g157043_t01 0

grmzm2g157061_t01 0

grmzm2g157102_t01 0

grmzm2g157113_t02 -0.95

grmzm2g157115_t01 0

grmzm2g157132_t01 0

grmzm2g157147_t01 0

grmzm2g157156_t01 0

grmzm2g157157_t01 0

grmzm2g157168_t01 0

grmzm2g157177_t01 0

grmzm2g157197_t01 0

grmzm2g157219_t02 0

grmzm2g157246_t01 0

grmzm2g157247_t01 0

grmzm2g157263_t01 0

grmzm2g157267_t01 0

grmzm2g157269_t01 0

grmzm2g157296_t02 0

grmzm2g157306_t02 0

grmzm2g157310_t03 0

grmzm2g157316_t02 0

grmzm2g157317_t03 0

grmzm2g157320_t03 0

grmzm2g157329_t03 0

grmzm2g157332_t06 0

grmzm2g157334_t04 0

grmzm2g157350_t01 0

grmzm2g157354_t03 0

grmzm2g157369_t01 0

grmzm2g157379_t01 0

grmzm2g157384_t01 0

grmzm2g157389_t01 0

grmzm2g157407_t01 0

grmzm2g157443_t01 0

grmzm2g157458_t01 0

grmzm2g157462_t01 0

grmzm2g157470_t01 0

grmzm2g157472_t03 0

grmzm2g157481_t01 1.03

grmzm2g157510_t01 0

grmzm2g157522_t01 0

grmzm2g157536_t03 0

grmzm2g157564_t02 0

grmzm2g157574_t02 0

grmzm2g157588_t01 0

grmzm2g157589_t01 0

grmzm2g157596_t03 0

grmzm2g157598_t01 0

grmzm2g157621_t01 0

grmzm2g157631_t01 0

grmzm2g157633_t01 0

grmzm2g157679_t01 0

grmzm2g157705_t01 0

grmzm2g157716_t01 0

grmzm2g157722_t03 0

grmzm2g157729_t01 0.86

grmzm2g157749_t01 1.71

grmzm2g157758_t02 0

grmzm2g157760_t01 -1.45

grmzm2g157812_t01 0

grmzm2g157817_t01 0

grmzm2g157855_t01 0

grmzm2g157875_t02 0

grmzm2g157878_t01 0

grmzm2g157879_t01 0

grmzm2g157913_t01 0

grmzm2g157925_t01 0

grmzm2g157953_t01 0

grmzm2g157990_t02 0

grmzm2g158013_t01 0

grmzm2g158021_t01 0

grmzm2g158024_t02 0

grmzm2g158029_t01 0

grmzm2g158034_t01 0

grmzm2g158043_t01 0

grmzm2g158045_t01 0

grmzm2g158069_t01 0

grmzm2g158083_t02 0

grmzm2g158091_t02 0

grmzm2g158097_t02 0

grmzm2g158117_t01 0

grmzm2g158130_t01 0

grmzm2g158131_t01 0

grmzm2g158136_t01 0

grmzm2g158141_t01 0

grmzm2g158147_t01 0

grmzm2g158153_t03 0

grmzm2g158162_t01 0

grmzm2g158179_t01 0

grmzm2g158197_t01 0

grmzm2g158205_t01 -0.98

grmzm2g158228_t02 0

grmzm2g158232_t01 0

grmzm2g158237_t05 0

grmzm2g158240_t01 -0.89

grmzm2g158248_t01 0

grmzm2g158252_t02 0.83

grmzm2g158261_t01 0

grmzm2g158277_t01 0

grmzm2g158281_t01 0

grmzm2g158328_t01 0

grmzm2g158359_t01 0

grmzm2g158384_t01 0

grmzm2g158394_t01 0

grmzm2g158434_t01 0

grmzm2g158437_t01 0

grmzm2g158447_t01 0

grmzm2g158452_t02 0.83

grmzm2g158462_t01 0

grmzm2g158479_t04 0

grmzm2g158489_t01 0

grmzm2g158496_t01 0

grmzm2g158515_t01 0

grmzm2g158538_t03 0

grmzm2g158562_t04 0

grmzm2g158568_t02 0

grmzm2g158575_t02 0

grmzm2g158595_t02 0

grmzm2g158609_t01 0

grmzm2g158627_t01 0

grmzm2g158629_t03 0

grmzm2g158645_t01 0

grmzm2g158662_t01 0

grmzm2g158676_t01 0

grmzm2g158679_t02 0

grmzm2g158734_t03 0

grmzm2g158746_t01 0

grmzm2g158766_t04 0

grmzm2g158789_t01 1.68

grmzm2g158809_t01 0

grmzm2g158811_t01 0

grmzm2g158835_t01 0

grmzm2g158839_t01 0

grmzm2g158860_t03 0

grmzm2g158872_t01 0

grmzm2g158885_t01 0

grmzm2g158887_t01 0

grmzm2g158901_t01 0

grmzm2g158911_t01 0

grmzm2g158972_t01 0

grmzm2g158976_t01 0

grmzm2g158985_t03 0

grmzm2g159013_t01 0

grmzm2g159016_t01 0

grmzm2g159028_t01 0

grmzm2g159032_t05 0

grmzm2g159034_t01 0

grmzm2g159041_t04 0

grmzm2g159047_t01 0

grmzm2g159049_t01 0

grmzm2g159082_t01 0

grmzm2g159105_t01 0

grmzm2g159110_t02 -2.46

grmzm2g159134_t03 0

grmzm2g159142_t01 0

grmzm2g159155_t01 0

grmzm2g159160_t01 0

grmzm2g159169_t01 0

grmzm2g159171_t02 0

grmzm2g159200_t01 -1.14

grmzm2g159218_t01 0

grmzm2g159221_t02 0

grmzm2g159237_t01 0

grmzm2g159250_t01 0

grmzm2g159285_t01 0

grmzm2g159295_t02 0

grmzm2g159330_t01 0

grmzm2g159357_t01 0

grmzm2g159364_t01 0

grmzm2g159369_t02 0

grmzm2g159388_t02 1.29

grmzm2g159389_t03 0

grmzm2g159399_t01 -0.83

grmzm2g159404_t01 0

grmzm2g159427_t01 0

grmzm2g159431_t02 -0.96

grmzm2g159475_t01 0

grmzm2g159477_t01 0

grmzm2g159498_t01 0

grmzm2g159500_t02 0

grmzm2g159535_t01 0

grmzm2g159538_t01 0

grmzm2g159542_t02 0

grmzm2g159547_t01 0

grmzm2g159554_t01 0

grmzm2g159581_t01 0

grmzm2g159587_t01 0

grmzm2g159632_t01 0

grmzm2g159641_t01 0

grmzm2g159643_t02 0

grmzm2g159660_t01 0

grmzm2g159675_t01 0

grmzm2g159678_t03 0

grmzm2g159691_t01 0

grmzm2g159700_t02 0

grmzm2g159715_t01 0

grmzm2g159720_t01 0

grmzm2g159724_t01 0

grmzm2g159732_t02 0

grmzm2g159741_t04 0

grmzm2g159744_t01 0

grmzm2g159756_t01 0

grmzm2g159759_t01 0

grmzm2g159766_t01 0

grmzm2g159768_t01 0

grmzm2g159777_t01 0

grmzm2g159824_t01 0

grmzm2g159828_t01 0

grmzm2g159849_t01 0

grmzm2g159854_t02 0

grmzm2g159890_t01 0

grmzm2g159901_t01 0

grmzm2g159908_t01 0

grmzm2g159924_t02 0

grmzm2g159926_t01 0

grmzm2g159935_t01 0

grmzm2g159937_t01 0

grmzm2g159956_t01 0

grmzm2g159975_t01 0

grmzm2g159992_t02 0

grmzm2g159996_t01 0

grmzm2g160005_t01 0

grmzm2g160013_t01 0

grmzm2g160032_t05 0

grmzm2g160041_t04 0

grmzm2g160046_t01 0

grmzm2g160064_t01 0

grmzm2g160069_t01 0

grmzm2g160149_t01 1.37

grmzm2g160174_t04 0

grmzm2g160184_t01 0

grmzm2g160198_t01 0

grmzm2g160211_t01 0

grmzm2g160231_t01 0

grmzm2g160237_t01 0

grmzm2g160268_t04 -1.42

grmzm2g160273_t05 0

grmzm2g160304_t09 0

grmzm2g160316_t02 0

grmzm2g160324_t04 0

grmzm2g160351_t01 0

grmzm2g160354_t01 0

grmzm2g160370_t01 0

grmzm2g160417_t01 0

grmzm2g160428_t02 0

grmzm2g160430_t04 0

grmzm2g160452_t02 0

grmzm2g160454_t01 0

grmzm2g160458_t01 0

grmzm2g160460_t03 0

grmzm2g160473_t02 1.62

grmzm2g160504_t02 0

grmzm2g160506_t06 0

grmzm2g160514_t01 0

grmzm2g160523_t01 0

grmzm2g160527_t01 0

grmzm2g160541_t03 -1.49

grmzm2g160556_t01 0

grmzm2g160560_t01 0

grmzm2g160563_t02 0

grmzm2g160569_t02 0

grmzm2g160585_t01 0

grmzm2g160609_t01 0

grmzm2g160619_t01 0

grmzm2g160702_t01 0

grmzm2g160710_t01 0

grmzm2g160730_t02 0

grmzm2g160752_t02 0

grmzm2g160770_t01 0

grmzm2g160801_t01 0

grmzm2g160838_t01 0

grmzm2g160842_t02 0

grmzm2g160862_t01 0

grmzm2g160863_t01 0

grmzm2g160887_t01 0

grmzm2g160902_t01 0

grmzm2g160922_t02 0

grmzm2g160925_t01 0

grmzm2g160927_t01 0

grmzm2g160958_t01 0

grmzm2g160983_t02 1.65

grmzm2g160990_t01 0

grmzm2g160994_t01 0

grmzm2g160996_t01 0

grmzm2g161009_t01 0

grmzm2g161019_t02 0

grmzm2g161040_t01 0

grmzm2g161087_t01 0

grmzm2g161097_t01 0

grmzm2g161102_t02 0

grmzm2g161121_t01 0

grmzm2g161146_t01 0

grmzm2g161154_t01 0

grmzm2g161168_t01 0

grmzm2g161175_t01 0

grmzm2g161212_t01 0

grmzm2g161222_t01 0

grmzm2g161242_t02 0

grmzm2g161244_t01 0

grmzm2g161245_t01 0

grmzm2g161274_t02 0

grmzm2g161285_t01 0.82

grmzm2g161291_t03 0

grmzm2g161295_t01 0

grmzm2g161299_t05 0

grmzm2g161302_t01 0

grmzm2g161306_t01 0

grmzm2g161315_t01 0

grmzm2g161335_t01 -0.83

grmzm2g161337_t02 0

grmzm2g161368_t01 0

grmzm2g161377_t01 0

grmzm2g161380_t01 0

grmzm2g161418_t03 0

grmzm2g161452_t01 0

grmzm2g161459_t01 0

grmzm2g161491_t03 0

grmzm2g161493_t01 0

grmzm2g161506_t01 0

grmzm2g161514_t01 0

grmzm2g161544_t01 0

grmzm2g161545_t01 0

grmzm2g161560_t01 0

grmzm2g161566_t03 0

grmzm2g161587_t01 0

grmzm2g161611_t01 0

grmzm2g161619_t03 0

grmzm2g161641_t04 0

grmzm2g161658_t01 0

grmzm2g161664_t01 0

grmzm2g161668_t03 0

grmzm2g161673_t01 0

grmzm2g161680_t01 0

grmzm2g161696_t01 0

grmzm2g161728_t01 0

grmzm2g161761_t01 0

grmzm2g161780_t01 0

grmzm2g161787_t01 0

grmzm2g161809_t01 0

grmzm2g161868_t01 0

grmzm2g161905_t01 0

grmzm2g161913_t01 0

grmzm2g161932_t04 0

grmzm2g161969_t01 0

grmzm2g161988_t03 0

grmzm2g162007_t03 0

grmzm2g162065_t01 0

grmzm2g162079_t05 0

grmzm2g162086_t02 0

grmzm2g162127_t01 0

grmzm2g162145_t01 0

grmzm2g162177_t01 0

grmzm2g162184_t01 0

grmzm2g162200_t01 0

grmzm2g162233_t01 0

grmzm2g162250_t01 0

grmzm2g162251_t01 0

grmzm2g162261_t01 0

grmzm2g162266_t01 0

grmzm2g162276_t01 0

grmzm2g162282_t01 0

grmzm2g162290_t01 0

grmzm2g162292_t02 0

grmzm2g162305_t01 -1.45

grmzm2g162329_t01 0

grmzm2g162347_t01 0

grmzm2g162353_t04 0

grmzm2g162356_t01 0

grmzm2g162359_t01 0

grmzm2g162369_t01 0

grmzm2g162382_t02 0

grmzm2g162388_t01 0

grmzm2g162405_t01 0

grmzm2g162409_t04 0

grmzm2g162413_t02 0.96

grmzm2g162434_t01 0.92

grmzm2g162443_t01 0

grmzm2g162450_t01 0

grmzm2g162451_t01 0

grmzm2g162453_t03 -1.11

grmzm2g162467_t01 0

grmzm2g162486_t01 0

grmzm2g162490_t01 0

grmzm2g162505_t01 0

grmzm2g162508_t02 -1.48

grmzm2g162525_t01 0

grmzm2g162527_t03 0

grmzm2g162529_t02 0

grmzm2g162531_t03 0

grmzm2g162535_t02 0

grmzm2g162537_t02 0

grmzm2g162544_t02 0

grmzm2g162567_t04 0

grmzm2g162598_t01 0

grmzm2g162605_t03 0

grmzm2g162616_t01 0

grmzm2g162637_t03 0

grmzm2g162640_t01 0

grmzm2g162663_t02 0

grmzm2g162668_t01 0

grmzm2g162670_t01 1.2

grmzm2g162672_t02 0

grmzm2g162688_t01 0

grmzm2g162690_t03 0

grmzm2g162699_t05 0

grmzm2g162725_t01 0

grmzm2g162727_t02 0

grmzm2g162737_t01 0

grmzm2g162748_t01 0

grmzm2g162749_t01 0

grmzm2g162755_t01 -0.84

grmzm2g162758_t01 0

grmzm2g162764_t02 0

grmzm2g162768_t03 0

grmzm2g162776_t03 0

grmzm2g162781_t01 0

grmzm2g162783_t01 0

grmzm2g162786_t02 0

grmzm2g162798_t01 0.9

grmzm2g162800_t03 0

grmzm2g162814_t03 0

grmzm2g162840_t01 0

grmzm2g162868_t03 0

grmzm2g162874_t01 0

grmzm2g162949_t01 0

grmzm2g162954_t01 0

grmzm2g162968_t01 0

grmzm2g162988_t01 0

grmzm2g162992_t02 0

grmzm2g163045_t01 0

grmzm2g163059_t01 0

grmzm2g163067_t01 -0.81

grmzm2g163072_t01 0

grmzm2g163081_t05 0

grmzm2g163086_t01 0

grmzm2g163129_t01 0

grmzm2g163136_t01 0

grmzm2g163138_t01 0

grmzm2g163141_t04 0

grmzm2g163154_t06 0

grmzm2g163157_t01 0

grmzm2g163189_t01 -1.19

grmzm2g163193_t04 0

grmzm2g163200_t01 0

grmzm2g163227_t01 0

grmzm2g163247_t02 0

grmzm2g163277_t01 0

grmzm2g163291_t02 0

grmzm2g163296_t01 0

grmzm2g163297_t02 0

grmzm2g163307_t02 0

grmzm2g163311_t01 0

grmzm2g163338_t01 0

grmzm2g163396_t01 0

grmzm2g163398_t01 0

grmzm2g163406_t07 -1.02

grmzm2g163407_t01 0

grmzm2g163418_t02 0

grmzm2g163421_t01 0

grmzm2g163427_t01 0

grmzm2g163437_t03 0

grmzm2g163440_t01 0

grmzm2g163471_t01 0

grmzm2g163473_t02 0

grmzm2g163476_t01 0

grmzm2g163514_t01 0

grmzm2g163544_t08 0

grmzm2g163550_t01 0

grmzm2g163561_t02 0

grmzm2g163578_t01 1.44

grmzm2g163599_t01 0

grmzm2g163641_t01 0

grmzm2g163658_t01 0

grmzm2g163671_t02 0

grmzm2g163709_t01 0

grmzm2g163713_t01 0

grmzm2g163717_t04 0

grmzm2g163724_t01 0

grmzm2g163726_t01 0

grmzm2g163749_t01 0

grmzm2g163751_t01 0

grmzm2g163769_t01 0

grmzm2g163798_t01 0

grmzm2g163809_t03 0

grmzm2g163813_t01 0

grmzm2g163826_t02 0

grmzm2g163848_t03 0

grmzm2g163849_t02 0

grmzm2g163861_t03 0

grmzm2g163877_t01 0

grmzm2g163888_t01 0

grmzm2g163905_t01 0

grmzm2g163912_t02 0

grmzm2g163914_t01 0

grmzm2g163925_t01 0

grmzm2g163939_t01 0

grmzm2g163988_t01 0

grmzm2g163996_t01 0

grmzm2g164018_t02 0

grmzm2g164020_t02 -1.03

grmzm2g164036_t01 0

grmzm2g164062_t02 0

grmzm2g164072_t01 0

grmzm2g164074_t01 0

grmzm2g164085_t01 0

grmzm2g164088_t01 0

grmzm2g164113_t02 0

grmzm2g164117_t02 0

grmzm2g164131_t01 0

grmzm2g164134_t02 0

grmzm2g164136_t01 0

grmzm2g164141_t02 0

grmzm2g164160_t02 0

grmzm2g164165_t03 0

grmzm2g164175_t03 0

grmzm2g164185_t01 0

grmzm2g164202_t01 0

grmzm2g164224_t01 0

grmzm2g164263_t02 0

grmzm2g164265_t01 0

grmzm2g164277_t03 0

grmzm2g164304_t03 0

grmzm2g164308_t03 0

grmzm2g164318_t01 0

grmzm2g164341_t01 0

grmzm2g164352_t03 0

grmzm2g164358_t01 0

grmzm2g164378_t02 -0.84

grmzm2g164400_t01 0

grmzm2g164401_t03 0

grmzm2g164413_t01 0

grmzm2g164418_t01 0

grmzm2g164426_t01 0

grmzm2g164428_t01 0

grmzm2g164440_t01 0

grmzm2g164470_t01 0

grmzm2g164475_t01 0.99

grmzm2g164489_t01 0

grmzm2g164493_t04 0

grmzm2g164502_t01 0

grmzm2g164527_t01 0

grmzm2g164538_t01 0

grmzm2g164547_t01 0

grmzm2g164558_t01 0

grmzm2g164562_t01 0

grmzm2g164563_t01 0

grmzm2g164580_t01 0

grmzm2g164622_t02 0

grmzm2g164649_t01 0

grmzm2g164654_t01 0

grmzm2g164663_t02 0

grmzm2g164669_t01 0

grmzm2g164696_t03 0

grmzm2g164714_t01 0

grmzm2g164735_t01 0

grmzm2g164743_t01 0

grmzm2g164759_t01 0

grmzm2g164797_t01 0

grmzm2g164814_t01 -0.82

grmzm2g164821_t01 0

grmzm2g164835_t01 0

grmzm2g164854_t01 0

grmzm2g164862_t01 1.2

grmzm2g164868_t02 0

grmzm2g164878_t01 0

grmzm2g164912_t01 0

grmzm2g164945_t01 0

grmzm2g164948_t02 0

grmzm2g164956_t02 0

grmzm2g164963_t02 0

grmzm2g164965_t01 0

grmzm2g164974_t02 0

grmzm2g165007_t01 0

grmzm2g165042_t01 0

grmzm2g165044_t02 0

grmzm2g165060_t01 0

grmzm2g165063_t01 0

grmzm2g165137_t01 0

grmzm2g165176_t01 0

grmzm2g165177_t01 0

grmzm2g165179_t02 0.9

grmzm2g165192_t01 0

grmzm2g165195_t03 0

grmzm2g165208_t02 0

grmzm2g165209_t01 0

grmzm2g165231_t01 0

grmzm2g165255_t02 0

grmzm2g165272_t01 0

grmzm2g165290_t01 0

grmzm2g165325_t02 0

grmzm2g165351_t03 0

grmzm2g165355_t01 0

grmzm2g165357_t01 0

grmzm2g165383_t02 0

grmzm2g165390_t02 0

grmzm2g165418_t04 0

grmzm2g165422_t01 0

grmzm2g165426_t01 0

grmzm2g165428_t01 0

grmzm2g165461_t02 0

grmzm2g165483_t01 0

grmzm2g165506_t03 0

grmzm2g165511_t02 0

grmzm2g165521_t01 0

grmzm2g165530_t03 0

grmzm2g165535_t01 0

grmzm2g165567_t03 -1.67

grmzm2g165581_t02 0

grmzm2g165597_t01 0.98

grmzm2g165619_t02 0

grmzm2g165631_t01 0

grmzm2g165633_t01 0

grmzm2g165692_t01 0

grmzm2g165694_t01 0

grmzm2g165757_t02 0

grmzm2g165769_t01 0

grmzm2g165778_t01 0

grmzm2g165815_t03 0

grmzm2g165901_t03 0

grmzm2g165917_t01 0

grmzm2g165919_t01 0

grmzm2g165924_t01 0

grmzm2g165926_t02 0

grmzm2g165931_t01 0

grmzm2g165939_t01 0

grmzm2g165944_t01 0

grmzm2g165945_t02 0

grmzm2g165949_t01 0

grmzm2g165961_t01 0

grmzm2g165963_t02 0

grmzm2g165966_t01 0

grmzm2g165969_t04 0

grmzm2g165972_t01 0

grmzm2g165987_t03 -0.9

grmzm2g165998_t01 0

grmzm2g166032_t01 0

grmzm2g166035_t02 0

grmzm2g166064_t01 0

grmzm2g166089_t06 0

grmzm2g166109_t04 0

grmzm2g166145_t01 0

grmzm2g166159_t01 0

grmzm2g166162_t01 0

grmzm2g166166_t01 0

grmzm2g166176_t01 -0.85

grmzm2g166218_t02 1.11

grmzm2g166230_t01 0

grmzm2g166245_t01 0

grmzm2g166278_t02 0

grmzm2g166281_t02 0

grmzm2g166297_t01 -2.1

grmzm2g166299_t01 0

grmzm2g166345_t04 0

grmzm2g166355_t03 0

grmzm2g166383_t02 0

grmzm2g166407_t01 0

grmzm2g166424_t05 0

grmzm2g166441_t01 0

grmzm2g166459_t01 0

grmzm2g166507_t01 0

grmzm2g166524_t02 0

grmzm2g166537_t01 0

grmzm2g166572_t01 0

grmzm2g166597_t01 0

grmzm2g166631_t03 0

grmzm2g166639_t01 0

grmzm2g166646_t01 0

grmzm2g166653_t01 0

grmzm2g166658_t01 0

grmzm2g166659_t02 0

grmzm2g166665_t02 0

grmzm2g166671_t01 0

grmzm2g166692_t02 0

grmzm2g166694_t04 0

grmzm2g166695_t01 0

grmzm2g166701_t01 0

grmzm2g166713_t02 0

grmzm2g166719_t01 0

grmzm2g166721_t01 0

grmzm2g166738_t02 0

grmzm2g166759_t01 0

grmzm2g166767_t02 0.82

grmzm2g166771_t02 0

grmzm2g166780_t03 0

grmzm2g166811_t01 0

grmzm2g166855_t01 0

grmzm2g166873_t01 0

grmzm2g166875_t01 0

grmzm2g166877_t01 0

grmzm2g166897_t01 0

grmzm2g166899_t01 0

grmzm2g166903_t02 0

grmzm2g166931_t01 0.84

grmzm2g166940_t01 0

grmzm2g166944_t02 0

grmzm2g166946_t01 0

grmzm2g166963_t01 0

grmzm2g166976_t01 0

grmzm2g166985_t01 0

grmzm2g166987_t02 0

grmzm2g166994_t01 0.96

grmzm2g167018_t01 0

grmzm2g167031_t04 0

grmzm2g167085_t02 0

grmzm2g167138_t01 0

grmzm2g167156_t01 0

grmzm2g167164_t01 -0.81

grmzm2g167165_t01 0

grmzm2g167207_t01 0

grmzm2g167220_t01 -1.11

grmzm2g167243_t01 0

grmzm2g167245_t01 0

grmzm2g167247_t01 0

grmzm2g167253_t01 0

grmzm2g167262_t01 0

grmzm2g167280_t01 0

grmzm2g167283_t02 0

grmzm2g167290_t01 0

grmzm2g167336_t01 0

grmzm2g167356_t01 0

grmzm2g167387_t02 0

grmzm2g167431_t02 0

grmzm2g167438_t02 -1.31

grmzm2g167463_t01 0

grmzm2g167492_t01 0

grmzm2g167497_t02 0

grmzm2g167505_t01 0

grmzm2g167548_t01 0

grmzm2g167549_t01 -0.85

grmzm2g167584_t02 0

grmzm2g167594_t02 0

grmzm2g167613_t02 0

grmzm2g167637_t02 0

grmzm2g167649_t01 0

grmzm2g167651_t01 0

grmzm2g167669_t01 0

grmzm2g167694_t02 0

grmzm2g167718_t01 0

grmzm2g167728_t04 0

grmzm2g167741_t01 0

grmzm2g167758_t02 0

grmzm2g167766_t01 -0.89

grmzm2g167767_t01 -1.05

grmzm2g167786_t01 0

grmzm2g167787_t01 0

grmzm2g167794_t01 0

grmzm2g167809_t01 0

grmzm2g167836_t01 0

grmzm2g167854_t01 0

grmzm2g167856_t01 0

grmzm2g167868_t01 0

grmzm2g167872_t01 0

grmzm2g167875_t02 1.71

grmzm2g167892_t03 0

grmzm2g167932_t01 0

grmzm2g167957_t02 0

grmzm2g167966_t01 0

grmzm2g167999_t01 0

grmzm2g168002_t03 -0.83

grmzm2g168077_t02 0

grmzm2g168087_t01 0

grmzm2g168096_t01 0

grmzm2g168115_t01 0

grmzm2g168119_t01 -1.02

grmzm2g168143_t01 0

grmzm2g168149_t01 0

grmzm2g168163_t01 0

grmzm2g168166_t02 0

grmzm2g168176_t02 0

grmzm2g168190_t02 0

grmzm2g168200_t01 0

grmzm2g168205_t01 0

grmzm2g168214_t02 0

grmzm2g168222_t01 0

grmzm2g168240_t01 1.14

grmzm2g168249_t01 0

grmzm2g168252_t01 0

grmzm2g168253_t01 0

grmzm2g168257_t04 0

grmzm2g168269_t04 0

grmzm2g168281_t01 0

grmzm2g168299_t01 0

grmzm2g168301_t02 0

grmzm2g168304_t01 -1.06

grmzm2g168330_t03 0

grmzm2g168335_t01 0

grmzm2g168337_t01 0

grmzm2g168364_t02 -0.81

grmzm2g168375_t01 0

grmzm2g168378_t01 0

grmzm2g168384_t01 1.01

grmzm2g168393_t01 0

grmzm2g168412_t01 0

grmzm2g168416_t01 0

grmzm2g168428_t01 0

grmzm2g168439_t01 -1.05

grmzm2g168449_t02 0

grmzm2g168474_t01 0

grmzm2g168476_t01 0

grmzm2g168506_t02 0

grmzm2g168510_t01 0

grmzm2g168552_t01 0

grmzm2g168590_t01 0

grmzm2g168629_t02 0

grmzm2g168651_t01 0

grmzm2g168665_t01 0

grmzm2g168674_t01 0

grmzm2g168679_t01 0

grmzm2g168685_t01 0

grmzm2g168690_t01 0

grmzm2g168693_t01 0

grmzm2g168706_t04 0

grmzm2g168707_t01 0

grmzm2g168744_t02 0

grmzm2g168760_t01 0

grmzm2g168762_t01 0

grmzm2g168791_t02 0

grmzm2g168807_t01 0

grmzm2g168817_t02 0

grmzm2g168829_t01 0

grmzm2g168833_t01 -0.81

grmzm2g168855_t01 0

grmzm2g168858_t01 0

grmzm2g168886_t03 0

grmzm2g168888_t10 0

grmzm2g168890_t01 0

grmzm2g168898_t01 0

grmzm2g168909_t03 0

grmzm2g168913_t02 0

grmzm2g168917_t01 0

grmzm2g168920_t02 0

grmzm2g168953_t03 0

grmzm2g168976_t01 0

grmzm2g169000_t01 0

grmzm2g169005_t01 0

grmzm2g169020_t02 1.25

grmzm2g169044_t01 0

grmzm2g169064_t01 0

grmzm2g169087_t01 0

grmzm2g169095_t02 0

grmzm2g169114_t02 0.94

grmzm2g169116_t01 0

grmzm2g169152_t01 0

grmzm2g169160_t01 0

grmzm2g169167_t01 0

grmzm2g169182_t01 0

grmzm2g169184_t01 0

grmzm2g169198_t01 0

grmzm2g169201_t02 0

grmzm2g169207_t01 0

grmzm2g169220_t01 0

grmzm2g169270_t02 0

grmzm2g169280_t01 0

grmzm2g169293_t01 0

grmzm2g169316_t02 0

grmzm2g169320_t02 0

grmzm2g169321_t04 0

grmzm2g169326_t01 0

grmzm2g169329_t01 0

grmzm2g169333_t02 0

grmzm2g169363_t01 0

grmzm2g169365_t01 0

grmzm2g169372_t01 0

grmzm2g169382_t01 0

grmzm2g169398_t01 0

grmzm2g169402_t01 0

grmzm2g169405_t01 0

grmzm2g169412_t02 0

grmzm2g169432_t04 0

grmzm2g169449_t01 0

grmzm2g169451_t01 0

grmzm2g169458_t01 1.63

grmzm2g169462_t01 0

grmzm2g169470_t02 0

grmzm2g169481_t03 0

grmzm2g169486_t03 0

grmzm2g169516_t04 0

grmzm2g169539_t03 0

grmzm2g169558_t01 0

grmzm2g169580_t01 0

grmzm2g169593_t03 0

grmzm2g169615_t02 0

grmzm2g169648_t01 0

grmzm2g169654_t01 0

grmzm2g169671_t01 0

grmzm2g169681_t01 0

grmzm2g169688_t01 0

grmzm2g169699_t02 0

grmzm2g169747_t02 0

grmzm2g169773_t01 0

grmzm2g169788_t01 0

grmzm2g169820_t01 0

grmzm2g169871_t01 0

grmzm2g169890_t03 0

grmzm2g169911_t01 0

grmzm2g169927_t03 0

grmzm2g169929_t01 0

grmzm2g169931_t02 0

grmzm2g169994_t01 0

grmzm2g169998_t01 0

grmzm2g170008_t01 0

grmzm2g170013_t01 0

grmzm2g170016_t01 0

grmzm2g170035_t01 0

grmzm2g170044_t01 1.95

grmzm2g170101_t01 0

grmzm2g170120_t04 0

grmzm2g170128_t01 0

grmzm2g170137_t01 0

grmzm2g170148_t01 0.84

grmzm2g170161_t01 0

grmzm2g170253_t01 0

grmzm2g170276_t01 0

grmzm2g170281_t01 0

grmzm2g170291_t02 0

grmzm2g170299_t01 0

grmzm2g170307_t01 0

grmzm2g170313_t01 0.83

grmzm2g170326_t01 0

grmzm2g170336_t01 0.84

grmzm2g170365_t04 1.33

grmzm2g170382_t05 0

grmzm2g170388_t01 0

grmzm2g170397_t01 0

grmzm2g170400_t01 0

grmzm2g170412_t01 0

grmzm2g170413_t01 0

grmzm2g170434_t02 0

grmzm2g170457_t02 0

grmzm2g170489_t01 0

grmzm2g170501_t01 0

grmzm2g170516_t01 0

grmzm2g170522_t01 0

grmzm2g170532_t02 0

grmzm2g170567_t01 0

grmzm2g170591_t02 0

grmzm2g170595_t01 0

grmzm2g170602_t01 0

grmzm2g170612_t02 0

grmzm2g170632_t01 0

grmzm2g170646_t01 0

grmzm2g170692_t01 0

grmzm2g170699_t01 0

grmzm2g170727_t06 0

grmzm2g170760_t02 0

grmzm2g170798_t02 0

grmzm2g170805_t03 0

grmzm2g170812_t01 0

grmzm2g170842_t02 0

grmzm2g170843_t03 0

grmzm2g170870_t01 0

grmzm2g170890_t03 0

grmzm2g170896_t01 0

grmzm2g170927_t01 0

grmzm2g170934_t01 0

grmzm2g170941_t01 0

grmzm2g170982_t01 0

grmzm2g171006_t02 0

grmzm2g171022_t01 0

grmzm2g171028_t01 0

grmzm2g171031_t01 0

grmzm2g171060_t01 0

grmzm2g171068_t01 0

grmzm2g171080_t01 0

grmzm2g171111_t04 0

grmzm2g171116_t01 -0.98

grmzm2g171118_t01 0

grmzm2g171122_t01 0

grmzm2g171139_t01 0

grmzm2g171159_t01 0

grmzm2g171179_t01 0

grmzm2g171181_t01 0

grmzm2g171187_t01 0

grmzm2g171232_t01 0

grmzm2g171236_t02 0

grmzm2g171254_t01 0

grmzm2g171277_t02 0

grmzm2g171317_t01 0

grmzm2g171324_t03 0

grmzm2g171333_t01 0

grmzm2g171354_t03 0

grmzm2g171367_t02 0

grmzm2g171370_t05 0

grmzm2g171372_t01 0

grmzm2g171373_t01 0

grmzm2g171383_t01 0

grmzm2g171387_t02 0

grmzm2g171390_t03 -1.37

grmzm2g171394_t01 0

grmzm2g171395_t01 0

grmzm2g171400_t01 0

grmzm2g171401_t02 0

grmzm2g171406_t06 0

grmzm2g171408_t03 0

grmzm2g171410_t05 0

grmzm2g171420_t01 0

grmzm2g171423_t02 -1.16

grmzm2g171426_t01 0

grmzm2g171428_t01 0

grmzm2g171430_t01 0

grmzm2g171435_t02 0

grmzm2g171440_t01 0

grmzm2g171444_t01 0

grmzm2g171452_t01 0.99

grmzm2g171466_t01 0

grmzm2g171468_t03 -1.71

grmzm2g171469_t01 0

grmzm2g171484_t01 0

grmzm2g171501_t01 0

grmzm2g171507_t03 0

grmzm2g171518_t02 0

grmzm2g171520_t01 0

grmzm2g171556_t05 0

grmzm2g171565_t01 0

grmzm2g171569_t01 0

grmzm2g171588_t01 0

grmzm2g171595_t01 0

grmzm2g171600_t01 0

grmzm2g171604_t01 0

grmzm2g171616_t01 -1.07

grmzm2g171620_t02 0

grmzm2g171622_t02 0

grmzm2g171628_t02 0

grmzm2g171660_t01 0

grmzm2g171662_t04 0

grmzm2g171664_t01 0

grmzm2g171677_t01 1.14

grmzm2g171688_t01 0

grmzm2g171716_t01 0

grmzm2g171723_t02 0

grmzm2g171745_t02 0

grmzm2g171781_t01 0

grmzm2g171807_t01 0

grmzm2g171818_t03 0

grmzm2g171847_t01 0

grmzm2g171852_t01 0

grmzm2g171871_t01 0

grmzm2g171880_t01 0

grmzm2g171884_t02 0

grmzm2g171887_t01 0

grmzm2g171921_t04 0

grmzm2g171934_t01 0

grmzm2g171961_t01 0

grmzm2g171967_t01 0

grmzm2g171987_t01 0

grmzm2g171994_t01 0

grmzm2g171998_t01 0

grmzm2g172001_t03 0

grmzm2g172011_t01 0

grmzm2g172014_t01 0

grmzm2g172021_t01 0

grmzm2g172032_t01 0

grmzm2g172043_t02 -1.42

grmzm2g172098_t01 0

grmzm2g172101_t01 0

grmzm2g172132_t04 0

grmzm2g172138_t01 0

grmzm2g172152_t04 0

grmzm2g172183_t01 0

grmzm2g172204_t01 0

grmzm2g172210_t01 0

grmzm2g172214_t05 -1.19

grmzm2g172230_t01 0

grmzm2g172239_t02 0

grmzm2g172244_t04 0

grmzm2g172258_t01 0

grmzm2g172274_t01 0

grmzm2g172322_t02 0

grmzm2g172330_t01 0.91

grmzm2g172342_t01 0

grmzm2g172357_t06 0

grmzm2g172369_t03 0

grmzm2g172376_t01 0

grmzm2g172396_t02 0

grmzm2g172399_t01 0

grmzm2g172410_t01 0

grmzm2g172413_t01 0

grmzm2g172420_t02 0

grmzm2g172427_t01 0

grmzm2g172442_t04 0

grmzm2g172451_t01 0

grmzm2g172485_t01 0

grmzm2g172491_t01 -1.07

grmzm2g172506_t01 0

grmzm2g172523_t03 0

grmzm2g172529_t01 0

grmzm2g172581_t01 0

grmzm2g172605_t01 0

grmzm2g172621_t01 0

grmzm2g172642_t01 0

grmzm2g172647_t01 0

grmzm2g172652_t01 0

grmzm2g172695_t01 0

grmzm2g172710_t03 0

grmzm2g172723_t01 0

grmzm2g172726_t01 0

grmzm2g172826_t02 0

grmzm2g172829_t01 0

grmzm2g172834_t01 0

grmzm2g172883_t02 0

grmzm2g172900_t01 0

grmzm2g172930_t03 0

grmzm2g172932_t01 0

grmzm2g172956_t02 0

grmzm2g172997_t01 0

grmzm2g173030_t01 0

grmzm2g173035_t01 0

grmzm2g173040_t01 0

grmzm2g173067_t01 0

grmzm2g173085_t01 0

grmzm2g173119_t01 0

grmzm2g173162_t01 0

grmzm2g173186_t02 0

grmzm2g173195_t01 0

grmzm2g173209_t01 0

grmzm2g173251_t01 0

grmzm2g173272_t01 0

grmzm2g173277_t01 0

grmzm2g173309_t01 0

grmzm2g173315_t01 0

grmzm2g173317_t03 0

grmzm2g173341_t02 0

grmzm2g173354_t02 0

grmzm2g173360_t01 0

grmzm2g173387_t02 0

grmzm2g173404_t01 0

grmzm2g173413_t01 0

grmzm2g173425_t03 0

grmzm2g173428_t01 0

grmzm2g173429_t01 0

grmzm2g173472_t01 0

grmzm2g173534_t01 0

grmzm2g173536_t04 0

grmzm2g173542_t04 0

grmzm2g173563_t01 0

grmzm2g173579_t01 0

grmzm2g173597_t03 0

grmzm2g173612_t01 0

grmzm2g173615_t01 0

grmzm2g173628_t01 0

grmzm2g173630_t01 0

grmzm2g173636_t02 0

grmzm2g173641_t01 0

grmzm2g173647_t01 0

grmzm2g173649_t03 0

grmzm2g173666_t01 0

grmzm2g173669_t01 0

grmzm2g173680_t02 0

grmzm2g173682_t02 0

grmzm2g173684_t01 0

grmzm2g173693_t01 0

grmzm2g173710_t01 0

grmzm2g173718_t01 0

grmzm2g173734_t01 0

grmzm2g173739_t01 0

grmzm2g173747_t01 0

grmzm2g173756_t01 0

grmzm2g173759_t01 0

grmzm2g173763_t01 0

grmzm2g173777_t03 0

grmzm2g173792_t02 1.06

grmzm2g173852_t01 0

grmzm2g173858_t01 0

grmzm2g173863_t01 0

grmzm2g173868_t01 0

grmzm2g173874_t01 0

grmzm2g173878_t01 0

grmzm2g173910_t01 0

grmzm2g173928_t01 0

grmzm2g173943_t02 0

grmzm2g173950_t01 0

grmzm2g173962_t01 0

grmzm2g173967_t03 0

grmzm2g173989_t03 0

grmzm2g174005_t01 0

grmzm2g174048_t01 0

grmzm2g174070_t01 0

grmzm2g174099_t01 0

grmzm2g174107_t02 0

grmzm2g174136_t01 0

grmzm2g174145_t05 0

grmzm2g174149_t02 0

grmzm2g174161_t01 0

grmzm2g174170_t01 0

grmzm2g174196_t02 0

grmzm2g174204_t01 0

grmzm2g174218_t01 0

grmzm2g174221_t01 0

grmzm2g174236_t01 0

grmzm2g174240_t01 0

grmzm2g174249_t04 0

grmzm2g174284_t01 0

grmzm2g174286_t04 0

grmzm2g174310_t01 0

grmzm2g174315_t03 0

grmzm2g174335_t01 0

grmzm2g174347_t01 0

grmzm2g174396_t01 0

grmzm2g174427_t01 0

grmzm2g174440_t01 0

grmzm2g174444_t03 0

grmzm2g174449_t01 0

grmzm2g174479_t01 0

grmzm2g174481_t01 0

grmzm2g174511_t01 0

grmzm2g174549_t01 0

grmzm2g174554_t01 0

grmzm2g174558_t01 0

grmzm2g174562_t01 0

grmzm2g174568_t01 0

grmzm2g174570_t03 0

grmzm2g174572_t01 0

grmzm2g174596_t02 0

grmzm2g174598_t02 0

grmzm2g174610_t03 0

grmzm2g174615_t01 0

grmzm2g174619_t02 0

grmzm2g174644_t03 0

grmzm2g174667_t01 0

grmzm2g174669_t04 0

grmzm2g174671_t02 0

grmzm2g174673_t01 0

grmzm2g174675_t02 0

grmzm2g174680_t02 0

grmzm2g174684_t01 0

grmzm2g174696_t02 0

grmzm2g174708_t01 0

grmzm2g174716_t04 0

grmzm2g174719_t02 1.45

grmzm2g174730_t02 0

grmzm2g174736_t02 1.17

grmzm2g174738_t01 0

grmzm2g174741_t01 0

grmzm2g174757_t01 0

grmzm2g174773_t01 0

grmzm2g174776_t01 0

grmzm2g174784_t02 0

grmzm2g174785_t02 0

grmzm2g174797_t02 0

grmzm2g174799_t01 0

grmzm2g174803_t01 0

grmzm2g174807_t02 0

grmzm2g174819_t01 0

grmzm2g174830_t01 0

grmzm2g174855_t02 -1.14

grmzm2g174896_t03 0

grmzm2g174900_t01 0

grmzm2g174906_t01 0

grmzm2g174917_t01 -0.89

grmzm2g174919_t03 0

grmzm2g174926_t02 0

grmzm2g174938_t01 0

grmzm2g174942_t04 0

grmzm2g174949_t01 0.84

grmzm2g174975_t02 0

grmzm2g174984_t01 0

grmzm2g174990_t02 0

grmzm2g175000_t01 0

grmzm2g175008_t01 0

grmzm2g175019_t02 0

grmzm2g175031_t01 0

grmzm2g175038_t01 0

grmzm2g175065_t03 0

grmzm2g175071_t01 0

grmzm2g175076_t01 0

grmzm2g175089_t03 0

grmzm2g175134_t02 0

grmzm2g175140_t01 0

grmzm2g175141_t05 0

grmzm2g175171_t01 0

grmzm2g175218_t02 0

grmzm2g175232_t01 0

grmzm2g175236_t01 0

grmzm2g175269_t01 0

grmzm2g175272_t03 0

grmzm2g175280_t02 0

grmzm2g175362_t01 0

grmzm2g175396_t01 0

grmzm2g175406_t01 0

grmzm2g175419_t01 0

grmzm2g175423_t02 0

grmzm2g175447_t01 0

grmzm2g175453_t02 0

grmzm2g175463_t01 0

grmzm2g175499_t01 0

grmzm2g175504_t01 0

grmzm2g175510_t02 0

grmzm2g175543_t01 0

grmzm2g175552_t02 0

grmzm2g175562_t02 0

grmzm2g175563_t01 0

grmzm2g175576_t02 0

grmzm2g175593_t01 0.89

grmzm2g175610_t01 0.9

grmzm2g175642_t01 0

grmzm2g175661_t01 0

grmzm2g175676_t03 0

grmzm2g175685_t01 0

grmzm2g175718_t01 0

grmzm2g175728_t01 0

grmzm2g175761_t01 0

grmzm2g175782_t02 0

grmzm2g175797_t01 0

grmzm2g175799_t01 0

grmzm2g175816_t05 0

grmzm2g175818_t01 0

grmzm2g175867_t02 0

grmzm2g175870_t03 0

grmzm2g175989_t01 0

grmzm2g176028_t01 0

grmzm2g176029_t01 0

grmzm2g176056_t01 0

grmzm2g176129_t01 0

grmzm2g176175_t01 0

grmzm2g176206_t01 0

grmzm2g176209_t01 0

grmzm2g176225_t01 0.84

grmzm2g176235_t02 0

grmzm2g176253_t01 0

grmzm2g176294_t01 0

grmzm2g176307_t01 0

grmzm2g176340_t02 0

grmzm2g176347_t01 0

grmzm2g176358_t02 0

grmzm2g176375_t01 0

grmzm2g176382_t01 0

grmzm2g176394_t01 -0.99

grmzm2g176396_t04 0

grmzm2g176397_t01 0

grmzm2g176407_t02 0

grmzm2g176419_t01 0

grmzm2g176437_t01 0

grmzm2g176443_t01 0

grmzm2g176446_t01 0

grmzm2g176455_t01 0

grmzm2g176465_t01 0

grmzm2g176486_t01 0

grmzm2g176499_t01 0

grmzm2g176506_t03 0

grmzm2g176519_t01 0

grmzm2g176521_t01 0

grmzm2g176523_t01 0

grmzm2g176542_t01 1.3

grmzm2g176546_t01 0

grmzm2g176558_t01 0

grmzm2g176559_t01 0

grmzm2g176576_t01 0

grmzm2g176595_t01 0

grmzm2g176612_t02 0

grmzm2g176647_t01 0

grmzm2g176651_t01 0

grmzm2g176662_t01 0

grmzm2g176677_t01 0

grmzm2g176688_t01 0

grmzm2g176692_t03 0

grmzm2g176698_t01 0

grmzm2g176699_t01 0

grmzm2g176707_t01 0

grmzm2g176718_t02 0

grmzm2g176735_t02 0

grmzm2g176737_t03 0

grmzm2g176748_t01 0

grmzm2g176780_t01 0

grmzm2g176820_t01 0

grmzm2g176837_t01 0

grmzm2g176840_t01 0

grmzm2g176881_t03 0

grmzm2g176903_t03 0

grmzm2g176910_t01 0

grmzm2g176918_t02 0

grmzm2g176977_t04 0

grmzm2g176995_t01 0

grmzm2g176998_t01 -1.97

grmzm2g177001_t02 0

grmzm2g177005_t01 0

grmzm2g177019_t01 0

grmzm2g177026_t03 0

grmzm2g177050_t01 0

grmzm2g177052_t01 0

grmzm2g177070_t01 0

grmzm2g177072_t01 1.16

grmzm2g177076_t01 0

grmzm2g177077_t01 0.89

grmzm2g177098_t03 0

grmzm2g177107_t04 0

grmzm2g177138_t01 0

grmzm2g177151_t02 0

grmzm2g177169_t01 0

grmzm2g177198_t01 0

grmzm2g177203_t01 0

grmzm2g177213_t02 0

grmzm2g177220_t01 0

grmzm2g177224_t01 0

grmzm2g177227_t02 0

grmzm2g177229_t01 0

grmzm2g177231_t05 0

grmzm2g177242_t01 0

grmzm2g177259_t01 0

grmzm2g177263_t02 0

grmzm2g177273_t01 0

grmzm2g177276_t01 0

grmzm2g177326_t01 0

grmzm2g177356_t01 0

grmzm2g177386_t01 0

grmzm2g177412_t05 0

grmzm2g177424_t01 0

grmzm2g177447_t01 0

grmzm2g177508_t01 0

grmzm2g177518_t01 0

grmzm2g177532_t01 0

grmzm2g177539_t01 0

grmzm2g177549_t04 1.37

grmzm2g177570_t01 1.44

grmzm2g177575_t01 0

grmzm2g177599_t01 0

grmzm2g177617_t06 0

grmzm2g177621_t01 0

grmzm2g177627_t03 0

grmzm2g177631_t01 0

grmzm2g177654_t01 0

grmzm2g177659_t04 0

grmzm2g177668_t01 0

grmzm2g177693_t03 0

grmzm2g177720_t01 0

grmzm2g177776_t01 1.61

grmzm2g177781_t03 0

grmzm2g177812_t01 0

grmzm2g177842_t01 0

grmzm2g177853_t01 0

grmzm2g177867_t02 0

grmzm2g177883_t01 0

grmzm2g177885_t03 0

grmzm2g177895_t01 0

grmzm2g177906_t01 0

grmzm2g177912_t02 0

grmzm2g177914_t02 0

grmzm2g177923_t01 0

grmzm2g177928_t02 0

grmzm2g177937_t02 0

grmzm2g177947_t02 0

grmzm2g177970_t03 0

grmzm2g177974_t02 0

grmzm2g177991_t01 0

grmzm2g178014_t02 0

grmzm2g178025_t01 -0.99

grmzm2g178072_t02 0

grmzm2g178079_t01 0

grmzm2g178102_t01 0

grmzm2g178106_t01 0

grmzm2g178120_t05 0

grmzm2g178169_t02 0

grmzm2g178190_t01 0

grmzm2g178192_t01 0

grmzm2g178199_t01 0

grmzm2g178227_t01 0

grmzm2g178244_t03 0

grmzm2g178254_t01 0

grmzm2g178261_t02 0

grmzm2g178278_t02 0

grmzm2g178294_t02 0

grmzm2g178319_t01 0

grmzm2g178333_t01 0

grmzm2g178341_t02 0

grmzm2g178371_t04 0

grmzm2g178398_t01 0

grmzm2g178415_t04 0

grmzm2g178455_t01 0

grmzm2g178460_t01 0

grmzm2g178486_t02 0

grmzm2g178497_t01 0

grmzm2g178517_t05 0

grmzm2g178522_t03 0.87

grmzm2g178533_t02 0

grmzm2g178546_t02 0

grmzm2g178571_t01 0

grmzm2g178576_t06 0

grmzm2g178595_t02 0

grmzm2g178602_t01 0

grmzm2g178616_t01 0

grmzm2g178618_t01 0

grmzm2g178628_t02 0

grmzm2g178645_t01 0

grmzm2g178650_t03 0

grmzm2g178663_t01 0

grmzm2g178681_t01 0

grmzm2g178704_t01 0

grmzm2g178747_t01 0.99

grmzm2g178756_t01 0

grmzm2g178758_t01 0.83

grmzm2g178769_t01 0

grmzm2g178775_t01 0

grmzm2g178787_t03 0

grmzm2g178797_t02 0

grmzm2g178801_t03 0

grmzm2g178807_t02 0

grmzm2g178822_t04 0

grmzm2g178826_t01 0

grmzm2g178829_t03 0

grmzm2g178839_t01 0

grmzm2g178847_t01 0

grmzm2g178852_t01 0

grmzm2g178859_t03 0

grmzm2g178875_t02 0

grmzm2g178880_t02 0

grmzm2g178886_t01 0

grmzm2g178892_t01 0

grmzm2g178910_t02 1.31

grmzm2g178916_t01 0

grmzm2g178917_t01 0

grmzm2g178919_t01 0

grmzm2g178924_t03 0

grmzm2g178933_t02 0

grmzm2g178945_t01 0

grmzm2g178955_t01 0

grmzm2g178958_t01 0

grmzm2g178960_t03 0

grmzm2g178968_t02 0

grmzm2g178972_t01 0

grmzm2g178996_t01 0

grmzm2g178998_t01 0

grmzm2g179002_t02 0

grmzm2g179005_t03 0

grmzm2g179024_t01 -1.41

grmzm2g179031_t02 0

grmzm2g179069_t01 -1.29

grmzm2g179090_t01 0

grmzm2g179097_t07 0

grmzm2g179106_t02 0

grmzm2g179120_t01 0

grmzm2g179133_t02 0

grmzm2g179146_t01 0

grmzm2g179147_t01 -1.69

grmzm2g179155_t01 0

grmzm2g179167_t01 0

grmzm2g179215_t01 0

grmzm2g179217_t01 0

grmzm2g179220_t01 0

grmzm2g179253_t01 0

grmzm2g179279_t01 0

grmzm2g179281_t03 0

grmzm2g179292_t01 0

grmzm2g179301_t01 0

grmzm2g179308_t04 0

grmzm2g179313_t01 0

grmzm2g179325_t01 0

grmzm2g179329_t01 0

grmzm2g179336_t01 0

grmzm2g179346_t01 0

grmzm2g179351_t01 0

grmzm2g179366_t02 0

grmzm2g179411_t01 0

grmzm2g179421_t02 0

grmzm2g179429_t01 0

grmzm2g179454_t01 0

grmzm2g179459_t01 0

grmzm2g179465_t01 0

grmzm2g179505_t05 0

grmzm2g179507_t01 0

grmzm2g179518_t01 0

grmzm2g179523_t01 0

grmzm2g179532_t01 0

grmzm2g179551_t01 0

grmzm2g179633_t01 0

grmzm2g179638_t01 0

grmzm2g179658_t01 0

grmzm2g179662_t02 0

grmzm2g179677_t02 0

grmzm2g179688_t01 0

grmzm2g179689_t01 0

grmzm2g179693_t01 0

grmzm2g179703_t02 -1.29

grmzm2g179709_t01 0

grmzm2g179715_t04 0

grmzm2g179732_t03 0

grmzm2g179737_t03 0

grmzm2g179792_t09 -1.52

grmzm2g179793_t02 0

grmzm2g179797_t01 0

grmzm2g179800_t01 0

grmzm2g179802_t01 0

grmzm2g179827_t01 0

grmzm2g179838_t01 0

grmzm2g179906_t01 0

grmzm2g179907_t02 0

grmzm2g179941_t01 0

grmzm2g179976_t01 0

grmzm2g179981_t01 0

grmzm2g179985_t01 0

grmzm2g180021_t01 0

grmzm2g180033_t01 0

grmzm2g180065_t01 0

grmzm2g180082_t01 0

grmzm2g180086_t01 0

grmzm2g180166_t01 0

grmzm2g180172_t03 0

grmzm2g180205_t02 0

grmzm2g180211_t01 0

grmzm2g180244_t01 0

grmzm2g180251_t02 0

grmzm2g180283_t02 0

grmzm2g180335_t02 0

grmzm2g180343_t02 0

grmzm2g180372_t02 0

grmzm2g180384_t01 0

grmzm2g180399_t01 0

grmzm2g180406_t01 0

grmzm2g180418_t01 0

grmzm2g180422_t01 0

grmzm2g180430_t02 0

grmzm2g180458_t01 0

grmzm2g180471_t03 0.96

grmzm2g180509_t01 0

grmzm2g180555_t01 0

grmzm2g180558_t01 0

grmzm2g180575_t01 0

grmzm2g180578_t01 0

grmzm2g180596_t01 0

grmzm2g180605_t01 0

grmzm2g180612_t01 0

grmzm2g180620_t01 0

grmzm2g180622_t01 0

grmzm2g180625_t02 0

grmzm2g180639_t05 0

grmzm2g180654_t03 0

grmzm2g180659_t02 0

grmzm2g180668_t01 0

grmzm2g180691_t02 0

grmzm2g180699_t01 0

grmzm2g180704_t01 0

grmzm2g180716_t03 0

grmzm2g180720_t04 0

grmzm2g180724_t01 0

grmzm2g180728_t02 0

grmzm2g180732_t02 0

grmzm2g180811_t01 0

grmzm2g180847_t01 0

grmzm2g180863_t01 0

grmzm2g180870_t04 -1.06

grmzm2g180889_t01 0

grmzm2g180909_t01 0

grmzm2g180916_t07 0

grmzm2g180920_t01 0

grmzm2g180922_t01 0

grmzm2g180930_t01 0

grmzm2g180951_t01 0

grmzm2g180967_t01 0

grmzm2g180971_t02 0

grmzm2g180979_t01 0

grmzm2g180982_t01 0

grmzm2g180983_t01 0

grmzm2g180988_t01 0

grmzm2g180990_t01 0

grmzm2g181002_t01 0

grmzm2g181018_t01 0

grmzm2g181021_t02 0

grmzm2g181028_t01 0

grmzm2g181030_t01 0

grmzm2g181081_t02 0

grmzm2g181104_t02 0

grmzm2g181148_t04 0

grmzm2g181151_t01 0

grmzm2g181153_t01 0

grmzm2g181192_t03 0

grmzm2g181194_t03 0

grmzm2g181219_t02 0

grmzm2g181227_t01 0

grmzm2g181231_t02 0

grmzm2g181236_t01 0

grmzm2g181251_t01 0

grmzm2g181258_t01 0

grmzm2g181259_t01 0

grmzm2g181266_t03 0

grmzm2g181273_t01 0

grmzm2g181336_t05 0

grmzm2g181354_t02 0

grmzm2g181359_t01 0

grmzm2g181362_t05 0

grmzm2g181371_t02 0

grmzm2g181376_t01 0

grmzm2g181378_t01 0

grmzm2g181390_t02 0

grmzm2g181422_t02 0

grmzm2g181453_t05 0

grmzm2g181484_t02 0

grmzm2g181519_t03 0

grmzm2g181522_t01 0

grmzm2g181542_t02 0

grmzm2g181551_t01 0

grmzm2g181568_t01 0

grmzm2g181605_t01 -1.7

grmzm2g181607_t03 0

grmzm2g181637_t01 0

grmzm2g300066_t01 0

grmzm2g300133_t01 0

grmzm2g300135_t01 0

grmzm2g300139_t01 0

grmzm2g300242_t01 0

grmzm2g300258_t01 0

grmzm2g300375_t02 0

grmzm2g300500_t01 0

grmzm2g300589_t01 0

grmzm2g300692_t01 0

grmzm2g300801_t01 0

grmzm2g300841_t02 0

grmzm2g300862_t07 0

grmzm2g300866_t01 0

grmzm2g300955_t01 0

grmzm2g301071_t01 0

grmzm2g301089_t02 0

grmzm2g301098_t02 0

grmzm2g301122_t01 0

grmzm2g301150_t01 0

grmzm2g301208_t01 0

grmzm2g301246_t01 0

grmzm2g301271_t01 0

grmzm2g301325_t01 0

grmzm2g301355_t01 0

grmzm2g301389_t01 0

grmzm2g301405_t01 0

grmzm2g301512_t01 0

grmzm2g301536_t02 0

grmzm2g301642_t01 0

grmzm2g301663_t01 0

grmzm2g301738_t01 0

grmzm2g301803_t03 0

grmzm2g301805_t02 0

grmzm2g301823_t01 0

grmzm2g301884_t01 0

grmzm2g301885_t03 0

grmzm2g301904_t01 0

grmzm2g301908_t01 0

grmzm2g301932_t01 0

grmzm2g301934_t01 0

grmzm2g301939_t01 0

grmzm2g302045_t01 0

grmzm2g302074_t01 0

grmzm2g302195_t01 0

grmzm2g302233_t01 0

grmzm2g302259_t04 0

grmzm2g302405_t02 0

grmzm2g302601_t01 0

grmzm2g302604_t01 0

grmzm2g302639_t01 0

grmzm2g302712_t01 0

grmzm2g302778_t01 0

grmzm2g302785_t01 0

grmzm2g302832_t01 0

grmzm2g302912_t01 0

grmzm2g302913_t01 0

grmzm2g303007_t01 0

grmzm2g303118_t02 0

grmzm2g303132_t01 0

grmzm2g303312_t04 0

grmzm2g303374_t04 0

grmzm2g303431_t01 0

grmzm2g303465_t04 0

grmzm2g303525_t01 0

grmzm2g303530_t01 0

grmzm2g303587_t01 0

grmzm2g303631_t04 0

grmzm2g303655_t01 0

grmzm2g303728_t01 0

grmzm2g303752_t02 0

grmzm2g303915_t01 0

grmzm2g303964_t01 0

grmzm2g303993_t01 0

grmzm2g304010_t01 0

grmzm2g304073_t01 0

grmzm2g304108_t01 0

grmzm2g304212_t01 0

grmzm2g304274_t01 0

grmzm2g304362_t01 0

grmzm2g304474_t01 0

grmzm2g304575_t02 -0.85

grmzm2g304638_t01 0

grmzm2g304712_t01 0

grmzm2g304915_t01 0

grmzm2g304952_t01 0

grmzm2g304953_t01 0

grmzm2g304965_t01 0

grmzm2g305007_t04 0

grmzm2g305027_t01 0

grmzm2g305046_t02 0

grmzm2g305060_t01 0

grmzm2g305115_t03 0

grmzm2g305154_t01 0

grmzm2g305247_t01 0

grmzm2g305254_t01 0

grmzm2g305264_t01 0

grmzm2g305280_t01 0

grmzm2g305569_t01 -0.98

grmzm2g305822_t01 1.28

grmzm2g305851_t01 0

grmzm2g305856_t02 0

grmzm2g305864_t02 0

grmzm2g305901_t01 0

grmzm2g306028_t02 0

grmzm2g306079_t01 0

grmzm2g306094_t02 0

grmzm2g306099_t05 0

grmzm2g306104_t01 0

grmzm2g306105_t01 0

grmzm2g306216_t01 0

grmzm2g306258_t01 0

grmzm2g306357_t01 0

grmzm2g306482_t01 0

grmzm2g306597_t01 0

grmzm2g306732_t02 0

grmzm2g306741_t01 0

grmzm2g306760_t01 0

grmzm2g306771_t01 0

grmzm2g306781_t01 0

grmzm2g306806_t01 0

grmzm2g306851_t01 0

grmzm2g306859_t01 0

grmzm2g306898_t01 0

grmzm2g306935_t02 0

grmzm2g307252_t01 0

grmzm2g307368_t01 0

grmzm2g307553_t01 0

grmzm2g307561_t02 0

grmzm2g307600_t01 0

grmzm2g307665_t01 0

grmzm2g307720_t01 1.33

grmzm2g307906_t01 0

grmzm2g307992_t01 0

grmzm2g308064_t01 1.31

grmzm2g308083_t01 0

grmzm2g308097_t01 0

grmzm2g308106_t01 0

grmzm2g308189_t01 0

grmzm2g308192_t01 0

grmzm2g308193_t04 0

grmzm2g308203_t02 0

grmzm2g308358_t01 0

grmzm2g308412_t01 0

grmzm2g308535_t01 0

grmzm2g308570_t01 0

grmzm2g308595_t01 0

grmzm2g308687_t01 0

grmzm2g308689_t01 0

grmzm2g308707_t01 0

grmzm2g308713_t01 0

grmzm2g308860_t01 0

grmzm2g308875_t01 0

grmzm2g308884_t01 0

grmzm2g308944_t01 0

grmzm2g308957_t01 0

grmzm2g309025_t01 0.9

grmzm2g309035_t01 0

grmzm2g309063_t02 0

grmzm2g309152_t02 0

grmzm2g309278_t01 0

grmzm2g309285_t01 0

grmzm2g309327_t01 0

grmzm2g309568_t01 0

grmzm2g309624_t01 0

grmzm2g309660_t02 0

grmzm2g309711_t01 0

grmzm2g309822_t01 0

grmzm2g309868_t01 0

grmzm2g309897_t01 0

grmzm2g309899_t01 0

grmzm2g309933_t01 0

grmzm2g309970_t01 0

grmzm2g310069_t01 0

grmzm2g310115_t04 0

grmzm2g310155_t02 0

grmzm2g310251_t01 0

grmzm2g310283_t01 0

grmzm2g310321_t01 0

grmzm2g310368_t01 -0.94

grmzm2g310431_t01 0

grmzm2g310453_t01 0

grmzm2g310569_t01 0

grmzm2g310607_t01 0

grmzm2g310880_t01 0

grmzm2g310947_t02 0.93

grmzm2g311003_t02 0

grmzm2g311036_t01 0

grmzm2g311051_t01 0

grmzm2g311165_t03 0

grmzm2g311220_t01 0

grmzm2g311232_t01 0

grmzm2g311316_t01 0

grmzm2g311367_t01 0

grmzm2g311665_t01 0

grmzm2g311680_t01 0

grmzm2g311744_t01 0

grmzm2g311865_t01 0

grmzm2g311898_t01 -1.99

grmzm2g311914_t01 0

grmzm2g311919_t02 0

grmzm2g311961_t01 0

grmzm2g312075_t01 0

grmzm2g312078_t01 -1.67

grmzm2g312091_t01 -1.68

grmzm2g312110_t01 0

grmzm2g312154_t01 0

grmzm2g312201_t04 0

grmzm2g312231_t01 0

grmzm2g312244_t01 0

grmzm2g312365_t01 0

grmzm2g312461_t01 0

grmzm2g312501_t01 0

grmzm2g312510_t01 0

grmzm2g312521_t01 -1.01

grmzm2g312661_t02 0

grmzm2g312693_t01 0

grmzm2g312712_t02 0

grmzm2g312738_t02 0

grmzm2g312806_t01 0

grmzm2g312817_t01 0

grmzm2g312838_t04 0

grmzm2g312839_t01 0

grmzm2g312877_t01 0

grmzm2g312910_t02 0

grmzm2g312944_t01 0

grmzm2g312954_t01 0

grmzm2g312970_t04 0

grmzm2g312997_t01 0

grmzm2g313009_t04 0

grmzm2g313020_t01 0

grmzm2g313027_t01 0

grmzm2g313101_t01 0

grmzm2g313162_t01 0

grmzm2g313316_t01 -0.86

grmzm2g313341_t01 0

grmzm2g313351_t01 0

grmzm2g313460_t01 0

grmzm2g313481_t02 0

grmzm2g313509_t01 0

grmzm2g313529_t01 0

grmzm2g313553_t02 0

grmzm2g313643_t01 0

grmzm2g313670_t01 0

grmzm2g313672_t01 0

grmzm2g313854_t01 0

grmzm2g313867_t01 0

grmzm2g313944_t01 0

grmzm2g314094_t01 0

grmzm2g314171_t01 0

grmzm2g314233_t02 0

grmzm2g314386_t02 0

grmzm2g314396_t01 0

grmzm2g314520_t01 0

grmzm2g314546_t01 0

grmzm2g314554_t06 0

grmzm2g314647_t01 0

grmzm2g314652_t01 0

grmzm2g314656_t01 0

grmzm2g314660_t02 0

grmzm2g314661_t01 0

grmzm2g314679_t08 0

grmzm2g314692_t03 0

grmzm2g314769_t02 0

grmzm2g314882_t03 0

grmzm2g314898_t01 0

grmzm2g314903_t01 0

grmzm2g314955_t02 0

grmzm2g315037_t01 0

grmzm2g315072_t05 0

grmzm2g315121_t01 0

grmzm2g315125_t01 0

grmzm2g315127_t01 0

grmzm2g315176_t02 0

grmzm2g315199_t01 0

grmzm2g315264_t01 0

grmzm2g315321_t01 -0.98

grmzm2g315375_t01 -1

grmzm2g315401_t02 0

grmzm2g315769_t01 0

grmzm2g315806_t01 0

grmzm2g315848_t01 0

grmzm2g315902_t01 0

grmzm2g315911_t01 0

grmzm2g315931_t03 0

grmzm2g316007_t01 0

grmzm2g316030_t01 0

grmzm2g316113_t05 0

grmzm2g316136_t02 0

grmzm2g316191_t02 0

grmzm2g316232_t01 0

grmzm2g316275_t01 1.63

grmzm2g316277_t01 0

grmzm2g316534_t01 0

grmzm2g316635_t03 0

grmzm2g316749_t01 0

grmzm2g316778_t01 0

grmzm2g316807_t01 0

grmzm2g316864_t02 0

grmzm2g316889_t02 0

grmzm2g316904_t01 0

grmzm2g316907_t01 0

grmzm2g316967_t01 0

grmzm2g317262_t01 0

grmzm2g317330_t01 0

grmzm2g317386_t01 0

grmzm2g317450_t03 0

grmzm2g317451_t01 0

grmzm2g317474_t01 0

grmzm2g317584_t01 0

grmzm2g317586_t01 0

grmzm2g317652_t01 0

grmzm2g317738_t02 0

grmzm2g317743_t01 0

grmzm2g317770_t01 0

grmzm2g317898_t01 0

grmzm2g317900_t02 1.24

grmzm2g317931_t01 0

grmzm2g317938_t01 0

grmzm2g318010_t01 0

grmzm2g318213_t01 0

grmzm2g318220_t01 0

grmzm2g318270_t01 0

grmzm2g318319_t01 0

grmzm2g318346_t03 0

grmzm2g318375_t01 0

grmzm2g318408_t01 0

grmzm2g318475_t02 0

grmzm2g318527_t01 0

grmzm2g318535_t01 0

grmzm2g318633_t02 0

grmzm2g318635_t01 0

grmzm2g318780_t01 0

grmzm2g318794_t01 0

grmzm2g318803_t01 0

grmzm2g318843_t01 0

grmzm2g318860_t01 0

grmzm2g318882_t01 0

grmzm2g318956_t01 0

grmzm2g319057_t01 0

grmzm2g319062_t01 0

grmzm2g319109_t01 0

grmzm2g319130_t02 0

grmzm2g319169_t02 0

grmzm2g319187_t01 0

grmzm2g319307_t01 0

grmzm2g319357_t02 0

grmzm2g319402_t01 0

grmzm2g319465_t03 0

grmzm2g319509_t01 0

grmzm2g319573_t04 0

grmzm2g319578_t01 0

grmzm2g319747_t01 0

grmzm2g319878_t01 0

grmzm2g319947_t01 0

grmzm2g320013_t01 0

grmzm2g320023_t01 0

grmzm2g320099_t01 0

grmzm2g320135_t02 0

grmzm2g320152_t01 0

grmzm2g320206_t02 0

grmzm2g320283_t01 0

grmzm2g320319_t01 0

grmzm2g320325_t01 0

grmzm2g320379_t02 0

grmzm2g320399_t01 0

grmzm2g320506_t01 0

grmzm2g320521_t01 0

grmzm2g320689_t05 0

grmzm2g320703_t01 0

grmzm2g320723_t01 0

grmzm2g320799_t01 0

grmzm2g320802_t03 0

grmzm2g320827_t01 0

grmzm2g320920_t01 0

grmzm2g321010_t01 -0.81

grmzm2g321041_t01 0

grmzm2g321053_t01 0

grmzm2g321239_t01 0

grmzm2g321290_t02 0

grmzm2g321404_t01 0

grmzm2g321606_t01 1.21

grmzm2g321725_t02 0

grmzm2g321753_t02 0

grmzm2g321767_t01 0

grmzm2g321778_t01 0

grmzm2g321839_t02 0

grmzm2g321940_t03 0

grmzm2g322001_t01 0

grmzm2g322024_t01 0

grmzm2g322047_t01 0

grmzm2g322129_t01 0

grmzm2g322186_t01 0

grmzm2g322287_t01 0

grmzm2g322299_t01 1.98

grmzm2g322314_t01 0

grmzm2g322328_t01 0

grmzm2g322348_t01 0

grmzm2g322413_t03 0

grmzm2g322493_t02 0

grmzm2g322506_t01 0

grmzm2g322593_t02 0

grmzm2g322615_t01 0

grmzm2g322618_t01 0

grmzm2g322634_t01 0

grmzm2g322641_t01 0

grmzm2g322661_t03 0

grmzm2g322723_t01 0

grmzm2g322728_t01 0

grmzm2g322817_t01 0

grmzm2g322846_t01 0

grmzm2g322953_t02 0

grmzm2g323013_t02 0

grmzm2g323024_t01 0

grmzm2g323060_t01 0

grmzm2g323261_t01 0

grmzm2g323309_t01 0

grmzm2g323353_t01 0

grmzm2g323387_t01 0

grmzm2g323422_t01 0

grmzm2g323470_t01 0

grmzm2g323473_t01 0

grmzm2g323504_t01 0

grmzm2g323622_t01 0

grmzm2g323719_t01 0

grmzm2g323754_t03 0

grmzm2g323830_t01 -0.87

grmzm2g323838_t02 0

grmzm2g323844_t01 0

grmzm2g323888_t01 0

grmzm2g323912_t01 0

grmzm2g324162_t01 0

grmzm2g324248_t01 0

grmzm2g324292_t01 0

grmzm2g324297_t01 0

grmzm2g324314_t03 0

grmzm2g324390_t01 0

grmzm2g324398_t01 0

grmzm2g324417_t01 0

grmzm2g324462_t01 0

grmzm2g324467_t01 0

grmzm2g324471_t06 0

grmzm2g324478_t01 0

grmzm2g324540_t01 0

grmzm2g324643_t02 0

grmzm2g324705_t01 0

grmzm2g324738_t01 0

grmzm2g324860_t01 0

grmzm2g324863_t01 0

grmzm2g324886_t01 0

grmzm2g324903_t01 0

grmzm2g324956_t01 0

grmzm2g324973_t01 0

grmzm2g325008_t02 -0.81

grmzm2g325019_t01 0

grmzm2g325131_t01 0

grmzm2g325143_t01 0

grmzm2g325247_t01 0

grmzm2g325350_t01 0

grmzm2g325376_t01 0

grmzm2g325462_t01 0

grmzm2g325477_t01 0

grmzm2g325513_t01 0

grmzm2g325540_t01 0

grmzm2g325575_t01 0

grmzm2g325643_t01 0

grmzm2g325650_t01 0

grmzm2g325679_t02 0

grmzm2g325683_t01 0

grmzm2g325693_t01 0

grmzm2g325749_t01 0

grmzm2g325762_t01 0

grmzm2g325804_t01 0

grmzm2g325931_t02 0

grmzm2g326066_t01 0

grmzm2g326111_t01 0

grmzm2g326116_t01 0

grmzm2g326235_t01 0

grmzm2g326263_t01 0

grmzm2g326270_t01 0

grmzm2g326272_t01 0

grmzm2g326363_t01 0

grmzm2g326472_t02 0

grmzm2g326545_t01 0

grmzm2g326643_t02 0

grmzm2g326707_t01 0

grmzm2g326734_t01 0

grmzm2g326775_t01 0

grmzm2g326783_t01 0

grmzm2g326980_t01 0

grmzm2g327042_t01 0

grmzm2g327059_t05 0

grmzm2g327174_t02 0

grmzm2g327229_t01 0

grmzm2g327234_t01 0

grmzm2g327245_t01 0

grmzm2g327247_t01 0

grmzm2g327337_t01 0

grmzm2g327354_t02 0

grmzm2g327394_t01 0

grmzm2g327400_t01 0

grmzm2g327406_t09 0

grmzm2g327459_t01 0

grmzm2g327564_t01 0

grmzm2g327595_t01 0

grmzm2g327635_t01 0

grmzm2g327686_t01 0

grmzm2g327692_t01 0

grmzm2g328094_t01 0

grmzm2g328118_t01 0

grmzm2g328168_t01 0

grmzm2g328171_t01 -1.6

grmzm2g328213_t01 0

grmzm2g328224_t01 0

grmzm2g328309_t01 0

grmzm2g328341_t01 0

grmzm2g328438_t01 0

grmzm2g328500_t02 0

grmzm2g328545_t01 0

grmzm2g328612_t01 0

grmzm2g328785_t01 0

grmzm2g328893_t01 0

grmzm2g328908_t02 0

grmzm2g329002_t02 0

grmzm2g329033_t02 0

grmzm2g329040_t03 0

grmzm2g329047_t01 0

grmzm2g329144_t01 0

grmzm2g329159_t01 1.71

grmzm2g329177_t01 0

grmzm2g329181_t01 0

grmzm2g329293_t01 0

grmzm2g329300_t01 0

grmzm2g329306_t01 0

grmzm2g329325_t01 0

grmzm2g329353_t01 0

grmzm2g329532_t03 0

grmzm2g329750_t01 0

grmzm2g329885_t02 0

grmzm2g329909_t01 0

grmzm2g329944_t01 0

grmzm2g330019_t01 0

grmzm2g330024_t02 0

grmzm2g330218_t01 0

grmzm2g330298_t01 0

grmzm2g330302_t01 0

grmzm2g330424_t01 0

grmzm2g330453_t01 0

grmzm2g330526_t01 0

grmzm2g330635_t04 -0.85

grmzm2g330650_t01 0

grmzm2g330690_t03 0

grmzm2g330719_t01 -0.91

grmzm2g330907_t01 0

grmzm2g330945_t01 -1.35

grmzm2g331015_t01 0

grmzm2g331032_t01 0

grmzm2g331085_t01 0

grmzm2g331105_t01 0

grmzm2g331253_t01 0

grmzm2g331368_t01 0

grmzm2g331374_t02 0

grmzm2g331638_t01 0

grmzm2g331720_t01 0

grmzm2g331752_t01 0

grmzm2g331754_t01 0

grmzm2g331779_t01 0

grmzm2g331782_t01 0

grmzm2g331797_t03 0

grmzm2g331833_t01 0

grmzm2g331902_t01 0

grmzm2g332225_t01 0

grmzm2g332259_t01 0

grmzm2g332368_t01 0

grmzm2g332478_t01 0

grmzm2g332495_t01 0

grmzm2g332505_t03 0

grmzm2g332522_t01 0

grmzm2g332562_t01 0

grmzm2g332654_t01 0.82

grmzm2g332687_t01 0

grmzm2g332798_t01 0

grmzm2g332809_t01 0

grmzm2g332821_t01 0

grmzm2g332838_t01 0

grmzm2g332843_t01 0

grmzm2g332976_t02 0

grmzm2g333142_t01 0

grmzm2g333433_t01 0

grmzm2g333623_t01 0

grmzm2g333641_t01 0

grmzm2g333659_t01 0

grmzm2g333756_t01 0

grmzm2g333811_t01 0

grmzm2g333833_t01 0

grmzm2g333875_t01 0

grmzm2g333926_t01 0

grmzm2g333980_t01 0

grmzm2g334041_t02 0

grmzm2g334165_t03 0

grmzm2g334409_t01 0

grmzm2g334457_t01 0

grmzm2g334574_t01 0

grmzm2g334584_t01 0

grmzm2g334592_t01 0

grmzm2g334628_t01 0

grmzm2g334655_t01 0

grmzm2g334660_t01 0

grmzm2g334791_t02 0

grmzm2g334899_t01 0

grmzm2g335019_t01 0

grmzm2g335054_t01 0

grmzm2g335126_t02 0

grmzm2g335146_t01 0

grmzm2g335229_t01 0

grmzm2g335272_t02 0

grmzm2g335280_t01 0

grmzm2g335287_t01 0

grmzm2g335618_t01 0

grmzm2g335635_t01 0

grmzm2g335638_t01 0

grmzm2g335716_t02 0

grmzm2g335720_t01 0

grmzm2g335978_t02 0

grmzm2g335989_t02 0

grmzm2g336065_t01 0

grmzm2g336285_t01 0

grmzm2g336326_t01 3.02

grmzm2g336337_t01 -1.23

grmzm2g336456_t01 0

grmzm2g336463_t01 0

grmzm2g336513_t01 0

grmzm2g336583_t01 0

grmzm2g336815_t02 0

grmzm2g336824_t01 0

grmzm2g336858_t01 0

grmzm2g336875_t01 0

grmzm2g336879_t02 0

grmzm2g336888_t01 0

grmzm2g336902_t01 0

grmzm2g336908_t02 0

grmzm2g336909_t02 0

grmzm2g336962_t01 0

grmzm2g337048_t01 0

grmzm2g337113_t03 0

grmzm2g337128_t01 0

grmzm2g337143_t01 0

grmzm2g337190_t01 0

grmzm2g337191_t01 -1.08

grmzm2g337242_t01 1.01

grmzm2g337330_t01 0

grmzm2g337425_t01 0

grmzm2g337532_t01 0

grmzm2g337534_t01 0

grmzm2g337659_t01 0

grmzm2g337706_t01 0

grmzm2g337766_t01 0

grmzm2g338037_t03 0

grmzm2g338160_t01 0

grmzm2g338205_t01 0

grmzm2g338259_t01 0

grmzm2g338465_t01 0

grmzm2g338696_t04 0

grmzm2g338702_t02 1.05

grmzm2g338916_t01 0

grmzm2g339091_t02 0

grmzm2g339122_t01 1.04

grmzm2g339151_t01 0

grmzm2g339260_t02 0

grmzm2g339336_t01 0

grmzm2g339435_t01 0

grmzm2g339488_t01 0

grmzm2g339503_t01 0

grmzm2g339510_t01 0

grmzm2g339540_t04 0

grmzm2g339563_t01 0

grmzm2g339699_t01 0

grmzm2g339736_t01 0

grmzm2g339820_t01 0

grmzm2g339848_t01 0

grmzm2g339866_t01 0

grmzm2g339957_t01 0

grmzm2g340021_t01 0

grmzm2g340065_t01 0

grmzm2g340084_t01 0

grmzm2g340130_t01 0

grmzm2g340251_t01 0

grmzm2g340279_t01 0

grmzm2g340359_t01 0

grmzm2g340416_t02 0

grmzm2g340656_t05 -1.08

grmzm2g340749_t02 0

grmzm2g340756_t02 0

grmzm2g340807_t01 0

grmzm2g341027_t01 0

grmzm2g341031_t01 0

grmzm2g341036_t01 0

grmzm2g341058_t01 0

grmzm2g341083_t01 0

grmzm2g341089_t02 0

grmzm2g341216_t01 0

grmzm2g341271_t01 0

grmzm2g341304_t01 0

grmzm2g341375_t01 0

grmzm2g341404_t01 0

grmzm2g341405_t07 0

grmzm2g341410_t01 0

grmzm2g341479_t01 0

grmzm2g341621_t01 0

grmzm2g341712_t01 0

grmzm2g341729_t01 0

grmzm2g341732_t03 0

grmzm2g341741_t01 0

grmzm2g341747_t02 0

grmzm2g341934_t01 0

grmzm2g341957_t01 0

grmzm2g342039_t01 0

grmzm2g342105_t02 0

grmzm2g342243_t02 0

grmzm2g342327_t02 0

grmzm2g342386_t01 0

grmzm2g342401_t01 0

grmzm2g342424_t01 0

grmzm2g342509_t01 0

grmzm2g342518_t02 0

grmzm2g342539_t01 0

grmzm2g342588_t01 0

grmzm2g342685_t01 0

grmzm2g342881_t01 0

grmzm2g342985_t01 1

grmzm2g343024_t02 0

grmzm2g343080_t03 0

grmzm2g343149_t01 0

grmzm2g343157_t01 0

grmzm2g343317_t01 0

grmzm2g343351_t01 0

grmzm2g343442_t01 0

grmzm2g343449_t02 0

grmzm2g343543_t01 0

grmzm2g343563_t01 0

grmzm2g343688_t02 0

grmzm2g344061_t02 0

grmzm2g344163_t01 0

grmzm2g344205_t02 0

grmzm2g344212_t03 0

grmzm2g344388_t01 0

grmzm2g344476_t01 0

grmzm2g344508_t01 0

grmzm2g344521_t01 0

grmzm2g344630_t01 -1.07

grmzm2g344634_t01 0

grmzm2g344924_t01 0

grmzm2g344933_t02 0

grmzm2g344967_t01 0

grmzm2g344977_t01 0

grmzm2g344993_t01 0

grmzm2g345039_t01 0

grmzm2g345055_t02 0

grmzm2g345493_t03 0

grmzm2g345544_t01 0

grmzm2g345622_t01 0

grmzm2g345624_t01 0

grmzm2g345667_t01 0

grmzm2g345687_t02 0

grmzm2g345700_t01 0

grmzm2g345717_t01 0

grmzm2g345725_t01 0

grmzm2g345754_t02 0

grmzm2g345795_t01 0

grmzm2g345840_t01 0

grmzm2g346132_t01 0

grmzm2g346133_t01 -1.37

grmzm2g346138_t01 1.23

grmzm2g346207_t01 0

grmzm2g346263_t03 0

grmzm2g346466_t02 0

grmzm2g346639_t01 0

grmzm2g346706_t01 0

grmzm2g346861_t01 0

grmzm2g346865_t01 0

grmzm2g346982_t01 0

grmzm2g347043_t01 0

grmzm2g347174_t02 0

grmzm2g347188_t01 0

grmzm2g347270_t01 0

grmzm2g347280_t01 0

grmzm2g347319_t01 0

grmzm2g347384_t01 0

grmzm2g347541_t01 0

grmzm2g347583_t01 0

grmzm2g347645_t01 0

grmzm2g347721_t02 0

grmzm2g347743_t02 0

grmzm2g347766_t01 0

grmzm2g347767_t03 0

grmzm2g347808_t02 0

grmzm2g347836_t01 0

grmzm2g347956_t01 0

grmzm2g348022_t01 0

grmzm2g348090_t01 0

grmzm2g348125_t01 0

grmzm2g348452_t02 0

grmzm2g348512_t05 0

grmzm2g348666_t02 0

grmzm2g348675_t01 0

grmzm2g348697_t01 0

grmzm2g348846_t01 0

grmzm2g348855_t01 0

grmzm2g348866_t02 0

grmzm2g348873_t01 0

grmzm2g348890_t01 0

grmzm2g348909_t01 0

grmzm2g348956_t01 0

grmzm2g348959_t01 0

grmzm2g349062_t01 0

grmzm2g349187_t01 -1.39

grmzm2g349268_t01 0

grmzm2g349344_t01 0

grmzm2g349603_t01 0

grmzm2g349651_t01 0

grmzm2g349665_t04 0

grmzm2g349735_t01 0

grmzm2g349745_t01 0

grmzm2g349791_t01 -2.49

grmzm2g349795_t01 0

grmzm2g349875_t01 -1.47

grmzm2g349895_t01 1.61

grmzm2g349988_t01 0

grmzm2g349996_t01 0

grmzm2g350071_t01 -2.58

grmzm2g350165_t01 0

grmzm2g350225_t01 -0.82

grmzm2g350312_t01 0

grmzm2g350319_t01 0

grmzm2g350410_t01 0

grmzm2g350447_t01 0

grmzm2g350626_t02 0

grmzm2g350628_t01 0

grmzm2g350693_t01 0

grmzm2g350711_t01 0

grmzm2g350800_t01 0

grmzm2g350802_t01 0

grmzm2g350818_t01 0

grmzm2g350918_t01 0

grmzm2g350966_t01 0

grmzm2g351018_t01 0

grmzm2g351074_t01 0

grmzm2g351125_t01 0

grmzm2g351160_t01 0

grmzm2g351259_t03 0

grmzm2g351318_t01 0

grmzm2g351382_t02 0

grmzm2g351416_t02 -1.1

grmzm2g351467_t02 0

grmzm2g351469_t01 -1.5

grmzm2g351482_t01 -1.07

grmzm2g351577_t03 0

grmzm2g351582_t01 0

grmzm2g351716_t03 0

grmzm2g351758_t02 0

grmzm2g351775_t01 0

grmzm2g351832_t01 -1.5

grmzm2g351937_t01 -1.97

grmzm2g351977_t01 -1.95

grmzm2g351990_t01 0

grmzm2g352084_t01 0

grmzm2g352129_t02 0

grmzm2g352132_t01 0

grmzm2g352159_t07 0

grmzm2g352415_t01 0

grmzm2g352428_t01 0

grmzm2g352431_t01 0

grmzm2g352439_t01 0

grmzm2g352577_t01 0

grmzm2g352607_t02 0

grmzm2g352627_t01 0

grmzm2g352695_t02 0

grmzm2g352855_t02 0

grmzm2g352883_t01 0

grmzm2g352891_t02 0

grmzm2g352912_t01 0

grmzm2g353003_t01 0

grmzm2g353097_t01 0

grmzm2g353103_t01 0

grmzm2g353147_t02 0

grmzm2g353195_t01 0

grmzm2g353209_t01 0

grmzm2g353261_t03 0

grmzm2g353276_t01 0

grmzm2g353301_t01 0

grmzm2g353313_t02 0

grmzm2g353342_t01 0

grmzm2g353444_t02 0

grmzm2g353533_t01 0

grmzm2g353548_t03 0

grmzm2g353753_t01 -1.36

grmzm2g353779_t01 0

grmzm2g353822_t01 0

grmzm2g353874_t01 0

grmzm2g353885_t01 0

grmzm2g353905_t01 0

grmzm2g354053_t01 0

grmzm2g354093_t01 0

grmzm2g354558_t01 1.37

grmzm2g354575_t01 0

grmzm2g354604_t01 0

grmzm2g354610_t03 0

grmzm2g354621_t04 0

grmzm2g354801_t01 0

grmzm2g354851_t01 0

grmzm2g354867_t01 0

grmzm2g354909_t01 1.37

grmzm2g355316_t01 0

grmzm2g355448_t01 0

grmzm2g355450_t02 0

grmzm2g355523_t01 0

grmzm2g355525_t01 0

grmzm2g355610_t01 0

grmzm2g355636_t02 0

grmzm2g355667_t01 0

grmzm2g355752_t01 0

grmzm2g355771_t01 0

grmzm2g355846_t01 0

grmzm2g355894_t01 0

grmzm2g355906_t03 0

grmzm2g356034_t01 0

grmzm2g356074_t01 0

grmzm2g356076_t01 0

grmzm2g356198_t02 0

grmzm2g356423_t01 0

grmzm2g356545_t01 0

grmzm2g356579_t03 0

grmzm2g356813_t01 0

grmzm2g356817_t01 0

grmzm2g356839_t02 0

grmzm2g356894_t02 0

grmzm2g356938_t01 0

grmzm2g357112_t01 0

grmzm2g357296_t01 0

grmzm2g357399_t02 0

grmzm2g357595_t02 0

grmzm2g357620_t01 0

grmzm2g357737_t01 0

grmzm2g357804_t01 0

grmzm2g357834_t01 0

grmzm2g357923_t01 0

grmzm2g357972_t01 0

grmzm2g358009_t01 0

grmzm2g358050_t01 0

grmzm2g358051_t01 0

grmzm2g358059_t01 0

grmzm2g358153_t01 -2.15

grmzm2g358238_t01 0

grmzm2g358279_t01 0

grmzm2g358311_t01 0

grmzm2g358381_t01 0

grmzm2g358416_t01 0

grmzm2g358467_t02 0

grmzm2g358491_t01 0

grmzm2g358540_t01 0

grmzm2g358774_t01 0

grmzm2g358877_t01 0

grmzm2g358956_t01 0

grmzm2g358987_t01 0

grmzm2g358989_t01 0

grmzm2g359018_t03 0

grmzm2g359038_t01 0

grmzm2g359070_t02 0

grmzm2g359102_t01 0

grmzm2g359127_t01 0

grmzm2g359234_t02 0

grmzm2g359260_t01 0

grmzm2g359322_t01 -1.76

grmzm2g359331_t01 0

grmzm2g359333_t02 0

grmzm2g359365_t01 0

grmzm2g359397_t01 0

grmzm2g359505_t01 0

grmzm2g359559_t02 0

grmzm2g359564_t02 0

grmzm2g359664_t02 0

grmzm2g359735_t02 0

grmzm2g359874_t01 0

grmzm2g360021_t01 0

grmzm2g360023_t01 -2.46

grmzm2g360234_t01 0

grmzm2g360339_t01 0

grmzm2g360352_t01 0

grmzm2g360429_t01 0

grmzm2g360455_t04 0

grmzm2g360517_t01 0

grmzm2g360523_t01 0

grmzm2g360541_t01 0.91

grmzm2g360589_t01 0

grmzm2g360615_t01 0

grmzm2g360626_t01 0

grmzm2g360677_t02 0

grmzm2g360688_t01 0

grmzm2g360698_t01 0

grmzm2g360835_t01 0

grmzm2g361049_t01 0

grmzm2g361064_t02 0

grmzm2g361066_t01 0

grmzm2g361074_t01 0

grmzm2g361164_t01 0

grmzm2g361220_t02 0

grmzm2g361256_t01 0

grmzm2g361391_t01 0

grmzm2g361398_t02 0

grmzm2g361569_t01 0

grmzm2g361593_t02 0

grmzm2g361602_t01 0

grmzm2g361605_t01 0

grmzm2g361611_t01 0

grmzm2g361625_t01 0

grmzm2g361652_t02 0

grmzm2g361662_t01 0

grmzm2g361688_t01 0

grmzm2g361693_t01 0

grmzm2g361699_t01 0

grmzm2g361842_t01 0

grmzm2g361847_t02 0

grmzm2g361902_t01 0

grmzm2g361993_t01 0

grmzm2g362088_t02 0

grmzm2g362306_t01 0

grmzm2g362312_t01 0

grmzm2g362368_t01 0

grmzm2g362413_t01 -1.04

grmzm2g362693_t02 0

grmzm2g362718_t02 0

grmzm2g362807_t01 -1.5

grmzm2g362848_t01 0

grmzm2g362850_t03 0

grmzm2g362883_t01 0

grmzm2g362942_t01 0

grmzm2g362949_t03 0

grmzm2g363038_t01 0

grmzm2g363138_t01 0

grmzm2g363183_t01 0

grmzm2g363186_t01 0

grmzm2g363229_t01 -1.51

grmzm2g363272_t01 -1.54

grmzm2g363447_t03 0

grmzm2g363535_t01 0

grmzm2g363537_t01 0

grmzm2g363540_t01 0

grmzm2g363545_t02 0

grmzm2g363554_t01 0

grmzm2g363595_t01 -1.28

grmzm2g363715_t01 0

grmzm2g363728_t01 0

grmzm2g363801_t02 0

grmzm2g363813_t01 0

grmzm2g363908_t02 0

grmzm2g364060_t01 0

grmzm2g364068_t03 0

grmzm2g364069_t01 0

grmzm2g364172_t02 0

grmzm2g364528_t01 0

grmzm2g364597_t01 0

grmzm2g364601_t01 0

grmzm2g364612_t01 0

grmzm2g364643_t01 0

grmzm2g364735_t01 0

grmzm2g364748_t01 0

grmzm2g364901_t02 0

grmzm2g364977_t01 0

grmzm2g364982_t01 0

grmzm2g364988_t04 0

grmzm2g365035_t01 0

grmzm2g365160_t01 0

grmzm2g365185_t01 0

grmzm2g365250_t01 0

grmzm2g365292_t01 0

grmzm2g365319_t01 0

grmzm2g365374_t01 0

grmzm2g365423_t01 0

grmzm2g365515_t01 0

grmzm2g365736_t01 0

grmzm2g365768_t01 0

grmzm2g365770_t01 0

grmzm2g365774_t01 0

grmzm2g365888_t01 0

grmzm2g365899_t01 0

grmzm2g365957_t01 0

grmzm2g365961_t01 0

grmzm2g365983_t01 0

grmzm2g366020_t01 0

grmzm2g366065_t01 0

grmzm2g366077_t01 0

grmzm2g366142_t01 -1.43

grmzm2g366146_t01 0

grmzm2g366150_t03 0

grmzm2g366199_t01 0

grmzm2g366270_t01 0

grmzm2g366301_t01 0

grmzm2g366389_t01 0.82

grmzm2g366392_t01 0.84

grmzm2g366402_t01 0

grmzm2g366622_t01 0

grmzm2g366659_t01 0

grmzm2g366681_t01 0

grmzm2g366688_t01 0

grmzm2g366698_t01 0

grmzm2g366792_t01 0

grmzm2g366935_t02 0

grmzm2g367026_t03 0

grmzm2g367092_t01 0

grmzm2g367206_t05 0

grmzm2g367340_t01 0

grmzm2g367367_t01 0

grmzm2g367564_t01 0

grmzm2g367638_t01 0

grmzm2g367650_t01 0

grmzm2g367668_t01 0

grmzm2g367678_t01 0

grmzm2g367701_t01 0

grmzm2g367857_t01 0

grmzm2g367907_t01 0

grmzm2g367941_t02 0

grmzm2g368047_t01 0

grmzm2g368151_t01 0

grmzm2g368206_t04 0

grmzm2g368312_t01 0

grmzm2g368398_t01 0

grmzm2g368410_t01 0

grmzm2g368448_t01 0

grmzm2g368491_t01 0

grmzm2g368608_t01 0

grmzm2g368698_t01 0

grmzm2g368758_t01 0

grmzm2g368799_t01 0

grmzm2g368898_t01 0

grmzm2g368902_t01 0

grmzm2g368908_t01 0

grmzm2g368909_t01 0

grmzm2g369047_t01 0

grmzm2g369130_t01 0

grmzm2g369228_t01 0

grmzm2g369237_t02 0

grmzm2g369340_t03 0

grmzm2g369652_t01 0

grmzm2g369703_t01 -1.2

grmzm2g369814_t01 0

grmzm2g369815_t02 0

grmzm2g369839_t01 0

grmzm2g369912_t01 0

grmzm2g369931_t01 0

grmzm2g369939_t01 0

grmzm2g370048_t01 0

grmzm2g370081_t01 0

grmzm2g370193_t01 0

grmzm2g370205_t02 0

grmzm2g370332_t01 0

grmzm2g370356_t01 0

grmzm2g370405_t01 0

grmzm2g370425_t01 0

grmzm2g370715_t01 0

grmzm2g370745_t01 0

grmzm2g370777_t05 0

grmzm2g370780_t01 -3.01

grmzm2g370815_t02 0

grmzm2g370915_t01 0

grmzm2g370920_t01 0

grmzm2g371062_t01 0

grmzm2g371079_t01 0

grmzm2g371316_t01 0

grmzm2g371345_t01 0

grmzm2g371606_t01 0

grmzm2g371670_t03 0

grmzm2g371721_t01 0

grmzm2g371793_t01 0

grmzm2g371795_t01 0

grmzm2g371944_t01 0

grmzm2g372074_t01 0

grmzm2g372077_t01 0

grmzm2g372297_t01 0

grmzm2g372398_t02 0

grmzm2g372475_t02 0

grmzm2g372477_t01 0

grmzm2g372553_t01 0

grmzm2g372632_t01 0

grmzm2g372870_t01 0

grmzm2g372892_t01 0

grmzm2g372928_t01 0

grmzm2g373023_t01 0

grmzm2g373124_t01 0

grmzm2g373132_t01 0

grmzm2g373175_t01 0

grmzm2g373195_t01 0

grmzm2g373341_t01 0

grmzm2g373420_t01 0

grmzm2g373435_t01 0

grmzm2g373522_t01 1.17

grmzm2g373653_t01 0

grmzm2g373717_t01 0

grmzm2g374065_t02 0

grmzm2g374068_t01 0

grmzm2g374076_t01 0

grmzm2g374084_t01 0

grmzm2g374085_t02 0

grmzm2g374088_t01 0

grmzm2g374203_t01 0

grmzm2g374252_t01 0

grmzm2g374302_t01 0

grmzm2g374385_t02 0

grmzm2g374475_t01 -1.17

grmzm2g374574_t01 0

grmzm2g374629_t01 0

grmzm2g374779_t01 0

grmzm2g374812_t01 0

grmzm2g374827_t01 0

grmzm2g374969_t02 0

grmzm2g374973_t01 0

grmzm2g375015_t02 0

grmzm2g375064_t01 0

grmzm2g375116_t02 0

grmzm2g375159_t01 -1.17

grmzm2g375172_t01 0

grmzm2g375197_t01 0

grmzm2g375222_t01 0

grmzm2g375307_t02 0

grmzm2g375310_t01 0

grmzm2g375504_t05 0

grmzm2g375675_t01 0

grmzm2g375807_t01 0

grmzm2g375904_t03 0

grmzm2g375984_t02 0

grmzm2g376067_t01 0

grmzm2g376074_t02 0

grmzm2g376305_t01 0

grmzm2g376395_t01 1.68

grmzm2g376416_t02 0

grmzm2g376444_t01 0

grmzm2g376595_t03 0

grmzm2g376727_t01 0

grmzm2g376731_t01 0

grmzm2g376918_t01 0

grmzm2g376927_t01 0

grmzm2g377079_t01 0

grmzm2g377115_t01 0

grmzm2g377131_t01 0

grmzm2g377168_t01 0

grmzm2g377215_t01 0

grmzm2g377250_t04 0

grmzm2g377311_t01 0

grmzm2g377341_t01 0

grmzm2g377357_t02 0

grmzm2g377369_t01 0

grmzm2g377487_t01 0

grmzm2g377539_t04 0

grmzm2g377589_t01 0

grmzm2g377600_t03 0

grmzm2g377609_t04 0

grmzm2g377641_t01 0

grmzm2g377761_t01 0

grmzm2g377780_t01 0

grmzm2g377797_t01 0

grmzm2g377855_t01 0

grmzm2g377887_t02 0

grmzm2g377904_t01 0

grmzm2g378040_t01 0

grmzm2g378121_t01 1.63

grmzm2g378580_t06 0

grmzm2g378604_t02 0

grmzm2g378717_t01 0

grmzm2g378762_t01 0

grmzm2g378770_t01 0

grmzm2g378836_t01 0

grmzm2g378852_t01 0

grmzm2g378907_t02 0

grmzm2g379002_t01 1.08

grmzm2g379005_t04 0

grmzm2g379021_t02 0

grmzm2g379035_t01 0

grmzm2g379044_t02 0

grmzm2g379179_t01 0

grmzm2g379252_t01 0

grmzm2g379286_t01 0

grmzm2g379327_t01 0

grmzm2g379538_t01 0

grmzm2g379540_t02 0

grmzm2g379686_t01 0

grmzm2g379706_t01 0

grmzm2g379746_t01 0

grmzm2g379758_t01 0

grmzm2g379773_t01 0

grmzm2g379780_t01 1.32

grmzm2g379804_t01 0

grmzm2g379913_t01 0

grmzm2g379964_t01 0

grmzm2g380088_t01 0

grmzm2g380091_t01 0

grmzm2g380094_t01 0

grmzm2g380113_t01 0

grmzm2g380177_t01 0

grmzm2g380184_t01 0

grmzm2g380195_t06 0

grmzm2g380197_t01 0

grmzm2g380286_t01 0

grmzm2g380319_t01 0

grmzm2g380361_t01 0

grmzm2g380414_t01 0

grmzm2g380432_t01 -1.25

grmzm2g380456_t01 0

grmzm2g380457_t01 0

grmzm2g380515_t04 1.02

grmzm2g380650_t01 0

grmzm2g380665_t01 0

grmzm2g380668_t01 0

grmzm2g380732_t01 0

grmzm2g380754_t01 0

grmzm2g380939_t01 0

grmzm2g381051_t02 0

grmzm2g381059_t02 0

grmzm2g381071_t01 0

grmzm2g381086_t01 0

grmzm2g381129_t02 0

grmzm2g381267_t01 0

grmzm2g381386_t02 0

grmzm2g381395_t01 0

grmzm2g381402_t01 0

grmzm2g381404_t01 0

grmzm2g381453_t06 0

grmzm2g381473_t01 0

grmzm2g381488_t01 0

grmzm2g381576_t01 0

grmzm2g381646_t01 0

grmzm2g381744_t01 0

grmzm2g381782_t01 -1.02

grmzm2g381802_t01 1.43

grmzm2g381822_t01 0

grmzm2g381933_t07 0.94

grmzm2g382077_t03 0

grmzm2g382104_t03 0

grmzm2g382106_t01 0

grmzm2g382273_t01 0

grmzm2g382323_t01 0

grmzm2g382341_t01 0

grmzm2g382534_t01 0

grmzm2g382568_t01 0

grmzm2g382569_t02 0

grmzm2g382673_t01 0

grmzm2g382711_t01 2.13

grmzm2g382717_t01 0

grmzm2g382774_t01 0

grmzm2g382785_t01 0

grmzm2g382914_t05 0

grmzm2g383088_t01 0

grmzm2g383122_t01 0

grmzm2g383154_t02 0

grmzm2g383210_t03 0

grmzm2g383240_t07 0

grmzm2g383404_t01 -1.15

grmzm2g383408_t01 0

grmzm2g383540_t02 0

grmzm2g383807_t01 0

grmzm2g383854_t01 0

grmzm2g384070_t02 0

grmzm2g384090_t01 0

grmzm2g384293_t03 0

grmzm2g384311_t01 1.78

grmzm2g384327_t01 0

grmzm2g384338_t01 0

grmzm2g384339_t02 0

grmzm2g384394_t01 0

grmzm2g384439_t02 0

grmzm2g384528_t01 0

grmzm2g384661_t01 0

grmzm2g384706_t01 0

grmzm2g384716_t01 0

grmzm2g384755_t01 0

grmzm2g385050_t01 0

grmzm2g385182_t01 0

grmzm2g385287_t02 0

grmzm2g385338_t01 0

grmzm2g385390_t01 -1.69

grmzm2g385413_t01 0

grmzm2g385428_t01 0

grmzm2g385925_t02 0

grmzm2g385931_t03 0

grmzm2g385945_t01 0

grmzm2g385979_t01 0

grmzm2g385989_t01 0

grmzm2g385999_t01 0

grmzm2g386046_t02 0

grmzm2g386051_t02 0

grmzm2g386113_t01 0

grmzm2g386209_t04 0

grmzm2g386228_t01 0

grmzm2g386229_t01 0

grmzm2g386261_t01 0

grmzm2g386272_t01 0

grmzm2g386281_t01 0

grmzm2g386466_t01 0

grmzm2g386525_t01 0

grmzm2g386608_t01 0

grmzm2g386643_t01 0

grmzm2g386714_t01 0.83

grmzm2g386802_t01 0

grmzm2g386817_t01 0

grmzm2g386824_t04 0

grmzm2g386923_t01 0

grmzm2g386971_t02 0

grmzm2g386973_t01 0

grmzm2g386991_t01 0

grmzm2g386998_t01 0

grmzm2g387076_t03 0

grmzm2g387360_t01 0

grmzm2g387381_t01 0

grmzm2g387383_t01 0

grmzm2g387394_t01 0

grmzm2g387419_t02 0

grmzm2g387485_t02 0

grmzm2g387528_t01 -1.08

grmzm2g387569_t01 0

grmzm2g387594_t01 0

grmzm2g387890_t01 0

grmzm2g388201_t02 0

grmzm2g388253_t02 0

grmzm2g388371_t01 0

grmzm2g388420_t01 0

grmzm2g388502_t01 0

grmzm2g388539_t01 0

grmzm2g388585_t01 0

grmzm2g388778_t01 0

grmzm2g388855_t02 0

grmzm2g388892_t01 0

grmzm2g388911_t01 0

grmzm2g388915_t01 0

grmzm2g388987_t01 0

grmzm2g389118_t01 0

grmzm2g389155_t01 0

grmzm2g389233_t01 0

grmzm2g389362_t01 0

grmzm2g389379_t01 0

grmzm2g389517_t01 0

grmzm2g389543_t01 0

grmzm2g389567_t01 -0.87

grmzm2g389645_t01 0

grmzm2g389700_t01 0

grmzm2g389768_t01 0

grmzm2g389903_t01 0

grmzm2g389948_t01 0

grmzm2g389958_t01 0

grmzm2g390013_t01 0

grmzm2g390051_t01 0

grmzm2g390076_t03 0

grmzm2g390211_t01 0

grmzm2g390221_t01 0

grmzm2g390236_t01 0

grmzm2g390400_t01 0

grmzm2g390432_t05 0

grmzm2g390436_t01 0

grmzm2g390489_t01 0

grmzm2g390562_t01 0

grmzm2g390576_t01 0

grmzm2g390641_t02 -1.43

grmzm2g390786_t01 0

grmzm2g390798_t01 0

grmzm2g390876_t01 0

grmzm2g390889_t01 0

grmzm2g390894_t01 0

grmzm2g391000_t02 0.96

grmzm2g391042_t02 0

grmzm2g391364_t01 0

grmzm2g391413_t01 0

grmzm2g391511_t01 0

grmzm2g391794_t01 0

grmzm2g391815_t01 0

grmzm2g391936_t03 0

grmzm2g391943_t01 0

grmzm2g391983_t01 0

grmzm2g392101_t01 0

grmzm2g392125_t02 -1.38

grmzm2g392168_t01 -2.12

grmzm2g392320_t02 0

grmzm2g392649_t01 0

grmzm2g392700_t02 0

grmzm2g392710_t03 0

grmzm2g392791_t01 0

grmzm2g392798_t01 0

grmzm2g392863_t02 0

grmzm2g392956_t03 0

grmzm2g392975_t02 0

grmzm2g393146_t04 0

grmzm2g393334_t01 0

grmzm2g393337_t02 0

grmzm2g393347_t01 0

grmzm2g393349_t01 0

grmzm2g393471_t01 0

grmzm2g393650_t01 0

grmzm2g393671_t01 -0.93

grmzm2g393742_t01 0

grmzm2g393897_t01 0

grmzm2g393935_t01 0

grmzm2g394027_t01 0

grmzm2g394162_t01 0

grmzm2g394212_t02 0

grmzm2g394307_t01 0

grmzm2g394321_t01 0

grmzm2g394410_t01 0

grmzm2g394450_t01 0

grmzm2g394500_t02 0

grmzm2g394507_t01 0

grmzm2g394528_t02 -1.3

grmzm2g394688_t01 0

grmzm2g394827_t06 0

grmzm2g394941_t01 0

grmzm2g394968_t01 1.96

grmzm2g395114_t02 0

grmzm2g395236_t01 0

grmzm2g395244_t03 0

grmzm2g395354_t01 0

grmzm2g395736_t01 0

grmzm2g395844_t04 0

grmzm2g395853_t01 0

grmzm2g396231_t01 0

grmzm2g396248_t01 0

grmzm2g396292_t01 0

grmzm2g396397_t01 0

grmzm2g396434_t01 0

grmzm2g396451_t02 0

grmzm2g396477_t01 0

grmzm2g396535_t01 0

grmzm2g396540_t01 0

grmzm2g396541_t01 0

grmzm2g396550_t01 0

grmzm2g396553_t01 0

grmzm2g396562_t03 0

grmzm2g396618_t01 0

grmzm2g396752_t02 0

grmzm2g396856_t03 0

grmzm2g396959_t01 0

grmzm2g396965_t01 0

grmzm2g396969_t01 0

grmzm2g397044_t01 0

grmzm2g397247_t04 0

grmzm2g397261_t01 0

grmzm2g397402_t02 0

grmzm2g397557_t01 0

grmzm2g397583_t01 0

grmzm2g397658_t01 0

grmzm2g397661_t01 0

grmzm2g397675_t01 0

grmzm2g397679_t01 0

grmzm2g397836_t02 0

grmzm2g397948_t04 0.89

grmzm2g398055_t01 0

grmzm2g398135_t01 0

grmzm2g398279_t01 0

grmzm2g398288_t01 0

grmzm2g398423_t01 0

grmzm2g398500_t01 0

grmzm2g398506_t01 0

grmzm2g398527_t01 0

grmzm2g398559_t01 0

grmzm2g398668_t02 0

grmzm2g398698_t01 0

grmzm2g398736_t01 0

grmzm2g398755_t01 0

grmzm2g398781_t03 0

grmzm2g398807_t01 -1.2

grmzm2g398809_t01 0

grmzm2g399073_t01 0

grmzm2g399114_t02 0

grmzm2g399183_t01 0

grmzm2g399207_t02 0

grmzm2g399212_t01 0

grmzm2g399284_t03 0

grmzm2g399313_t01 0

grmzm2g399320_t01 0

grmzm2g399383_t01 0

grmzm2g399433_t01 0

grmzm2g399484_t01 0

grmzm2g399677_t03 0

grmzm2g399700_t01 0

grmzm2g399750_t01 0

grmzm2g399821_t01 0

grmzm2g399844_t03 0

grmzm2g399858_t01 0

grmzm2g399890_t01 0

grmzm2g399901_t02 0

grmzm2g399952_t03 0

grmzm2g400092_t01 0

grmzm2g400135_t01 0

grmzm2g400167_t01 0

grmzm2g400173_t01 0

grmzm2g400238_t01 0

grmzm2g400268_t01 0

grmzm2g400382_t01 0

grmzm2g400470_t03 0

grmzm2g400604_t01 0

grmzm2g400694_t01 0

grmzm2g400714_t01 0

grmzm2g400720_t01 0.83

grmzm2g400725_t01 0.84

grmzm2g400837_t02 0

grmzm2g400907_t04 0

grmzm2g400928_t01 0

grmzm2g400938_t01 0

grmzm2g400961_t01 -1.48

grmzm2g401026_t01 1.25

grmzm2g401040_t01 -0.91

grmzm2g401050_t01 0

grmzm2g401062_t01 0

grmzm2g401147_t01 0

grmzm2g401308_t04 -0.85

grmzm2g401485_t01 0

grmzm2g401511_t01 0

grmzm2g401521_t01 0

grmzm2g401561_t01 0

grmzm2g401664_t01 0

grmzm2g401831_t01 0

grmzm2g401848_t01 0

grmzm2g401869_t01 0

grmzm2g401883_t01 0

grmzm2g401934_t04 0

grmzm2g402002_t02 0

grmzm2g402088_t01 0

grmzm2g402092_t01 0

grmzm2g402242_t02 0

grmzm2g402295_t02 0

grmzm2g402309_t01 0

grmzm2g402319_t01 0

grmzm2g402341_t01 0

grmzm2g402453_t01 0

grmzm2g402493_t01 0

grmzm2g402612_t05 0

grmzm2g402631_t01 0

grmzm2g402653_t03 0

grmzm2g402675_t01 0

grmzm2g402804_t01 0

grmzm2g402936_t01 -1.74

grmzm2g403149_t01 0

grmzm2g403151_t01 0

grmzm2g403162_t01 0

grmzm2g403218_t01 0

grmzm2g403371_t02 0

grmzm2g403562_t01 0

grmzm2g403609_t01 0

grmzm2g403620_t01 0

grmzm2g403636_t01 0

grmzm2g403719_t01 0

grmzm2g403797_t01 0

grmzm2g403801_t04 0

grmzm2g403828_t01 0

grmzm2g403886_t01 0

grmzm2g403915_t01 1.32

grmzm2g404043_t01 0

grmzm2g404126_t01 0

grmzm2g404132_t01 0

grmzm2g404207_t01 0

grmzm2g404316_t01 -0.86

grmzm2g404377_t01 0

grmzm2g404416_t01 0

grmzm2g404426_t01 0

grmzm2g404443_t01 0

grmzm2g404647_t01 0

grmzm2g404676_t01 0

grmzm2g404702_t01 0

grmzm2g404881_t01 0

grmzm2g404888_t02 0

grmzm2g404893_t01 0

grmzm2g404895_t01 0

grmzm2g404897_t04 0

grmzm2g404965_t01 0

grmzm2g404973_t01 0

grmzm2g405052_t01 0

grmzm2g405094_t01 0

grmzm2g405185_t01 0

grmzm2g405203_t01 0

grmzm2g405286_t01 0

grmzm2g405368_t04 0

grmzm2g405459_t01 -1.03

grmzm2g405474_t01 0

grmzm2g405499_t01 0

grmzm2g405581_t04 0

grmzm2g405662_t01 -1.26

grmzm2g405690_t01 0

grmzm2g405699_t01 0

grmzm2g405722_t01 0

grmzm2g405760_t01 0

grmzm2g405815_t01 0

grmzm2g406014_t01 0

grmzm2g406099_t01 0

grmzm2g406101_t01 0

grmzm2g406108_t01 0

grmzm2g406119_t01 0

grmzm2g406155_t01 0

grmzm2g406165_t01 0

grmzm2g406170_t04 0

grmzm2g406196_t01 -1.19

grmzm2g406268_t03 0

grmzm2g406601_t01 0

grmzm2g406603_t01 0

grmzm2g406672_t01 0

grmzm2g406674_t02 0.87

grmzm2g406712_t01 0

grmzm2g406715_t01 0

grmzm2g406746_t01 0

grmzm2g406830_t01 0

grmzm2g406859_t01 0

grmzm2g406871_t01 0

grmzm2g406951_t01 0

grmzm2g406977_t01 0

grmzm2g407044_t03 0

grmzm2g407249_t01 0

grmzm2g407287_t01 0

grmzm2g407396_t03 0

grmzm2g407748_t01 0

grmzm2g407825_t01 0

grmzm2g407996_t01 0

grmzm2g408038_t01 -1.23

grmzm2g408096_t01 0

grmzm2g408110_t01 0

grmzm2g408174_t01 0

grmzm2g408242_t02 0

grmzm2g408305_t04 0

grmzm2g408357_t01 0

grmzm2g408359_t01 1.01

grmzm2g408376_t01 0

grmzm2g408379_t02 0

grmzm2g408428_t01 0

grmzm2g408430_t01 0

grmzm2g408458_t01 0

grmzm2g408465_t01 0

grmzm2g408476_t01 0

grmzm2g408620_t02 0

grmzm2g408703_t02 0

grmzm2g408706_t03 0

grmzm2g408768_t02 0

grmzm2g408809_t01 0

grmzm2g408963_t01 0

grmzm2g408967_t03 0

grmzm2g408987_t01 0

grmzm2g408989_t01 0

grmzm2g409093_t02 0

grmzm2g409104_t01 0

grmzm2g409128_t01 0

grmzm2g409133_t02 0

grmzm2g409213_t03 0

grmzm2g409265_t02 0

grmzm2g409343_t01 0

grmzm2g409407_t01 0

grmzm2g409630_t01 0

grmzm2g409638_t01 0

grmzm2g409642_t02 0

grmzm2g409658_t01 0

grmzm2g409722_t01 0

grmzm2g409726_t01 0

grmzm2g409865_t01 0

grmzm2g409934_t01 0

grmzm2g409974_t01 0

grmzm2g409976_t01 0

grmzm2g410293_t01 0

grmzm2g410352_t01 0

grmzm2g410357_t06 0

grmzm2g410393_t01 0

grmzm2g410479_t01 0

grmzm2g410515_t01 0

grmzm2g410595_t01 0

grmzm2g410623_t01 0

grmzm2g410757_t01 0

grmzm2g410766_t01 0

grmzm2g410782_t01 0

grmzm2g410916_t02 -1.37

grmzm2g410951_t01 0

grmzm2g410963_t01 0

grmzm2g410975_t01 0

grmzm2g410991_t02 0

grmzm2g411032_t01 0

grmzm2g411071_t01 0

grmzm2g411084_t01 0

grmzm2g411159_t01 0

grmzm2g411260_t02 0

grmzm2g411333_t01 0

grmzm2g411438_t01 0

grmzm2g411536_t01 0

grmzm2g411653_t01 1.04

grmzm2g411668_t01 0

grmzm2g411899_t01 0

grmzm2g411916_t01 1.15

grmzm2g411938_t01 -3.02

grmzm2g411940_t02 0

grmzm2g411956_t01 0

grmzm2g412085_t01 0

grmzm2g412150_t01 0

grmzm2g412229_t03 0

grmzm2g412304_t01 0

grmzm2g412430_t01 0

grmzm2g412440_t01 0

grmzm2g412441_t01 0

grmzm2g412470_t02 0

grmzm2g412492_t01 0

grmzm2g412524_t01 0

grmzm2g412601_t01 -1

grmzm2g412611_t01 0

grmzm2g412888_t01 0

grmzm2g412911_t01 0

grmzm2g412986_t01 0

grmzm2g413006_t01 -1.54

grmzm2g413044_t01 0

grmzm2g413069_t01 0

grmzm2g413113_t01 0

grmzm2g413193_t01 0

grmzm2g413337_t01 0

grmzm2g413635_t01 0

grmzm2g413647_t01 0

grmzm2g413652_t02 0

grmzm2g413717_t01 0

grmzm2g413788_t01 0

grmzm2g413857_t03 -1.06

grmzm2g413897_t01 0

grmzm2g413943_t01 0

grmzm2g414002_t01 0

grmzm2g414043_t02 0

grmzm2g414047_t01 0

grmzm2g414141_t02 0

grmzm2g414159_t01 0

grmzm2g414241_t01 0

grmzm2g414315_t01 0

grmzm2g414317_t01 0

grmzm2g414373_t01 0

grmzm2g414384_t01 0

grmzm2g414387_t12 0

grmzm2g414496_t02 0

grmzm2g414525_t02 0

grmzm2g414528_t02 0

grmzm2g414537_t02 0

grmzm2g414540_t01 0

grmzm2g414569_t02 0

grmzm2g414620_t01 0

grmzm2g414621_t01 0

grmzm2g414727_t01 0

grmzm2g414805_t01 0

grmzm2g414813_t01 0

grmzm2g414834_t01 0

grmzm2g414844_t01 0

grmzm2g414848_t03 0

grmzm2g414866_t01 0

grmzm2g414915_t01 -1.81

grmzm2g414995_t02 0

grmzm2g415007_t01 0

grmzm2g415012_t01 0

grmzm2g415206_t01 0

grmzm2g415327_t03 0

grmzm2g415359_t01 0

grmzm2g415431_t01 0

grmzm2g415433_t03 0

grmzm2g415491_t02 0

grmzm2g415538_t01 0

grmzm2g415579_t01 -1.74

grmzm2g415653_t01 0

grmzm2g415772_t02 0

grmzm2g415793_t03 0

grmzm2g415811_t01 0

grmzm2g415837_t01 0

grmzm2g415846_t01 0

grmzm2g416061_t02 0

grmzm2g416069_t03 0

grmzm2g416082_t01 0

grmzm2g416120_t01 0

grmzm2g416142_t03 0

grmzm2g416156_t01 0

grmzm2g416184_t02 0

grmzm2g416216_t01 0

grmzm2g416222_t01 0

grmzm2g416308_t02 0

grmzm2g416386_t01 0

grmzm2g416388_t07 0

grmzm2g416426_t01 0

grmzm2g416484_t02 0

grmzm2g416491_t01 0

grmzm2g416498_t01 0

grmzm2g416625_t01 0

grmzm2g416632_t02 0

grmzm2g416701_t02 0

grmzm2g416751_t01 0

grmzm2g416817_t02 -0.82

grmzm2g416887_t03 0

grmzm2g417072_t01 0

grmzm2g417107_t01 0

grmzm2g417125_t03 0

grmzm2g417223_t01 0

grmzm2g417454_t01 0

grmzm2g417455_t02 0

grmzm2g417658_t01 0

grmzm2g417682_t01 0

grmzm2g417715_t01 0

grmzm2g417765_t03 0

grmzm2g417770_t01 0

grmzm2g417843_t02 0

grmzm2g417859_t01 0

grmzm2g417963_t01 0

grmzm2g418037_t01 0

grmzm2g418194_t01 0

grmzm2g418206_t01 0

grmzm2g418258_t01 0

grmzm2g418263_t01 0

grmzm2g418294_t01 0

grmzm2g418343_t01 -1.86

grmzm2g418415_t01 1.23

grmzm2g418515_t01 0

grmzm2g418604_t02 0

grmzm2g418689_t01 -1.06

grmzm2g418752_t01 0

grmzm2g418805_t01 0

grmzm2g418916_t01 0

grmzm2g418956_t01 0

grmzm2g419085_t01 0

grmzm2g419111_t01 0

grmzm2g419182_t01 0

grmzm2g419239_t01 0

grmzm2g419267_t02 0

grmzm2g419290_t01 0

grmzm2g419305_t01 0.96

grmzm2g419328_t01 0

grmzm2g419606_t01 0

grmzm2g419643_t01 0

grmzm2g419739_t02 0

grmzm2g419777_t01 0

grmzm2g419806_t01 0

grmzm2g419836_t01 0

grmzm2g419844_t01 0

grmzm2g419891_t05 0

grmzm2g419938_t01 -2.48

grmzm2g419953_t01 0

grmzm2g419969_t01 0

grmzm2g419994_t01 0

grmzm2g420055_t02 0

grmzm2g420119_t01 0

grmzm2g420121_t01 0

grmzm2g420357_t01 0

grmzm2g420432_t01 0

grmzm2g420436_t01 0

grmzm2g420469_t02 0

grmzm2g420684_t02 0

grmzm2g420713_t01 0

grmzm2g420723_t01 0

grmzm2g420733_t01 0

grmzm2g420743_t01 0

grmzm2g420772_t01 0

grmzm2g420789_t01 0

grmzm2g420856_t02 0

grmzm2g420865_t01 0

grmzm2g420882_t01 0

grmzm2g420883_t01 0

grmzm2g420910_t01 0

grmzm2g420926_t02 0

grmzm2g421033_t01 -2.97

grmzm2g421042_t01 0

grmzm2g421212_t01 0

grmzm2g421231_t01 0

grmzm2g421234_t01 0

grmzm2g421240_t01 0

grmzm2g421256_t01 0

grmzm2g421279_t01 0

grmzm2g421415_t01 0

grmzm2g421491_t01 0

grmzm2g421500_t01 0

grmzm2g421541_t03 0

grmzm2g421604_t03 0

grmzm2g421680_t01 0

grmzm2g421690_t01 0

grmzm2g421791_t01 0

grmzm2g421857_t01 0

grmzm2g421883_t01 0

grmzm2g421921_t01 0

grmzm2g422090_t01 0

grmzm2g422175_t01 0

grmzm2g422205_t02 0

grmzm2g422210_t01 0

grmzm2g422340_t01 0

grmzm2g422464_t02 0

grmzm2g422499_t01 0

grmzm2g422510_t01 0

grmzm2g422516_t02 0

grmzm2g422576_t01 0

grmzm2g422641_t01 0

grmzm2g422644_t01 0

grmzm2g422649_t01 0

grmzm2g422651_t01 0

grmzm2g422670_t01 0

grmzm2g422671_t01 0

grmzm2g422938_t01 0

grmzm2g423027_t04 0

grmzm2g423111_t01 0

grmzm2g423129_t01 0

grmzm2g423137_t01 0

grmzm2g423169_t03 0

grmzm2g423193_t01 0

grmzm2g423202_t01 0

grmzm2g423292_t01 0

grmzm2g423301_t01 0

grmzm2g423337_t03 0

grmzm2g423456_t02 0

grmzm2g423476_t01 0

grmzm2g423538_t01 0

grmzm2g423561_t01 0

grmzm2g423643_t01 0

grmzm2g423831_t02 0

grmzm2g423833_t01 0

grmzm2g423851_t01 0

grmzm2g423861_t01 0

grmzm2g423886_t01 0

grmzm2g423900_t01 0

grmzm2g423917_t01 0

grmzm2g423956_t01 0

grmzm2g424053_t01 0

grmzm2g424088_t02 0

grmzm2g424112_t01 0

grmzm2g424181_t01 0

grmzm2g424205_t01 2.37

grmzm2g424241_t02 0

grmzm2g424491_t01 0

grmzm2g424577_t01 0

grmzm2g424582_t01 0

grmzm2g424595_t01 0

grmzm2g424783_t03 0

grmzm2g424832_t01 0

grmzm2g424857_t01 0

grmzm2g424873_t06 0

grmzm2g424908_t01 0

grmzm2g425004_t01 0

grmzm2g425129_t01 0

grmzm2g425249_t01 0

grmzm2g425377_t01 0

grmzm2g425430_t01 0

grmzm2g425482_t01 0

grmzm2g425500_t01 0

grmzm2g425708_t01 0

grmzm2g425728_t01 0

grmzm2g425729_t01 0

grmzm2g425731_t01 0

grmzm2g425736_t01 0

grmzm2g425774_t01 0

grmzm2g425863_t03 0

grmzm2g426108_t02 0

grmzm2g426122_t01 0

grmzm2g426140_t01 0

grmzm2g426154_t01 0

grmzm2g426156_t02 0

grmzm2g426200_t02 0

grmzm2g426229_t03 0

grmzm2g426275_t01 0

grmzm2g426336_t01 0

grmzm2g426415_t01 0

grmzm2g426461_t02 0

grmzm2g426556_t01 0

grmzm2g426591_t04 0

grmzm2g426613_t01 0

grmzm2g426735_t01 0

grmzm2g426802_t01 0

grmzm2g426888_t01 0

grmzm2g426953_t02 0

grmzm2g426964_t01 0

grmzm2g427014_t01 0

grmzm2g427031_t01 0

grmzm2g427054_t01 0

grmzm2g427067_t01 0

grmzm2g427087_t01 0

grmzm2g427090_t01 0

grmzm2g427097_t02 0

grmzm2g427337_t02 0

grmzm2g427350_t01 0

grmzm2g427451_t01 0

grmzm2g427468_t01 0

grmzm2g427529_t01 0

grmzm2g427692_t01 -1.42

grmzm2g427697_t01 -1.33

grmzm2g427729_t01 0

grmzm2g427815_t01 0

grmzm2g427826_t01 0

grmzm2g427910_t01 0

grmzm2g428096_t01 0

grmzm2g428168_t01 0

grmzm2g428184_t01 0

grmzm2g428242_t05 0

grmzm2g428280_t01 0

grmzm2g428356_t01 0

grmzm2g428386_t01 0

grmzm2g428391_t02 0

grmzm2g428410_t02 0

grmzm2g428470_t01 0

grmzm2g428471_t01 0

grmzm2g428518_t02 0

grmzm2g428579_t01 0

grmzm2g428765_t01 0

grmzm2g428933_t01 0

grmzm2g428964_t01 0

grmzm2g429000_t01 0

grmzm2g429035_t01 0

grmzm2g429113_t01 0

grmzm2g429118_t01 0

grmzm2g429169_t01 0

grmzm2g429213_t01 0

grmzm2g429237_t01 0

grmzm2g429241_t01 0

grmzm2g429254_t01 0

grmzm2g429396_t01 -1.2

grmzm2g429442_t01 0

grmzm2g429493_t01 0

grmzm2g429540_t01 0

grmzm2g429560_t01 0

grmzm2g429714_t01 0

grmzm2g429759_t04 0

grmzm2g429778_t01 0

grmzm2g429807_t01 0

grmzm2g429842_t02 0

grmzm2g429940_t01 0

grmzm2g429955_t01 -2.03

grmzm2g429972_t01 0

grmzm2g429994_t01 -2.84

grmzm2g430039_t01 -2.84

grmzm2g430152_t01 0

grmzm2g430362_t01 0

grmzm2g430365_t01 0

grmzm2g430387_t01 0

grmzm2g430394_t01 0

grmzm2g430600_t04 0

grmzm2g430675_t01 0

grmzm2g430680_t02 0

grmzm2g430685_t03 0

grmzm2g430710_t02 0

grmzm2g430745_t01 0

grmzm2g430780_t01 0

grmzm2g430807_t01 0

grmzm2g430849_t01 0

grmzm2g430871_t01 0

grmzm2g430946_t01 0

grmzm2g431030_t01 0

grmzm2g431039_t01 0

grmzm2g431157_t01 0

grmzm2g431243_t02 0

grmzm2g431251_t01 0

grmzm2g431288_t01 0

grmzm2g431291_t02 0

grmzm2g431306_t01 0

grmzm2g431309_t01 0

grmzm2g431314_t01 0

grmzm2g431350_t04 0

grmzm2g431506_t01 0

grmzm2g431509_t01 0

grmzm2g431524_t04 0

grmzm2g431703_t01 0

grmzm2g431708_t01 0

grmzm2g431821_t01 0

grmzm2g431850_t01 0

grmzm2g431900_t01 0

grmzm2g432128_t01 0

grmzm2g432167_t01 0

grmzm2g432335_t02 0

grmzm2g432390_t01 0

grmzm2g432480_t01 0

grmzm2g432513_t01 1.22

grmzm2g432566_t01 0

grmzm2g432642_t02 0

grmzm2g432662_t01 0

grmzm2g432722_t01 0

grmzm2g432738_t01 0

grmzm2g432774_t03 0

grmzm2g432796_t01 0

grmzm2g432801_t01 0

grmzm2g432850_t01 0

grmzm2g433002_t01 0

grmzm2g433025_t02 0

grmzm2g433117_t01 0

grmzm2g433162_t01 0

grmzm2g433184_t01 -1.2

grmzm2g433333_t01 0.9

grmzm2g433433_t01 0

grmzm2g433579_t01 0

grmzm2g433591_t02 0

grmzm2g433731_t01 0

grmzm2g433767_t03 0

grmzm2g433801_t01 0

grmzm2g434173_t01 0

grmzm2g434194_t01 0

grmzm2g434219_t01 0

grmzm2g434277_t01 0

grmzm2g434301_t01 0

grmzm2g434383_t01 0

grmzm2g434541_t01 -1.01

grmzm2g434557_t01 0

grmzm2g434669_t01 0

grmzm2g434696_t03 0

grmzm2g434809_t01 0

grmzm2g435034_t01 0

grmzm2g435104_t01 0

grmzm2g435274_t01 0

grmzm2g435310_t01 -1.12

grmzm2g435338_t02 0

grmzm2g435445_t01 0

grmzm2g435587_t01 0

grmzm2g435589_t01 0

grmzm2g435592_t02 0

grmzm2g435627_t01 0

grmzm2g435979_t01 0

grmzm2g436000_t01 0

grmzm2g436001_t01 0

grmzm2g436084_t01 1.51

grmzm2g436092_t01 0

grmzm2g436199_t02 0

grmzm2g436226_t01 0

grmzm2g436295_t01 0

grmzm2g436305_t01 0

grmzm2g436319_t02 0

grmzm2g436328_t01 0

grmzm2g436511_t01 0

grmzm2g436593_t01 0

grmzm2g436707_t01 0

grmzm2g436710_t02 0

grmzm2g436730_t01 0

grmzm2g436835_t01 0

grmzm2g436981_t02 0

grmzm2g436986_t01 0

grmzm2g437119_t03 0

grmzm2g437215_t01 0

grmzm2g437314_t01 0

grmzm2g437456_t01 0

grmzm2g437460_t02 0

grmzm2g437512_t01 0

grmzm2g437575_t01 0

grmzm2g437675_t01 0

grmzm2g437776_t01 0

grmzm2g437912_t01 0

grmzm2g437977_t01 0

grmzm2g438007_t01 0

grmzm2g438176_t01 0

grmzm2g438178_t01 0

grmzm2g438210_t01 0

grmzm2g438239_t01 0

grmzm2g438243_t01 0

grmzm2g438299_t01 0

grmzm2g438386_t01 0

grmzm2g438429_t01 0

grmzm2g438438_t01 0

grmzm2g438524_t01 0

grmzm2g438551_t01 0

grmzm2g438561_t01 0

grmzm2g438583_t01 0

grmzm2g438622_t01 0

grmzm2g438673_t02 0

grmzm2g438722_t01 0

grmzm2g438859_t01 0

grmzm2g438895_t01 0

grmzm2g438938_t02 0

grmzm2g439195_t01 0

grmzm2g439201_t04 0

grmzm2g439203_t03 0

grmzm2g439276_t01 0

grmzm2g439311_t01 0

grmzm2g439339_t04 0

grmzm2g439400_t01 0

grmzm2g439457_t01 0

grmzm2g439799_t01 0

grmzm2g439884_t02 0

grmzm2g439897_t01 0

grmzm2g439950_t04 0

grmzm2g439951_t01 0

grmzm2g440005_t01 0.99

grmzm2g440009_t01 0

grmzm2g440198_t01 0

grmzm2g440208_t01 0

grmzm2g440221_t01 0

grmzm2g440259_t05 0

grmzm2g440313_t01 0

grmzm2g440349_t01 0

grmzm2g440529_t01 0.99

grmzm2g440537_t01 0

grmzm2g440562_t01 0

grmzm2g440569_t01 0

grmzm2g440614_t02 0

grmzm2g440746_t02 0.82

grmzm2g440866_t02 -1.29

grmzm2g440916_t01 0

grmzm2g440925_t01 0

grmzm2g440943_t01 0

grmzm2g440949_t01 0

grmzm2g440968_t01 0

grmzm2g441144_t01 0

grmzm2g441325_t02 0

grmzm2g441343_t01 0

grmzm2g441347_t02 0

grmzm2g441381_t01 0

grmzm2g441489_t01 0

grmzm2g441565_t01 0

grmzm2g441583_t01 0

grmzm2g441656_t01 0

grmzm2g441888_t01 0

grmzm2g441903_t01 0

grmzm2g442000_t01 0

grmzm2g442057_t01 0

grmzm2g442195_t02 0

grmzm2g442277_t01 0

grmzm2g442404_t04 0

grmzm2g442523_t03 0

grmzm2g442551_t02 0

grmzm2g442658_t03 0

grmzm2g442675_t01 0

grmzm2g442685_t01 0

grmzm2g442763_t01 0

grmzm2g442804_t01 0

grmzm2g442831_t01 0

grmzm2g443119_t01 0

grmzm2g443187_t02 0

grmzm2g443193_t01 0

grmzm2g443264_t01 0

grmzm2g443265_t01 0

grmzm2g443272_t01 0.9

grmzm2g443287_t06 0

grmzm2g443340_t01 0

grmzm2g443447_t01 0

grmzm2g443453_t01 0

grmzm2g443509_t03 0

grmzm2g443525_t02 0

grmzm2g443560_t02 0

grmzm2g443655_t01 0

grmzm2g443728_t02 0

grmzm2g443785_t01 0

grmzm2g443814_t01 0

grmzm2g443881_t01 0

grmzm2g443888_t01 0

grmzm2g443903_t01 0

grmzm2g443939_t01 0

grmzm2g443953_t04 0

grmzm2g443985_t03 0

grmzm2g444029_t03 0

grmzm2g444075_t01 0

grmzm2g444138_t01 0

grmzm2g444141_t01 0

grmzm2g444438_t01 0

grmzm2g444533_t01 0

grmzm2g444567_t01 -2.22

grmzm2g444623_t02 0

grmzm2g444643_t01 0

grmzm2g444692_t02 0

grmzm2g444743_t01 0

grmzm2g444748_t01 0

grmzm2g444762_t01 0

grmzm2g444801_t01 0

grmzm2g444808_t01 0

grmzm2g444845_t02 0

grmzm2g445057_t01 0

grmzm2g445100_t01 0

grmzm2g445421_t01 0

grmzm2g445478_t03 0

grmzm2g445575_t04 0

grmzm2g445602_t01 0

grmzm2g445613_t02 0

grmzm2g445617_t01 1.13

grmzm2g445634_t01 0

grmzm2g445905_t03 0

grmzm2g445944_t01 0

grmzm2g446050_t01 0

grmzm2g446171_t01 0

grmzm2g446213_t01 -1.76

grmzm2g446218_t01 0

grmzm2g446234_t01 0

grmzm2g446313_t01 0

grmzm2g446426_t01 0

grmzm2g446515_t01 0

grmzm2g446625_t03 0

grmzm2g446872_t01 0

grmzm2g446895_t04 0

grmzm2g446921_t02 0

grmzm2g446929_t02 0

grmzm2g446960_t01 0

grmzm2g447195_t03 0

grmzm2g447271_t01 0

grmzm2g447406_t01 0

grmzm2g447433_t10 0

grmzm2g447455_t01 0

grmzm2g447535_t01 0

grmzm2g447617_t03 0

grmzm2g447632_t01 0

grmzm2g447691_t05 0

grmzm2g447745_t01 0

grmzm2g447785_t01 0

grmzm2g447857_t01 0

grmzm2g447867_t01 0

grmzm2g447976_t02 0

grmzm2g447987_t01 0

grmzm2g448001_t01 0

grmzm2g448104_t01 0

grmzm2g448161_t01 0

grmzm2g448174_t02 0

grmzm2g448185_t01 0

grmzm2g448213_t01 0

grmzm2g448241_t02 0

grmzm2g448258_t02 0

grmzm2g448282_t01 0

grmzm2g448330_t01 0

grmzm2g448446_t01 0

grmzm2g448456_t01 0

grmzm2g448603_t01 0

grmzm2g448607_t01 0

grmzm2g448687_t02 0

grmzm2g448715_t01 0

grmzm2g448834_t04 0

grmzm2g449033_t01 0

grmzm2g449065_t01 0

grmzm2g449083_t01 0

grmzm2g449123_t02 0

grmzm2g449160_t01 0

grmzm2g449163_t01 0

grmzm2g449165_t01 0

grmzm2g449177_t01 0

grmzm2g449200_t01 0

grmzm2g449219_t01 0

grmzm2g449274_t01 0

grmzm2g449355_t02 0

grmzm2g449496_t02 0

grmzm2g449569_t01 0

grmzm2g449681_t02 0

grmzm2g449695_t01 0

grmzm2g449709_t01 0

grmzm2g449779_t01 0

grmzm2g449817_t01 0

grmzm2g449893_t01 0

grmzm2g449909_t01 0

grmzm2g449950_t01 0

grmzm2g449951_t01 0

grmzm2g450125_t01 0

grmzm2g450163_t01 0

grmzm2g450233_t02 0

grmzm2g450424_t01 1.69

grmzm2g450488_t01 0

grmzm2g450498_t02 -0.82

grmzm2g450659_t03 0

grmzm2g450851_t02 0

grmzm2g450920_t01 0

grmzm2g450937_t07 0

grmzm2g450974_t01 0

grmzm2g451007_t01 0

grmzm2g451132_t02 0

grmzm2g451147_t01 0

grmzm2g451187_t01 0

grmzm2g451224_t01 0

grmzm2g451254_t01 0

grmzm2g451281_t04 0

grmzm2g451314_t02 0

grmzm2g451325_t01 0

grmzm2g451327_t01 0

grmzm2g451357_t02 0

grmzm2g451366_t02 0

grmzm2g451443_t01 0

grmzm2g451483_t01 0

grmzm2g451528_t01 0

grmzm2g451604_t03 0

grmzm2g451672_t03 0

grmzm2g451716_t02 0

grmzm2g451792_t03 0

grmzm2g451856_t02 0

grmzm2g451861_t01 -1.59

grmzm2g451882_t01 0

grmzm2g452016_t01 0

grmzm2g452026_t02 0

grmzm2g452067_t02 0

grmzm2g452142_t01 0

grmzm2g452523_t02 0

grmzm2g452529_t01 0

grmzm2g452564_t01 0

grmzm2g452580_t01 0

grmzm2g452630_t02 0

grmzm2g452633_t01 0

grmzm2g452717_t02 0

grmzm2g452811_t01 0

grmzm2g452896_t01 0

grmzm2g452935_t01 0

grmzm2g452955_t01 0

grmzm2g453111_t02 0

grmzm2g453296_t01 0

grmzm2g453320_t01 0

grmzm2g453349_t01 0

grmzm2g453388_t01 0

grmzm2g453424_t01 0

grmzm2g453485_t01 0

grmzm2g453684_t01 0

grmzm2g453772_t01 0

grmzm2g453805_t01 0

grmzm2g453832_t01 0

grmzm2g454081_t02 0

grmzm2g454176_t02 0

grmzm2g454204_t02 0

grmzm2g454299_t01 0

grmzm2g454425_t02 0

grmzm2g454891_t01 0

grmzm2g454928_t06 0

grmzm2g454952_t01 0

grmzm2g455068_t01 0

grmzm2g455075_t01 0

grmzm2g455085_t01 0

grmzm2g455122_t01 -1.14

grmzm2g455128_t01 0

grmzm2g455321_t01 0

grmzm2g455433_t01 0

grmzm2g455476_t01 0

grmzm2g455564_t01 0

grmzm2g455587_t01 0

grmzm2g455687_t01 0

grmzm2g455769_t01 0

grmzm2g455809_t01 0

grmzm2g455817_t01 0

grmzm2g455828_t01 0

grmzm2g455869_t01 0

grmzm2g455909_t01 0

grmzm2g456000_t03 0

grmzm2g456023_t02 0

grmzm2g456059_t01 0

grmzm2g456086_t01 0

grmzm2g456123_t01 0

grmzm2g456132_t02 0

grmzm2g456217_t01 0

grmzm2g456357_t01 0

grmzm2g456367_t01 0

grmzm2g456422_t01 0

grmzm2g456471_t01 1.08

grmzm2g456473_t01 0

grmzm2g456564_t01 0

grmzm2g456568_t01 0

grmzm2g456570_t02 0

grmzm2g456603_t01 0

grmzm2g456626_t01 0

grmzm2g456768_t01 0

grmzm2g456835_t02 0

grmzm2g456960_t01 0

grmzm2g457003_t01 0

grmzm2g457040_t03 -1.24

grmzm2g457147_t01 0

grmzm2g457178_t01 0

grmzm2g457201_t03 0

grmzm2g457211_t01 0

grmzm2g457267_t01 0

grmzm2g457309_t01 0

grmzm2g457361_t01 0

grmzm2g457381_t02 0

grmzm2g457411_t01 0

grmzm2g457415_t01 1.28

grmzm2g457424_t01 0

grmzm2g457467_t01 0

grmzm2g457534_t01 0

grmzm2g457621_t01 0

grmzm2g457672_t01 0

grmzm2g457697_t02 0

grmzm2g457782_t01 0

grmzm2g457789_t01 0

grmzm2g457889_t01 0

grmzm2g457929_t01 -1.09

grmzm2g458009_t01 0

grmzm2g458164_t01 0

grmzm2g458283_t01 0

grmzm2g458538_t01 0

grmzm2g458548_t02 0

grmzm2g458549_t01 0

grmzm2g458596_t01 0

grmzm2g458613_t01 0

grmzm2g458665_t01 0

grmzm2g458728_t01 0

grmzm2g458755_t01 0

grmzm2g458824_t02 0

grmzm2g459063_t02 0

grmzm2g459291_t03 0

grmzm2g459363_t01 0

grmzm2g459391_t01 0

grmzm2g459474_t01 0

grmzm2g459484_t01 0

grmzm2g459532_t01 0

grmzm2g459563_t01 0

grmzm2g459581_t03 0

grmzm2g459642_t02 0

grmzm2g459702_t04 0

grmzm2g459715_t01 0

grmzm2g459811_t01 0

grmzm2g459861_t01 0

grmzm2g459874_t01 0

grmzm2g460012_t01 0

grmzm2g460078_t05 0

grmzm2g460090_t01 0

grmzm2g460383_t01 0

grmzm2g460396_t01 0

grmzm2g460406_t02 0

grmzm2g460472_t01 0

grmzm2g460559_t01 0

grmzm2g460566_t01 0

grmzm2g460617_t01 0

grmzm2g460860_t01 0

grmzm2g460861_t01 0

grmzm2g460988_t01 1.43

grmzm2g461139_t01 0

grmzm2g461145_t01 0

grmzm2g461269_t01 0

grmzm2g461356_t01 0

grmzm2g461427_t01 0

grmzm2g461569_t01 0

grmzm2g461586_t01 0

grmzm2g461716_t01 0

grmzm2g461791_t01 0

grmzm2g461793_t01 0

grmzm2g461808_t02 0

grmzm2g461861_t01 0

grmzm2g461936_t02 0

grmzm2g461948_t01 0

grmzm2g461988_t01 0

grmzm2g462062_t01 0

grmzm2g462118_t01 0

grmzm2g462261_t01 0

grmzm2g462325_t01 0

grmzm2g462613_t01 0

grmzm2g462625_t01 0

grmzm2g462639_t01 0

grmzm2g462690_t01 0

grmzm2g462803_t02 0

grmzm2g462860_t01 0

grmzm2g462883_t01 0

grmzm2g462885_t01 0

grmzm2g462904_t01 0

grmzm2g463032_t01 0

grmzm2g463227_t02 0

grmzm2g463257_t01 0

grmzm2g463267_t04 0

grmzm2g463280_t01 0

grmzm2g463462_t02 0

grmzm2g463464_t02 0

grmzm2g463493_t01 -1.44

grmzm2g463498_t01 -1.39

grmzm2g463525_t01 0

grmzm2g463545_t01 0

grmzm2g463574_t01 1.7

grmzm2g463580_t01 0

grmzm2g463785_t01 0

grmzm2g463891_t01 0

grmzm2g463904_t03 0

grmzm2g463913_t02 0

grmzm2g463953_t01 0

grmzm2g463996_t01 0

grmzm2g464000_t04 0

grmzm2g464013_t01 0

grmzm2g464043_t01 0

grmzm2g464137_t01 0

grmzm2g464176_t01 0

grmzm2g464308_t01 0

grmzm2g464326_t01 0

grmzm2g464328_t01 0

grmzm2g464401_t01 0

grmzm2g464491_t01 0

grmzm2g464515_t01 0

grmzm2g464575_t01 0

grmzm2g464754_t01 0

grmzm2g464782_t09 0

grmzm2g464885_t02 0

grmzm2g464891_t01 0

grmzm2g464976_t04 0

grmzm2g464985_t01 -0.9

grmzm2g465046_t01 0

grmzm2g465086_t01 0

grmzm2g465087_t01 0

grmzm2g465169_t01 0

grmzm2g465333_t01 0

grmzm2g465444_t01 0

grmzm2g465553_t01 0

grmzm2g465685_t01 0

grmzm2g465764_t01 0

grmzm2g465771_t01 0

grmzm2g465806_t01 0

grmzm2g465833_t01 0

grmzm2g465849_t01 0

grmzm2g465868_t01 -2.1

grmzm2g466032_t01 0

grmzm2g466139_t01 0

grmzm2g466224_t01 0

grmzm2g466265_t01 0

grmzm2g466270_t04 0

grmzm2g466281_t03 0

grmzm2g466292_t01 0

grmzm2g466298_t01 0

grmzm2g466309_t01 0

grmzm2g466365_t01 0

grmzm2g466534_t01 0

grmzm2g466543_t01 0

grmzm2g466545_t01 0

grmzm2g466560_t01 0

grmzm2g466578_t01 0

grmzm2g466780_t01 0

grmzm2g466823_t01 0

grmzm2g466833_t01 0

grmzm2g466909_t01 0

grmzm2g467007_t04 0

grmzm2g467016_t01 0

grmzm2g467059_t03 0

grmzm2g467069_t04 0

grmzm2g467086_t02 0

grmzm2g467112_t01 0

grmzm2g467154_t01 0

grmzm2g467159_t01 0

grmzm2g467169_t02 0

grmzm2g467184_t01 0

grmzm2g467338_t02 0

grmzm2g467356_t01 0

grmzm2g467370_t01 0

grmzm2g467424_t01 0

grmzm2g467435_t02 0

grmzm2g467446_t01 0

grmzm2g467466_t01 0

grmzm2g467576_t01 0

grmzm2g467640_t01 0

grmzm2g467682_t01 0

grmzm2g467717_t01 0

grmzm2g467799_t01 0

grmzm2g467886_t03 0

grmzm2g467893_t01 0

grmzm2g467907_t01 0

grmzm2g467992_t01 0

grmzm2g468132_t01 0

grmzm2g468260_t03 0

grmzm2g468405_t01 0

grmzm2g468439_t01 0

grmzm2g468479_t01 0

grmzm2g468525_t01 0

grmzm2g468535_t03 0

grmzm2g468574_t01 0

grmzm2g468661_t01 0

grmzm2g468682_t01 0

grmzm2g468693_t03 0

grmzm2g468756_t01 0

grmzm2g468799_t01 0

grmzm2g468855_t03 0

grmzm2g468932_t01 0

grmzm2g469012_t01 0

grmzm2g469111_t01 0

grmzm2g469142_t01 0

grmzm2g469150_t01 0

grmzm2g469224_t01 0

grmzm2g469380_t02 0

grmzm2g469409_t01 0

grmzm2g469414_t01 0

grmzm2g469499_t01 0

grmzm2g469523_t01 0

grmzm2g469551_t02 0

grmzm2g469747_t03 0

grmzm2g469795_t01 0

grmzm2g469807_t01 0

grmzm2g469809_t02 0

grmzm2g469873_t01 0

grmzm2g469898_t02 0

grmzm2g469901_t01 0

grmzm2g469903_t01 0

grmzm2g469920_t01 0

grmzm2g469969_t01 0

grmzm2g470427_t01 0

grmzm2g470442_t03 0

grmzm2g470461_t01 0

grmzm2g470513_t01 0

grmzm2g470556_t01 0

grmzm2g470589_t02 0

grmzm2g470740_t01 0

grmzm2g470862_t01 0

grmzm2g470882_t02 0

grmzm2g470942_t04 0

grmzm2g471027_t01 0

grmzm2g471065_t01 0

grmzm2g471083_t01 0

grmzm2g471208_t01 0

grmzm2g471253_t01 0

grmzm2g471269_t02 0

grmzm2g471348_t01 0

grmzm2g471357_t03 0

grmzm2g471479_t01 1.55

grmzm2g471517_t01 0

grmzm2g471529_t02 0

grmzm2g471600_t01 0

grmzm2g471733_t01 0

grmzm2g471805_t03 0

grmzm2g471904_t03 0

grmzm2g471926_t01 0

grmzm2g472052_t01 0

grmzm2g472060_t02 0

grmzm2g472064_t01 0

grmzm2g472167_t02 0

grmzm2g472171_t03 0

grmzm2g472231_t01 0

grmzm2g472266_t01 0

grmzm2g472346_t02 -0.92

grmzm2g472378_t01 0

grmzm2g472382_t02 0

grmzm2g472428_t02 0

grmzm2g472432_t01 0

grmzm2g472453_t01 0

grmzm2g472565_t01 0

grmzm2g472625_t01 0

grmzm2g472643_t01 -0.97

grmzm2g472651_t02 0

grmzm2g472671_t01 0

grmzm2g472693_t01 0

grmzm2g472696_t01 0

grmzm2g472770_t01 0

grmzm2g472827_t01 0

grmzm2g472852_t01 0

grmzm2g472991_t01 0

grmzm2g473001_t02 0

grmzm2g473016_t01 0

grmzm2g473111_t02 0

grmzm2g473138_t01 0

grmzm2g473147_t01 0

grmzm2g473162_t01 0

grmzm2g473356_t01 0.88

grmzm2g473367_t01 0

grmzm2g473385_t01 0

grmzm2g473456_t01 -0.99

grmzm2g473463_t01 0

grmzm2g473480_t01 -1.12

grmzm2g473533_t05 0

grmzm2g473586_t01 0

grmzm2g473676_t01 0

grmzm2g473709_t01 0

grmzm2g473788_t01 0

grmzm2g473811_t01 0

grmzm2g473906_t01 0

grmzm2g473976_t02 0

grmzm2g474019_t01 0

grmzm2g474092_t01 0

grmzm2g474119_t01 -1.72

grmzm2g474190_t01 0

grmzm2g474236_t01 0

grmzm2g474332_t02 0

grmzm2g474531_t01 0

grmzm2g474537_t01 0

grmzm2g474546_t03 0

grmzm2g474554_t01 0

grmzm2g474555_t01 0

grmzm2g474575_t01 0

grmzm2g474651_t01 0

grmzm2g474658_t02 0

grmzm2g474726_t03 0

grmzm2g474755_t01 0

grmzm2g474769_t02 0

grmzm2g474777_t01 0

grmzm2g474798_t01 0

grmzm2g474883_t01 0

grmzm2g475017_t04 0

grmzm2g475059_t02 0

grmzm2g475263_t01 0

grmzm2g475265_t01 0

grmzm2g475289_t02 0

grmzm2g475293_t03 0

grmzm2g475305_t01 0

grmzm2g475349_t01 0

grmzm2g475362_t01 0

grmzm2g475380_t01 0

grmzm2g475495_t01 0

grmzm2g475504_t01 0

grmzm2g475554_t01 0

grmzm2g475583_t01 0

grmzm2g475683_t01 0

grmzm2g475743_t01 0

grmzm2g475867_t01 0

grmzm2g475882_t02 0

grmzm2g475897_t01 0

grmzm2g476009_t03 0

grmzm2g476230_t01 -1.45

grmzm2g476448_t01 0

grmzm2g476538_t01 0

grmzm2g476555_t01 0

grmzm2g476637_t04 0

grmzm2g476652_t01 0

grmzm2g476685_t03 0

grmzm2g476699_t01 0

grmzm2g476762_t01 0

grmzm2g476843_t02 0

grmzm2g476898_t01 0

grmzm2g476914_t01 0

grmzm2g476933_t01 0

grmzm2g476973_t01 0

grmzm2g477084_t01 0

grmzm2g477146_t01 0

grmzm2g477205_t03 0

grmzm2g477236_t02 0

grmzm2g477325_t01 0

grmzm2g477340_t02 0

grmzm2g477503_t01 0

grmzm2g477603_t01 0

grmzm2g477694_t03 0

grmzm2g477741_t01 0

grmzm2g477743_t01 1.12

grmzm2g477829_t01 0

grmzm2g477846_t01 0

grmzm2g477847_t01 0

grmzm2g477869_t01 0

grmzm2g477872_t01 0

grmzm2g477879_t01 0

grmzm2g477917_t01 0

grmzm2g478160_t01 0

grmzm2g478212_t01 0

grmzm2g478370_t01 0

grmzm2g478382_t01 0

grmzm2g478414_t04 0

grmzm2g478417_t01 0

grmzm2g478558_t01 0

grmzm2g478568_t01 0

grmzm2g478621_t01 0

grmzm2g478624_t01 0

grmzm2g478664_t01 0

grmzm2g478709_t01 0

grmzm2g478798_t01 0

grmzm2g478876_t01 0

grmzm2g478877_t01 0

grmzm2g479000_t01 0

grmzm2g479038_t01 0

grmzm2g479110_t01 0

grmzm2g479112_t01 0

grmzm2g479125_t01 0

grmzm2g479163_t01 0.9

grmzm2g479243_t01 0

grmzm2g479249_t01 0

grmzm2g479340_t01 0

grmzm2g479423_t05 0

grmzm2g479529_t01 0

grmzm2g479586_t01 0

grmzm2g479608_t01 0

grmzm2g479665_t01 0

grmzm2g479684_t01 0

grmzm2g479703_t01 0

grmzm2g479712_t01 1.66

grmzm2g479744_t01 0

grmzm2g479760_t01 0

grmzm2g479885_t01 0

grmzm2g479906_t02 0

grmzm2g479987_t01 0

grmzm2g480002_t01 0

grmzm2g480068_t01 0

grmzm2g480171_t02 0

grmzm2g480192_t01 0

grmzm2g480356_t01 0

grmzm2g480480_t03 0

grmzm2g480516_t02 0

grmzm2g480607_t01 0

grmzm2g480620_t01 0

grmzm2g480712_t01 0

grmzm2g480740_t01 0

grmzm2g480809_t01 0

grmzm2g480850_t01 0

grmzm2g480856_t01 0

grmzm2g481005_t01 1.03

grmzm2g481033_t01 0

grmzm2g481069_t01 1.04

grmzm2g481103_t04 0

grmzm2g481163_t01 0

grmzm2g481261_t01 0

grmzm2g481291_t01 0

grmzm2g481373_t01 0

grmzm2g481440_t01 0

grmzm2g481449_t01 0

grmzm2g481525_t01 0

grmzm2g481548_t01 0

grmzm2g481604_t01 0

grmzm2g481740_t01 0

grmzm2g481755_t03 0

grmzm2g481843_t01 0

grmzm2g481904_t01 0

grmzm2g482256_t02 0

grmzm2g482337_t01 0

grmzm2g482720_t01 0

grmzm2g484344_t01 0

grmzm2g484452_t01 0.86

grmzm2g485184_t01 0

grmzm2g485559_t01 0

grmzm2g487629_t02 0.89

grmzm2g494514_t01 0

grmzm2g504401_t01 0

grmzm2g505380_t01 0

grmzm2g506062_t01 0

grmzm2g509439_t01 0

grmzm2g509876_t01 0

grmzm2g513649_t01 0

grmzm2g518764_t01 0

grmzm2g520535_t01 0

grmzm2g529313_t01 0

grmzm2g531230_t01 -1.52

grmzm2g531738_t01 0

grmzm2g534593_t01 0

grmzm2g537291_t01 0

grmzm2g539821_t01 0

grmzm2g542205_t01 0

grmzm2g542227_t01 0

grmzm2g543629_t01 0

grmzm2g546172_t01 0

grmzm2g546254_t01 0

grmzm2g547542_t01 0

grmzm2g550472_t01 0

grmzm2g551197_t01 0

grmzm2g553607_t01 0

grmzm2g553687_t01 0

grmzm2g553846_t01 0

grmzm2g555108_t01 0

grmzm2g567897_t01 0

grmzm2g569855_t02 0

grmzm2g573732_t01 0

grmzm2g574534_t02 0

grmzm2g574858_t02 0

grmzm2g576752_t02 0.99

grmzm2g583779_t01 0

grmzm2g700046_t01 -1.63

grmzm2g700057_t01 0

grmzm2g700128_t01 0

grmzm2g700313_t01 -0.87

grmzm2g700427_t01 0

grmzm2g700443_t01 0

grmzm2g700512_t01 0

grmzm2g700607_t01 0

grmzm2g700655_t01 0

grmzm2g700665_t01 0

grmzm2g700682_t01 0

grmzm2g700683_t01 0

grmzm2g700759_t02 0

grmzm2g700926_t01 0

grmzm2g701004_t02 0

grmzm2g701055_t01 0

grmzm2g701063_t01 0

grmzm2g701082_t01 0

grmzm2g701096_t01 0

grmzm2g701108_t01 0

grmzm2g701169_t01 0

grmzm2g701180_t01 0

grmzm2g701201_t04 0

grmzm2g701204_t03 0

grmzm2g701206_t01 0

grmzm2g701207_t03 0

grmzm2g701218_t01 1.42

grmzm2g701221_t01 0

grmzm2g701297_t01 0

grmzm2g701373_t01 0

grmzm2g701486_t01 0

grmzm2g701551_t01 0

grmzm2g701576_t01 0

grmzm2g701641_t01 0

grmzm2g701684_t01 0

grmzm2g701688_t01 0

grmzm2g701746_t01 0

grmzm2g701801_t01 0

grmzm2g702026_t01 0

grmzm2g702036_t01 0

grmzm2g702059_t01 0

grmzm2g702184_t01 0

grmzm2g702302_t01 0

grmzm2g702426_t01 0

grmzm2g702490_t02 0

grmzm2g702573_t01 0

grmzm2g702864_t01 0

grmzm2g702880_t01 0

grmzm2g702991_t01 0

grmzm2g703231_t02 0

grmzm2g703296_t01 0

grmzm2g703303_t02 0

grmzm2g703415_t01 0

grmzm2g703490_t01 0

grmzm2g703491_t01 0

grmzm2g703565_t01 0

grmzm2g703582_t01 0

grmzm2g703600_t01 0

grmzm2g703749_t01 0

grmzm2g703858_t01 0

grmzm2g703893_t01 0

grmzm2g703918_t01 0

grmzm2g703928_t01 0

grmzm2g704005_t01 0

grmzm2g704020_t01 0

grmzm2g704032_t01 0

grmzm2g704039_t01 0

grmzm2g704053_t02 0

grmzm2g704098_t01 0

grmzm2g704277_t02 0

grmzm2g704301_t02 0

grmzm2g704338_t02 0

grmzm2g704347_t01 0
