# Supplementary material for: Gene Mapping via Bulked Segregant RNA-Seq (BSR-Seq)
Source: PLoS One. 2012 May 7;7(5):e36406. doi: 10.1371/journal.pone.0036406 (PMC3346754; doi:10.1371/journal.pone.0036406)
Supplement: Table S4 — (DOC) [file pone.0036406.s008.doc]

Table S4. Summary of SNP discovery

| Chr | No. SNPs | No. SNPs for BSA |
| --- | --- | --- |
| chr0* | 69 | 26 |
| chr1 | 10,250 | 6,633 |
| chr2 | 7,761 | 4,784 |
| chr3 | 7,100 | 4,478 |
| chr4 | 5,332 | 3,281 |
| chr5 | 8,650 | 5,551 |
| chr6 | 5,259 | 3,399 |
| chr7 | 5,382 | 3,451 |
| chr8 | 5,577 | 3,502 |
| chr9 | 5,016 | 3,150 |
| chr10 | 4,525 | 2,917 |
| Total | 64,921 | 41,172 |

* unknown chromosomes; sequence contigs were not mapped to any 10 maize chromosome
