# Supplementary material for: Gene Mapping via Bulked Segregant RNA-Seq (BSR-Seq)
Source: PLoS One. 2012 May 7;7(5):e36406. doi: 10.1371/journal.pone.0036406 (PMC3346754; doi:10.1371/journal.pone.0036406)
Supplement: Table S6 — (DOC) [file pone.0036406.s010.doc]

Table S6. Expression summary of genes involved in the biosynthesis of very-long-chain fatty acids

| Maize_gene | Associated_gene | gl3mut.RPKM | gl3wt.RPKM | gl3wt:mut_log2FC | gl3wt:mut_sig |
| --- | --- | --- | --- | --- | --- |
| GRMZM2G003501 | GLOSSY4A | 34.468 | 152.888 | 2.17 | yes |
| GRMZM2G101875 | CER8 | 69.978 | 208.233 | 1.6 | yes |
| GRMZM2G162434 | GLOSSY3 | 7.101 | 13.287 | 0.92 | yes |
| GRMZM2G323830 | MAH1 | 27.862 | 14.956 | -0.87 | yes |
| AC233893.1_FG003 | CER6 | 25.131 | 11.7 | -1.07 | yes |
| GRMZM2G029912 | CER3/WAX2/YRE/FLP1 | 6.198 | 2.821 | -1.1 | yes |
| GRMZM2G438622 | CER7 | 5.122 | 8.909 | 0.81 | no |
| GRMZM2G152127 | CER8 | 9.563 | 15.572 | 0.72 | no |
| GRMZM2G406603 | FATB | 33.346 | 51.475 | 0.65 | no |
| GRMZM2G075255 | CER1 | 4.374 | 6.41 | 0.57 | no |
| GRMZM2G481843 | CER10 | 176.668 | 249.049 | 0.52 | no |
| GRMZM2G177812 | WBC11 | 143.601 | 197.211 | 0.48 | no |
| GRMZM2G114642 | GLOSSY1/CER3 | 99.62 | 136.506 | 0.48 | no |
| GRMZM2G157564 | CER5 | 126.032 | 160.66 | 0.37 | no |
| GRMZM2G160730 | GLOSSY15 | 6.086 | 7.092 | 0.24 | no |
| AC205703.4_FG007 | GLOSSY8A | 53.838 | 54.748 | 0.05 | no |
| GRMZM2G164974 | CER6 | 181.249 | 178.587 | 0 | no |
| GRMZM2G087323 | GLOSSY8B | 13.269 | 12.768 | -0.03 | no |
| GRMZM2G393897 | CER6 | 9.616 | 8.735 | -0.11 | no |
| GRMZM2G007489 | FATB | 25.568 | 17.166 | -0.55 | no |
| GRMZM2G098239 | GLOSSY2_CER2 | 122.763 | 75.755 | -0.67 | no |
| GRMZM2G083526 | CER3/WAX2/YRE/FLP1 | 20.667 | 11.72 | -0.79 | no |
| GRMZM2G066578 | CER1 | 1.318 | 0.758 | NA | NA |
| GRMZM2G099097 | CER1 | 0.606 | 1.177 | NA | NA |
| GRMZM2G120938 | CER4 | 0.624 | 0.317 | NA | NA |
| GRMZM2G005216 | CER7 | 0 | 0 | NA | NA |
| GRMZM2G158586 | CER7 | 0 | 0.155 | NA | NA |
| GRMZM2G096952 | WBC11 | 0 | 0 | NA | NA |
| GRMZM2G177314 | WBC11 | 0.607 | 0.617 | NA | NA |
| GRMZM2G077375 | WSD1 | 0.329 | 0.134 | NA | NA |
