# Supplementary material for: Gene Mapping via Bulked Segregant RNA-Seq (BSR-Seq)
Source: PLoS One. 2012 May 7;7(5):e36406. doi: 10.1371/journal.pone.0036406 (PMC3346754; doi:10.1371/journal.pone.0036406)
Supplement: Table S7 — (DOC) [file pone.0036406.s011.doc]

Table S7. Number of differentially expressed genes in each chromosome

| Chr | Down-regulation | Up-regulation | Other informative genes | Down-to-Up* | Down%** |
| --- | --- | --- | --- | --- | --- |
| 1 | 53 | 118 | 2,492 | 0.4 | 2.0 |
| 2 | 40 | 61 | 1,845 | 0.7 | 2.1 |
| 3 | 47 | 64 | 1,693 | 0.7 | 2.6 |
| 4 | 78 | 50 | 1,544 | 1.6 | 4.7 |
| 5 | 43 | 71 | 1,919 | 0.6 | 2.1 |
| 6 | 43 | 42 | 1,322 | 1.0 | 3.1 |
| 7 | 39 | 70 | 1,216 | 0.6 | 2.9 |
| 8 | 31 | 78 | 1,387 | 0.4 | 2.1 |
| 9 | 42 | 47 | 1,165 | 0.9 | 3.3 |
| 10 | 29 | 31 | 1,043 | 0.9 | 2.6 |
| Total | 446 | 633 | 15,647 | 0.7 | 2.7 |

* The number of down-regulated genes to that of up-regulated genes

** The percentage of down-regulated genes in total informative genes, including the down-regulated genes, the up-regulated genes and other genes, in the chromosome.
